# An Efficient One-Pot Catalyzed Synthesis of 2,4-Disubstituted 5-Nitroimidazoles Displaying Antiparasitic and Antibacterial Activities

**DOI:** 10.3390/molecules22081278

**Published:** 2017-08-03

**Authors:** Fanny Mathias, Youssef Kabri, Liliane Okdah, Carole Di Giorgio, Jean-Marc Rolain, Cédric Spitz, Maxime D. Crozet, Patrice Vanelle

**Affiliations:** 1 Aix Marseille University, Institut de Chimie Radicalaire ICR, UMR CNRS 7273, Laboratoire de Pharmaco-Chimie Radicalaire, Faculté de Pharmacie, 27 Boulevard Jean Moulin—CS 30064, 13385 Marseille CEDEX 05, France; fanny.mathias@laposte.net (F.M.); youssef.kabri@univ-amu.fr (Y.K.); cedric.spitz@univ-amu.fr (C.S.); maxime.crozet@univ-amu.fr (M.D.C.); 2 IHU Méditerranée Infection, Aix Marseille University, Unité de Recherche sur les Maladies Infectieuses et Tropicales Emergentes, URMITE UMR 63, CNRS 7278, IRD 198, Inserm 1095, Faculté de Médecine de la Timone, 19-21 Boulevard Jean Moulin, 13005 Marseille, France; liliane.okdah@hotmail.com (L.O.); jean-marc.rolain@univ-amu.fr (J.-M.R.); 3 Aix Marseille University, CNRS, IRD, Avignon Université, IMBE UMR 7263, Laboratoire de Mutagénèse Environnementale, 13385 Marseille, France; carole.di-giorgio@univ-amu.fr

**Keywords:** 5-nitroimidazole, Suzuki-Miyaura/Sonogashira, regioselectivity, microwave, antiparasitic, antibacterial

## Abstract

A one-pot regioselective bis-Suzuki-Miyaura or Suzuki-Miyaura/Sonogashira reaction on 2,4-dibromo-1-methyl-5-nitro-1*H*-imidazole under microwave heating was developed. This method is applicable to a wide range of (hetero)arylboronic acids and terminal alkynes. Additionally, this approach provides a simple and efficient way to synthesize 2,4-disubstituted 5-nitroimidazole derivatives with antibacterial and antiparasitic properties.

## 1. Introduction

Nitroimidazole drugs show a wide range of activities against parasites and anaerobic bacteria. These activities vary according to the nature of the substituents and the position of the nitro group [1,2,3,4,5,6,7]. Metronidazole (mtz) appears on the WHO Model List of Essential Medicines and is today the most commonly used 5-nitroimidazole compound worldwide.

5-Nitroimidazoles are prodrugs which need to be activated by reduction of their nitro group. Reduced metronidazole forms covalent bonds to DNA macromolecules, leading to the loss of helical structure and irreversible breaking [8]. The reduction of these compounds involves several anaerobic enzymatic pathways such as ferredoxin oxidoreductase [9], nitroreductase [10] and thioredoxin reductase [11].

Resistance to metronidazole has emerged, but despite studies on *Trichomonas vaginalis* [9,12,13,14,15], *Giardia intestinalis* [16,17,18], and anaerobic bacteria [19,20,21,22,23], the mechanism is not yet fully understood. Such resistance leads physicians to use higher doses of metronidazole and longer courses of treatment to improve activity [24].

However, higher doses and longer regimens may promote side effects and decrease compliance. Although the most common side effects of metronidazole treatment are mild (headache, vertigo, nausea, a metallic taste in the mouth and an Antabuse-like effect with alcohol) [25], other more serious adverse effects have been documented, such as mutagenicity on bacteria [26,27,28,29,30,31] and carcinogenicity on mice and rats [32]. Moreover, in recent decades, an increasing number of anaerobic bacterial pathogens have been observed to cause severe diarrhea and enterocolitis [33,34,35,36].

Therefore, there is an ongoing search for alternative 5-nitroimidazoles effective on anaerobic-resistant strains without serious adverse effects [37,38,39,40,41,42,43,44,45,46,47,48]. Following the work of Walsh et al. in 1987 [49], we focused our research on pharmacomodulation of the 2- and 4-positions of the 5-nitroimidazole scaffold [50].

Palladium-catalyzed reactions, particularly Suzuki-Miyaura cross-coupling, are widely applied in the synthesis of bioactive complex molecules, offering mild reaction conditions and compatibility with a broad range of functional groups [51,52]. Moreover, some Suzuki-Miyaura cross-coupling conditions allow regioselective cross-coupling on polyhalogenated heterocycles [53,54], leaving a halogenated α position unchanged and able to react in a second step. This enables a wide range of compounds with the same starting material.

The regioselectivity in the Suzuki-Miyaura reaction is driven by the electrophilic properties of the halogenated carbon. During the oxidative addition step, the palladium catalyst which is a nucleophilic species, specifically links the most electrophilic halogenated carbon [53,54,55]. The nitro group, essential for medicinal properties, can also act as a chemical directing group during synthesis [56]. Thus, the nitro group in position 5 can significantly increase the electrophilic properties of the C-4, making this position the most reactive toward cross-coupling reactions, although on the naked imidazole ring, the C-2 position was described as the most reactive [57,58].

As part of our research program centered on the design and synthesis of novel 5-nitroimidazole compounds [40,43,48,50,59,60], we decided to exploit this particular reactivity of the 5-nitroimidazole series toward cross-coupling reactions, seeking a rapid and versatile pathway for the synthesis of 2,4-disubstitued 5-nitroimidazole derivatives. Therefore, we report herein a regioselective Suzuki-Miyaura cross-coupling between 2,4-dibromo-1-methyl-5-nitro-1*H*-imidazole (**2**) and aryl or heteroarylboronic acids resulting in the formation of 2-bromo-4-substituted-1-methyl-5-nitroimidazoles which can then undergo a second (Suzuki-Miyaura or Sonogashira) cross-coupling reaction to obtain 2,4-disubstituted 5-nitroimidazoles during a sequential one-pot process.

Finally, the antiparasitic activity of the obtained 5-nitroimidazole derivatives was assessed against *T. vaginalis* and anaerobic bacteria of the genus *Clostridium* and *Bacillus*.

## 2. Results

### 2.1. Chemistry

**Scheme 1:**
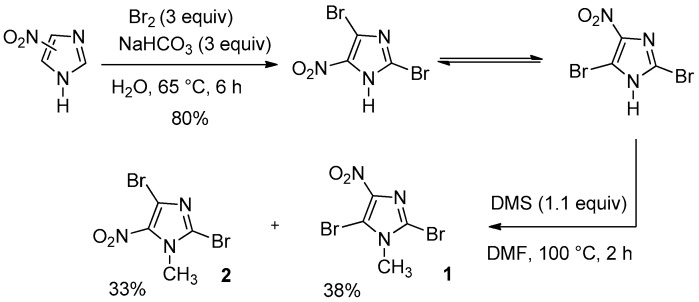
Synthesis of 2,4-dibromo-1-methyl-5-nitro-1*H*-imidazole (**2**).

The synthesis of 2,4-dibromo-1-methyl-5-nitro-1*H*-imidazole (**2**) was realized from the commercial product 4(5)-nitroimidazole in two sequential steps, as shown in Scheme 1. The first step consisted of bromination with elemental bromine in water and in the presence of sodium hydrogen carbonate, according to Pedada et al. [61]. The 2,4(5)-dibromo-5(4)-nitroimidazole mixture was then alkylated by dimethyl sulphate (DMS) in DMF leading to two isomeric products **1** and **2** in 71% overall yield.

Our particular aim in exploring the reactivity of 2,4-dibromo-1-methyl-5-nitro-1*H*-imidazole (**2**) in Suzuki-Miyaura reactions was to test the regioselectivity of this type of reaction toward the halogenated positions C-2 and C-4.

We began our study by optimizing the regioselective coupling reaction of compound **2** with phenylboronic acid (1.1 equiv.) in the presence of Pd(PPh_3_)_4_ (0.04 equiv.) as catalyst, Na_2_CO_3_ (3 equiv.) as base, in a mixture of DME/EtOH/H_2_O (3/1/1) as solvent. First, the reaction was performed at room temperature, and after 1.5 h, no conversion of compound **2** was observed (Table 1, entry 1). When the temperature was increased to 60 °C, after 48 h of reaction, a mixture of compounds **2**/**3a**/**4**/**5** was observed with both low conversion and selectivity (LC/MS ratio 25/28/26/21%, respectively) (Table 1, entry 2). In these conditions, the reactivity of positions C-2 and C-4 was similar. The use of microwave irradiation at 80 °C for 1 h (Table 1, entry 4) allowed better conversion of compound **2** in the Suzuki-Miyaura reaction, with lower selectivity and improved yield of the dicoupled compound **5**. Next, we examined the influence of other palladium sources such as PdCl_2_(PPh_3_)_2_, Pd(DPPF)Cl_2_ and Pd(OAc)_2_. With PdCl_2_(PPh_3_)_2_ and Pd(DPPF)Cl_2_, low conversion was observed and there was no improvement in terms of selectivity (Table 1, entries 5 and 6). To our satisfaction, good conversion and selectivity were observed with Pd(OAc)_2_ and compound **3a** was obtained in 60% yield (Table 1, entry 7). More importantly, the use of 1.3 equiv. of phenylboronic acid for a longer time afforded compound **3a** in better yield (64%, Table 1, entry 12). No yield improvement for compound **3a** was observed when we carried out the reaction in presence of Xantphos (Table 1, entry 8) or increased the temperature (Table 1, entries 10 and 11). Moderate selectivity was obtained when PPh_3_ was added (Table 1, entry 9). These trials with different catalysts revealed that regioselectivity depends on the catalytic system: Pd(OAc)_2_ performed best, both on conversion and selectivity, in our reaction conditions.

So, the best reaction conditions were 1 equivalent of imidazole **2**, 1.3 equivalents of phenyl-boronic acid, 0.04 equivalents of palladium acetate and 3 equivalents of sodium carbonate in DME/EtOH/H_2_O (3/1/1) for 2 h at 60 °C under microwave irradiation. With the optimized reaction conditions in hand, we assessed the scope and limitations of the regioselective Suzuki-Miyaura reaction of 2,4-dibromo-1-methyl-5-nitro-1*H*-imidazole (**2**). A variety of substituted arylboronic acids (with both electron-donating or electron-withdrawing groups) were used to give the corresponding coupling products **3a**–**i** in moderate to good yields (55–65%). However, the use of hydroxymethylphenylboronic acid (**3i**) and boronic acids substituted by withdrawing groups such as NO_2_ or CF_3_ required a temperature increase to 65 °C (Table 2, Compounds **3c**, **3d**). On the other hand, the use of heteroarylboronic acids (pyridine-3-ylboronic acid, 5-methylthiophen-2-ylboronic acid) led to very low conversion under our optimal conditions. Increasing the temperature in these cases improved the yield of the corresponding dicoupled product.

**Figure 1:**
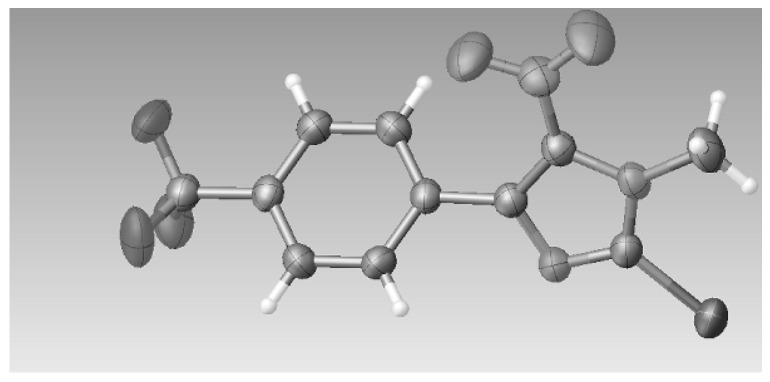
X-ray crystal structure of compound **3c**.

The X-ray crystal structure (Figure 1) was determined for compound **3c** to confirm the regioselective Suzuki-Miyaura reaction on 4-position of 2,4-dibromo-1-methyl-5-nitro-1*H*-imidazole (**2**).

Following optimization of the regioselective Suzuki-Miyaura reaction, we investigated the one-pot regioselective bis-Suzuki-Miyaura reactions of 2,4-dibromo-1-methyl-5-nitro-1*H*-imidazole (**2**).

The sequential one-pot two-step bis-Suzuki-Miyaura reactions of compound **2** were studied using phenylboronic acid and 4-methoxyphenylboronic acid under microwave irradiation. Phenylboronic acid can be expected to react selectively at the C-4 position, with the subsequent addition of 4-methoxyphenylboronic acid leading to reaction at the C-2 position of compound **2** (Table 3).

The first step was performed in the presence of phenylboronic acid (1.3 equiv.), Pd(OAc)_2_ (0.04 equiv.), Na_2_CO_3_ (3 equiv.) in DME/EtOH/H_2_O (3/1/1) at 60 °C under microwave irradiation for 2 h. In the second step, the reaction was carried out without addition of a palladium catalyst and in presence of 4-methoxyphenylboronic acid (1.3 equiv.), Na_2_CO_3_ (3 equiv.) at 60 °C under microwave irradiation for 1 h, giving the expected disubstituted imidazole **6** in moderate yield (22%, Table 3, entry 1). These conditions did not allow a complete conversion of the intermediate compound **3a**, which remains the majority product with a yield of 47%. Adding Pd(OAc)_2_ (0.04 equiv.) in the second step increased the yield of compound **6** to 30% (Table 3, entry 2). Performing the reaction at 110 °C increased the yield of compound **6** to 40% (Table 3, entry 3). Interestingly, using Pd(PPh_3_)_4_ (0.04 equiv.) or adding only PPh_3_ (0.1 equiv.) in the second step led to complete conversion of the intermediate **3a** and the corresponding product **6** isolated respectively in 60 and 64% yields (Table 3, entries 4 and 5). Note that, when the reaction temperature was reduced to 90 °C, incomplete conversion of the intermediate **3a** was observed (according to LC/MS analysis).

**Scheme 2:**
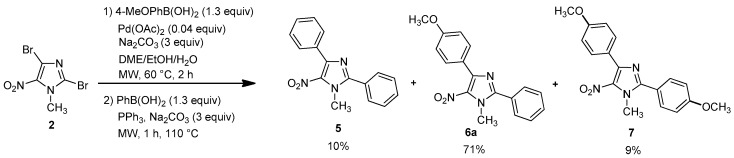
One-pot regioselective bis-Suzuki-Miyaura reaction.

In addition, the use of 4-methoxyphenylboronic acid in the first step and phenylboronic acid in the second step gave the corresponding product **6a** in better yield (71%, Scheme 2).

With optimized conditions established, the scope of the one-pot regioselective bis-Suzuki-Miyaura reaction was explored using a variety of (hetero)arylboronic acids. We chose 4-methoxy-phenylboronic acid for the first step and different boronic acids for the second step (Table 4). This approach enabled a broad range of (hetero)arylboronic acids bearing an electron-donating or withdrawing group to be used under standard conditions, and provided the disubstituted imidazole derivatives **6a**–**j** in moderate to good yields (Table 4, 50–71%).

In view of these encouraging results, we then turned our attention to the sequential one-pot regioselective Suzuki-Miyaura/Sonogashira reaction of 2,4-dibromo-1-methyl-5-nitro-1*H*-imidazole (**2**). Having optimized reaction conditions for the regioselective Suzuki-Miyaura coupling, we decided to develop the one-pot Suzuki-Miyaura/Sonogashira sequence.

**Scheme 3:**
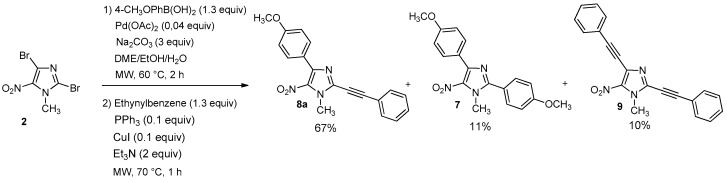
One-pot regioselective Suzuki-Miyaura/Sonogashira reaction.

Thus, based on the already optimized regioselective Suzuki-Miyaura reaction conditions and the classical conditions for Sonogashira reaction, we prepared the disubstituted 5-nitroimidazole derivative **8a** from 4-methoxyphenylboronic acid and ethynylbenzene (Scheme 3). The first step was performed in presence of 4-methoxyphenylboronic acid (1.3 equiv.), Na_2_CO_3_ (3 equiv.), Pd(OAc)_2_ (0.04 equiv.) in a mixture of DME/EtOH/H_2_O (3/1/1) under microwave irradiation for 2 h at 60 °C. After cooling, ethynylbenzene (1.3 equiv.), PPh_3_ (0.1 equiv.), CuI (0.1 equiv.) and Et_3_N (2 equiv.) were added and the mixture was heated again under microwave irradiation for 1 h at 70 °C. This sequence led to the desired compound **8a** in good yield (67%) and compounds **7** and **9** in 11% and 10% yields, respectively (Scheme 3).

The scope of this Suzuki-Miyaura/Sonogashira sequence was then investigated using the optimized procedure. We were pleased to observe the formation of the disubstituted imidazole derivatives **8a**–**j** in moderate to good yields when 4-methoxyphenylboronic acid was used in the first step and various terminal alkynes in the second step (Table 5). Both electron-poor (compounds **8c**, **8h** and **8i**) and electron-rich (compounds **8b**, **8d** and **8f**) substituents on aryl alkynes are well tolerated. Interestingly, this strategy was also tolerant to cycloalkyl alkynes (compounds **8e** and **8j**) and to a hydroxyl moiety (compound **8g**).

### 2.2. Antimicrobial Activity

The antimicrobial activity of the newly synthesized products was evaluated against anaerobic Gram positive bacteria of the genus *Clostridium* and *Bacillus*. They were also evaluated for their antiparasitic activity against *T. vaginalis*. Metronidazole (mtz) was used as control drug to evaluate the potency of the tested compounds under the same conditions.

#### 2.2.1. Antibacterial Activity

Antibacterial activity was determined by the disc diffusion method, according to European committee on Antimicrobial Susceptibility Testing (EUCAST) recommendations [62]. We first screened the 27 molecules synthesized for their antibacterial activity against *C. butyricum*, *C. perfringens*, *C. neonatale*, *C. difficile*, *B. circulans* and *E. coli.* The observed data on the antibacterial activity of the 3 series of compounds are reported for compounds **3a**–**3i** in Table 6, for compounds **6a**–**6j** in Table 7, and for compounds **8a**–**8j** in Table 8. Molecules are considered active when the inhibition diameter is larger than 20 mm, and very active when it is larger than the inhibition diameter of metronidazole.

Results show that none of the molecules tested, including metronidazole, were active on facultative anaerobic bacteria (*B. circulans*) and aerobic bacteria (*E. coli*). Indeed, to be active, 5-nitro-imidazole compounds must undergo a reduction of their nitro group which can only be carried out in an anaerobic environment [2]. If the molecules were active on aerobic bacteria, this would suggest another mechanism of action.

For the anaerobic bacteria, it was observed that compounds **3b**, **3e**–**i** (Table 6), with a bromine in 2-position, possessed an activity on *C. butyricum Nec 0*, *C. neonatale* and *C. difficile* equivalent or higher than the standard metronidazole. On the other hand, compounds **6a**–**j** (Table 7) with an aryl group in 2-position had a lower activity than compounds **3a**–**j**. Only compound **6a** was active on *C. butyricum Nec 0*, *C. neonatale* and *C. difficile*. Compounds **8a**–**j**, the last series, with an alkyne in 2-position, had a lower activity than the molecules of the first two series (Table 8).

The second step consisted in testing the most active molecules, **3b**, **3e**–**i** on other particularly virulent bacteria such as *B. cereus*, *C. beijerenckii*, *C. tetani* and *C. difficile* 027 (Table 9). The first results showed that a bromine in 2-position could improve the activity of our compounds, so, we decided to test the starting compound **2** with two bromine atoms in 2 and 4-position. Unfortunately, this last compound did not exhibit any activity (Table 9). Whatever the nature of the aryl group in 4-position, the molecules with a bromine in 2-position (**3b**, **3e**–**3i**) had promising activity on *C. butyricum Nec 0*, *Nec 1*, *Nec 8* and on *C. difficile* O27. One of them, compound **3g**, with a trimethoxyphenyl group in 4-position, exhibited higher activity against *C. butyricum Nec 8* than metronidazole. It was thus observed from the investigation on anaerobic bacteria screening data that the combination of a bromine atom in 2-position and an aryl group in 4-position enhanced the compounds’ antibacterial activities.

#### 2.2.2. Antiparasitic Activity

We selected the six molecules **3b**, **3e**–**3i** showing the most promising activities against anaerobic bacteria, and assessed their therapeutic interest on *T. vaginalis* by determining minimum inhibitory concentration (MIC, Table 10). We also performed a cytotoxicity evaluation via the Neutral Red Uptake (NRU) toxicity test [63] on CHO-K1 Chinese Hamster Ovary cells (ATCC CCL61) to detect cytotoxic concentrations of 50% (CC_50_). This procedure enabled us to determine the selectivity index of these 6 compounds (Table 10), all of which are shown to have a significantly higher activity, with lower MICs, than metronidazole. On the other hand, they have a significant and higher toxicity than metronidazole, especially for compounds **3b**, **3f** and **3i** with methoxyphenyl, fluorophenyl or hydroxymethylphenyl groups in the 4-position.

#### 2.2.3. Cytotoxicity Evaluation

The molecules with an aryl (**6a**–**j**) or alkyne (**8a**–**j**) group at 2-position show cytotoxicity equivalent to that of molecules with a bromine in 2-position (**3a**–**i**) (Table 11). The homogeneity of the cytotoxicity of our molecules in the three different series shows the importance of the substituent at the 2-position of the imidazole nucleus.

## 3. Experimental Section

### 3.1. General Information

Melting points were determined on Büchi B-540 (BÜCHI Labortechnik AG, Flawil, Switzerland) and are uncorrected. Elemental analyses and HRMS were carried out at the Spectropole, Faculté des Sciences et Techniques de Saint-Jérôme, Marseille. Elemental analyses were performed on a EA1112 instrument (Thermo Finnigan, San Jose, CA, USA). HRMS spectra were recorded on a QStar Elite (Applied Biosystems SCIEX, Foster City, CA, USA) spectrometer. PEG was a matrix for HRMS. LC/MS analyses were performed at the Faculté de Pharmacie de Marseille on an Accela High System^®^ chain U-HPLC coupled with a Thermo MSQ Plus^®^ simple quadrupole. A Thermo Hypersil Gold^®^ 50 × 2.1 mm chromatographic column was used (SiO_2_ C18) with 1.9 µm diameter particles. Analysis is 8 min running, with an MeOH/H_2_O eluent gradient from 50/50 to 95/05. Both ^1^H-NMR spectra (250 MHz, reference CDCl_3_ δ = 7.26, [*d*_6_]DMSO δ = 2.50) and ^13^C-NMR spectra (62.5 MHz, reference CDCl_3_ δ = 77.0, [*d*_6_]DMSO δ = 39.7) were recorded at 24 °C on an ARX 250 spectrometer (Bruker, Wissembourg, France) in CDCl_3_ and [*d*_6_]DMSO solvents at the Faculté de Pharmacie de Marseille. Solvents were dried by conventional methods. The following adsorbent was used for column chromatography: silica gel 60 (Merck, Darmstadt, Germany, particle size 0.063–0.200 mm, 70–230 mesh ASTM). TLC was performed on 5 cm × 10 cm aluminium plates coated with silica gel 60F-254 (Merck) in an appropriate eluent. Microwave reactions were performed with an Initiator microwave oven (Biotage^®^, Charlotte, NC, USA) using 2–5 mL sealed vials; temperatures were measured with an IR-sensor and reaction times are given as hold times.

### 3.2. Chemistry

#### 3.2.1. 2,4-Dibromo-1-methyl-5-nitro-1*H*-imidazole (**2**)

First step: to a solution of 4(5)-nitro-1*H*-imidazole (20 g, 177 mmol), NaHCO_3_ (44.6 g, 3 equiv., 531 mmol) in water (120 mL) was added dropwise at 0 °C dibromine (27 mL, 3 equiv., 531 mmol) and the reaction mixture was heated at 65 °C for 6 h. After cooling, the solution was poured into bath of ice. A yellow precipitate appeared and was filtered, washed with water (3 × 100 mL) and dried in vacuum drying oven (dessicator cabinet). The aqueous layer was extracted with ethyle acetate (3 × 100 mL) and the organic layer was washed with brine (3 × 100 mL), dried over Na_2_SO_4_ and evaporated. Yellow solid was obtained and added to the precipitate to give 36.3 g (80%) of 2,4(5)-dibromo-5(4)-nitroimidazole.

Second step; to a solution of 2,4(5)-dibromo-5(4)-nitroimidazole (34 g, 126 mmol) in DMF (80 mL) was added dropwise at 0 °C dimethyl sulfate (13.1 mL, 1.1 equiv., 138 mmol) and the reaction mixture was heated at 100 °C for 2 h. After cooling, the solution was treated with a saturated solution of sodium hydrogen carbonate at 0 °C. A white precipitate appeared and was filtered. One part of the precipitate was solubilised in ethyl acetate and evaporated after filtration. The precipitate not dissolved was the 2,5-dibromo-1-methyl-4-nitro-1*H*-imidazole (**1**, 38%, 14 g), M.p. 192 °C (lit. [26] 204–205 °C). ^1^H-NMR (CDCl_3_): δ = 3.75 (s, 3H, NCH_3_) ppm. ^13^C-NMR (CDCl_3_): δ = 35.0 (NCH_3_), 106.7 (C_Ar_), 120.4 (C_Ar_) ppm (CNO_2_ was not visible under these conditions). The aqueous layer was extracted with ethyl acetate (3 × 100 mL) and the organic layer was washed with brine (3 × 100 mL), dried over Na_2_SO_4_ and evaporated. The crude product was purified by column chromatography [silica gel, petroleum ether/ethyl acetate (9/1) and the 2,4-dibromo-1-methyl-5-nitro-1*H*-imidazole (**2**) was obtained in 33% in yield (12.1 g). M.p. 167 °C (lit. [42] 160–161 °C). ^1^H-NMR (CDCl_3_): δ = 4.02 (s, 3H, NCH_3_) ppm. ^13^C-NMR (CDCl_3_): δ = 37.2 (NCH_3_), 119.8 (C_Ar_), 126.4 (C_Ar_) ppm (CNO_2_ was not visible under these conditions).

#### 3.2.2. General Procedure for the Regioselective Suzuki-Miyaura Coupling Reaction

A solution of 2,4-dibromo-1-methyl-5-nitro-1*H*-imidazole (**2**, 0.1 g, 0.35 mmol), boronic acid (0.46 mmol, 1.3 equiv.), Pd(OAc)_2_ (3 mg, 0.014 mmol, 0.04 equiv.), Na_2_CO_3_ (0.11 g, 1.05 mmol, 3 equiv.), in a DME/EtOH/H_2_O mixture (3/1/1, 5 mL) was heated at 60 °C under microwave irradiation for 2 h. After cooling, 60 mL of water were added and the solution was extracted with dichloromethane (3 × 50 mL). The organic layer was washed with water (3 × 100 mL), dried over Na_2_SO_4_ and evaporated. The crude product was purified by column chromatography [silica gel, petroleum ether/ethyl acetate (9/1), (1/1 for **3i**), cyclohexane/ethyl acetate (9/1 for **3a**)] and recrystallized from propan-2-ol.

2-Bromo-1-methyl-5-nitro-4-phenyl-1*H*-imidazole (**3a**): Yield 64% (63 mg); yellow solid; M.p. 96 °C. ^1^H-NMR (CDCl_3_): δ = 4.01 (s, 3H, NCH_3_), 7.43–7.45 (m, 3H, 3 Ar-H), 7.73–7.77 (m, 2H, 2 Ar-H) ppm. ^13^C-NMR (CDCl_3_): δ = 36.5 (NCH_3_), 126.6 (C_Ar_), 128.1 (2 CH_Ar_), 129.5 (2 CH_Ar_), 129.9 (CH_Ar_), 130.7 (C_Ar_), 136.0 (C_Ar_), 144.3 (C_Ar_) ppm. HRMS (ESI) *m*/*z* [M + H]^+^ calcd. for [C_10_H_8_BrN_3_O_2_]^+^: 281.9873; found 281.9874.

2-Bromo-4-(4-methoxypheny)-1-methyl-5-nitro-1*H*-imidazole (**3b**): Yield 65% (71 mg); orange solid; M.p. 125 °C. ^1^H-NMR (CDCl_3_): δ = 3.86 (s, 3H, NCH_3_), 4.00 (s, 3H, OCH_3_), 6.95 (d, ^3^*J*_H-H_ = 8.9 Hz, 2H, 2 Ar-H), 7.77 (d, ^3^*J*_H-H_ = 8.8 Hz, 2H, 2 Ar-H) ppm. ^13^C-NMR (CDCl_3_): δ = 36.6 (NCH_3_), 55.3 (OCH_3_), 113.6 (2 CH_Ar_), 123.0 (C_Ar_), 126.8 (C_Ar_), 131.3 (2 CH_Ar_), 135.8 (C_Ar_), 144.5 (C_Ar_), 161.0 (C_Ar_) ppm. HRMS (ESI) *m*/*z* [M + H]^+^ calcd. for [C_11_H_10_BrN_3_O_3_]^+^: 311.9978; found 311.9977.

2-Bromo-1-methyl-5-nitro-4-[4-(trifluoromethyl)phenyl]-1*H*-imidazole (**3c**): Yield 62% (76 mg); yellow solid; M.p. 102 °C. ^1^H-NMR (CDCl_3_): δ = 4.03 (s, 3H, NCH_3_), 7.69 (d, ^3^*J*_H-H_ = 8.2 Hz, 2H, 2 Ar-H), 8.87 (d, ^3^*J*_H-H_ = 8.2 Hz, 2H, 2 Ar-H) ppm. ^13^C-NMR (CDCl_3_): δ = 36.6 (NCH_3_), 123.9 (q, ^1^*J*_C-F_ = 272.4 Hz, CF_3_), 125.1 (q, ^3^*J*_C-F_ = 3.9 Hz, 2 CH_Ar_), 126.9 (C), 129.9 (2 CH_Ar_), 131.6 (q, ^2^*J*_C-F_ = 32.5 Hz, C_Ar_), 134.2 (C_Ar_), 136.3 (C_Ar_), 142.4 (C_Ar_) ppm. HRMS (ESI) *m*/*z* [M + H]^+^ calcd. for [C_11_H_7_BrF_3_N_3_O_2_]^+^: 349.9746; found 349.9744.

2-Bromo-1-methyl-5-nitro-4-(3-nitrophenyl)-1*H*-imidazole (**3d**): Yield 61% (70 mg); yellow solid; M.p. 166 °C. ^1^H-NMR (CDCl_3_): δ = 4.06 (s, 3H, NCH_3_), 7.63 (t, ^3^*J*_H-H_ = 8.2 Hz, 1H, Ar-H), 8.10 (d, ^3^*J*_H-H_ = 7.7 Hz, 1H, Ar-H), 8.28-8.32 (m, 1H, Ar-H), 8.66 (s, 1H, Ar-H) ppm. ^13^C-NMR (CDCl_3_): δ = 36.8 (NCH_3_), 124.5 (CH_Ar_), 124.8 (CH_Ar_), 127.1 (C_Ar_), 129.2 (CH_Ar_), 132.4 (C_Ar_), 135.4 (CH_Ar_), 136.3 (C_Ar_), 141.5 (C_Ar_), 148.0 (C_Ar_) ppm. Anal. Calcd. for C_10_H_7_BrN_4_O_4_ (327.09): C 36.72, H 2.16, N 17.13; found C 36.82, H 2.06, N 17.13.

4-(2-Bromo-1-methyl-5-nitro-1*H*-imidazol-4-yl)benzonitrile (**3e**): Yield 64% (69 mg); yellow solid; M.p. 155 °C. ^1^H-NMR (CDCl_3_): δ = 4.04 (s, 3H, NCH_3_), 7.73 (d, ^3^*J*_H-H_ = 8.5 Hz, 2H, 2 Ar-H), 7.88 (d, ^3^*J*_H-H_ = 8.5 Hz, 2H, 2 Ar-H) ppm. ^13^C-NMR (CDCl_3_): δ = 35.8 (NCH_3_), 113.3 (C), 118.4 (C_Ar_), 127.1 (C_Ar_), 130.2 (2 CH_Ar_), 131.9 (2 CH_Ar_), 135.0 (C_Ar_), 136.4 (C_Ar_), 141.8 (C_Ar_) ppm. HRMS (ESI) *m*/*z* [M + H]^+^ calcd. for [C_11_H_7_BrN_4_O_2_]^+^: 306.9825; found 306.9824.

2-Bromo-4-(4-fluorophenyl)-1-methyl-5-nitro-1*H*-imidazole (**3f**): Yield 62% (65 mg); yellow solid; M.p. 112 °C. ^1^H-NMR (CDCl_3_): δ = 4.01 (s, 3H, NCH_3_), 7.08–7.15 (m, 2H, 2 Ar-H), 7.74–7.80 (m, 2H, 2 Ar-H) ppm. ^13^C-NMR (CDCl_3_): δ = 36.6 (NCH_3_), 115.2 (d, ^2^*J*_C-F_ = 21.5 Hz, 2 CH_Ar_), 126.7 (C_Ar_), 126.8 (C_Ar_), 131.7 (d, ^3^*J*_C-F_ = 8.8 Hz, 2 CH_Ar_), 135.9 (C_Ar_), 143.3 (C_Ar_), 163.6 (d, ^1^*J*_C-F_ = 250.9 Hz, C_Ar_) ppm. Anal. Calcd. for C_10_H_7_BrFN_3_O_2_ (300.08): C 40.02, H 2.35, N 14.00; found C 39.86, H 2.23, N 13.80.

2-Bromo-1-methyl-5-nitro-4-(3,4,5-trimethoxyphenyl)-1*H*-imidazole (**3g**): Yield 55% (72 mg); yellow solid; M.p. 134 °C. ^1^H-NMR (CDCl_3_): δ = 3.89 (s, 9H, NCH_3,_ 2 OCH_3_), 4.00 (s, 3H, OCH_3_), 7.07 (s, 2H, 2 Ar-H), ppm. ^13^C-NMR (CDCl_3_δ = 36.7 (NCH_3_), 56.2 (2 OCH_3_), 60.9 (OCH_3_), 107.0 (2 CH_Ar_), 107.4 (C_Ar_), 125.7 (C_Ar_), 126.6 (C_Ar_), 139.6 (C_Ar_), 144.0 (C_Ar_), 152.8 (2 C_Ar_) ppm. HRMS (ESI) *m*/*z* [M + H]^+^ calcd. for [C_13_H_14_BrN_3_O_5_]^+^: 372.0190; found 372.0187.

2-Bromo-1-methyl-5-nitro-4-*p*-tolyl-1*H*-imidazole (**3h**): Yield 63% (65 mg); yellow solid; M.p. 160 °C. ^1^H-NMR (CDCl_3_): δ = 2.40 (s, 3H, CH_3_), 4.01 (s, 3H, NCH_3_), 7.24 (d, ^3^*J*_H-H_ = 8.2 Hz, 2H, 2 CH ar), 7.66 (d, ^3^*J*_H-H_ = 8.2 Hz, 2H, 2 CH ar) ppm. ^13^C-NMR (CDCl_3_): δ = 21.4 (CH_3_), 36.5 (NCH_3_), 125.4 (C_Ar_), 126.6 (C_Ar_), 127.8 (C_Ar_), 128.9 (2 CH_Ar_), 129.4 (2 CH_Ar_), 140.2 (C_Ar_), 144.5 (C_Ar_) ppm. Anal. Calcd. for C_11_H_10_BrN_3_O_2_ (296.12): C 44.62, H 3.40, N 14.19; found C 44.63, H 3.27, N 13.95.

[4-(2-Bromo-1-methyl-5-nitro-1*H*-imidazol-4-yl)phenyl]methanol (**3i**): Yield 58% (65 mg); yellow solid; M.p. 132 °C. ^1^H-NMR (DMSO-*d*_6_): δ = 3.91 (s, 3H, NCH_3_), 4.55 (d, ^3^*J*_H-H_ = 5.8 Hz, 2H, CH_2_), 5.29 (t, ^3^*J*_H-H_ = 5.7 Hz, 1H, OH), 7.40 (d, ^3^*J*_H-H_ = 8.2 Hz, 2H, 2 Ar-H), 7.63 (d, ^3^*J*_H-H_ = 8.2 Hz, 2H, 2 Ar-H) ppm. ^13^C-NMR (DMSO-d_6_): δ = 36.7 (NCH_3_), 62.8 (CH_2_), 126.1 (2 CH_Ar_), 127.4 (C_Ar_), 129.1 (2 CH_Ar_), 129.6 (C_Ar_), 136.1 (C_Ar_), 143.0 (C_Ar_), 144.3 (C_Ar_) ppm. Anal. Calcd. for C_11_H_10_BrN_3_O_3_ (312.12): C 42.33, H 3.23, N 13.46; found C 42.34, H 3.11, N 13.33.

1-Methyl-5-nitro-2-4-diphenyl-1*H*-imidazole (**5**): Yield 9% using general procedure for the regioselective Suzuki-Miyaura coupling reaction (9 mg); yellow solid; M.p. 129 °C. ^1^H-NMR (CDCl_3_): δ = 4.00 (s, 3H, NCH_3_), 7.44–7.47 (m, 3H, 3 Ar-H), 7.54–7.56 (m, 3H, 3 Ar-H), 7.69–7.72 (m, 2H, 2 Ar-H), 7.83–7.87 (m, 2H, 2 Ar-H) ppm. ^13^C-NMR (CDCl_3_): δ = 36.2 (NCH_3_), 128.1 (2 CH_Ar_), 128.3 (C_Ar_), 128.9 (2 CH_Ar_), 129.5 (CH_Ar_), 129.7 (4 CH_Ar_), 130.7 (CH_Ar_), 131.7 (C_Ar_), 135.9 (C_Ar_), 144.2 (C_Ar_), 150.5 (C_Ar_) ppm. Anal. Calcd. for C_16_H_13_N_3_O_2_ (279.29): C 68.71, H 4.69, N 15.05; found C 68.43, H 4.62, N 14.85.

#### 3.2.3. General Procedure for the One-Pot Regioselective Bis-Suzuki-Miyaura Coupling Reaction

A solution of 2,4-dibromo-1-methyl-5-nitro-1*H*-imidazole (**2**, 0.1 g, 0.35 mmol), 4-methoxy-phenylboronic acid (72 mg, 0.46 mmol, 1.3 equiv.), Pd(OAc)_2_ (3 mg, 0.014 mmol, 0.04 equiv.), Na_2_CO_3_ (0.11 g, 1.05 mmol, 3 equiv.), in a DME/EtOH/H_2_O mixture (3/1/1, 5 mL) was heated at 60 °C under microwave irradiation for 2 h. After cooling, boronic acid (0.45 mmol, 1.3 equiv.), PPh_3_ (9 mg, 0.035 mmol, 0.1 equiv.), Na_2_CO_3_ (0.11 g, 1.05 mmol, 3 equiv.), were introduced under argon. The mixture was heated at 110 °C for 1 h under microwave irradiation. After cooling, 60 mL of water were added and the solution was extracted with dichloromethane (3 × 50 mL). The organic layer was washed with water (3 × 100 mL), dried over Na_2_SO_4_ and evaporated. The crude product was purified by column chromatography [silica gel, petroleum ether/ethyl acetate (9/1), (7/3 for **6e**), (5/5 for **6j**)] and recrystallized from propan-2-ol.

4-(4-Methoxyphenyl)-1-methyl-5-nitro-2-phenyl-1*H*-imidazole (**6a**): Yield 71% (77 mg); yellow solid; M.p. 120 °C. ^1^H-NMR (CDCl_3_): δ = 3.86 (s, 3H, NCH_3_), 3.99 (s, 3H, OCH_3_), 6.98 (d, ^3^*J*_H-H_ = 8.8 Hz, 2H, 2 Ar-H), 7.53–7.56 (m, 3H, 3 Ar-H), 7.68–7.72 (m, 2H, 2 Ar-H), 7.87 (d, ^3^*J*_H-H_ = 8.8 Hz, 2H, 2 Ar-H) ppm. ^13^C-NMR (CDCl_3_): δ = 36.3 (NCH_3_), 55.3 (OCH_3_), 113.5 (2 CH_Ar_), 123.9 (C_Ar_), 128.3 (C_Ar_), 128.9 (2 CH_Ar_), 129.7 (2 CH_Ar_), 130.7 (CH_Ar_), 131.4 (2 CH_Ar_), 135.6 (C_Ar_), 144.3 (C_Ar_), 150.6 (C_Ar_), 160.7 (C_Ar_) ppm. HRMS (ESI) *m*/*z* [M + H]^+^ calcd. for [C_17_H_15_N_3_O_3_]^+^: 310.1186; found 310.1185.

2-(4-Fluorophenyl)-4-(4-methoxyphenyl)-1-methyl-5-nitro-1*H*-imidazole (**6b**): Yield 70% (80 mg); yellow solid; M.p. 132 °C. ^1^H-NMR (CDCl_3_): δ = 3.87 (s, 3H, NCH_3_), 3.97 (s, 3H, OCH_3_), 6.98 (d, ^3^*J*_H-H_ = 9.0 Hz, 2H, 2 Ar-H), 7.21–7.28 (m, 2H, 2 Ar-H), 7.68–7.73 (m, 2H, 2 Ar-H), 7.85 (d, ^3^*J*_H-H_ = 9.0 Hz, 2H, 2 Ar-H) ppm. ^13^C-NMR (CDCl_3_): δ = 36.3 (NCH_3_), 55.4 (OCH_3_), 113.6 (2 CH_Ar_), 116.3 (d, ^2^*J*_C-F_ = 21.6 Hz, 2 CH_Ar_), 123.9 (C_Ar_), 123.6 (C_Ar_), 131.4 (2 CH_Ar_), 131.9 (d, ^3^*J*_C-F_ = 8.7 Hz, 2 CH_Ar_), 144.3 (C_Ar_), 148.9 (C_Ar_), 149.6 (C_Ar_), 160.0 (d, ^1^*J*_C-F_ = 272.1 Hz, C_Ar_), 160.8 (C_Ar_) ppm. HRMS (ESI) *m*/*z* [M + H]^+^ calcd. for [C_17_H_14_FN_3_O_3_]^+^: 328.1092; found 328.1093.

2-(4-Chlorophenyl)-4-(4-methoxyphenyl)-1-methyl-5-nitro-1*H*-imidazole (**6c**): Yield 60% (72 mg); yellow solid; M.p. 134 °C. ^1^H-NMR (CDCl_3_): δ = 3.87 (s, 3H, NCH_3_), 3.98 (s, 3H, OCH_3_), 6.98 (d, ^3^*J*_H-H_ = 9.0 Hz, 2H, 2 Ar-H), 7.53 (d, ^3^*J*_H-H_ = 8.7 Hz, 2H, 2 Ar-H), 7.66 (d, ^3^*J*_H-H_ = 8.7 Hz, 2H, 2 Ar-H), 7.84 (d, ^3^*J*_H-H_ = 8.9 Hz, 2H, 2 Ar-H) ppm. ^13^C-NMR (CDCl_3_): δ = 36.3 (NCH_3_), 55.3 (OCH_3_), 113.6 (2 CH_Ar_), 123.8 (C_Ar_), 126.8 (C_Ar_), 129.3 (2 CH_Ar_), 131.0 (2 CH_Ar_), 131.4 (2 CH_Ar_), 134.8 (C_Ar_), 137.1 (C_Ar_), 144.3 (C_Ar_), 149.4 (C_Ar_), 160.8 (C_Ar_) ppm. Anal. Calcd. for C_17_H_14_ClN_3_O_3_ (343.76): C 59.40, H 4.10, N 12.22; found C 59.16, H 3.82, N 12.31.

4-(4-Methoxyphenyl)-1-methyl-5-nitro-2-(3-nitrophenyl)-1*H*-imidazole (**6d**): Yield 65% (81 mg); yellow solid; M.p. 192 °C. ^1^H-NMR (CDCl_3_): δ = 3.87 (s, 3H, NCH_3_), 4.03 (s, 3H, OCH_3_), 6.99 (d, ^3^*J*_H-H_ = 8.9 Hz, 2H, 2 Ar-H), 7.76 (t, ^3^*J*_H-H_ = 8.0 Hz, 1H, Ar-H), 7.85 (d, ^3^*J*_H-H_ = 8.9 Hz, 2H, 2 Ar-H), 8.06 (d, ^3^*J*_H-H_ = 7.7 Hz, 1H, Ar-H), 8.42 (d, ^3^*J*_H-H_ = 8.2 Hz, 1H, Ar-H), 8.59 (t, ^4^*J*_H-H_ = 1.7 Hz, 1H, Ar-H) ppm. ^13^C-NMR (CDCl_3_): δ = 36.3 (NCH_3_), 55.4 (OCH_3_), 113.6 (2 CH_Ar_), 123.5 (C_Ar_), 124.6 (CH_Ar_), 125.2 (CH_Ar_), 130.2 (CH_Ar_), 131.3 (2 CH_Ar_), 135.3 (CH_Ar_), 135.9 (C_Ar_), 144.2 (C_Ar_), 147.7 (C_Ar_), 148.4 (C_Ar_), 156.1 (C_Ar_), 160.9 (C_Ar_) ppm. Anal. Calcd. for C_17_H_14_N_4_O_5_ (354.32): C 57.63, H 3.98, N 15.81; found C 57.38, H 3.79, N 15.59.

4-(4-Methoxyphenyl)-1-methyl-5-nitro-2-(3,4,5-trimethoxyphenyl)-1*H*-imidazole (**6e**): Yield 50% (70 mg); yellow solid; M.p. 158 °C. ^1^H-NMR (CDCl_3_): δ = 3.86 (s, 3H, NCH_3_), 3.91 (s, 9H, 3 OCH_3_), 3.98 (s, 3H, OCH_3_), 6.87 (s, 2H, 2 Ar-H), 6.97 (d, ^3^*J*_H-H_ = 8.8 Hz, 2H, 2 Ar-H), 7.86 (d, ^3^*J*_H-H_ = 8.8 Hz, 2H, 2 Ar-H) ppm. ^13^C-NMR (CDCl_3_): δ = 36.4 (NCH_3_), 55.3 (OCH_3_), 56.4 (2 OCH_3_), 61.0 (OCH_3_), 107.0 (2 CH_Ar_), 113.5 (2 CH_Ar_), 123.6 (C_Ar_), 123.9 (C_Ar_), 131.4 (2 CH_Ar_), 135.6 (C_Ar_), 140.2 (C_Ar_), 144.3 (C_Ar_), 150.7 (C_Ar_), 153.5 (2 C_Ar_), 160.7 (C_Ar_) ppm. Anal. Calcd. for C_20_H_21_N_3_O_6_ (399.40): C 60.14, H 5.30, N 10.52; found C 59.67, H 5.05, N 10.67.

4-(4-Methoxyphenyl)-1-methyl-5-nitro-2-[3-(trifluoromethyl)-phenyl]-1*H*-imidazole (**6f**): Yield 58% (76 mg); yellow solid; M.p. 117 °C. ^1^H-NMR (CDCl_3_): δ = 3.87 (s, 3H, NCH_3_), 4.00 (s, 3H, OCH_3_), 6.99 (d, ^3^*J*_H-H_ = 9.0 Hz, 2H, 2 Ar-H), 7.69 (t, ^3^*J*_H-H_ = 7.7 Hz, 1H, Ar-H), 7.81–7.90 (m, 4H, 4 Ar-H), 8.00 (s, 1H, Ar-H). ^13^C-NMR (CDCl_3_): δ = 36.2 (NCH_3_), 55.3 (OCH_3_), 113.6 (2 CH_Ar_), 123.5 (q, ^1^*J*_C-F_ = 272.9 Hz, CF_3_), 123.6 (C_Ar_), 126.7 (q, ^3^*J*_C-F_ = 3.7 Hz, CH_Ar_), 127.4 (q, ^3^*J*_C-F_ = 3.7 Hz, CH_Ar_), 129.3 (C_Ar_), 129.6 (CH_Ar_), 131.3 (2 CH_Ar_), 131.7 (q, ^2^*J*_C-F_ = 33.0 Hz, C_Ar_), 132.8 (CH_Ar_), 135.8 (C_Ar_), 144.2 (C_Ar_), 148.8 (C_Ar_), 160.9 (C_Ar_) ppm. HRMS (ESI) *m*/*z* [M + H]^+^ calcd. for [C_18_H_14_F_3_N_3_O_3_]^+^: 378.1060; found 378.1058.

4-(4-Methoxyphenyl)-1-methyl-5-nitro-2-*p*-tolyl-1*H*-imidazole (**6g**): Yield 56% (64 mg); yellow solid; M.p. 136 °C. ^1^H-NMR (CDCl_3_): δ = 2.45 (s, 3H, CH_3_), 3.86 (s, 3H, NCH_3_), 3.97 (s, 3H, OCH_3_), 6.98 (d, ^3^*J*_H-H_ = 8.9 Hz, 2H, 2 Ar-H), 7.35 (d, ^3^*J*_H-H_ = 7.9 Hz, 2H, 2 Ar-H), 7.58 (d, ^3^*J*_H-H_ = 8.2 Hz, 2H, 2 Ar-H), 7.86 (d, ^3^*J*_H-H_ = 8.9 Hz, 2H, 2 Ar-H) ppm. ^13^C-NMR (CDCl_3_): δ = 21.5 (CH_3_), 36.3 (NCH_3_), 55.3 (OCH_3_), 113.5 (2 CH_Ar_), 124.1 (C_Ar_), 125.5 (C_Ar_), 129.5 (2 CH_Ar_), 129.6 (2 CH_Ar_), 131.4 (2 CH_Ar_), 135.6 (C_Ar_), 141.1 (C_Ar_), 144.5 (C_Ar_), 150.8 (C_Ar_), 160.7 (C_Ar_) ppm. Anal. Calcd. for C_18_H_17_N_3_O_3_ (323.35): C 66.86, H 5.30, N 13.00; found C 66.74, H 5.22, N 12.81.

4-(4-Methoxyphenyl)-1-methyl-2-(naphthalen-2-yl)-5-nitro-1*H*-imidazole (**6h**): Yield 61% (77 mg); yellow solid; M.p. 143 °C. ^1^H-NMR (CDCl_3_): δ = 3.87 (s, 3H, NCH_3_), 4.05 (s, 3H, OCH_3_), 6.99 (d, ^3^*J*_H-H_ = 8.9 Hz, 2H, 2 Ar-H), 7.56–7.64 (m, 2H, 2 Ar-H), 7.77 (dd, ^4^*J*_H-H_ = 1.6 Hz, ^3^*J*_H-H_ = 8.5 Hz, 1H, Ar-H), 7.90 (d, ^3^*J*_H-H_ = 8.9 Hz, 2H, 2 Ar-H), 7.92–7.96 (m, 2H, 2 Ar-H), 8.01 (d, ^3^*J*_H-H_ = 8.5 Hz, 1H, Ar-H), 8.22 (s, 1H, Ar-H) ppm. ^13^C-NMR (CDCl_3_): δ = 36.4 (NCH_3_), 55.3 (OCH_3_), 113.6 (2 CH_Ar_), 124.0 (C_Ar_), 125.7 (C_Ar_), 125.9 (CH_Ar_), 127.1 (CH_Ar_), 127.8 (CH_Ar_), 127.9 (CH_Ar_), 128.6 (CH_Ar_), 128.8 (CH_Ar_), 130.2 (CH_Ar_), 131.4 (2 CH_Ar_), 132.8 (C_Ar_), 134.0 (C_Ar_), 135.8 (C_Ar_), 144.4 (C_Ar_), 150.7 (C_Ar_), 160.8 (C_Ar_) ppm. Anal. Calcd. for C_21_H_17_N_3_O_3_ (359.38): C 70.18, H 4.77, N 11.69; found C 70.23, H 4.69, N 11.69.

4-(4-Methoxyphenyl)-1-methyl-2-(5-methylthiophen-2-yl)-5-nitro-1*H*-imidazole (**6i**): Yield 52% (60 mg); yellow solid; M.p. 151 °C. ^1^H-NMR (CDCl_3_): δ = 2.57 (s, 3H, CH_3_), 3.86 (s, 3H, NCH_3_), 4.09 (s, 3H, OCH_3_), 6.86-6.88 (m, 1H, Ar-H), 6.97 (d, ^3^*J*_H-H_ = 9.0 Hz, 2H, 2 Ar-H), 7.37 (d, ^4^*J*_H-H_ = 3.6 Hz, 1H, Ar-H), 7.85 (d, ^3^*J*_H-H_ = 9.0 Hz, 2H, 2 Ar-H) ppm. ^13^C-NMR (CDCl_3_): δ = 15.4 (CH_3_), 36.0 (NCH_3_), 55.3 (OCH_3_), 113.5 (2 CH_Ar_), 124.0 (C_Ar_), 126.4 (CH_Ar_), 127.7 (C_Ar_), 129.9 (CH_Ar_), 131.5 (2 CH_Ar_), 135.5 (C_Ar_), 136.8 (C_Ar_), 145.0 (C_Ar_), 145.3 (C_Ar_), 160.8 (C_Ar_) ppm. HRMS (ESI) *m*/*z* [M + H]^+^ calcd. for [C_16_H_15_N_3_O_3_S]^+^: 330.0907; found 330.0907.

3-[4-(4-Methoxyphenyl)-1-methyl-5-nitro-1*H*-imidazol-2-yl]pyridine (**6j**): Yield 69% (75 mg); yellow solid; M.p. 152 °C. ^1^H-NMR (CDCl_3_): δ = 3.86 (s, 3H, NCH_3_), 4.01 (s, 3H, OCH_3_), 6.98 (d, ^3^*J*_H-H_ = 9.0 Hz, 2H, 2 Ar-H), 7.48–7.53 (m, 1H, Ar-H), 8.84 (d, ^3^*J*_H-H_ = 8.9 Hz, 2H, 2 Ar-H), 8.04–8.08 (m, 1H, Ar-H), 8.79–8.80 (m, 1H, Ar-H), 8.96 (s, 1H, Ar-H) ppm. ^13^C-NMR (CDCl_3_): δ = 36.2 (NCH_3_), 55.3 (OCH_3_), 113.6 (2 CH_Ar_), 123.7 (CH_Ar_), 125.0 (C_Ar_), 131.3 (2 CH_Ar_), 135.9 (C_Ar_), 137.2 (CH_Ar_), 144.4 (C_Ar_), 147.5 (C_Ar_), 149.9 (CH_Ar_), 151.4 (CH_Ar_), 160.8 (2 C_Ar_). HRMS (ESI) *m*/*z* [M + H]^+^ calcd. for [C_16_H_14_N_4_O_3_]^+^: 311.1139; found 311.1136.

2-(4-Methoxyphenyl)-1-methyl-5-nitro-4-phenyl-1*H*-imidazole (**6**): Yield 64% (69 mg); yellow solid; M.p. 128 °C. ^1^H-NMR (CDCl_3_): δ = 3.88 (s, 3H, NCH_3_), 3.99 (s, 3H, OCH_3_), 7.04 (d, ^3^*J*_H-H_ = 8.9 Hz, 2H, 2 Ar-H), 7.43–7.48 (m, 3H, 3 Ar-H), 7.65 (d, ^3^*J*_H-H_ = 8.7 Hz, 2H, 2 Ar-H), 7.82–7.86 (m, 2H, 2 Ar-H) ppm. ^13^C-NMR (CDCl_3_): δ = 36.1 (NCH_3_), 55.5 (OCH_3_), 114.6 (2 CH_Ar_), 121.0 (C_Ar_), 128.0 (2 CH_Ar_), 129.4 (CH_Ar_), 129.7 (2 CH_Ar_), 131.3 (2 CH_Ar_), 132.1 (C_Ar_), 136.1 (C_Ar_), 144.2 (C_Ar_), 150.6 (C_Ar_), 161.7 (C_Ar_) ppm. Anal. Calcd. for C_17_H_15_N_3_O_3_ (309.32): C 66.01, H 4.89, N 13.58; found C 65.98, H 4.75, N 13.42.

2,4-bis(4-methoxypheny)-1-methyl-5-nitro-1*H*-imidazole (**7**): Yield 9% using general procedure for the one-pot regioselective bis-Suzuki-Miyaura coupling reaction (11 mg); yellow solid; M.p. 121 °C. ^1^H-NMR (CDCl_3_24 °C): δ = 3.86 (s, 3H, NCH_3_), 3.89 (s, 3H, OCH_3_), 3.97 (s, 3H, OCH_3_), 6.98 (d, ^3^*J*_H-H_ = 7.6 Hz, 2H, 2 Ar-H), 7.02 (d, ^3^*J*_H-H_ = 8.1 Hz, 2H, 2 Ar-H), 7.65 (d, ^3^*J*_H-H_ = 7.9 Hz, 2H, 2 Ar-H), 7.86 (d, ^3^*J*_H-H_ = 7.6 Hz, 2H, 2 Ar-H) ppm. ^13^C-NMR (CDCl_3_): δ = 36.5 (NCH_3_), 55.3 (OCH_3_), 55.5 (OCH_3_), 113.6 (2 CH_Ar_), 114.5 (2 CH_Ar_), 120.5 (C_Ar_), 124.0 (C_Ar_), 131.3 (2 CH_Ar_), 131.5 (2 CH_Ar_), 135.6 (C_Ar_), 144.5 (C_Ar_), 150.8 (C_Ar_), 160.8 (C_Ar_), 161.6 (C_Ar_) ppm. HRMS (ESI) *m*/*z* [M + H]^+^ calcd. for [C_18_H_17_N_3_O_4_] 340.1292; found 340.1292.

#### 3.2.4. General Procedure for the One-Pot Regioselective Suzuki-Miyaura/Sonogashira Coupling Reaction

A solution of 2,4-dibromo-1-methyl-5-nitro-1*H*-imidazole (**2**, 0.1 g, 0.35 mmol), 4-methoxy-phenylboronic acid (72 mg, 0.46 mmol, 1.3 equiv.), Pd(OAc)_2_ (3 mg, 0.014 mmol, 0.04 equiv.), Na_2_CO_3_ (0.11 g, 1.05 mmol, 3 equiv.), in a DME/EtOH/H_2_O mixture (3/1/1, 5 mL) was heated at 60 °C under microwave irradiation for 2 h. After cooling, terminal alkynes (0.45 mmol, 1.3 equiv.), PPh_3_ (9 mg, 0.035 mmol, 0.1 equiv.), CuI (7 mg, 0.035 mmol, 0.1 equiv.), Et_3_N (0.1 mL, 0.7 mmol, 2 equiv.), were introduced under argon. The mixture was heated at 70 °C for 1 h under microwave irradiation. After cooling, 60 mL of water were added and the solution was extracted with dichloromethane (3 × 50 mL). The organic layer was washed with water (3 × 100 mL), dried over Na_2_SO_4_ and evaporated. The crude product was purified by column chromatography [silica gel, petroleum ether/ethyl acetate (10%), (50% for **8g**)] and recrystallized from propan-2-ol.

4-(4-Methoxyphenyl)-1-methyl-5-nitro-2-(phenylethynyl)-1*H*-imidazole (**8a**): Yield 67% (78 mg); yellow solid; M.p. 156 °C. ^1^H-NMR (CDCl_3_): δ = 3.87 (s, 3H, NCH_3_), 4.14 (s, 3H, OCH_3_), 6.97 (d, ^3^*J*_H-H_ = 8.0 Hz, 2H, 2 Ar-H), 7.39–7.51 (m, 3H, 3 Ar-H), 7.64 (d, ^3^*J*_H-H_ = 6.6 Hz, 2H, 2 Ar-H), 8.82 (d, ^3^*J*_H-H_ = 8.8 Hz, 2H, 2 Ar-H) ppm. ^13^C-NMR (CDCl_3_): δ = 35.8 (NCH_3_), 55.3 (OCH_3_), 77.2 (C), 96.5 (C), 113.5 (2 CH_Ar_), 120.3 (C_Ar_), 123.6 (C_Ar_), 128.7 (2 CH_Ar_), 130.3 (CH_Ar_), 131.2 (2 CH_Ar_), 132.2 (2 CH_Ar_), 134.1 (C_Ar_), 134.4 (C_Ar_), 144.7 (C_Ar_), 160.8 (C_Ar_) ppm. Anal. Calcd. for C_19_H_15_N_3_O_3_ (333.34): C 68.46, H 4.54, N 12.61; found C 68.39, H 4.44, N 12.53.

4-(4-Methoxyphenyl)-1-methyl-5-nitro-2-(*m*-tolylethynyl)-1*H*-imidazole (**8b**): Yield 74% (90 mg); yellow solid; M.p. 138 °C. ^1^H-NMR (CDCl_3_): δ = 2.56 (s, 3H, CH_3_), 3.86 (s, 3H, NCH_3_), 4.14 (s, 3H, OCH_3_), 6.97 (d, ^3^*J*_H-H_ = 9.0 Hz, 2H, 2 Ar-H), 7.20–7.39 (m, 3H, 3 Ar-H), 7.60 (d, ^3^*J*_H-H_ = 8.4 Hz, 1H, Ar-H), 8.81 (d, ^3^*J*_H-H_ = 9.0 Hz, 2H, 2 Ar-H) ppm. ^13^C-NMR (CDCl_3_): δ = 20.9 (CH_3_), 35.7 (NCH_3_), 55.3 (OCH_3_), 81.0 (C), 95.6 (C), 113.5 (2 CH_Ar_), 120.2 (C_Ar_), 123.6 (C_Ar_), 126.0 (CH_Ar_), 129.8 (CH_Ar_), 130.3 (CH_Ar_), 131.2 (2 CH_Ar_), 132.8 (CH_Ar_), 134.1 (C_Ar_), 134.6 (C_Ar_), 141.1 (C_Ar_), 144.7 (C_Ar_), 160.8 (C_Ar_) ppm. Anal. Calcd. for C_20_H_17_N_3_O_3_ (347.37): C 69.15, H 4.93, N 12.10; found C 68.94, H 4.79, N 11.89.

2-[(3-Fluorophenyl)ethynyl]-4-(4-methoxyphenyl)-1-methyl-5-nitro-1*H*-imidazole (**8c**): Yield 59% (73 mg); yellow solid; M.p. 148 °C. ^1^H-NMR (CDCl_3_): δ = 3.86 (s, 3H, NCH_3_), 4.13 (s, 3H, OCH_3_), 6.97 (d, ^3^*J*_H-H_ = 8.8 Hz, 2H, 2 Ar-H), 7.13–7.21 (m, 1H, Ar-H), 7.30–7.42 (m, 3H, 3 Ar-H), 8.80 (d, ^3^*J*_H-H_ = 8.8 Hz, 2H, 2 Ar-H) ppm. ^13^C-NMR (CDCl_3_): δ = 35.9 (NCH_3_), 55.3 (OCH_3_), 77.9 (C), 94.8 (d, ^4^*J*_C-F_ = 3.2 Hz, C), 113.5 (2 CH_Ar_), 117.7 (d, ^2^*J*_C-F_ = 21.1 Hz, CH_Ar_), 119.0 (d, ^2^*J*_C-F_ = 23.9 Hz, CH_Ar_), 122.1 (d, ^3^*J*_C-F_ = 9.7 Hz, C_Ar_), 123.4 (C_Ar_), 128.1 (d, ^4^*J*_C-F_ = 3.2 Hz, CH_Ar_), 130.4 (d, ^3^*J*_C-F_ = 8.7 Hz, CH_Ar_), 131.2 (2 CH_Ar_), 133.9 (C_Ar_), 134.2 (C_Ar_), 144.6 (C_Ar_), 160.8 (C_Ar_), 162.2 (d, ^1^*J*_C-F_ = 248.2 Hz, C_Ar_) ppm. Anal. Calcd. for C_19_H_14_FN_3_O_3_ (351.33): C 64.95, H 4.02, N 11.96; found C 64.67, H 3.84, N 11.79.

2-[(4-Methoxy-2-methylphenyl)ethynyl]-4-(4-methoxyphenyl)-1-methyl-5-nitro-1*H*-imidazole (**8d**): Yield 53% (70 mg); yellow solid; M.p. 182 °C. ^1^H-NMR (CDCl_3_): δ = 2.53 (s, 3H, CH_3_), 3.84 (s, 3H, NCH_3_), 3.86 (s, 3H, OCH_3_), 4.13 (s, 3H, OCH_3_), 6.74–6.81 (m, 2H, 2 Ar-H), 6.97 (d, ^3^*J*_H-H_ = 8.9 Hz, 2H, 2 Ar-H), 7.53 (d, ^3^*J*_H-H_ = 8.4 Hz, 1H, Ar-H), 7.81 (d, ^3^*J*_H-H_ = 8.9 Hz, 2H, 2 Ar-H) ppm. ^13^C-NMR (CDCl_3_): δ = 21.1 (CH_3_), 35.7 (NCH_3_), 55.3 (OCH_3_), 55.4 (OCH_3_), 80.1 (C), 96.2 (C), 111.8 (CH_Ar_), 112.3 (C_Ar_), 113.5 (2 CH_Ar_), 115.5 (CH_Ar_), 123.7 (C_Ar_), 131.3 (2 CH_Ar_), 134.0 (C_Ar_), 134.4 (CH_Ar_), 135.1 (C_Ar_), 143.2 (C_Ar_), 144.8 (C_Ar_), 160.7 (C_Ar_), 161.1 (C_Ar_) ppm. Anal. Calcd. for C_21_H_19_N_3_O_4_ (377.39): C 66.83, H 5.07, N 11.13; found C 66.56, H 4.91, N 11.10.

2-(Cyclopentylethynyl)-4-(4-methoxyphenyl)-1-methyl-5-nitro-1*H*-imidazole (**8e**): Yield 60% (69 mg); yellow solid; M.p. 98 °C. ^1^H-NMR (CDCl_3_): δ = 1.59–1.68 (m, 2H, CH_2_), 1.73–1.82 (m, 4H, 2 CH_2_), 2.00–2.10 (m, 2H, CH_2_), 2.92 (q, ^3^*J*_H-H_ = 7.5 Hz, 1H, CH), 3.84 (s, 3H, NCH_3_), 4.01 (s, 3H, OCH_3_), 6.94 (d, ^3^*J*_H-H_ = 9.0 Hz, 2H, 2 Ar-H), 7.77 (d, ^3^*J*_H-H_ = 9.0 Hz, 2H, 2 Ar-H) ppm. ^13^C-NMR (CDCl_3_): δ = 25.2 (2 CH_2_), 30.5 (CH), 33.4 (2 CH_2_), 35.5 (NCH_3_), 55.3 (OCH_3_), 68.9 (C), 103.5 (C), 113.4 (2 CH_Ar_), 123.7 (C_Ar_), 131.2 (2 CH_Ar_), 133.6 (C_Ar_), 134.9 (C_Ar_), 144.4 (C_Ar_), 160.7 (C_Ar_) ppm. Anal. Calcd. for C_18_H_19_N_3_O_3_ (325.36): C 66.45, H 5.89, N 12.91; found C 66.21, H 5.76, N 12.88.

2-[(4-*tert*-Butylphenyl)ethynyl]-4-(4-methoxyphenyl)-1-methyl-5-nitro-1*H*-imidazole (**8f**): Yield 63% (86 mg); yellow oil. ^1^H-NMR (CDCl_3_): δ = 1.34 (s, 9H, 3 CH_3_), 3.86 (s, 3H, NCH_3_), 4.12 (s, 3H, OCH_3_), 6.96 (d, ^3^*J*_H-H_ = 8.8 Hz, 2H, 2 Ar-H), 7.43 (d, ^3^*J*_H-H_ = 8.4 Hz, 2H, 2 Ar-H), 7.56 (d, ^3^*J*_H-H_ = 8.4 Hz, 2H, 2 Ar-H), 7.81 (d, ^3^*J*_H-H_ = 8.8 Hz, 2H, 2 Ar-H) ppm. ^13^C-NMR (CDCl_3_): δ = 31.0 (3 CH_3_), 35.0 (C), 35.7 (NCH_3_), 55.3 (OCH_3_), 76.8 (C), 96.9 (C), 113.5 (2 CH_Ar_), 117.2 (C_Ar_), 123.6 (C_Ar_), 125.7 (2 CH_Ar_), 131.2 (2 CH_Ar_), 132.0 (2 CH_Ar_), 134.0 (C_Ar_), 134.6 (C_Ar_), 144.7 (C_Ar_), 153.9 (C_Ar_), 160.7 (C_Ar_) ppm. HRMS (ESI) *m*/*z* [M + H]^+^ calcd. for [C_23_H_23_N_3_O_3_]^+^: 390.1812; found 390.1813.

3-[4-(4-Methoxyphenyl)-1-methyl-5-nitro-1*H*-imidazol-2-yl]prop-2-yn-1-ol (**8g**): Yield 51% (51 mg); yellow solid; M.p. 177 °C. ^1^H-NMR (DMSO-d_6_): δ = 3.81 (s, 3H, NCH_3_), 3.97 (s, 3H, OCH_3_), 4.43 (d, ^3^*J*_H-H_ = 5.7 Hz, 2H, CH_2_), 5.67 (t, ^3^*J*_H-H_ = 5.8 Hz, 1H, OH), 7.01 (d, ^3^*J*_H-H_ = 8.8 Hz, 2H, 2 Ar-H), 7.67 (d, ^3^*J*_H-H_ = 8.8 Hz, 2H, 2 Ar-H). ^13^C-NMR (DMSO-d_6_): δ = 35.9 (NCH_3_), 49.8 (CH_2_), 55.7 (OCH_3_), 72.7 (C), 97.5 (C), 113.9 (2 CH_Ar_), 124.1 (C_Ar_), 131.2 (2 CH_Ar_), 133.6 (C_Ar_), 134.5 (C_Ar_), 143.2 (C_Ar_), 160.6 (C_Ar_) ppm. Anal. Calcd. for C_14_H_13_N_3_O_4_ (287.27): C 58.53, H 4.56, N 14.63; found C 58.64, H 4.51, N 14.40.

2-[(3-Chlorophenyl)ethynyl]-4-(4-methoxyphenyl)-1-methyl-5-nitro-1*H*-imidazole (**8h**): Yield 61% (79 mg); yellow solid; M.p. 139 °C. ^1^H-NMR (CDCl_3_): δ = 3.86 (s, 3H, NCH_3_), 4.13 (s, 3H, OCH_3_), 6.97 (d, ^3^*J*_H-H_ = 8.9 Hz, 2H, 2 Ar-H), 7.32–7.38 (m, 1H, Ar-H), 7.42–7.47 (m, 1H, Ar-H), 7.51 (dt, ^4^*J*_H-H_ = 1.4 Hz, ^3^*J*_H-H_ = 7.4 Hz, 1H, Ar-H), 7.61 (t, ^4^*J*_H-H_ = 1.4 Hz, 1H, Ar-H), 8.80 (d, ^3^*J*_H-H_ = 8.9 Hz, 2H, 2 Ar-H) ppm. ^13^C-NMR (CDCl_3_): δ = 35.8 (NCH_3_), 55.3 (OCH_3_), 78.2 (C), 94.7 (C), 113.6 (2 CH_Ar_), 122.0 (C_Ar_), 123.4 (C_Ar_), 130.0 (CH_Ar_), 130.3 (CH_Ar_), 130.6 (CH_Ar_), 131.2 (2 CH_Ar_), 131.9 (CH_Ar_), 133.8 (C_Ar_), 134.2 (C_Ar_), 134.6 (C_Ar_), 144.6 (C_Ar_), 160.8 (C_Ar_). Anal. Calcd. for C_19_H_14_ClN_3_O_3_ (367.79): C 62.05, H 3.84, N 11.43; found C 61.90, H 3.77, N 11.35.

2-[(2-Chlorophenyl)ethynyl]-4-(4-methoxyphenyl)-1-methyl-5-nitro-1*H*-imidazole (**8i**): Yield 52% (67 mg); yellow solid; M.p. 171 °C. ^1^H-NMR (CDCl_3_): δ = 3.87 (s, 3H, NCH_3_), 4.19 (s, 3H, OCH_3_), 6.98 (d, ^3^*J*_H-H_ = 8.5 Hz, 2H, 2 Ar-H), 7.29–7.42 (m, 2H, 2 Ar-H), 7.49 (d, ^3^*J*_H-H_ = 7.9 Hz, 1H, Ar-H), 7.67 (d, ^3^*J*_H-H_ = 7.4 Hz, 1H, Ar-H), 7.80 (d, ^3^*J*_H-H_ = 8.7 Hz, 2H, 2 Ar-H) ppm. ^13^C-NMR (CDCl_3_): δ = 35.8 (NCH_3_), 55.3 (OCH_3_), 82.1 (C), 92.8 (C), 113.6 (2 CH_Ar_), 120.7 (C_Ar_), 123.6 (C_Ar_), 126.9 (CH_Ar_), 129.6 (CH_Ar_), 131.2 (CH_Ar_), 131.3 (2 CH_Ar_), 133.9 (CH_Ar_), 134.0 (C_Ar_), 134.1 (C_Ar_), 136.6 (C_Ar_), 144.6 (C_Ar_), 160.9 (C_Ar_) ppm. Anal. Calcd. for C_19_H_14_ClN_3_O_3_ (367.79): C 62.05, H 3.84, N 11.43; found C 61.86, H 3.66, N 11.31.

2-(Cyclopropylethynyl)-4-(4-methoxyphenyl)-1-methyl-5-nitro-1*H*-imidazole (**8j**): Yield 63% (66 mg); yellow solid; M.p. 124 °C. ^1^H-NMR (CDCl_3_): δ = 0.95–1.03 (m, 4H, 2 CH_2_), 1.50–1.60 (m, 1H, CH), 3.85 (s, 3H, NCH_3_), 4.02 (s, 3H, OCH_3_), 6.94 (d, ^3^*J*_H-H_ = 8.8 Hz, 2H, 2 Ar-H), 7.77 (d, ^3^*J*_H-H_ = 8.9 Hz, 2H, 2 Ar-H) ppm. ^13^C-NMR (CDCl_3_): δ = 0.2 (CH), 9.3 (2 CH_2_), 35.5 (NCH_3_), 55.3 (OCH_3_), 64.4 (C), 77.2 (C), 102.4 (C_Ar_), 113.5 (2 CH_Ar_), 123.7 (C_Ar_), 131.3 (2 CH_Ar_), 134.7 (C_Ar_), 144.3 (C_Ar_), 160.8 (C_Ar_) ppm. Anal. Calcd. for C_16_H_15_N_3_O_3_ (297.31): C 64.64, H 5.09, N 14.13; found C 64.46, H 4.92, N 14.22.

1-Methyl-5-nitro-2,4-bis(phenylethynyl)-1*H*-imidazole (**9**): Yield 10% using general procedure for the one-pot regioselective Suzuki-Miyaura/Sonogashira coupling reaction (12 mg); yellow solid; M.p. 166 °C. ^1^H-NMR (CDCl_3_): δ = 4.14 (s, 3H, NCH_3_), 7.38–7.48 (m, 6H, 6 Ar-H), 7.61–7.66 (m, 4H, 4 Ar-H) ppm. ^13^C-NMR (CDCl_3_): δ = 35.6 (NCH_3_), 76.7 (C), 81.0 (C), 96.8 (C), 97.1 (C), 120.0 (C_Ar_), 121.7 (C_Ar_), 127.7 (C_Ar_), 128.5 (2 CH_Ar_), 128.7 (2 CH_Ar_), 129.6 (CH_Ar_), 130.5 (CH_Ar_), 132.2 (2 CH_Ar_), 132.3 (2 CH_Ar_), 133.5 (C_Ar_), 135.1 (C_Ar_) ppm. HRMS (ESI) *m*/*z* [M + H]^+^ calcd. for [C_20_H_13_N_3_O_2_]^+^: 328.1081; found 328.1081.

#### 3.2.5. Crystal Data for Compound **3c**

2(C_11_H_7_BrF_3_N_3_O_2_), *M* = 700.21, *a* = 8.4376(4) Å, *b* = 12.5416(6) Å, *c* = 12.6319(5) Å, *α* = 82.406(4)°, *β* = 73.324(4)°, *γ* = 80.384(4)°, *V* = 1257.48(10) Å^3^, *T* = 293 K, space group *P*$\bar{1}$ *Z* = 2, 14503 reflections measured, 5058 independent reflections (*R_int_* = 0.0184). The final *R*_1_ values were 0.0320 (*I* > 2*σ*(*I*)). The final *wR*(*F*^2^) values were 0.0689 (*I* > 2*σ*(*I*)). The final *R_1_* values were 0.0469 (all data). The final *wR*(*F*^2^) values were 0.0752 (all data). The goodness of fit on *F*^2^ was 1.007. CCDC 1542022 contains the supplementary crystallographic data for this paper. These data can be obtained free of charge at www.cdcc.cam.ac.uk/data_request/cif of from the Cambridge Crystallographic Data Centre, 12, Union Road, Cambridge CB2 1EZ, UK; Fax: + 44 (1223) 336033; email: deposit@ccdc.cam.ac.uk.

Figures S1–S66: ^1^H- and ^13^C-NMR of all compounds **3a**–**i**, **5**, **6a**–**j**, **6**, **7**, **8a**–**j** and **9**.

### 3.3. Biology

#### 3.3.1. In Vitro Antibacterial Activity

Bacterial strains: the bacteria tested were obtained from the bacteriology laboratory of IHU Mediterranée Infection (Marseille, France). Each bacterium was cultured in anaerobic condition on 5% sheep blood-enriched Columbia agar (Biomérieux, Marcy l’Etoile, France), incubated at 37 °C for 48 h. The derivatives were dissolved in DMSO stock solution: 500 µg/mL. Thereafter, in a sterile medium and in a logarithmic growth phase, a concentration of 1 McFarland (3.10^8^ CFU/mL) was obtained using a spectrophotometer. First, in the 5% sheep blood-enriched Columbia agar medium, the superficial bacterial culture was performed with a swab impregnated with the bacterial suspension. Then, 10 µL of each derivative were deposited on blank sterile discs. For the negative control, the DMSO-impregnated disc was used. After 48 h of incubation, the growth inhibition zone diameter was measured.

#### 3.3.2. In Vitro Antiparasitic Activity

For *Trichomonas vaginalis* the MIC was carried out in a sterile 96-well plate by broth microdilution according to EUCAST guidelines, and using LYI medium. First, 190 µL of the LYI broth medium, was added to each well. Then, 10 µL of each imidazole derivative with the primary concentration 200 µg/mL was added to the first wells. After mixing, 100 µL of this mixture was embedded into the second well. A similar dilution procedure was carried out in the first wells line for metronidazole as control group. 100 µL of the trichomonas suspension (10^5^ cells/mL) was added to each well. For negative control, 200 µL of LYI broth medium were added to the border wells. The plates were incubated under anaerobic conditions 24 h at 37 °C.

#### 3.3.3. In Vitro Cytotoxicity Evaluation

##### Principle of the Assay

The NRU toxicity test is based on concentration-dependent reduction of the uptake of the vital dye Neutral Red measured 24 h after chemical treatment. Neutral red is a weak cationic dye that readily penetrates cell membranes by non-diffusion, accumulating intracellularly in lysosomes. Alterations of the cell surface or the sensitive lysosomal membrane lead to lysosomal fragility and other changes that gradually become irreversible. Such changes brought about by the action of xenobiotics result in a decreased uptake and binding of NR in non-viable cells.

##### Cell Line

CHO-K1 Chinese Hamster Ovary cells (ATCC CCL61), low passage number (<50).

##### Culture Medium

Mc Coy’s medium supplemented with penicillin 100 UI/mL and steptomycin 100 μg/mL, and 10% of inactivated calf serum, pΗ 7.2, freshly prepared, stored no longer than 1 week.

##### Test Procedure

Cells were seeded into two 96-well tissue culture plates (0.1 mL per well), at a concentration of 1.10^5^ cells/mL, and incubated at 37 °C (5% CΟ_2_) for 24 h until confluent. The culture medium was decanted and replaced by 100 µL of fresh medium containing the appropriate concentrations of the test substances (8 different concentrations in triplicate), then cells were incubated at 37 °C (5% CO_2_) in the dark for 24 h. At the end of the incubation period, cells were washed, placed into Neutral Red medium (50 μg/mL Neutral Red in complete medium) and incubated for 3 h at 37 °C, 5% CO_2_. Then, the medium was removed and cells were washed three times with 0.2 mL of PBS to remove excess dye. The Neutral Red medium was removed and the distaining solution (50% ethanol, 1% acetic acid, 49% distilled water; 50 µL per well) was added into the wells. Then, the plates were shaken for 15–20 min at room temperature in the dark. The degree of membrane damage (i.e., the increase of released Neutral Red) was measured by a fluorescence-luminescence reader. The Optical Density (OD) of each well was read at 540 nm. The results obtained for wells treated with the test material were compared to those of untreated control wells (100% viability) and converted to percentage values.

##### Calculation of CC_50_

The mean OD value of blank wells (containing only Neutral Red desorbed solution) was subtracted from the mean OD value of three treated wells (dilutions of the test material, positive control or HBSS). The percentages of cell viability were calculated as:

$$\begin{array}{c} \left(\mathrm{Mean}\;\mathrm{OD}\;\mathrm{of}\;\mathrm{test}\;\mathrm{wells}-\mathrm{mean}\;\mathrm{OD}\;\mathrm{of}\;\mathrm{blanks}\right)\times 100\\ \mathrm{Mean}\;\mathrm{OD}\;\mathrm{of}\;\mathrm{negative}\;\mathrm{control}-\mathrm{mean}\;\mathrm{OD}\;\mathrm{of}\;\mathrm{blanks} \end{array}$$

The concentration of the test substances causing a 50% release of Neutral Red as compared to the control culture was calculated by non-linear regression analysis using the Phototox Version 2.0 software (Zebet, Berlin, Germany).

## 4. Conclusions

In summary, we describe here a novel and efficient method to access 2,4-disubstituted 5-nitro-imidazole derivatives under microwave irradiation. We have developed original one-pot regioselective bis-Suzuki-Miyaura or Suzuki-Miyaura/Sonogashira reactions of 2,4-dibromo-1-methyl-5-nitro-1*H*-imidazole (**2**) allowing the functionalization at both C-2 and C-4 positions of this scaffold. Moreover, this method tolerates a wide range of boronic acids and terminal alkynes and provides versatile and rapid access to 2,4-disubstituted 5-nitroimidazole derivatives in moderate to good yields. The synthesized products were tested for their antimicrobial activities and compared with the standard metronidazole. Compounds obtained with the regioselective Suzuki-Miyaura reaction, bearing a bromine in 2-position and aryl or hetero aryl group in 4-position (compounds **3b**, **3e**–**i**), showed an equivalent activity to metronidazole against anaerobic bacteria, and higher activity against *T. vaginalis*.

## Figures and Tables

**Table 1:**
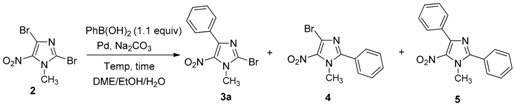
Optimization of the regioselective Suzuki-Miyaura reaction.

Entry	Pd (0.04 equiv.)	Temp (°C)	Time (h)	2 (%)	3a (%)	4 (%)	5 (%)
1 ^a^	Pd(PPh_3_)_4_	25	1.5	100	-	-	-
2 ^a^	Pd(PPh_3_)_4_	60	48	25	28	26	21
3 ^a^	Pd(PPh_3_)_4_	60 MW	1.0	29	22	23	26
4 ^a^	Pd(PPh_3_)_4_	80 MW	1.0	12	24	27	37
5 ^a^	PdCl_2_(PPh_3_)_2_	60 MW	1.0	48	27	16	9
6 ^a^	Pd(DPPF)Cl_2_	60 MW	1.0	42	37	14	7
7 ^c^	Pd(OAc)_2_	60 MW	1.0	20	60	traces	8
8 ^a^	Pd(OAc)_2_/Xantphos	60 MW	1.0	18	58	9	15
9 ^c^	Pd(OAc)_2_/PPh_3_	60 MW	1.0	16	30	24	14
10 ^c^	Pd(OAc)_2_	80 MW	1.0	5	36	traces	26
11 ^c^	Pd(OAc)_2_	70 MW	1.0	10	41	traces	19
12 ^b,c^	Pd(OAc)_2_	60 MW	2.0	13	64	traces	9

^a^ LC/MS ratio; ^b^ 1.3 equiv. of phenylboronic acid; ^c^ Isolated yields.

**Table 2:**
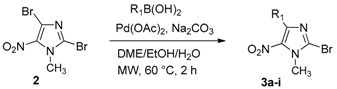
Scope of the regioselective Suzuki-Miyaura reaction.

Compound	R_1_	Yield (%)
**3a**	C_6_H_5_	64
**3b**	*p*-(OCH_3_)-C_6_H_4_	65
**3c ^a^**	*p*-(CF_3_)-C_6_H_4_	62
**3d ^a^**	*m*-(NO_2_)-C_6_H_4_	61
**3e**	*p*-(CN)-C_6_H_4_	64
**3f**	*p*-(F)-C_6_H_4_	62
**3g**	*m*, *m*, *p*-(OCH_3_)-C_6_H_2_	55
**3h**	*p*-(CH_3_)-C_6_H_4_	63
**3i ^a^**	*p*-(CH_2_OH)-C_6_H_4_	58

*Reaction conditions*: boronic acids (1.3 equiv.), Pd(OAc)_2_ (0.04 equiv.), Na_2_CO_3_ (3 equiv.), DME/EtOH/H_2_O (3/1/1), MW, 60 °C, 2 h. ^a^ MW, 65 °C, 2 h.

**Table 3:**
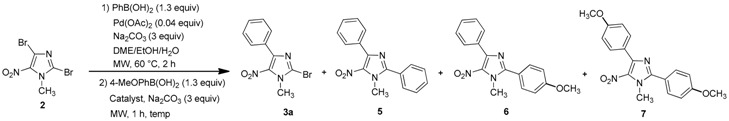
Optimization of the second step of the one-pot regioselective bis-Suzuki-Miyaura reaction.

Entry	Catalyst	Temp (°C)	3a (%)	5 (%)	6 (%)	7 (%)
1	-	60	47	6	22	13
2	Pd(OAc)_2_ 0.04 equiv.	60	35	6	30	16
3	Pd(OAc)_2_ 0.04 equiv.	110	21	6	40	15
4	Pd(PPh_3_)_4_ 0.04 equiv.	110	-	8	60	18
5	PPh_3_ 0.1 equiv.	110	-	12	64	10

**Table 4:**
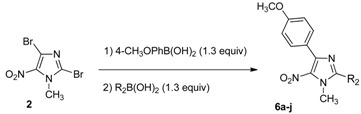
Scope of one-pot regioselective bis-Suzuki-Miyaura reaction. ^a^

Compound	R_2_	Yield (%)
**6a**	C_6_H_5_	71
**6b**	*p*-(F)-C_6_H_4_	70
**6c**	*p*-(Cl)-C_6_H_4_	60
**6d**	*m*-(NO_2_)-C_6_H_4_	65
**6e**	*m*, *m*, *p*-(OCH_3_)-C_6_H_2_	50
**6f**	*m*-(CF_3_)-C_6_H_4_	58
**6g**	*p*-(CH_3_)-C_6_H_4_	56
**6h**	naphthalen-2-yl	61
**6i**	5-methylthiophen-2-yl	52
**6j**	3-pyridine	69

^a^ *Reaction conditions*: Step 1: 4-methoxyphenylboronic acid (1.3 equiv.), Pd(OAc)_2_ (0.04 equiv.), Na_2_CO_3_ (3 equiv.), DME/EtOH/H_2_O (3/1/1), MW, 60 °C, 2 h; Step 2: boronic acid (1.3 equiv.), PPh_3_ (0.1 equiv.), Na_2_CO_3_ (3 equiv.), MW, 110 °C, 1 h.

**Table 5:**
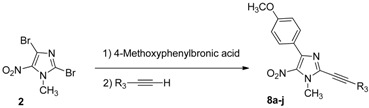
Scope of the one-pot Suzuki-Miyaura/Sonogashira reaction. ^a^

Compound	R_3_	Yield (%)
**8a**	C_6_H_5_	67
**8b**	*m*-(CH_3_)-C_6_H_4_	74
**8c**	*m*-fluoro-C_6_H_4_	59
**8d**	*o*-(CH_3_)-*p*-(OCH_3_)-C_6_H_3_	53
**8e**	cyclopentyl	60
**8f**	*p*-(*tert*-butyl)-C_6_H_4_	63
**8g**	CH_2_OH	51
**8h**	*m*-chloro-C_6_H_4_	61
**8i**	*o*-chloro-C_6_H_4_	52
**8j**	cyclopropyl	63

^a^ *Reaction conditions*: Step 1: 4-methoxyphenylboronic acid (1.3 equiv.), Pd(OAc)_2_ (0.04 equiv.), Na_2_CO_3_ (3 equiv.), DME/EtOH/H_2_O (3/1/1), MW, 60 °C, 2 h; Step 2: alkyne (1.3 equiv.), PPh_3_ (0.1 equiv.), Et_3_N (2 equiv.), CuI (0.1 equiv.), MW, 70 °C, 1 h.

**Table 6:** Antibacterial activity of molecules **3a**–**3i** obtained with the first regioselective Suzuki-Miyaura reaction.

Bacterial Species	Compounds									
	3a	3b	3c	3d	3e	3f	3g	3h	3i	mtz
*C. butyricum Nec 0*	14	38	0	26	18	14	27	21	0	>30
*C. perfringens*	>20	0	0	0	0	0	0	0	0	<20
*C. neonatale*	25	29	14	>20	24	28	30	25	22	32
*C. difficile*	>20	31	15	10	25	28	23	29	32	>20
*B. circulans*	0	0	0	0	0	0	0	0	0	<20
*E. coli*	0	0	0	0	0	0	0	0	0	0

Inhibition zone diameter (mm).

**Table 7:** Antibacterial activity of molecules **6a**–**j** obtained with the one-pot regioselective bis-Suzuki-Miyaura reaction.

Bacterial Species	Compounds								
	6a	6c	6d	6e	6g	6h	6i	6j	mtz
*C. butyricum Nec 0*	24	20	29	14	0	0	25	>20	>30
*C. perfringens*	0	0	0	0	0	>20	0	0	<20
*C. neonatale*	22	>20	>20	>20	14	0	>20	>20	32
*C. difficile*	23	12	14	14	16	13	0	15	>20
*B. circulans*	0	0	0	0	0	0	0	0	<20
*E. coli*	0	0	0	0	0	0	0	0	0

Inhibition zone diameter (mm).

**Table 8:** Antibacterial activity of molecules **8a**–**j** obtained with the one-pot Suzuki-Miyaura/ Sonogashira reaction.

Bacterial Species	Compounds									
	8a	8b	8c	8d	8e	8g	8h	8i	8j	mtz
*C. butyricum Nec 0*	0	25	0	0	17	17	8	18	23	>30
*C. perfringens*	0	0	0	24	0	0	>20	0	0	<20
*C. neonatale*	11	>20	29	0	15	23	0	13	18	32
*C. difficile*	0	13	0	0	17	20	0	0	19	>20
*B. circulans*	0	0	0	0	0	0	0	0	0	<20
*E. coli*	0	0	0	0	0	0	0	0	0	0

Inhibition zone diameter (mm).

**Table 9:** Antibacterial activity of the most promising molecules.

Bacterial Species	Compounds							
	2	3b	3e	3f	3g	3h	3i	mtz
*B. cereus*	0	10.9	9.6	9.6	10.9	10.7	11.5	0
*B. beijerenckii*	0	0	0	9	0	0	0	11
*C. butyricum Nec 0*	0	29.4	24.6	26.7	30.4	27.6	30.2	31
*C. butyricum Nec 1*	0	32.8	27	29.8	32.2	29.3	34.1	34
*C. butyricum Nec 8*	0	31.3	24.8	27.8	34.5	27.8	32	30
*C. tetani*	0	0	0	0	0	0	0	56
*C. perfringens*	0	0	20	0	26	0	29	30
*C. difficile O27*	5.8	32.4	35	30.6	33.7	29.7	32.8	37

Inhibition zone diameter (mm).

**Table 10:** Antiparasitic evaluation on *T. vaginalis*.

Compounds	Activity *T. vaginalis* MIC (µM)	Cytotoxicity CC_50_ (µM)	Selectivity Index ^a^
**3b**	4.00	2.58	0.64
**3e**	4.07	-	-
**3f**	1.04	1.72	1.65
**3g**	0.84	2.59	3.08
**3h**	1.05	3.59	3.42
**3i**	0.99	1.13	1.13
**mtz**	7.30	557.34	18.65

^a^ Selectivity index calculated according to the formula: IC_50_/MIC.

**Table 11:** Cytotoxicity evaluation on Chinese Hamster Ovary cells.

Compounds	Cytotoxicity CC_50_ (µM)
**3a**	1.89
**3b**	2.58
**3d**	1.87
**3f**	1.72
**3g**	2.59
**3h**	3.59
**3i**	1.13
**6a**	3.21
**6c**	1.41
**6d**	6.94
**6e**	0.90
**6g**	2.97
**6h**	0.83
**6i**	1.48
**6j**	1.24
**8a**	2.65
**8b**	1.33
**8c**	1.35
**8d**	0.43
**8e**	2.39
**8g**	0.27
**8h**	4.57
**8i**	2.73
**8j**	1.17
**mtz**	557.34

## Supplementary Materials

Supplementary Materials are available online. Figures S1–S66: ^1^H- and ^13^C-NMR of all compounds **3a**–**i**, **5**, **6a**–**j**, **6**, **7**, **8a**–**j** and **9**.

## References

[1] Citron D.M., Tyrrell K.L., Warren Y.A., Fernandez H., Merriam C.V., Goldstein E.J.C. (2005). In vitro activities of tinidazole and metronidazole against *Clostridium difficile*, *Prevotella bivia* and *Bacteroides fragilis*. Anaerobe.

[2] Ravdin J.I. (1995). Amebiasis. Clin. Infect. Dis..

[3] Kim P., Kang S., Boshoff H.I., Jiricek J., Collins M., Singh R., Manjunatha U.H., Niyomrattanakit P., Zhang L., Goodwin M. (2009). Structure−Activity Relationships of Antitubercular Nitroimidazoles. 2. Determinants of Aerobic Activity and Quantitative Structure−Activity Relationships. J. Med. Chem..

[4] Leitsch D., Kolarich D., Wilson I.B.H., Altmann F., Duchêne M. (2007). Nitroimidazole Action in *Entamoeba histolytica*: A Central Role for Thioredoxin Reductase. PLoS Biol..

[5] Löfmark S., Edlund C., Nord C.E. (2010). Metronidazole is still the drug of choice for treatment of anaerobic infections. Clin. Infect. Dis..

[6] Duan Y.-T., Yao Y.-F., Huang W., Makawana J.A., Teraiya S.B., Thumar N.J., Tang D.-J., Tao X.-X., Wang Z.-C., Jiang A.-Q. (2014). Synthesis, biological evaluation, and molecular docking studies of novel 2-styryl-5-nitroimidazole derivatives containing 1,4-benzodioxan moiety as FAK inhibitors with anticancer activity. Bioorg. Med. Chem..

[7] Leitsch D. (2016). Recent Advances in the *Trichomonas vaginalis* Field. F1000Research.

[8] Sisson G., Goodwin A., Raudonikiene A., Hughes N.J., Mukhopadhyay A.K., Berg D.E., Hoffman P.S. (2002). Enzymes Associated with Reductive Activation and Action of Nitazoxanide, Nitrofurans, and Metronidazole in *Helicobacter pylori*. Antimicrob. Agents Chemother..

[9] Kulda J. (1999). Trichomonads, hydrogenosomes and drug resistance. Int. J. Parasitol..

[10] Pal D., Banerjee S., Cui J., Schwartz A., Ghosh S.K., Samuelson J. (2009). *Giardia*, *Entamoeba*, and *Trichomonas* Enzymes Activate Metronidazole (Nitroreductases) and Inactivate Metronidazole (Nitroimidazole Reductases). Antimicrob. Agents Chemother..

[11] Leitsch D., Kolarich D., Binder M., Stadlmann J., Altmann F., Duchêne M. (2009). *Trichomonas vaginalis*: metronidazole and other nitroimidazole drugs are reduced by the flavin enzyme thioredoxin reductase and disrupt the cellular redox system. Implications for nitroimidazole toxicity and resistance. Mol. Microbiol..

[12] Upcroft P., Upcroft J.A. (2001). Drug Targets and mechanisms of resistance in the anaerobic protozoa. Clin. Microbiol. Rev..

[13] Land K.M.L., Clemens D.L., Johnson P.J. (2001). Loss of multiple hydrogenosomal proteins associated with organelle metabolism and high-Level drug resistance in trichomonads. Exp. Parasitol..

[14] Hrdy I., Cammack R., Stopka P., Kulda J., Tachezy J. (2005). Alternative pathway of metronidazole activation in *Trichomonas vaginalis* hydrogenosomes. Antimicrob. Agents Chemother..

[15] Leitsch D., Drinić M., Kolarich D., Duchêne M. (2012). Down-regulation of flavin reductase and alcohol dehydrogenase-1 (ADH1) in metronidazole-resistant isolates of *Trichomonas vaginalis*. Mol. Biochem. Parasitol..

[16] Lemée V., Zaharia I., Nevez G., Rabodonirina M., Brasseur P., Ballet J.J., Favennec L. (2000). Metronidazole and albendazole susceptibility of 11 clinical isolates of *Giardia duodenalis* from France. J. Antimicrob. Chemother..

[17] Solaymani-Mohammadi S., Genkinger J.M., Loffredo C.A., Singer S.M. (2010). A meta-analysis of the effectiveness of albendazole compared with metronidazole as treatments for infections with *Giardia duodenalis*. PLoS Negl. Trop. Dis..

[18] Nabarro L.E.B., Lever R.A., Armstrong M., Chiodini P.L. (2015). Increased incidence of nitroimidazole-refractory giardiasis at the Hospital for Tropical Diseases, London: 2008–2013. Clin. Microbiol. Infect..

[19] Breuil J., Dublanchet A., Truffaut N., Sebald M. (1989). Transferable 5-nitroimidazole resistance in the *Bacteroides fragilis* group. Plasmid.

[20] Tomb J.-F., White O., Kerlavage A.R., Clayton R.A., Sutton G.G., Fleischmann R.D., Ketchum K.A., Klenk H.P., Gill S., Dougherty B.A. (1997). The complete genome sequence of the gastric pathogen *Helicobacter pylori*. Nature.

[21] Schapiro J.M., Gupta R., Stefansson E., Fang F.C., Limaye A.P. (2004). Isolation of metronidazole-resistant *Bacteroides fragilis* carrying the nima nitroreductase gene from a patient in washington state. J. Clin. Microbiol..

[22] Graham D.Y., Fischbach L. (2010). *Helicobacter pylori* treatment in the era of increasing antibiotic resistance. Gut.

[23] Secka O., Berg D.E., Antonio M., Corrah T., Tapgun M., Walton R., Thomas V., Galano J.J., Sancho J., Adegbola R.A. (2013). Antimicrobial susceptibility and resistance patterns among *Helicobacter pylori* strains from the Gambia, West Africa. Antimicrob. Agents Chemother..

[24] Lossick J.G., Müller M., Gorrell T.E. (1986). In vitro drug susceptibility and doses of metronidazole required for cure in cases of refractory vaginal trichomoniasis. J. Infect. Dis..

[25] Gardner T.B., Hill D.R. (2001). Treatment of Giardiasis. Clin. Microbiol. Rev..

[26] Lindmark D.G., Müller M. (1976). Antitrichomonad action, mutagenicity, and reduction of metronidazole and other nitroimidazoles. Antimicrob. Agents Chemother..

[27] Voogd C.E. (1981). On the mutagenicity of nitroimidazoles. Mutat. Res. Genet. Toxicol..

[28] De Méo M., Vanelle P., Bernadini E., Laget M., Maldonado J., Jentzer O., Crozet M.P., Duménil G. (1992). Evaluation of the mutagenic and genotoxic activities of 48 nitroimidazoles and related imidazole derivatives by the Ames test and the SOS Chromotest. Environ. Mol. Mutagen..

[29] Ré J.L., De Méo M., Laget M., Guiraud H., Castegnaro M., Vanelle P., Duménil G. (1997). Evaluation of the genotoxic activity of metronidazole and dimetridazole in human lymphocytes by the comet assay. Mutat. Res. Mol. Mech. Mutagen..

[30] Trinh S., Reysset G. (1998). Mutagenic action of 5-nitroimidazoles: in vivo induction of GC-->CG transversion in two *Bacteroides fragilis* reporter genes. Mutat. Res. Mol. Mech. Mutagen..

[31] Upcroft J.A., Dunn L.A., Wright J.M., Benakli K., Upcroft P., Vanelle P. (2006). 5-Nitroimidazole drugs effective against metronidazole-resistant *Trichomonas vaginalis* and *Giardia duodenalis*. Antimicrob. Agents Chemother..

[32] Falagas M.E., Walker A.M., Jick H., Ruthazer R., Griffith J., Snydman D.R. (1998). Late incidence of cancer after metronidazole use: A matched metronidazole user/nonuser study. Clin. Infect. Dis..

[33] Ferraris L., Schönherr S., Bouvet P., Dauphin B., Popoff M., Butel M.J., Aires J. (2015). One-Step Multiplex PCR assay for differentiating proposed new species “*clostridium neonatale*” from closely related species. J. Clin. Microbiol..

[34] Cassir N., Benamar S., La Scola B. (2016). *Clostridium butyricum*: From beneficial to a new emerging pathogen. Clin. Microbiol. Infect..

[35] Aljarallah K.M. (2017). Conventional and alternative treatment approaches for *Clostridium difficile* infection. Int. J. Health Sci..

[36] Olivo G., Lucas T.M., Borges A.S., Silva R.O.S., Lobato F.C.F., Siqueira A.K., Da Silva Leite D., Brandão P.E., Gregori F., De Oliveira-Filho J.P. (2016). Enteric pathogens and coinfections in foals with and without diarrhea. Biomed Res. Int..

[37] Crozet M.P., Surzur J.-M., Vanelle P., Ghiglione C., Maldonado J. (1985). S_RN_1 reactions of heterocyclic compounds. II. Reactivity of 1-methyl-2-chloromethyl-5-nitroimidazole. Tetrahedron Lett..

[38] Vanelle P., Maldonado J., Crozet M., Senouki K., Delmas F., Gasquet M., Timon-David P. (1991). Preparation and antiparasitic activity of new imidazoles bearing dioxane or hexahydropyrimidine moiety. Eur. J. Med. Chem..

[39] Jentzer O., Vanelle P., Crozet M., Maldonado J., Barreau M. (1991). New highly active 5-nitroimidazoles bearing lactam nuclei: Synthesis and antibacterial properties. Eur. J. Med. Chem..

[40] Vanelle P., Benakli K., Maldonado J., Crozet M.P. (1998). An electron-transfer reaction in the imidazo[2,1-*b*]thiazole series. Heterocycles.

[41] Upcroft J.A., Campbell R.W., Benakli K., Upcroft P., Vanelle P. (1999). Efficacy of new 5-nitroimidazoles against metronidazole-susceptible and -resistant *Giardia*, *Trichomonas*, and *Entamoeba* spp.. Antimicrob. Agents Chemother..

[42] Chauvière G., Viodé C., Périé J. (2000). Nucleophilic substitution studies on nitroimidazoles, and applications to the synthesis of biologically active compounds. J. Heterocycl. Chem..

[43] Benakli K., Terme T., Vanelle P. (2002). Synthesis of new active sulfones in the 5-nitroimidazole series. Molecules.

[44] Valdez C.A., Tripp J.C., Miyamoto Y., Kalisiak J., Hruz P., Andersen Y.S., Brown S.E., Kangas K., Arzu L.V., Davids B.J. (2009). Synthesis and electrochemistry of 2-ethenyl and 2-ethanyl derivatives of 5-nitroimidazole and antimicrobial activity against *Giardia lamblia*. J. Med. Chem..

[45] Crozet M.D., Botta C., Gasquet M., Curti C., Rémusat V., Hutter S., Chapelle O., Azas N., De Méo M., Vanelle P. (2009). Lowering of 5-nitroimidazole’s mutagenicity: towards optimal antiparasitic pharmacophore. Eur. J. Med. Chem..

[46] Dunn L.A., Burgess A.G., Krauer K.G., Eckmann L., Vanelle P., Crozet M.D., Gillin F.D., Upcroft P., Upcroft J.A. (2010). A new-generation 5-nitroimidazole can induce highly metronidazole-resistant *Giardia lamblia* in vitro. Int. J. Antimicrob. Agents.

[47] Miyamoto Y., Kalisiak J., Korthals K., Lauwaet T., Cheung D.Y., Lozano R., Cobo E.R., Upcroft P., Upcroft J.A., Berg D.E. (2013). Expanded therapeutic potential in activity space of next-generation 5-nitroimidazole antimicrobials with broad structural diversity. Proc. Natl. Acad. Sci. USA.

[48] Neildé K., Crozet M.D., Terme T., Vanelle P. (2014). First single electron transfer reaction on propargylic chloride in 5-nitroimidazole series. Tetrahedron Lett..

[49] Walsh J.S., Wang R., Bagan E., Wang C.C., Wislocki P., Miwa G.T. (1987). Structural alterations that differentially affect the mutagenic and antitrichomonal activities of 5-nitroimidazoles. J. Med. Chem..

[50] Crozet M., Terme T., Vanelle P. (2013). Designing new 5-nitroimidazoles: Towards safer anti-infectious agents. Lett. Drug Des. Discov..

[51] Magano J., Dunetz J.R. (2011). Large-scale applications of transition metal-catalyzed couplings for the synthesis of pharmaceuticals. Chem. Rev..

[52] Johansson Seechurn C.C.C., Kitching M.O., Colacot T.J., Snieckus V. (2012). Palladium-catalyzed cross-coupling: A historical contextual perspective to the 2010 Nobel Prize. Angew. Chem. Int. Ed..

[53] Schröter S., Stock C., Bach T. (2005). Regioselective cross-coupling reactions of multiple halogenated nitrogen-, oxygen-, and sulfur-containing heterocycles. Tetrahedron.

[54] Fairlamb I.J.S. (2007). Regioselective (site-selective) functionalisation of unsaturated halogenated nitrogen, oxygen and sulfur heterocycles by Pd-catalysed cross-couplings and direct arylation processes. Chem. Soc. Rev..

[55] Handy S.T., Zhang Y. (2006). A simple guide for predicting regioselectivity in the coupling of polyhaloheteroaromatics. Chem. Commun..

[56] Iaroshenko V.O., Gevorgyan A., Mkrtchyan S., Arakelyan K., Grigoryan T., Yedoyan J., Villinger A., Langer P. (2015). Transition-metal-catalyzed arylation of nitroimidazoles and further transformations of manipulable nitro group. J. Org. Chem..

[57] Revesz L., Bonne F., Makavou P. (1998). Vicinal bromostannanes as novel building blocks for the preparation of di- and trisubstituted imidazoles. Tetrahedron Lett..

[58] Revesz L., Di Padova F.E., Buhl T., Feifel R., Gram H., Hiestand P., Manning U., Wolf R., Zimmerlin A.G. (2002). SAR of 2,6-diamino-3,5-difluoropyridinyl substituted heterocycles as novel p38 MAP kinase inhibitors. Bioorg. Med. Chem. Lett..

[59] Neildé K., Crozet M., Terme T., Vanelle P. (2013). Synthesis of 4-alkynyl-1,2-dimethyl-5-nitro-1*H*-imidazoles via microwave-assisted sonogashira cross-coupling reactions. Synthesis.

[60] Zink L., Crozet M.D., Terme T., Vanelle P. (2011). Long distance-S_RN_1 in nitroimidazole series favored by temperature. Tetrahedron Lett..

[61] Pedada S.R., Satam V.S., Tambade P.J., Kandadai S.A., Hindupur R.M., Pati H.N., Launay D., Martin D. (2013). An Improved Kilogram-scale synthesis of 2-Bromo-4-nitro-1*H*-imidazole: A key building block of nitroimidazole Drugs. Org. Process Res. Dev..

[62] (2017). European Committee on Antimicrobial Susceptibility Testing, Version 7.1. Http://eucast.org.

[63] Prieto P., Cole T., Curren R., Gibson R.M., Liebsch M., Raabe H., Tuomainen A.M., Whelan M., Kinsner-Ovaskainen A. (2013). Assessment of the predictive capacity of the 3T3 Neutral Red Uptake cytotoxicity test method to identify substances not classified for acute oral toxicity (LD50 > 2000 mg/kg): Results of an ECVAM validation study. Regul. Toxicol. Pharmacol..

