# Supplementary material for: An Efficient One-Pot Catalyzed Synthesis of 2,4-Disubstituted 5-Nitroimidazoles Displaying Antiparasitic and Antibacterial Activities
Source: Molecules. 2017 Aug 3;22(8):1278. doi: 10.3390/molecules22081278 (PMC6152245; doi:10.3390/molecules22081278)
Supplement: Supplementary file 1 [file molecules-22-01278-s001.pdf]

Supplementary Materials: An efficient one-pot catalyzed synthesis of 2,4-disubstituted 5-nitroimidazole displaying antiparasitic and antibacterial activities.

F. Mathias[1], Y. Kabri,[1] L. Okdah[2], C. Di Giorgio[3], J.-M. Rolain[2], C. Spitz[1], M. D. Crozet[1] and P. Vanelle[1]

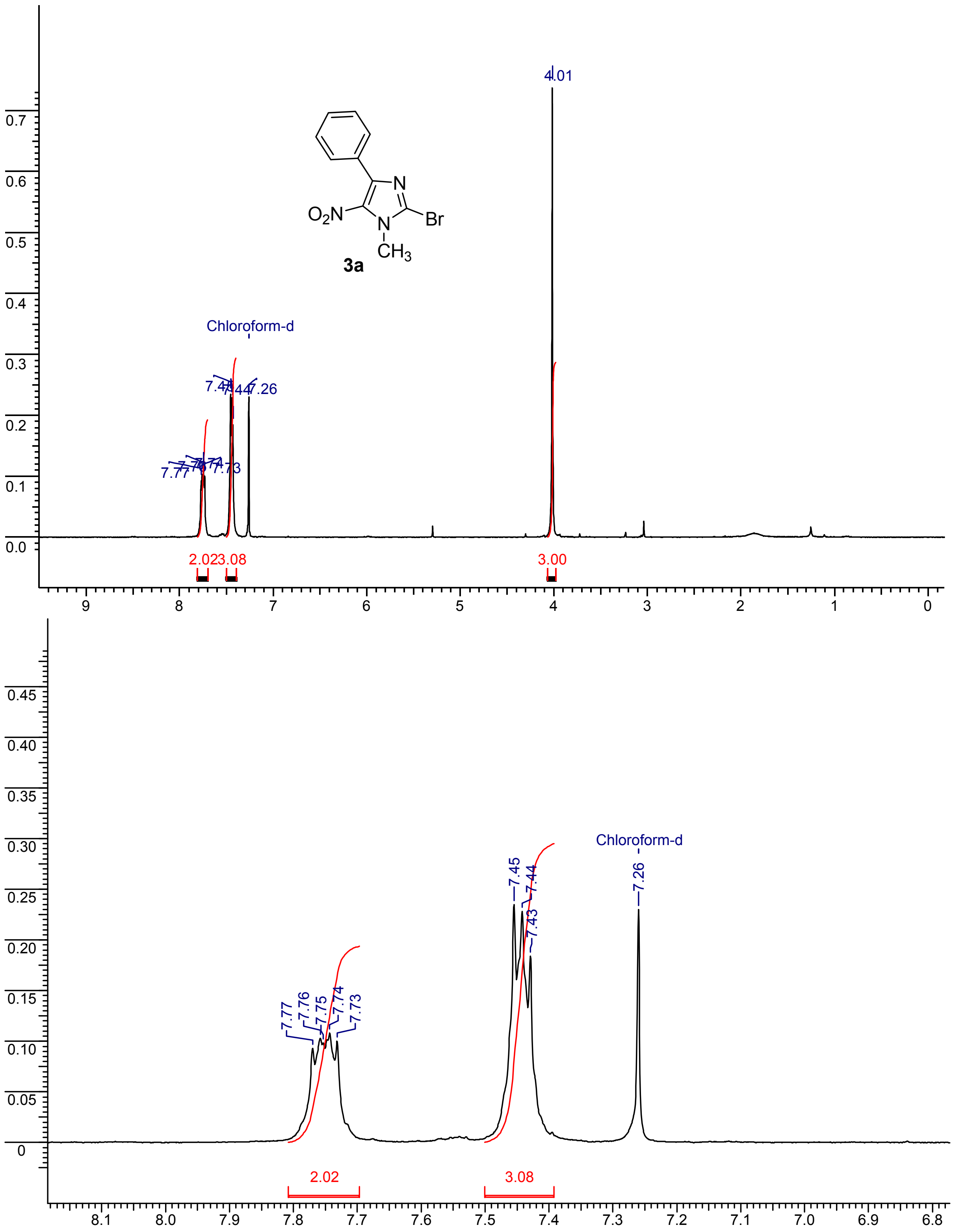

Figure S1. ^{1}H-NMR spectra of **3a**

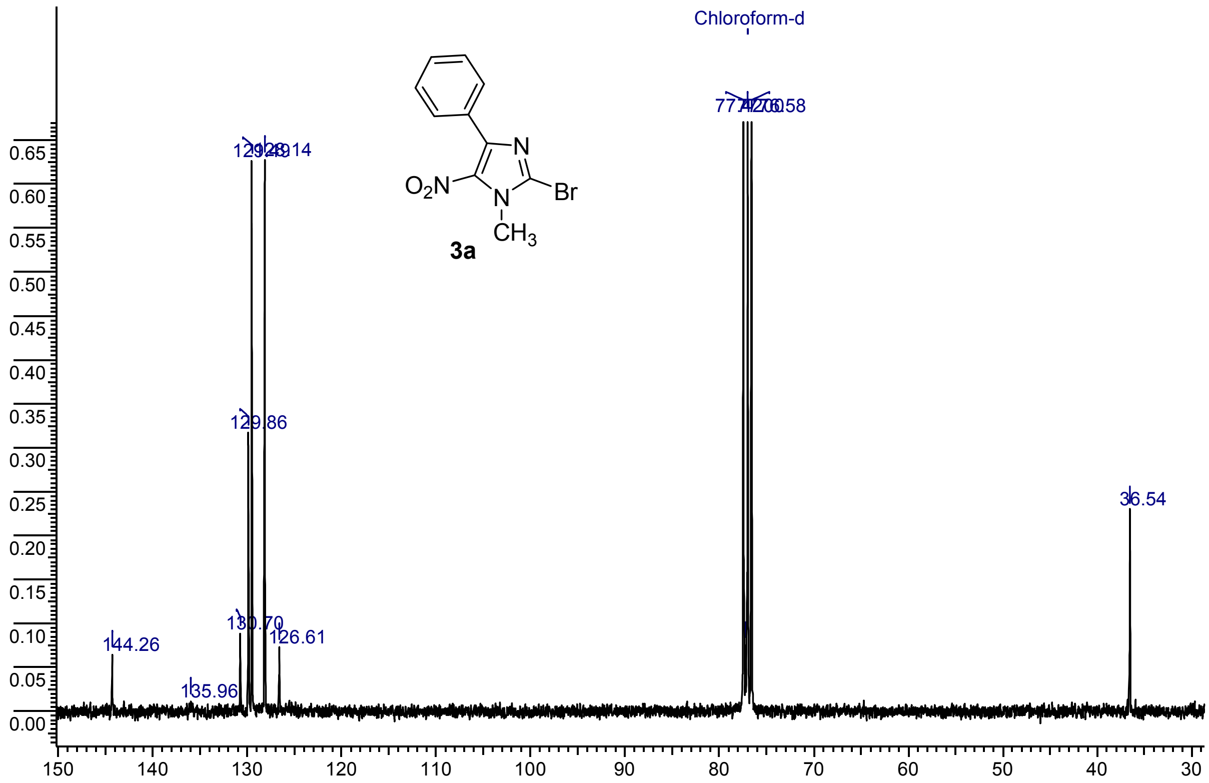

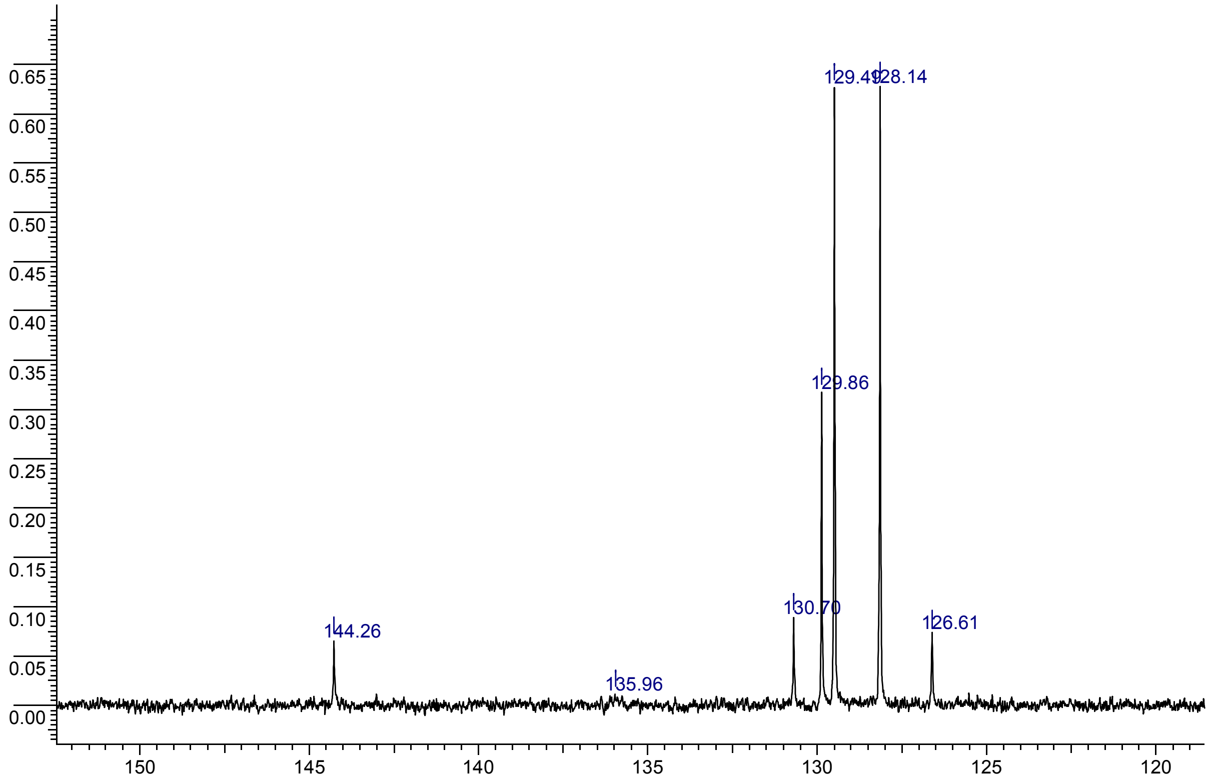

Figure S2. ^{13}C-NMR spectra of **3a**

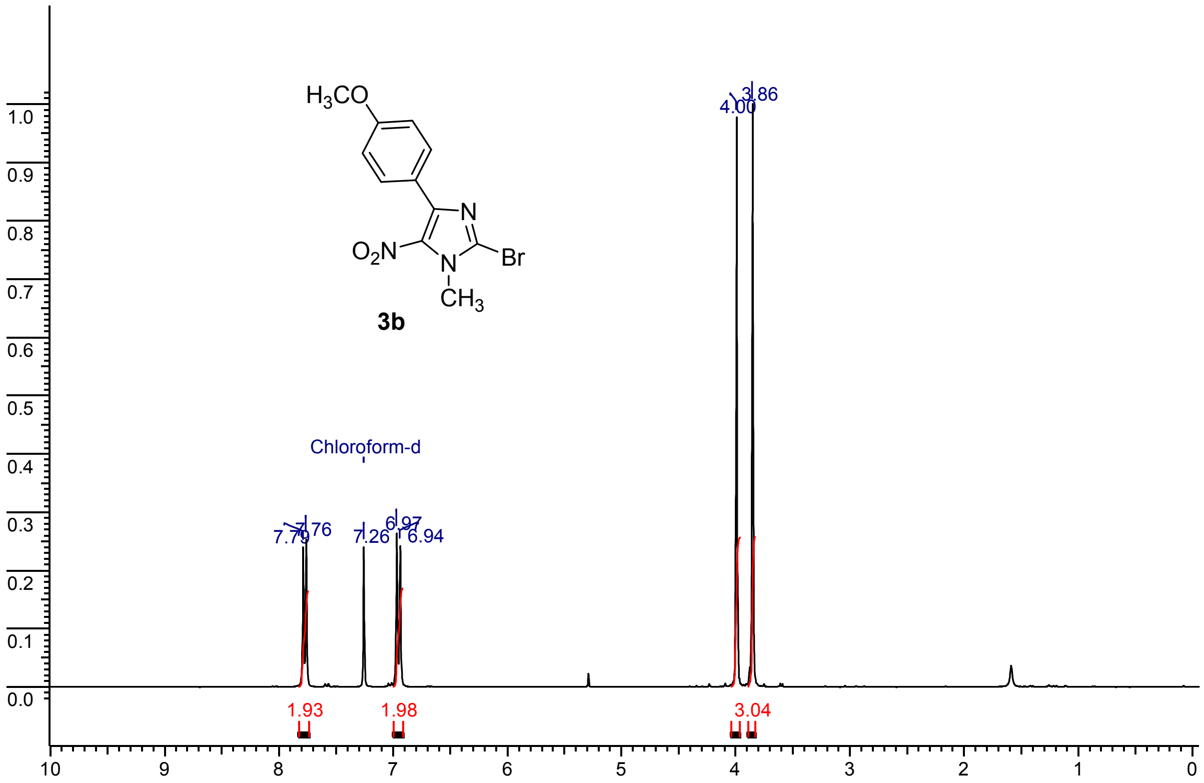

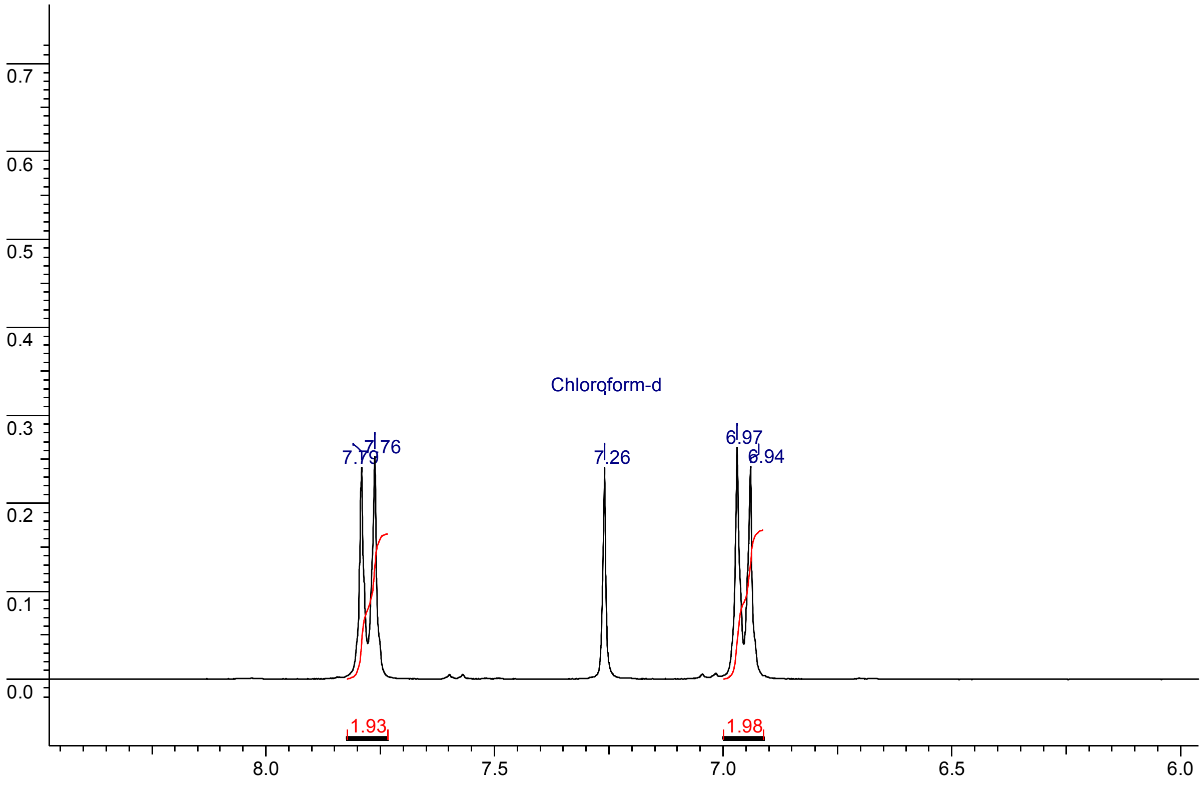

Figure S3. ^{1}H-NMR spectra of **3b**

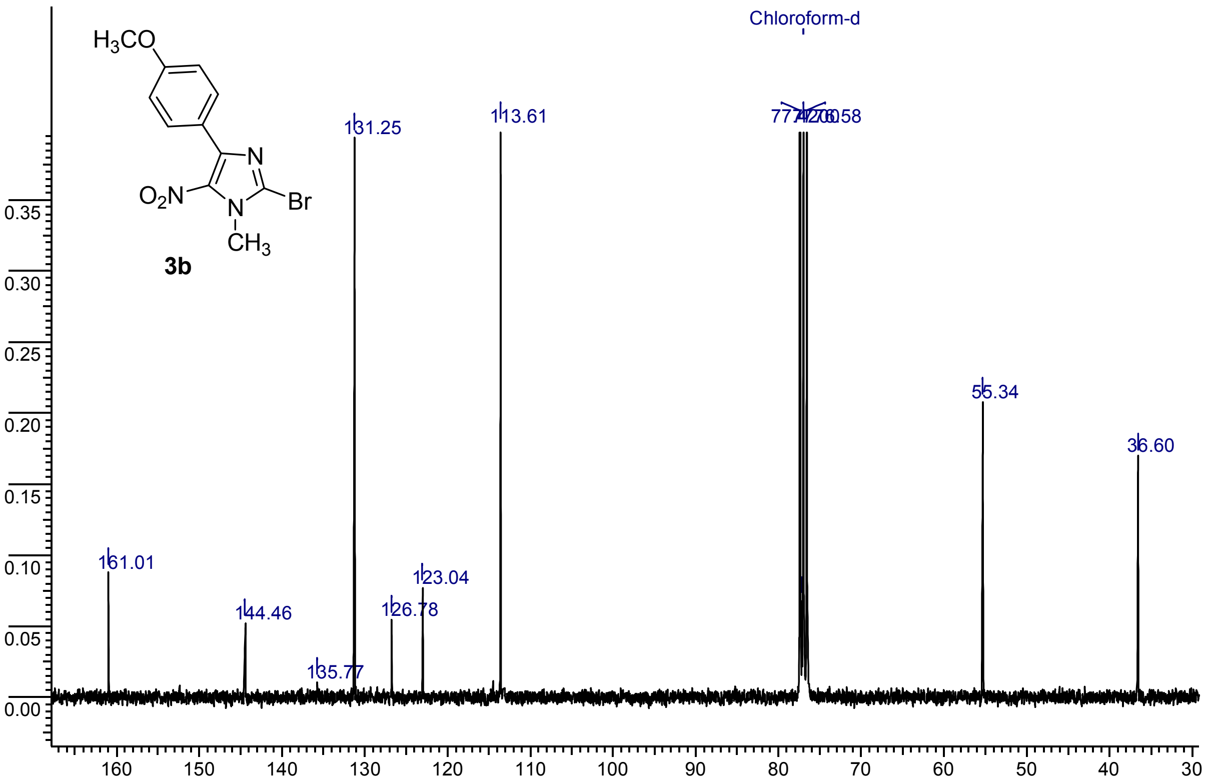

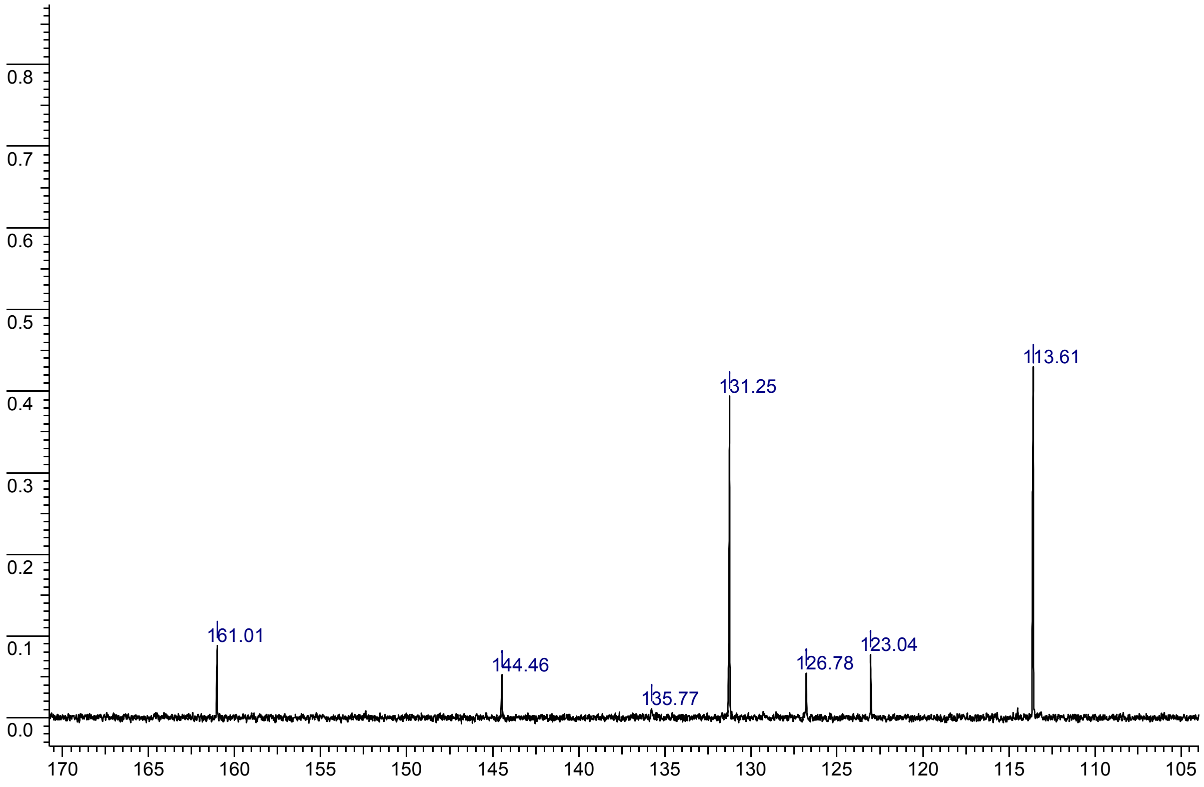

Figure S4. ^{13}C-NMR spectra of **3b**

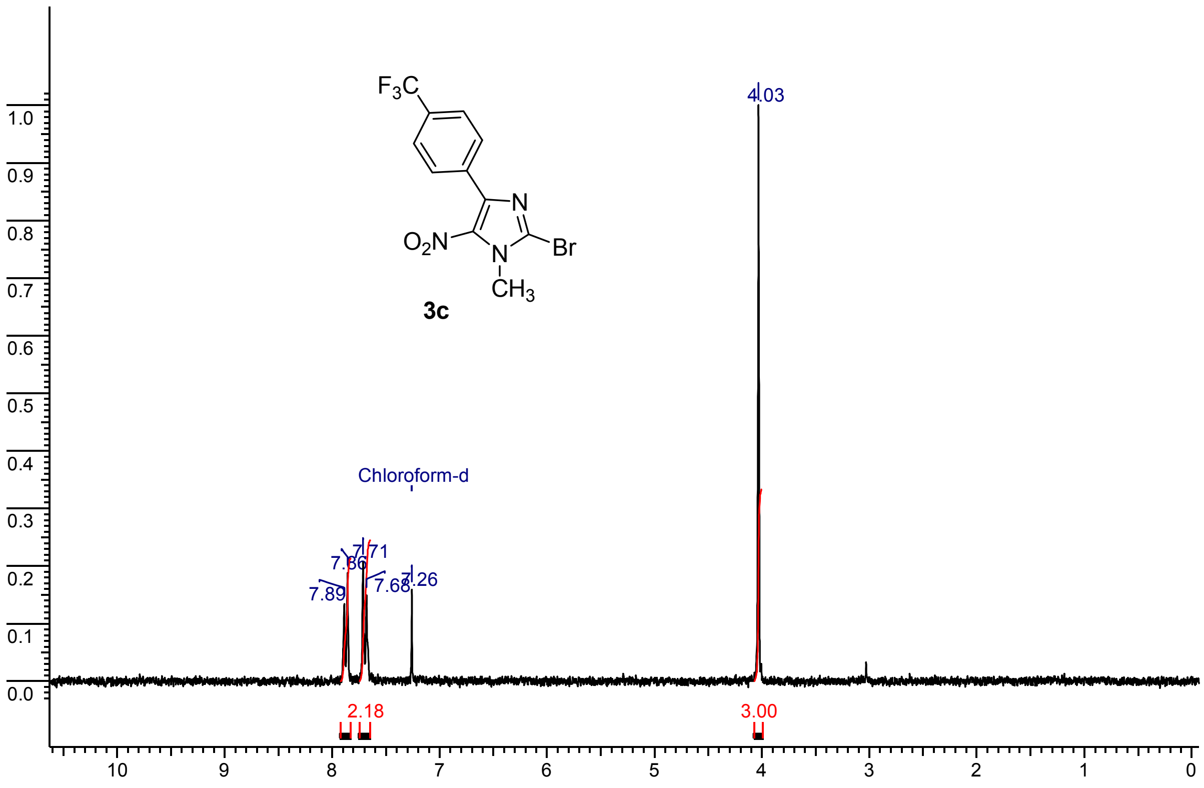

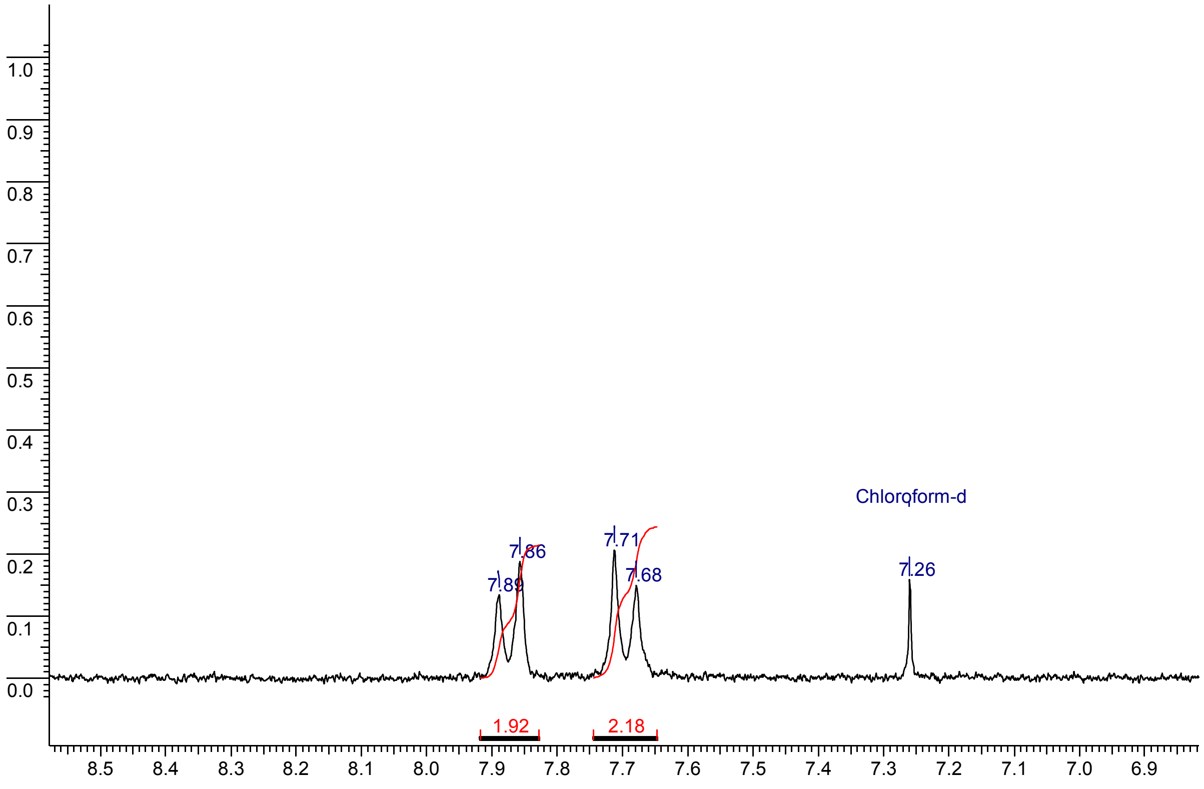

Figure S5. ^{1}H-NMR spectra of **3c**

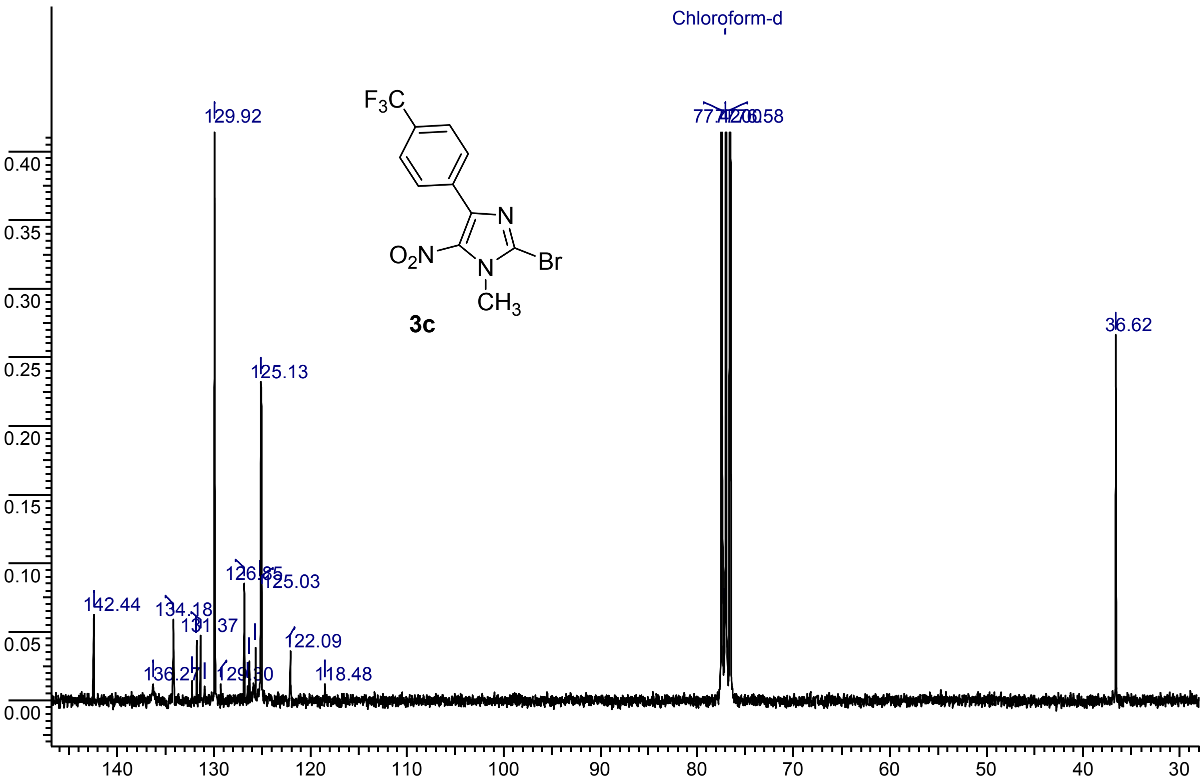

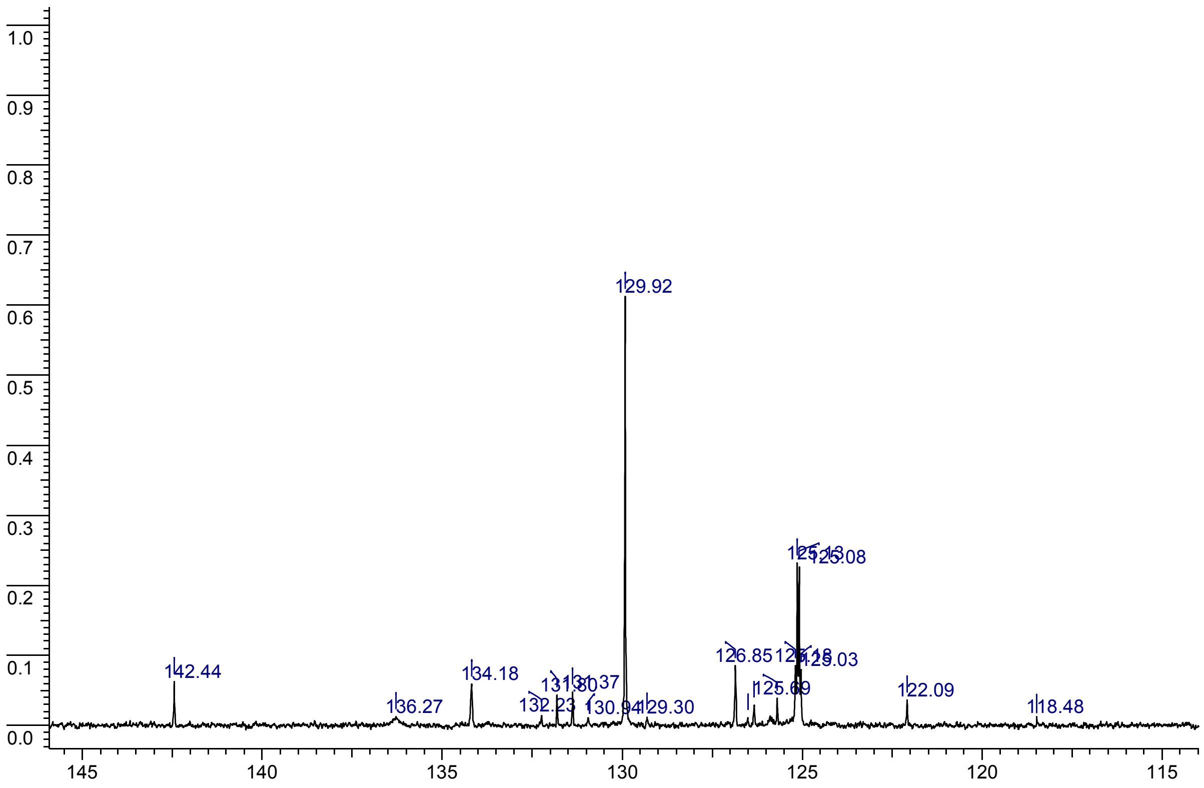

Figure S6. ^{13}C-NMR spectra of **3c**

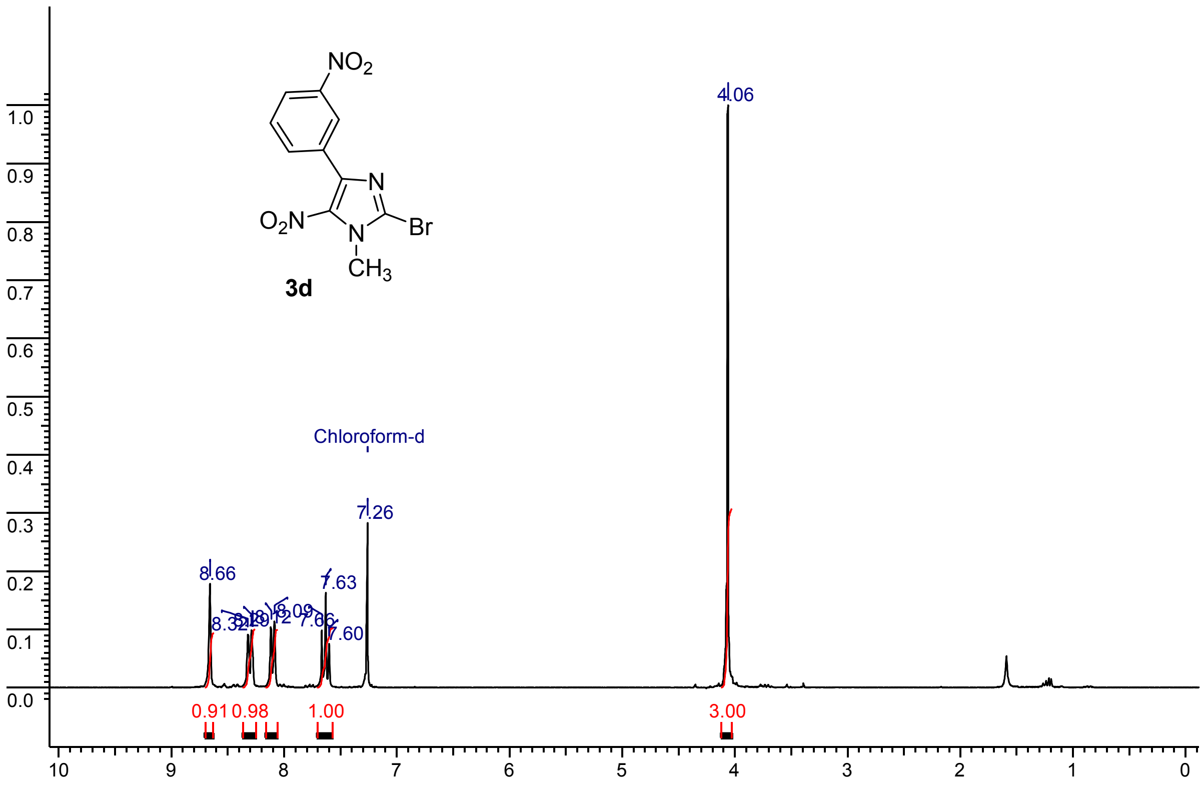

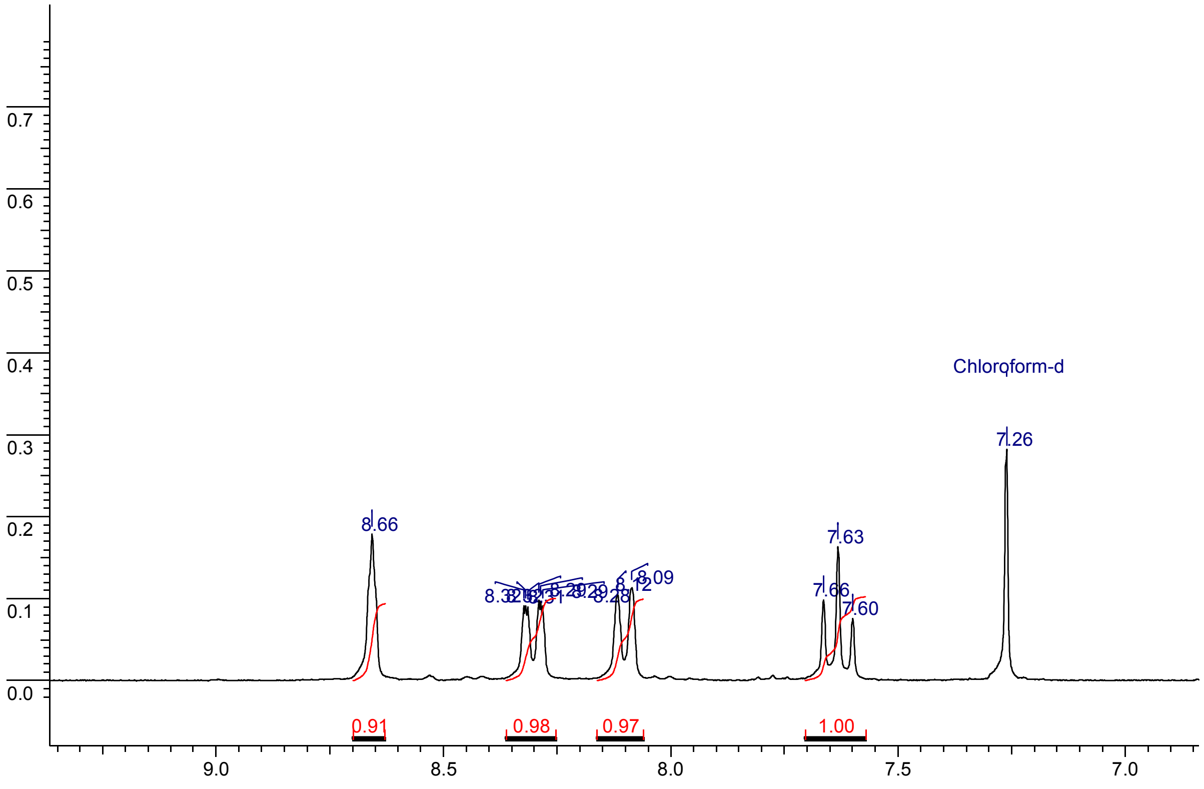

Figure S7. ^{1}H-NMR spectra of **3d**

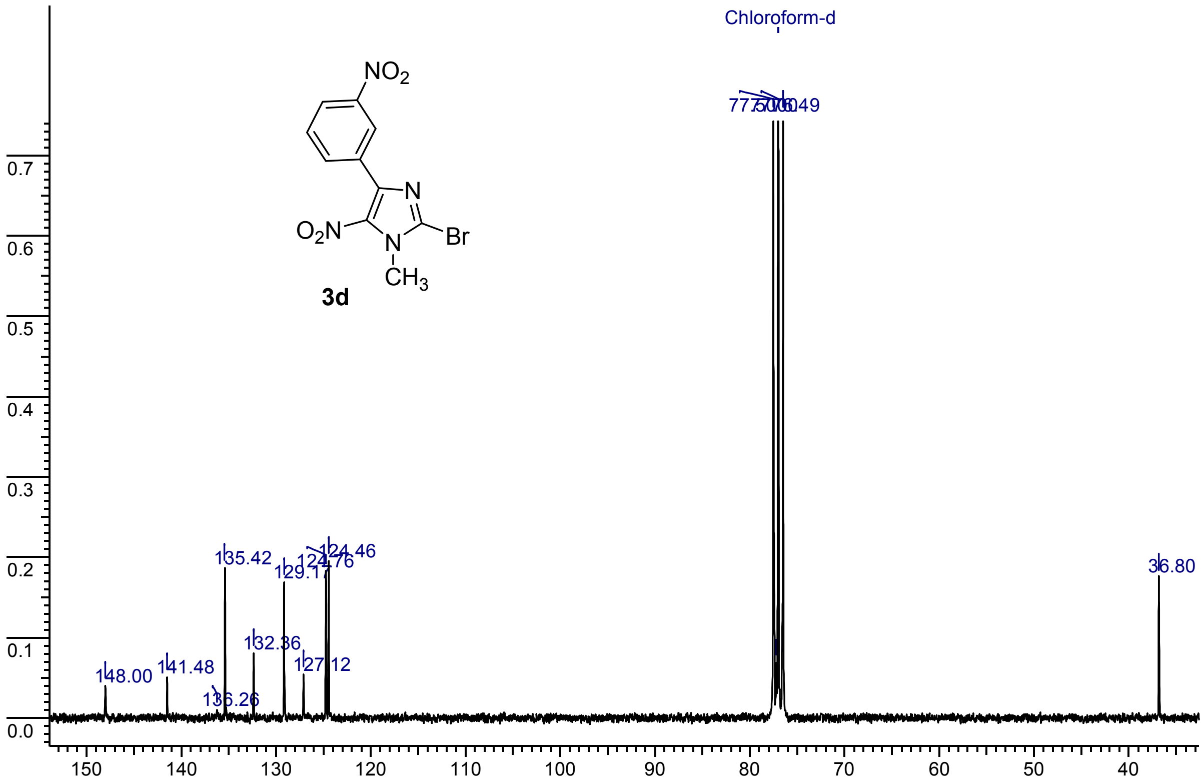

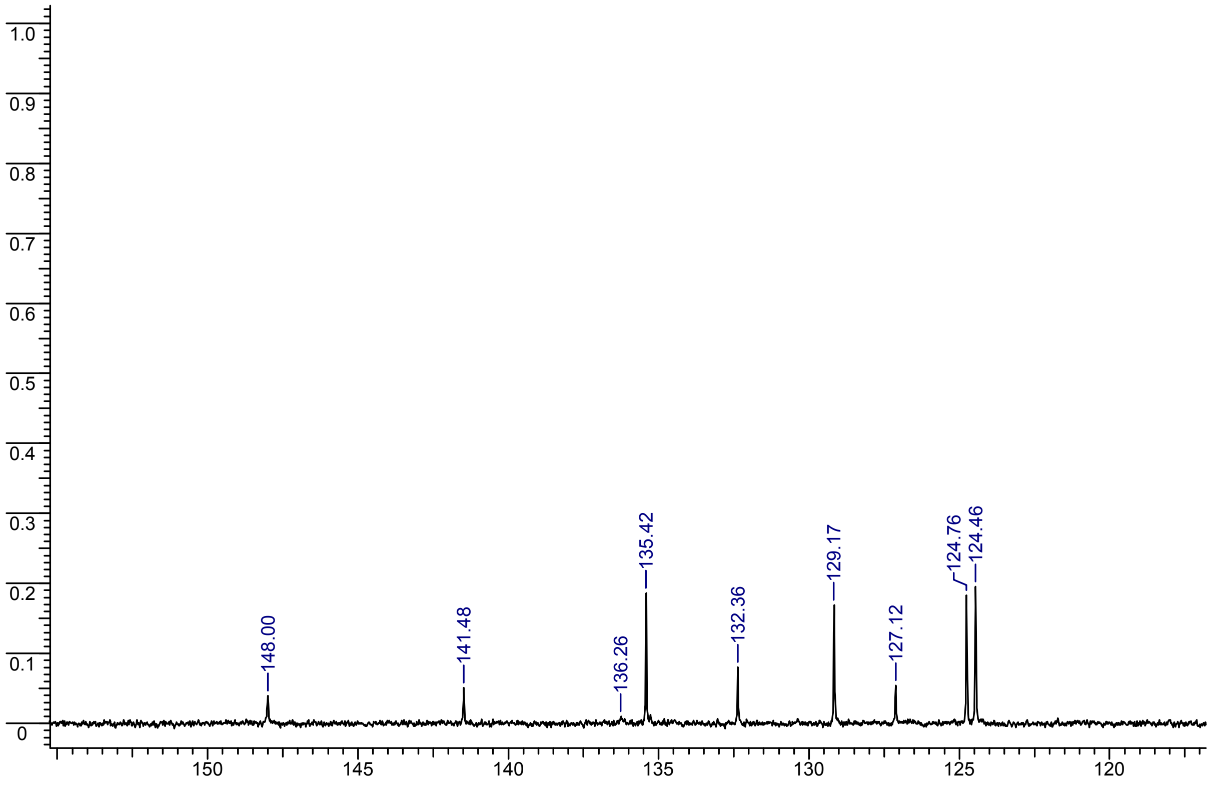

Figure S8. ^{13}C-NMR spectra of **3d**

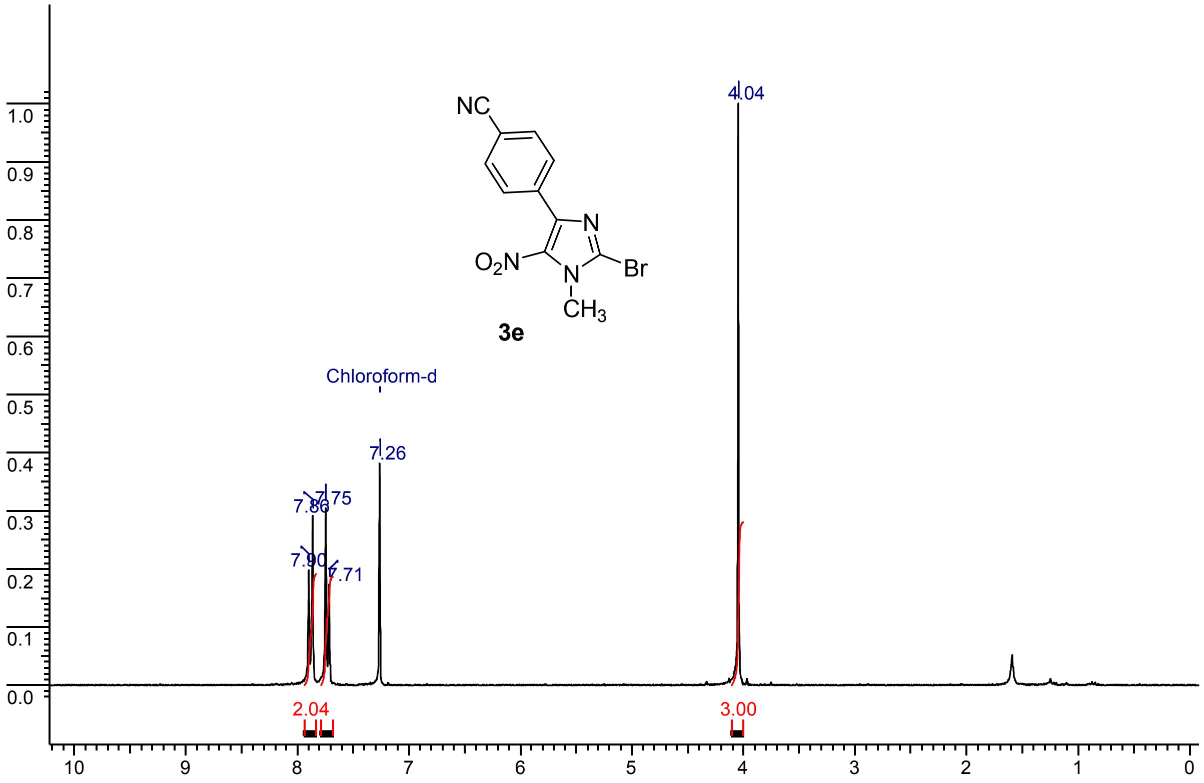

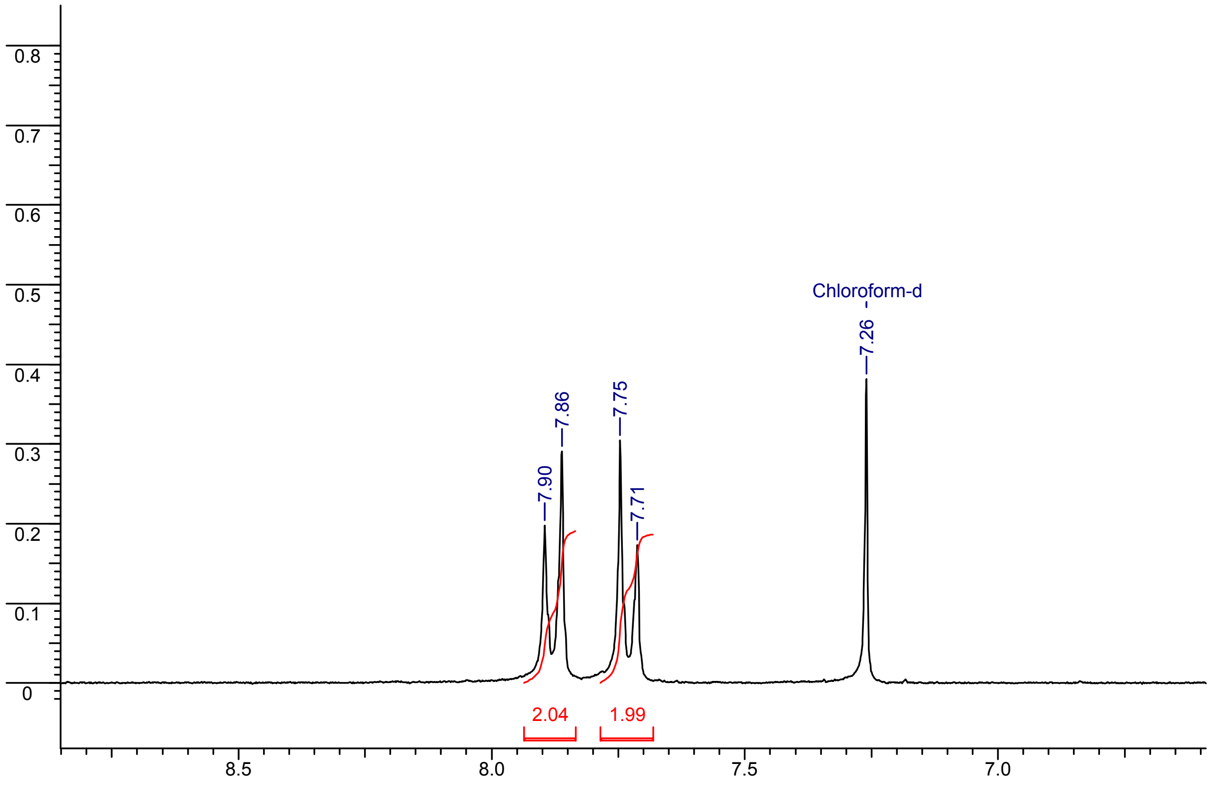

Figure S9. ^{1}H-NMR spectra of **3e**

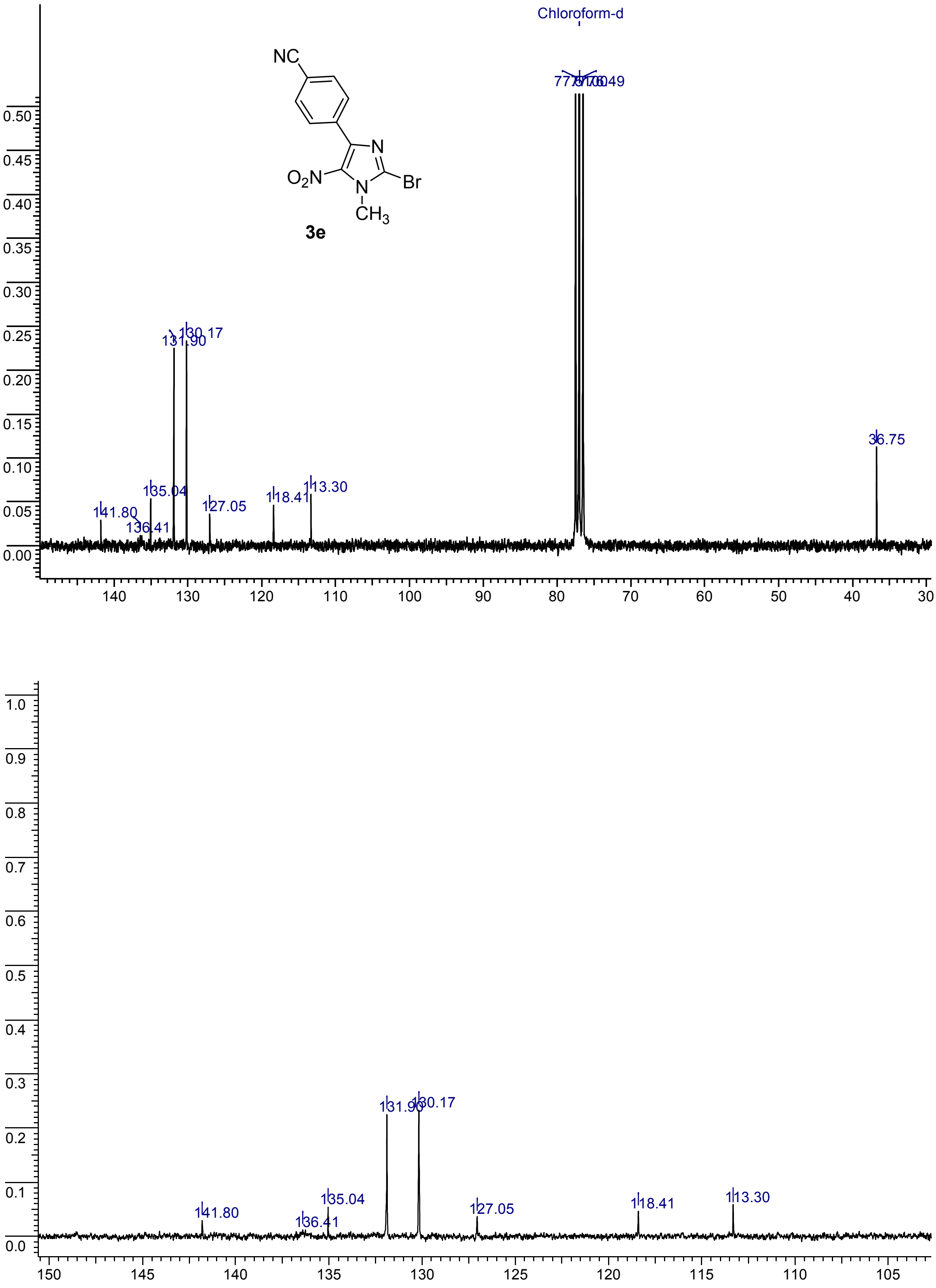

Figure S10. ^{13}C-NMR spectra of **3e**

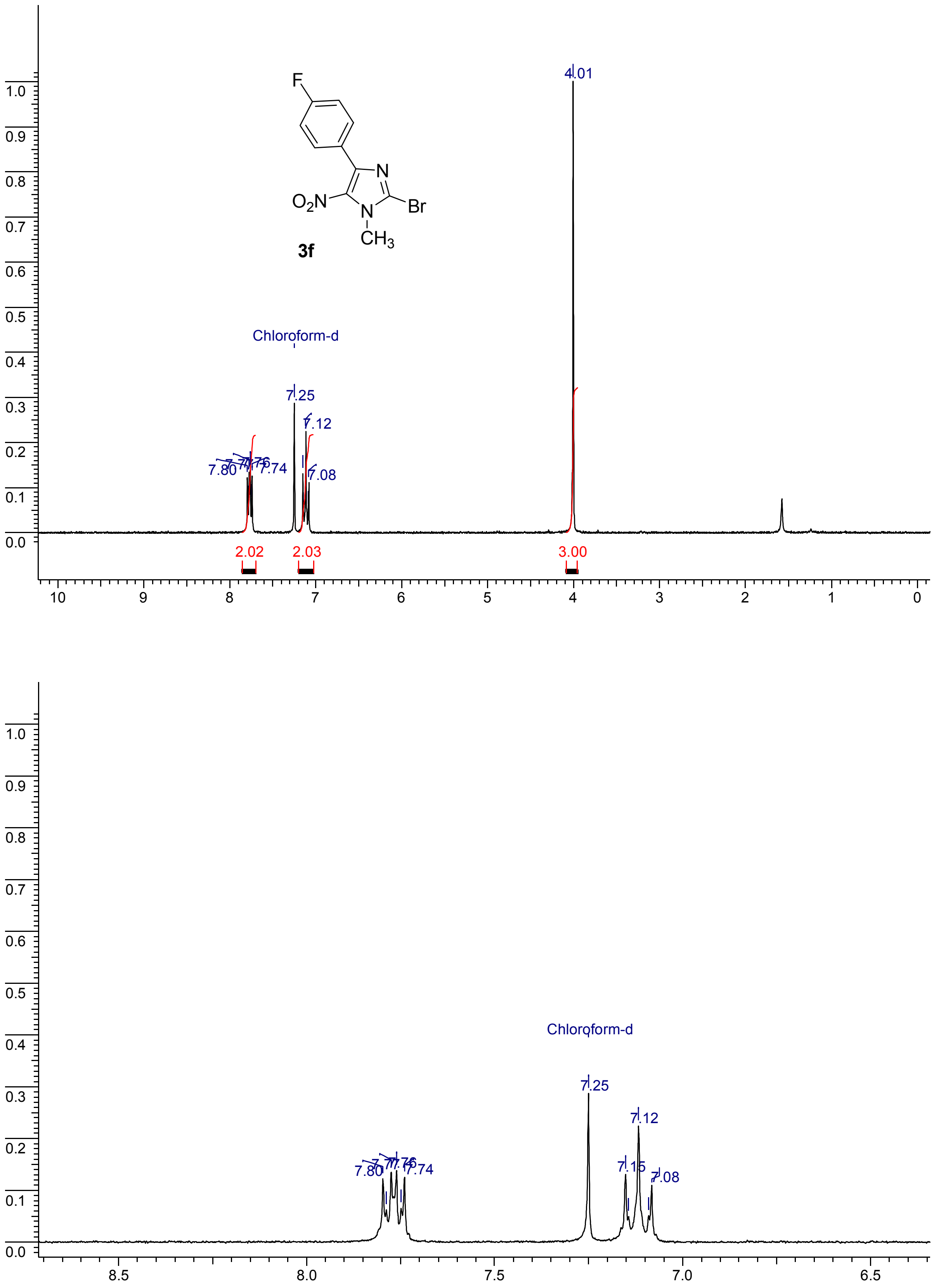

Figure S11. ^{1}H-NMR spectra of **3f**

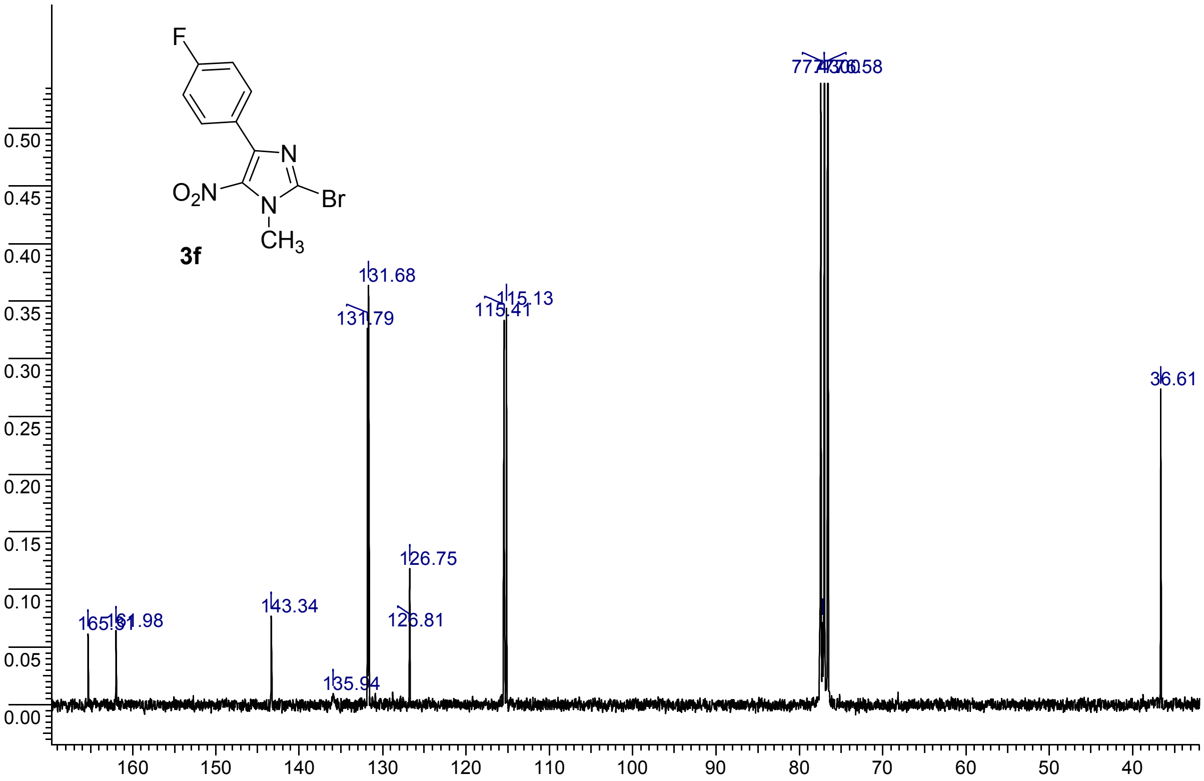

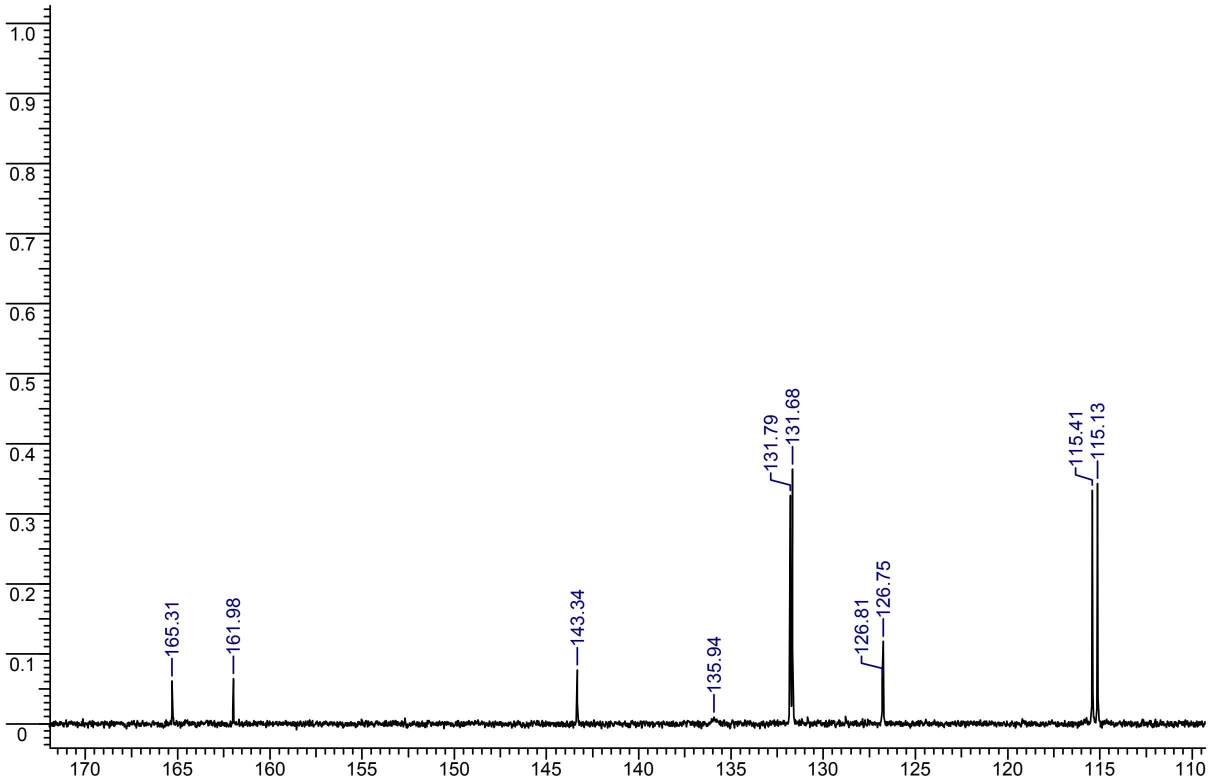

Figure S12. ^{13}C-NMR spectra of **3f**

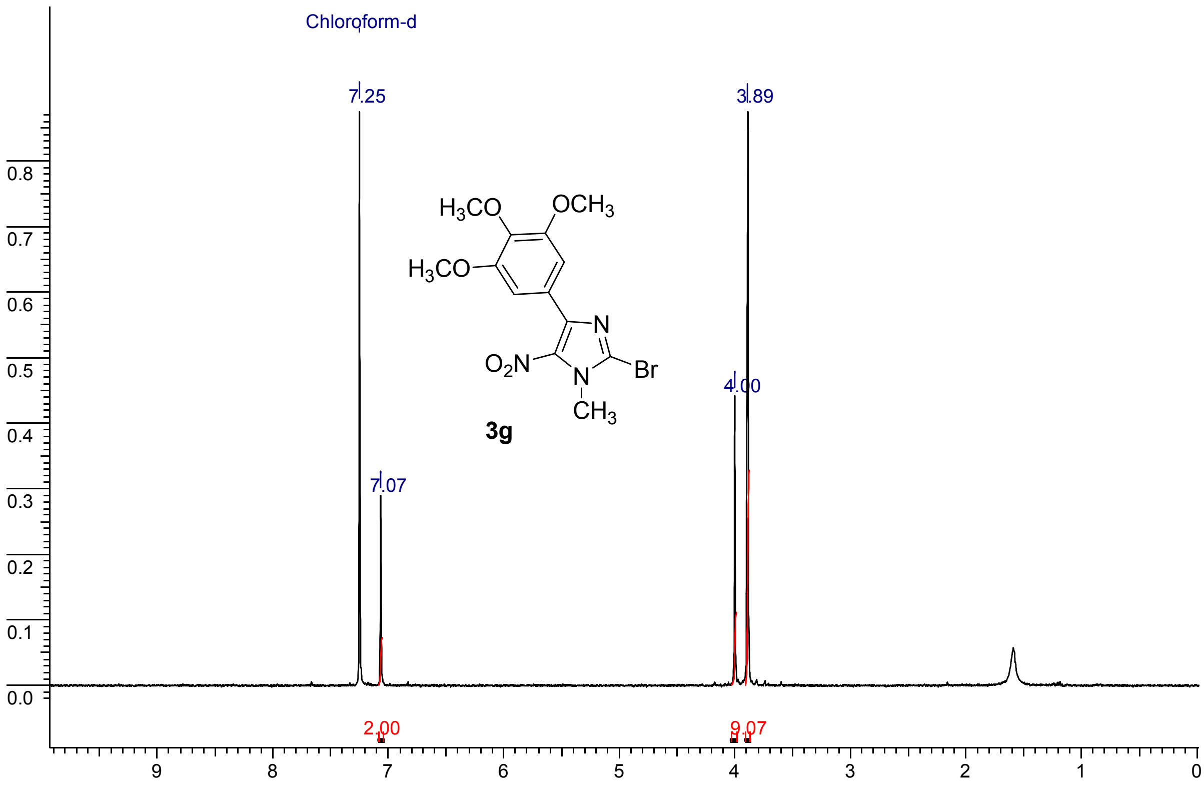

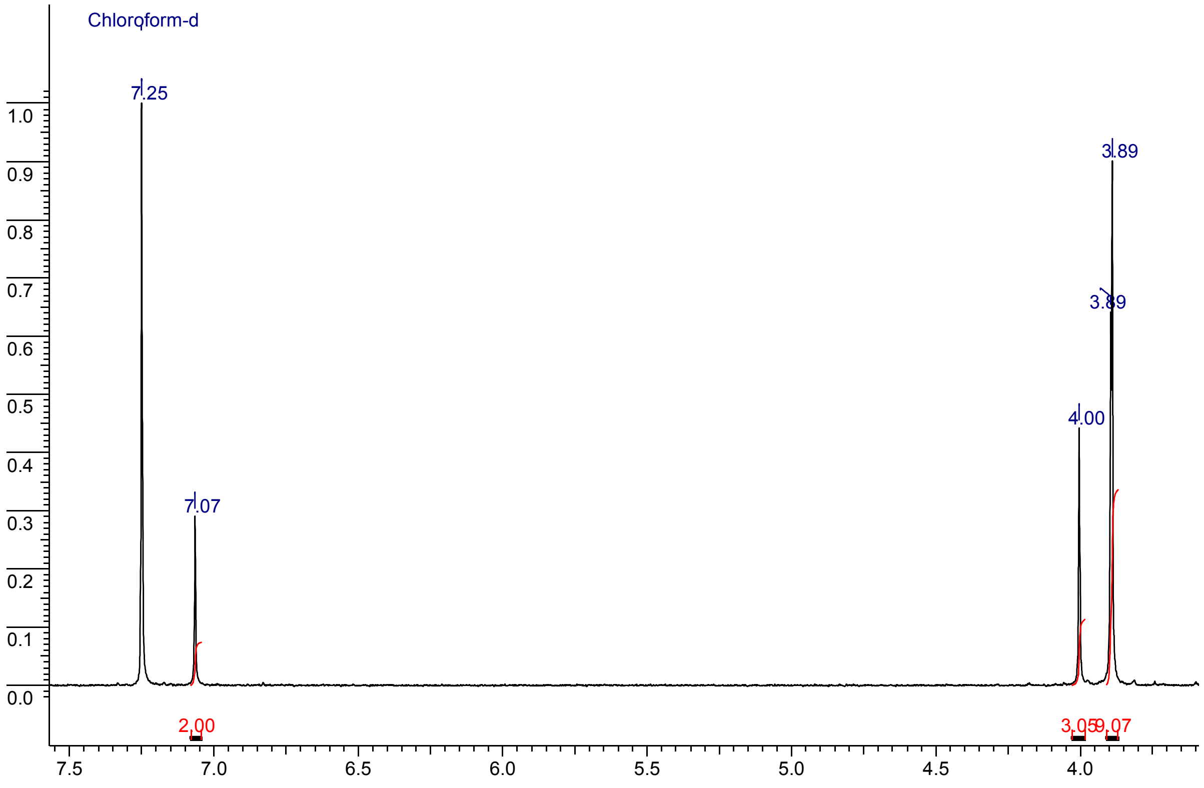

Figure S13. ^{1}H-NMR spectra of **3g**

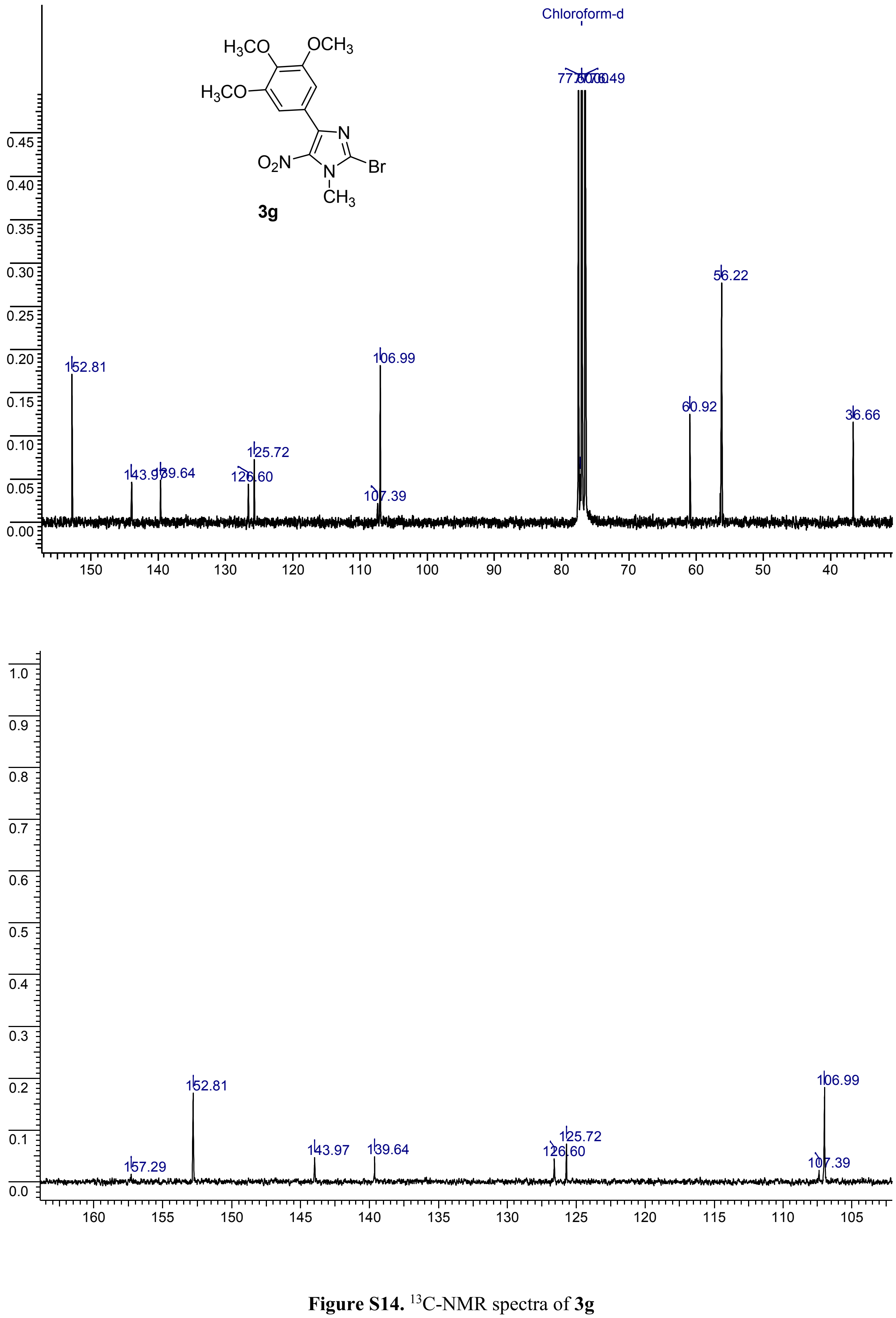

Figure S14. ^{13}C-NMR spectra of **3g**

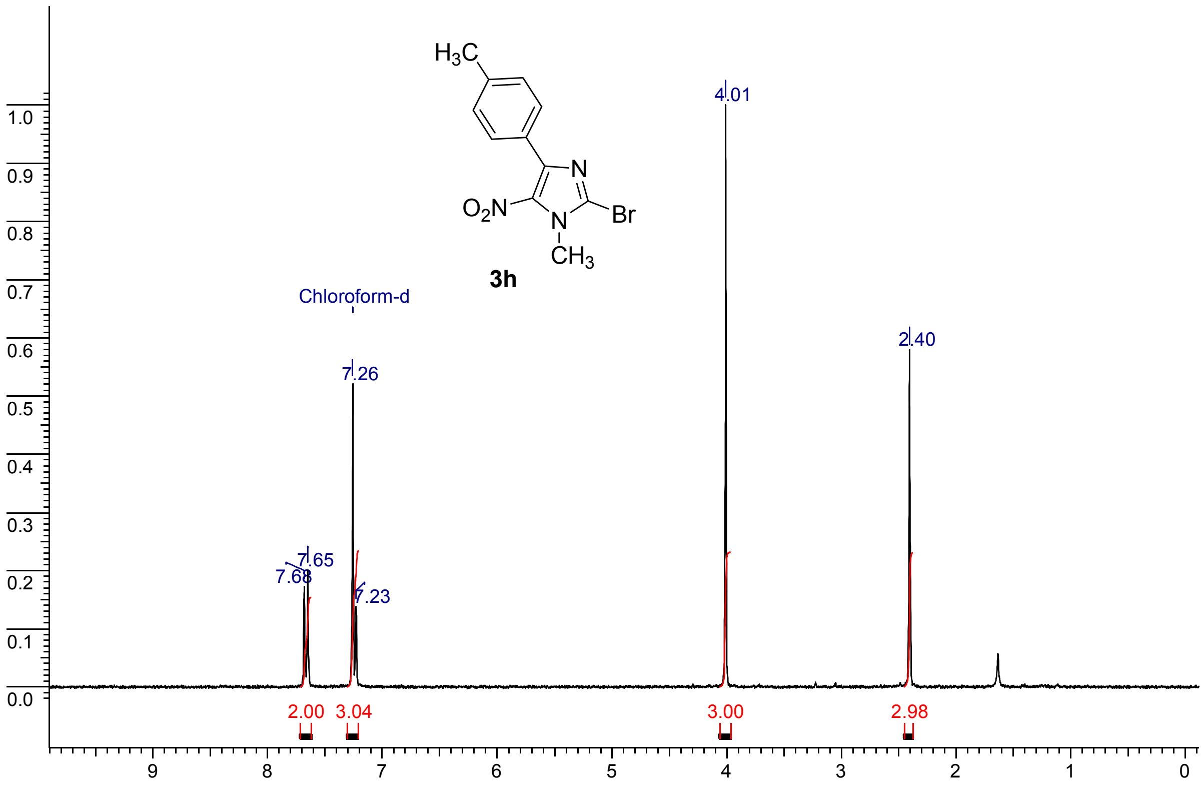

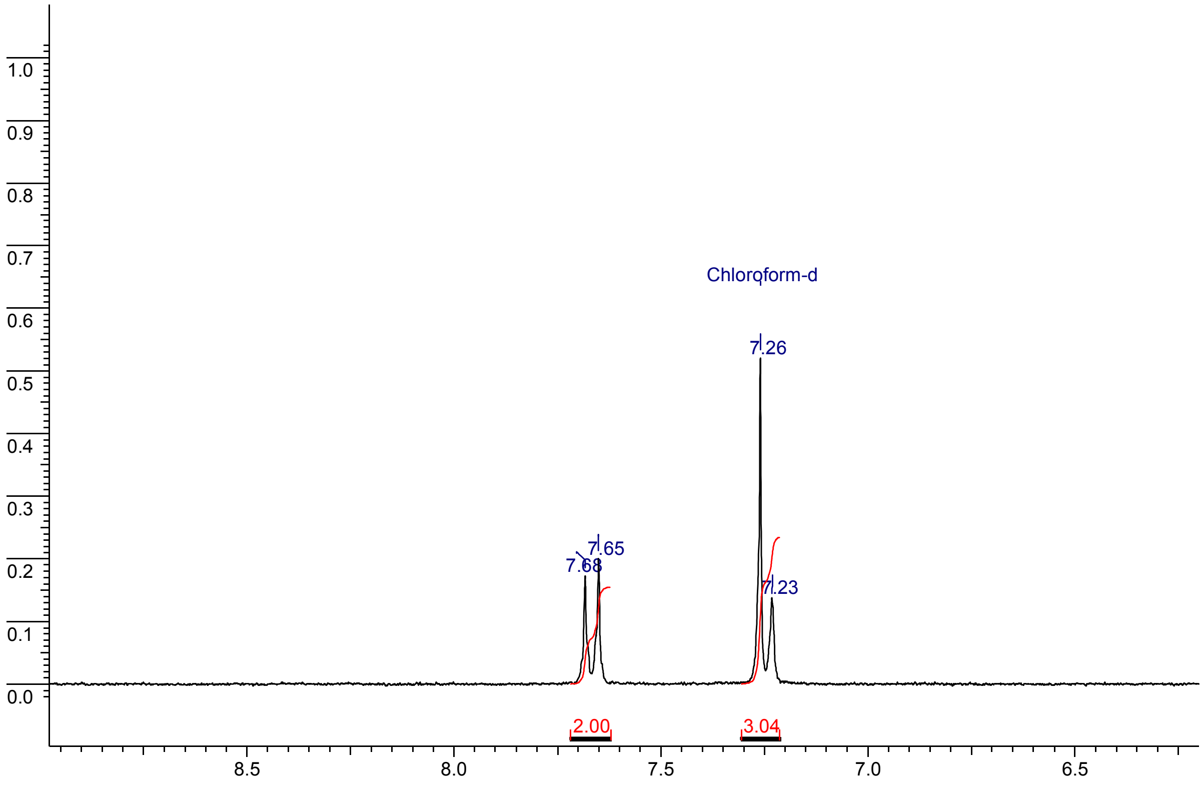

Figure S15. ^{1}H-NMR spectra of **3h**

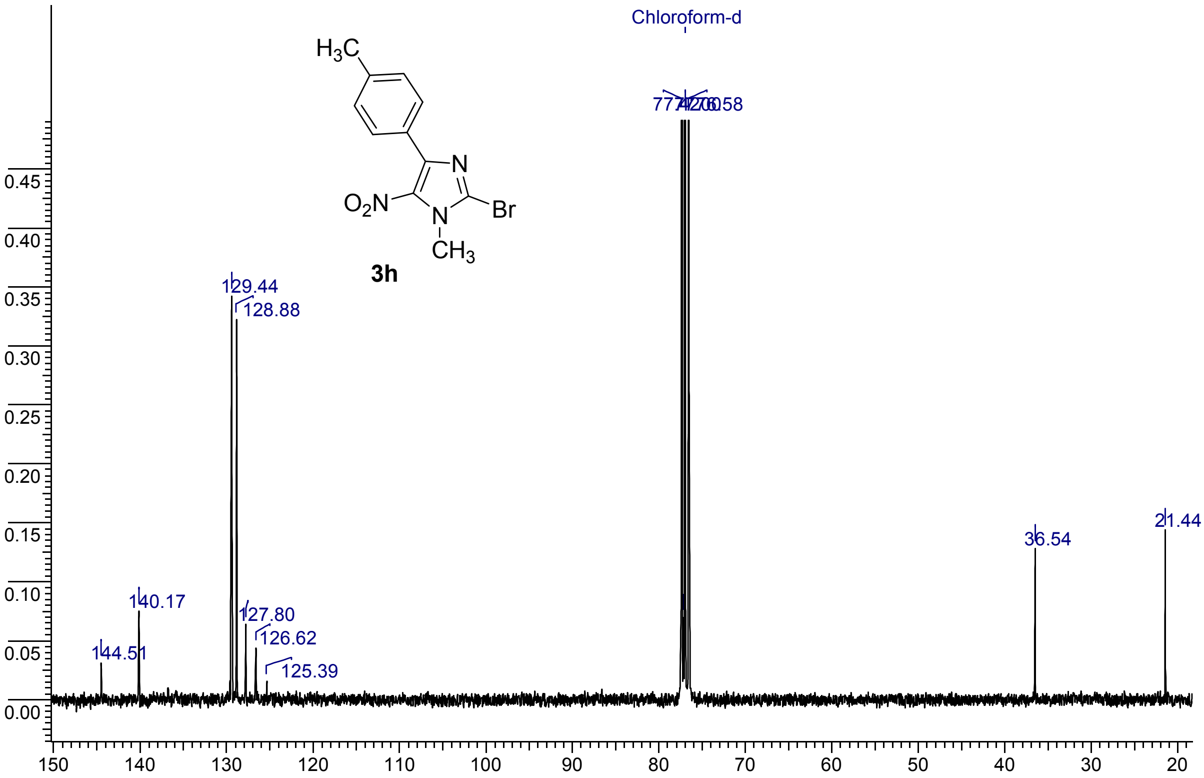

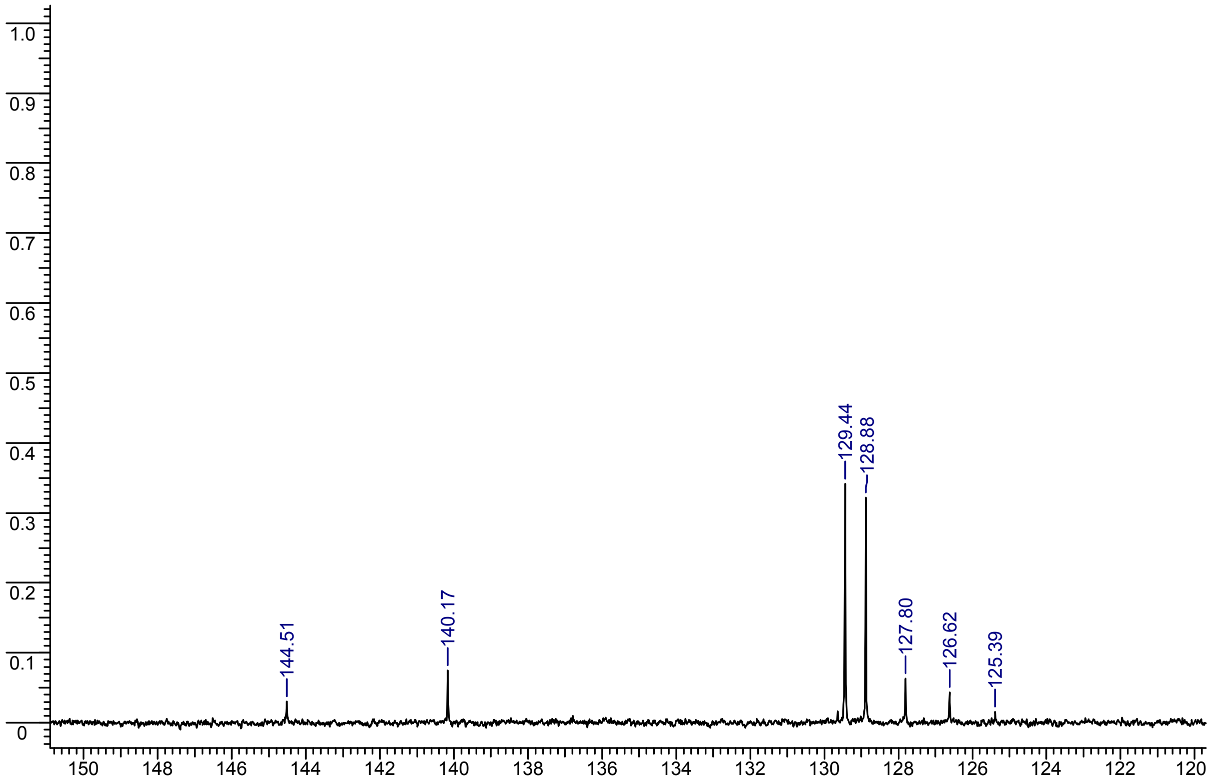

Figure S16. ^{13}C-NMR spectra of **3h**

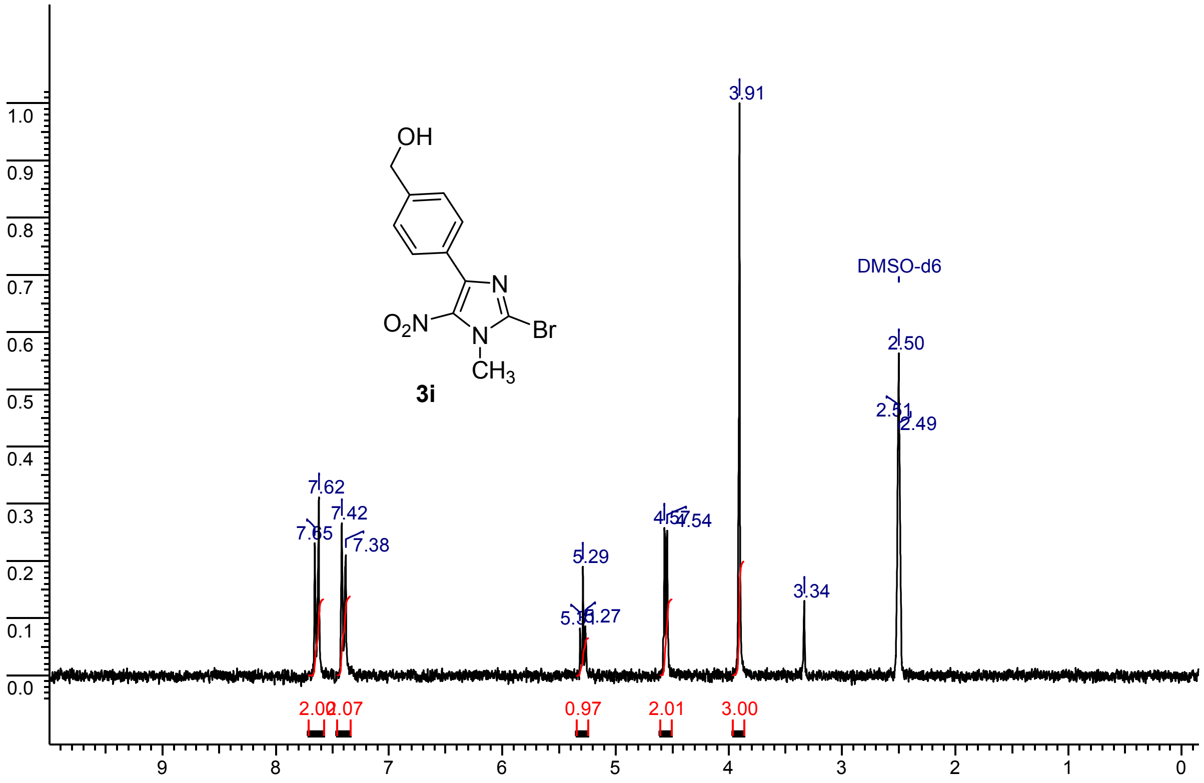

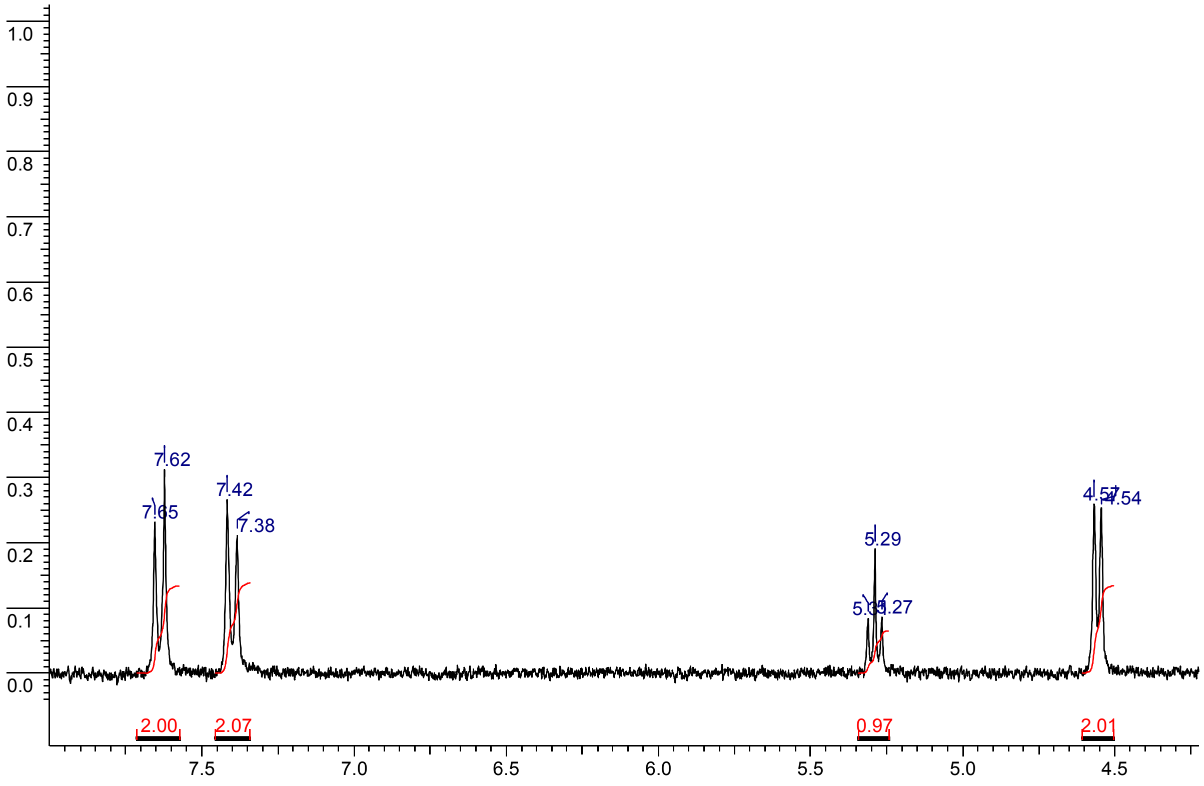

Figure S17. ^{1}H-NMR spectra of **3i**

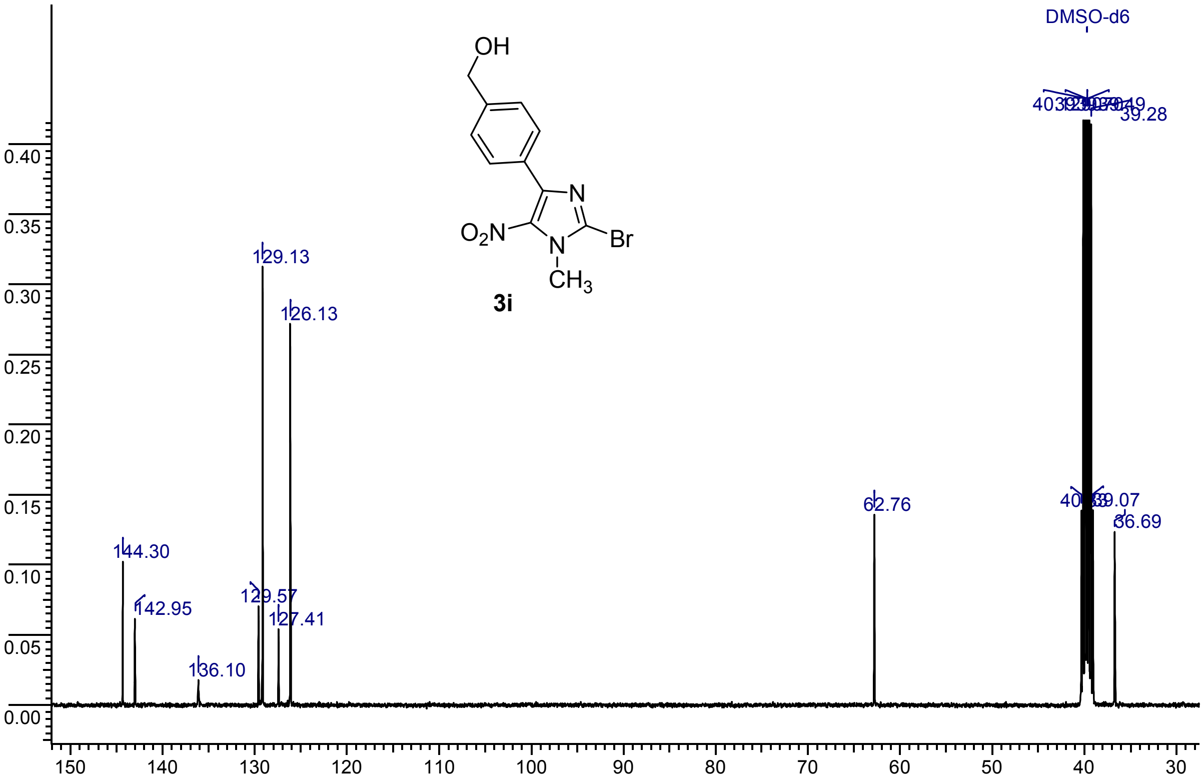

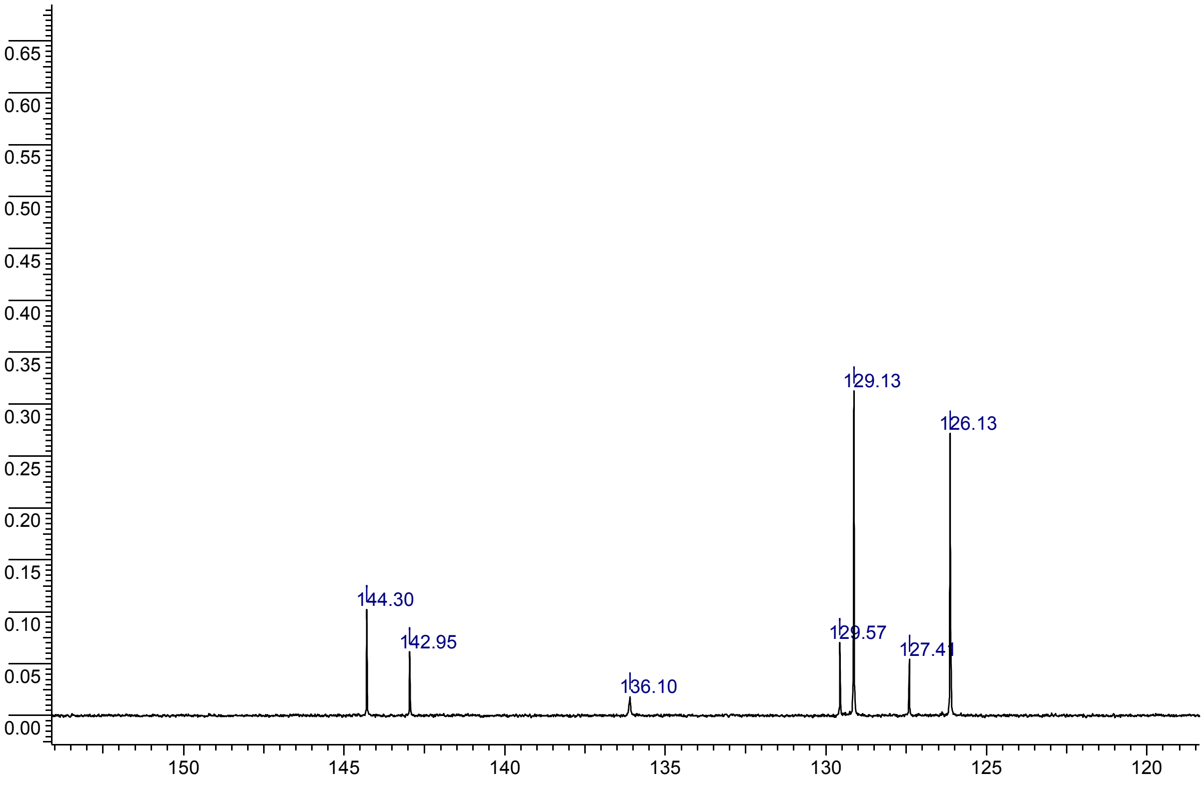

Figure S18. ^{13}C-NMR spectra of **3i**

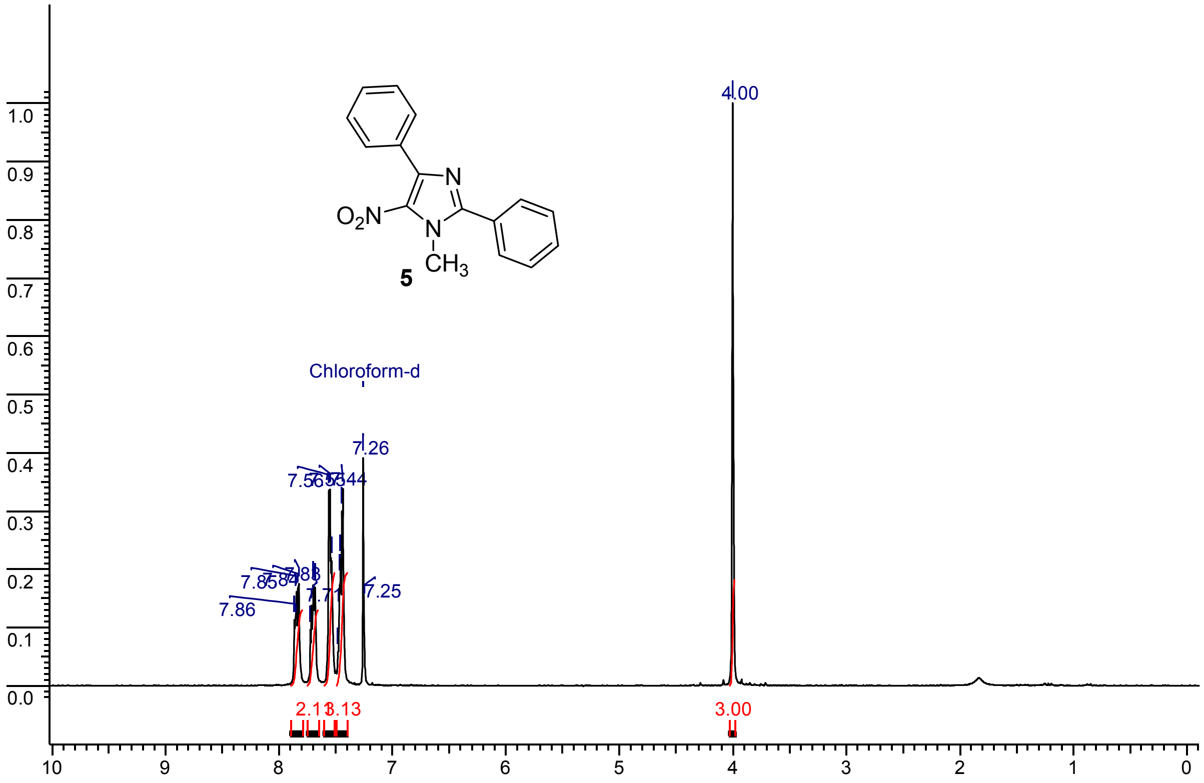

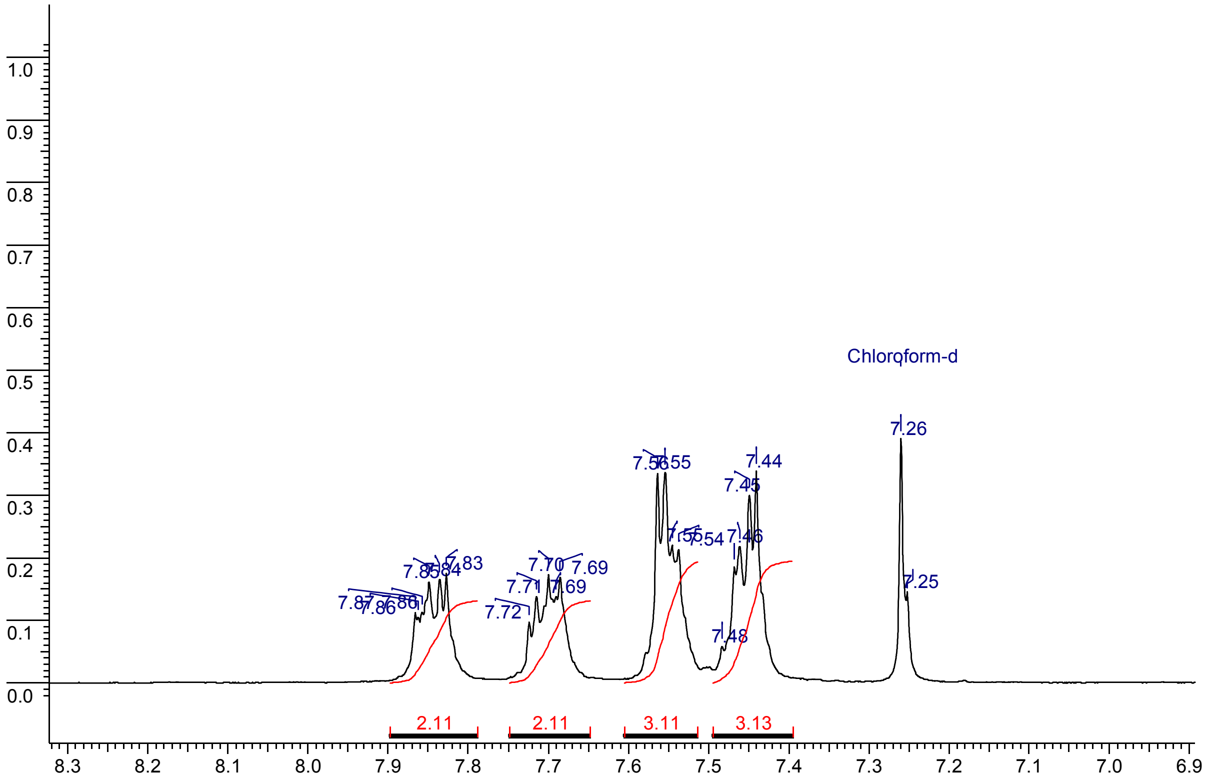

Figure S19. ^{1}H-NMR spectra of **5**

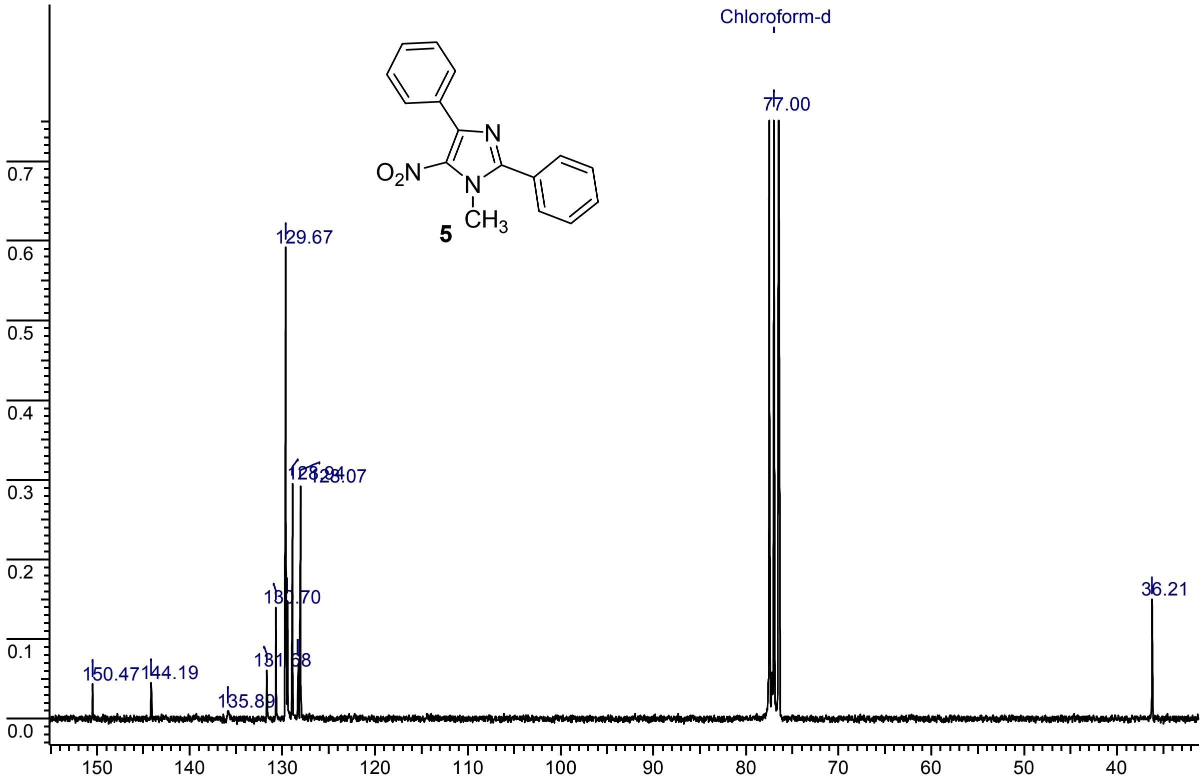

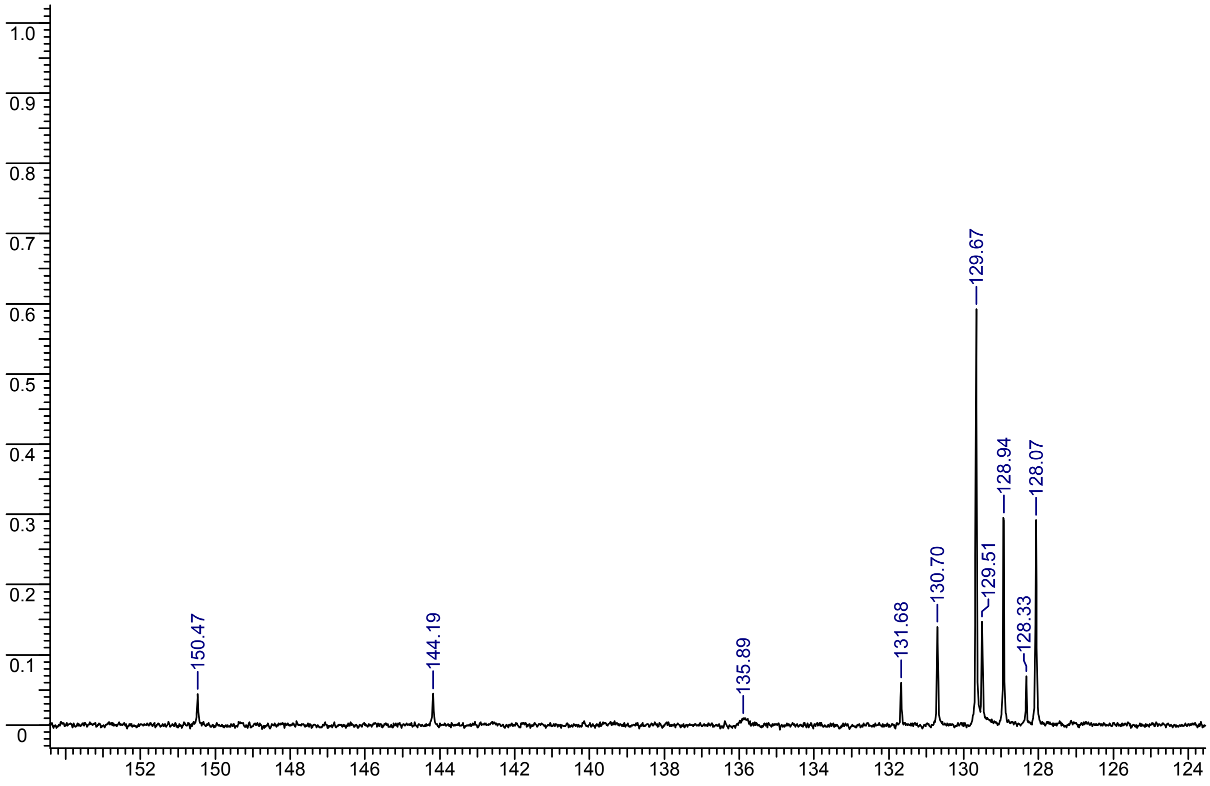

Figure S20. ^{13}C-NMR spectra of **5**

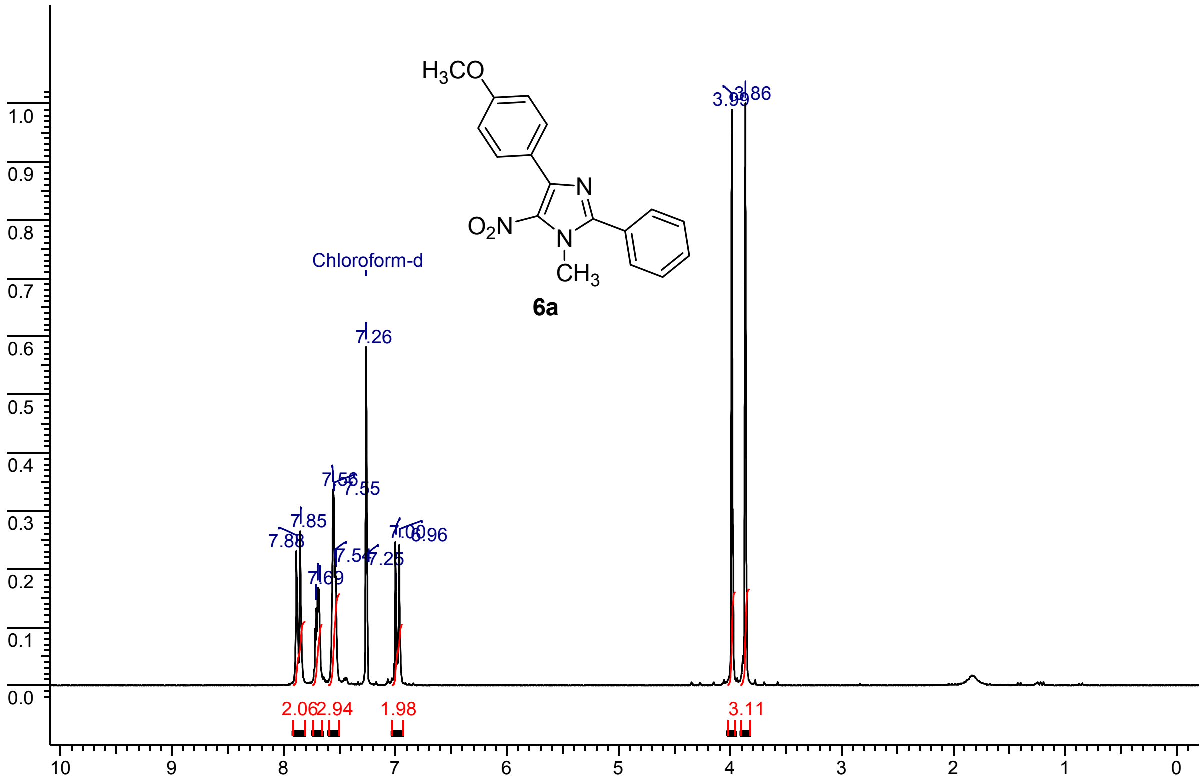

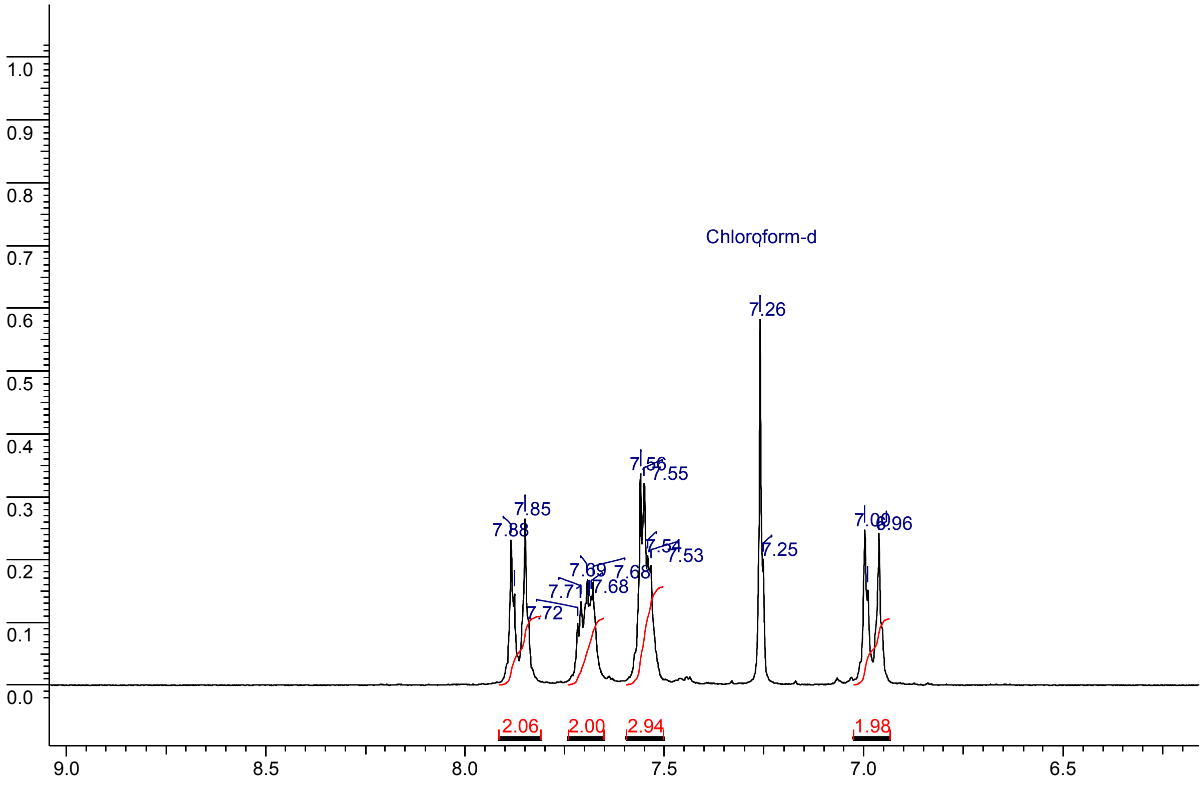

Figure S21. ^{1}H-NMR spectra of **6a**

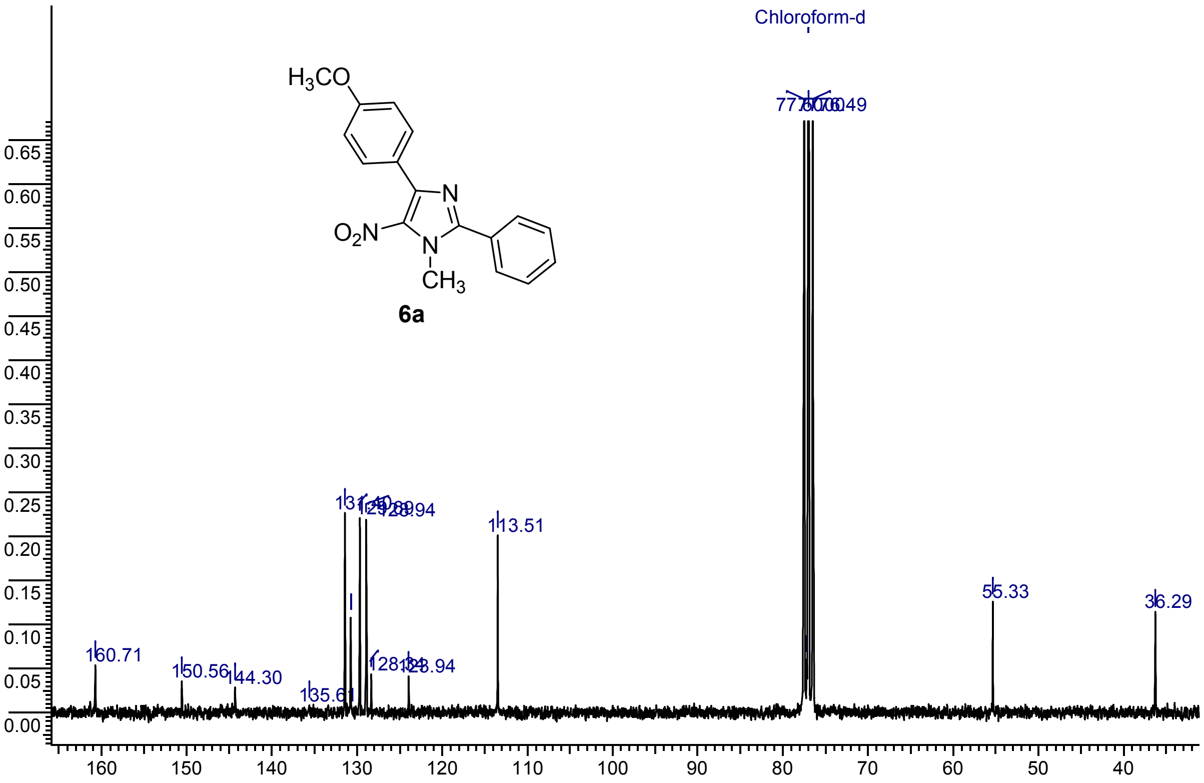

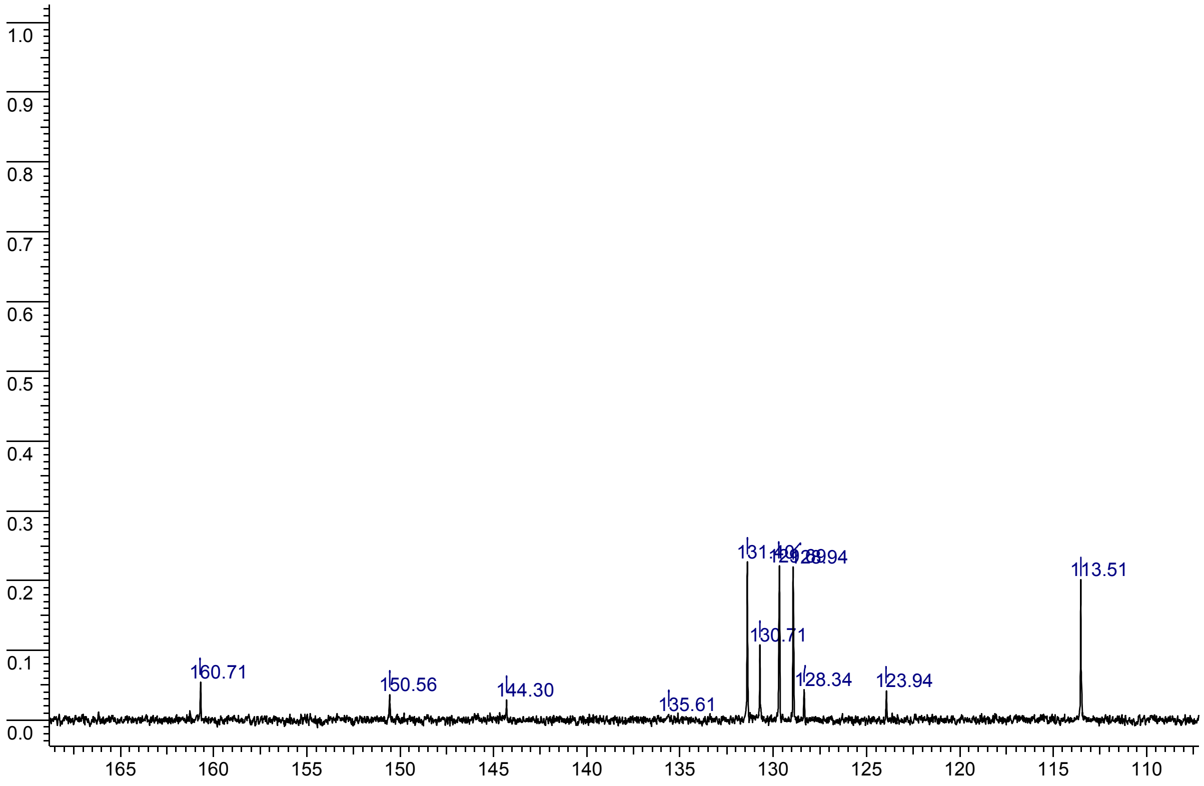

Figure S22. ^{13}C-NMR spectra of **6a**

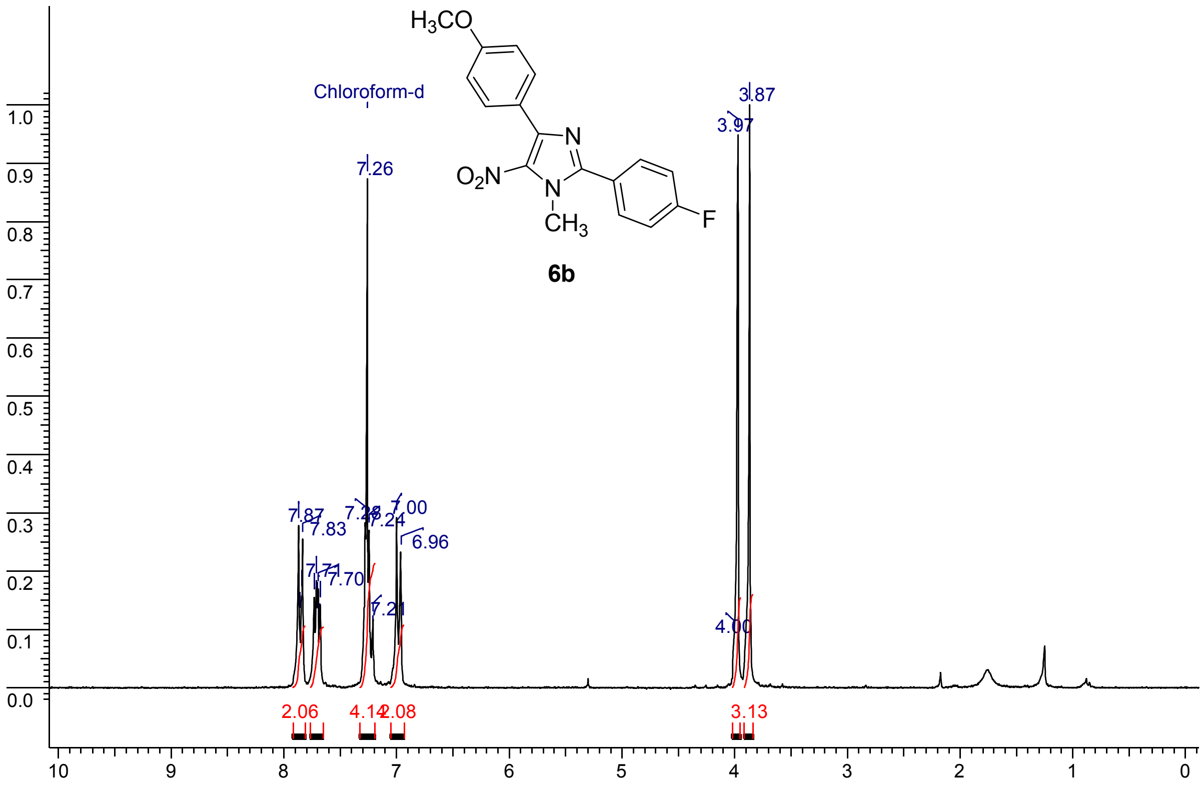

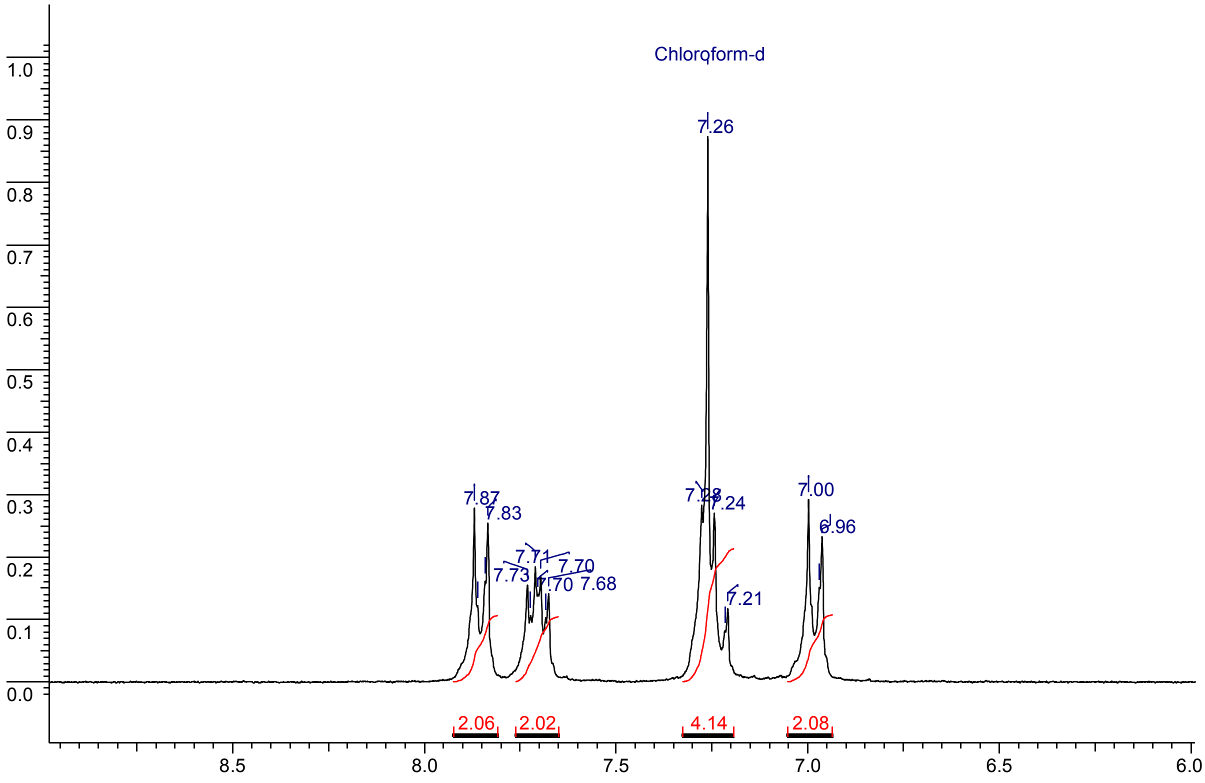

Figure S23. ^{1}H-NMR spectra of **6b**

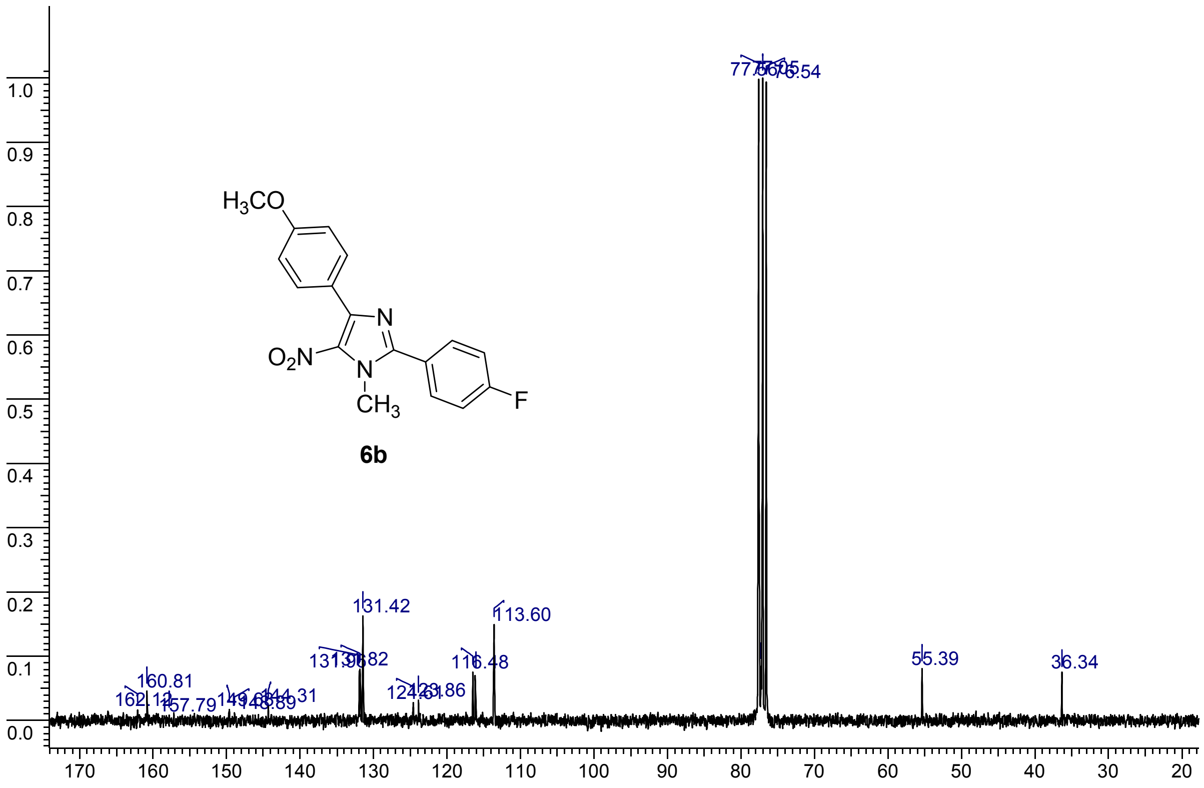

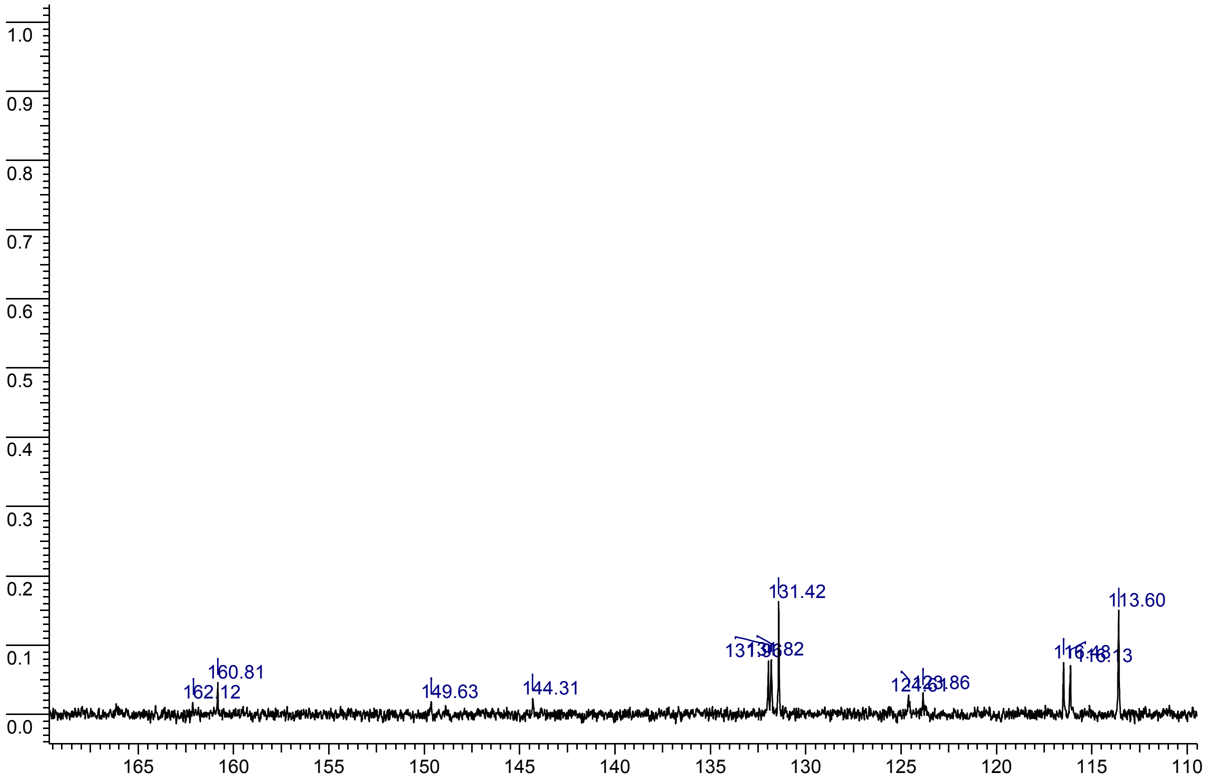

Figure S24. ^{13}C-NMR spectra of **6b**

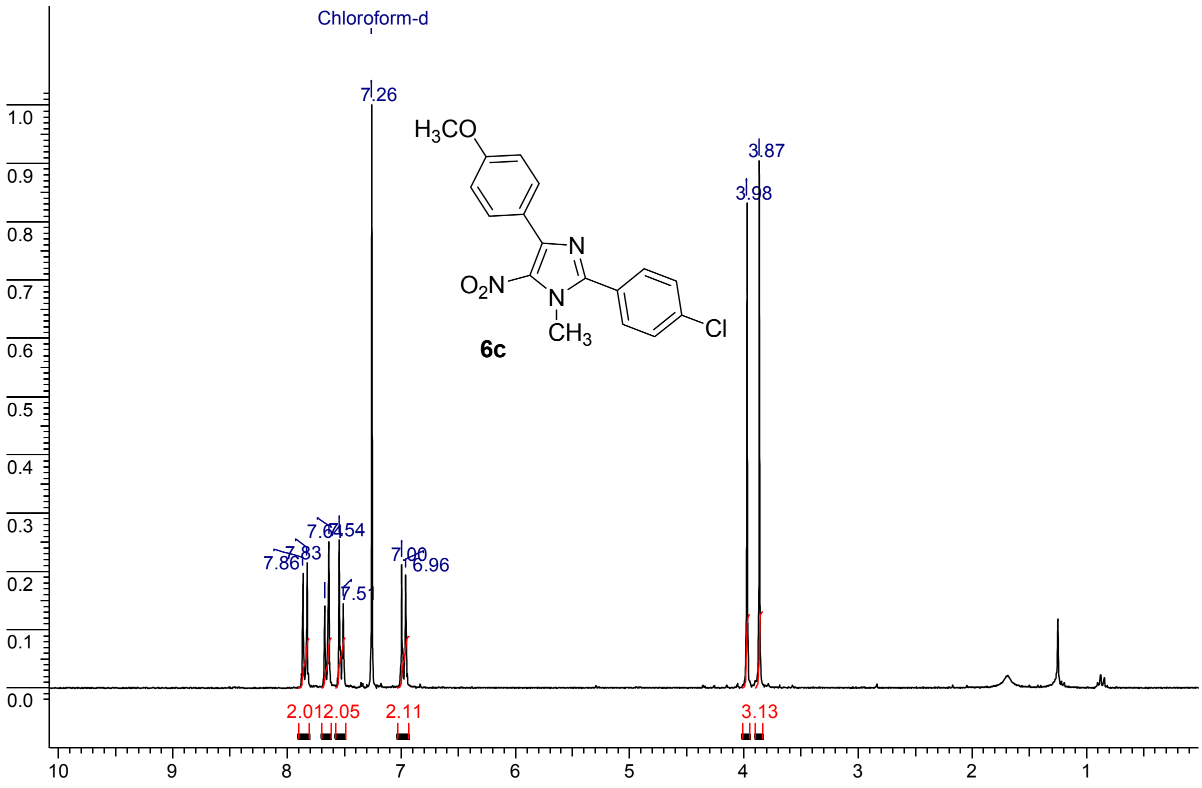

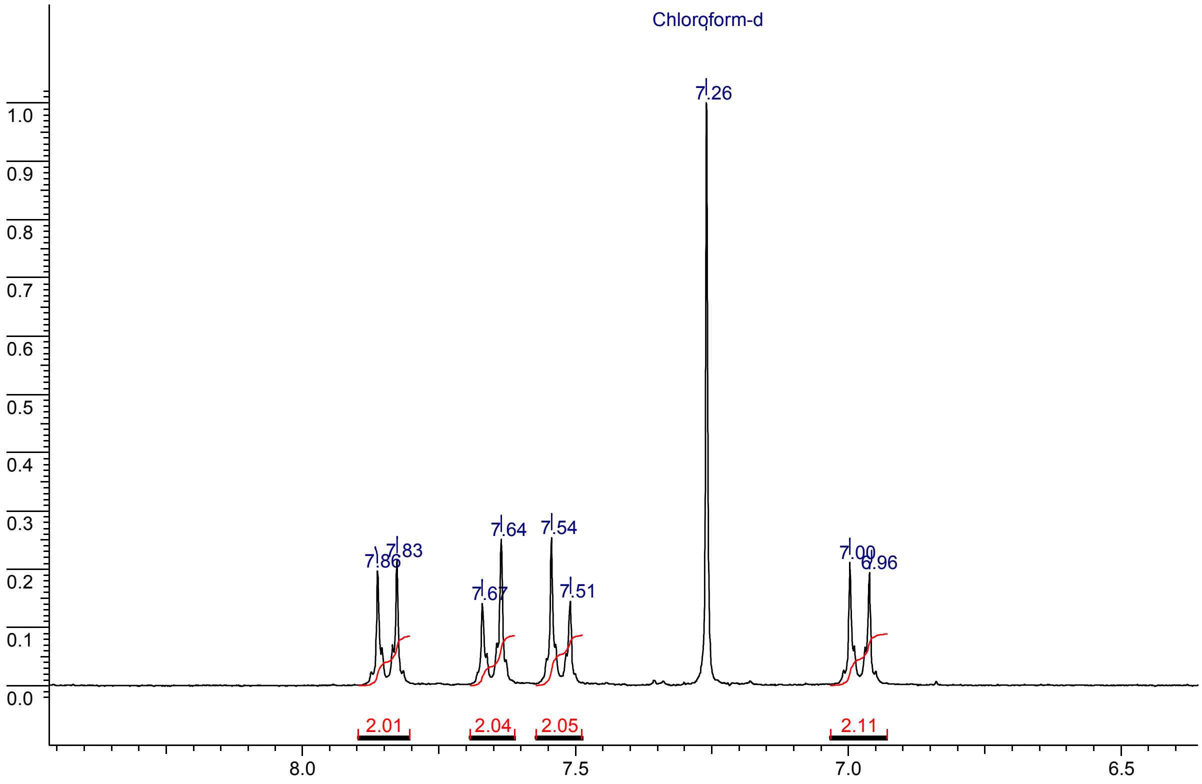

Figure S25. ^{1}H-NMR spectra of **6c**

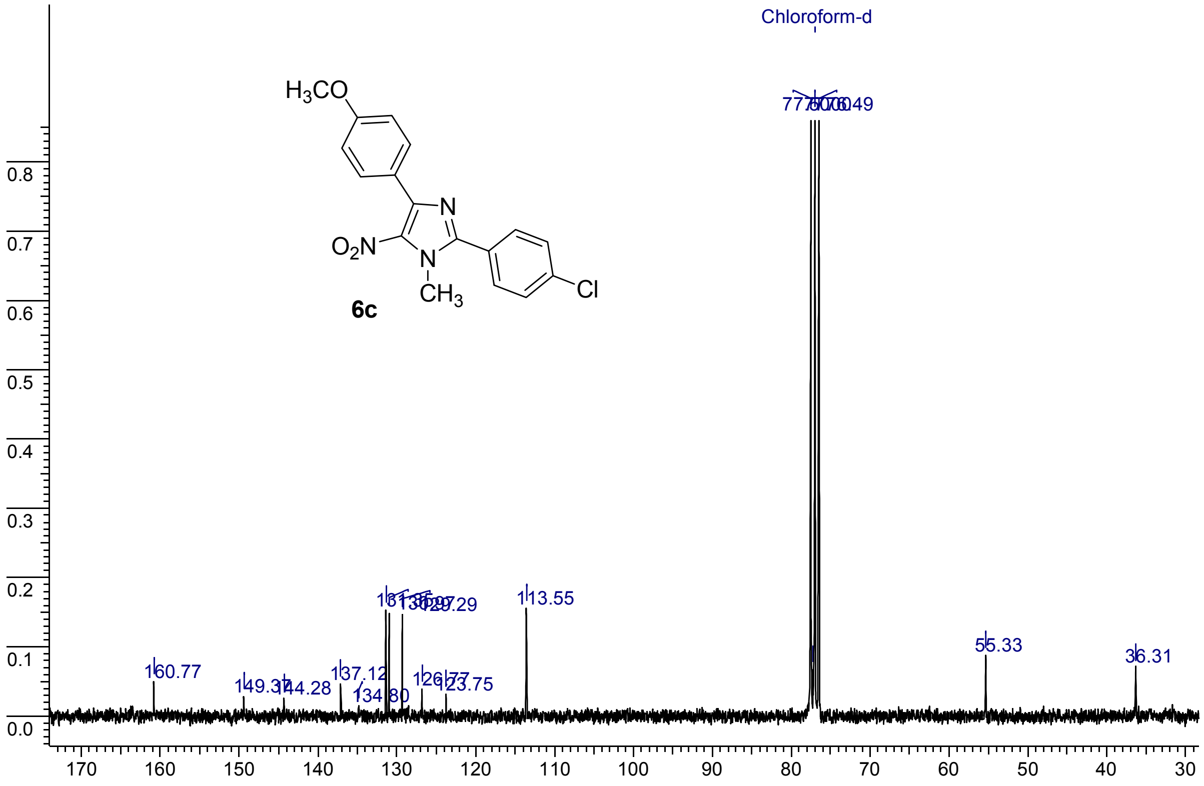

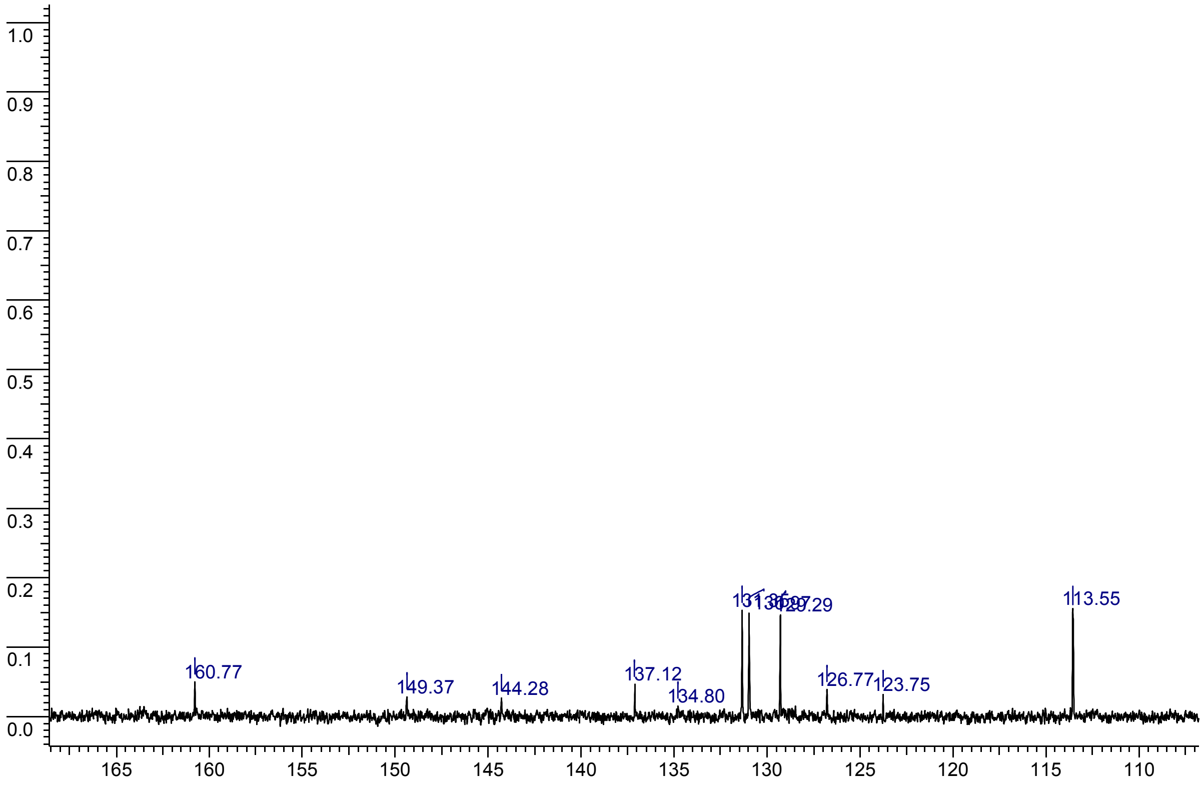

Figure S26. ^{13}C-NMR spectra of **6c**

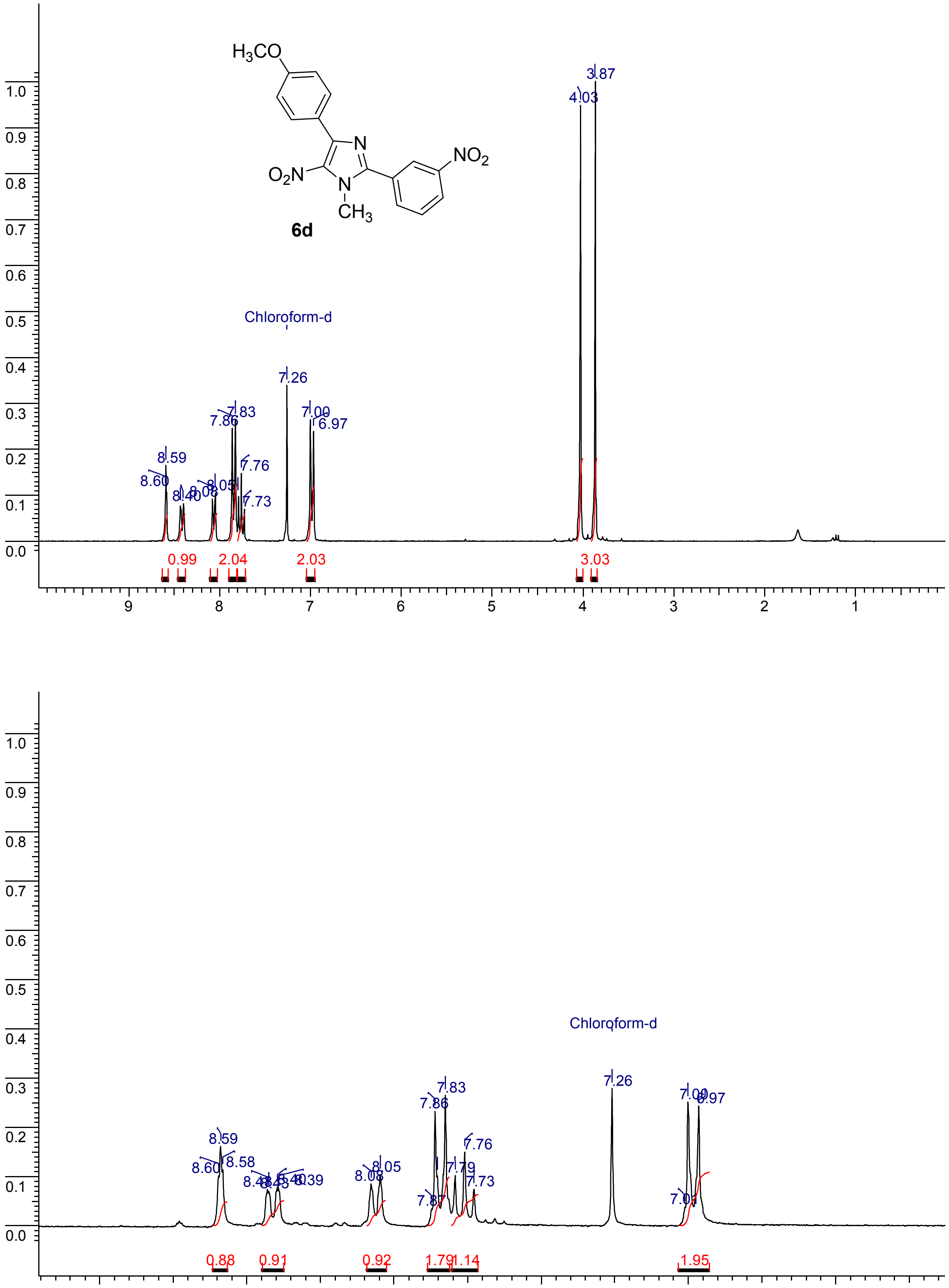

Figure S27. ^{1}H-NMR spectra of **6d**

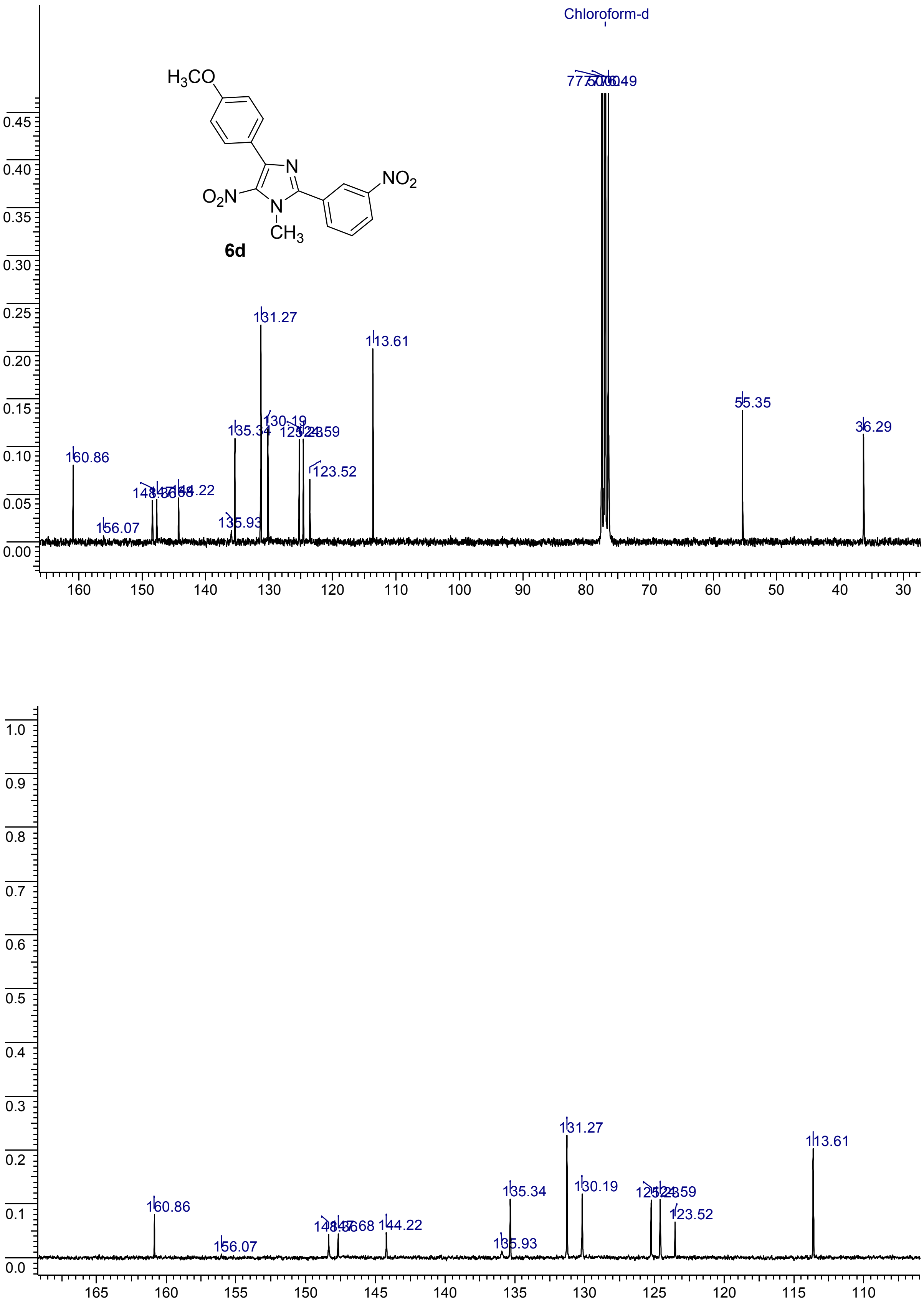

Figure S28. ^{13}C-NMR spectra of **6d**

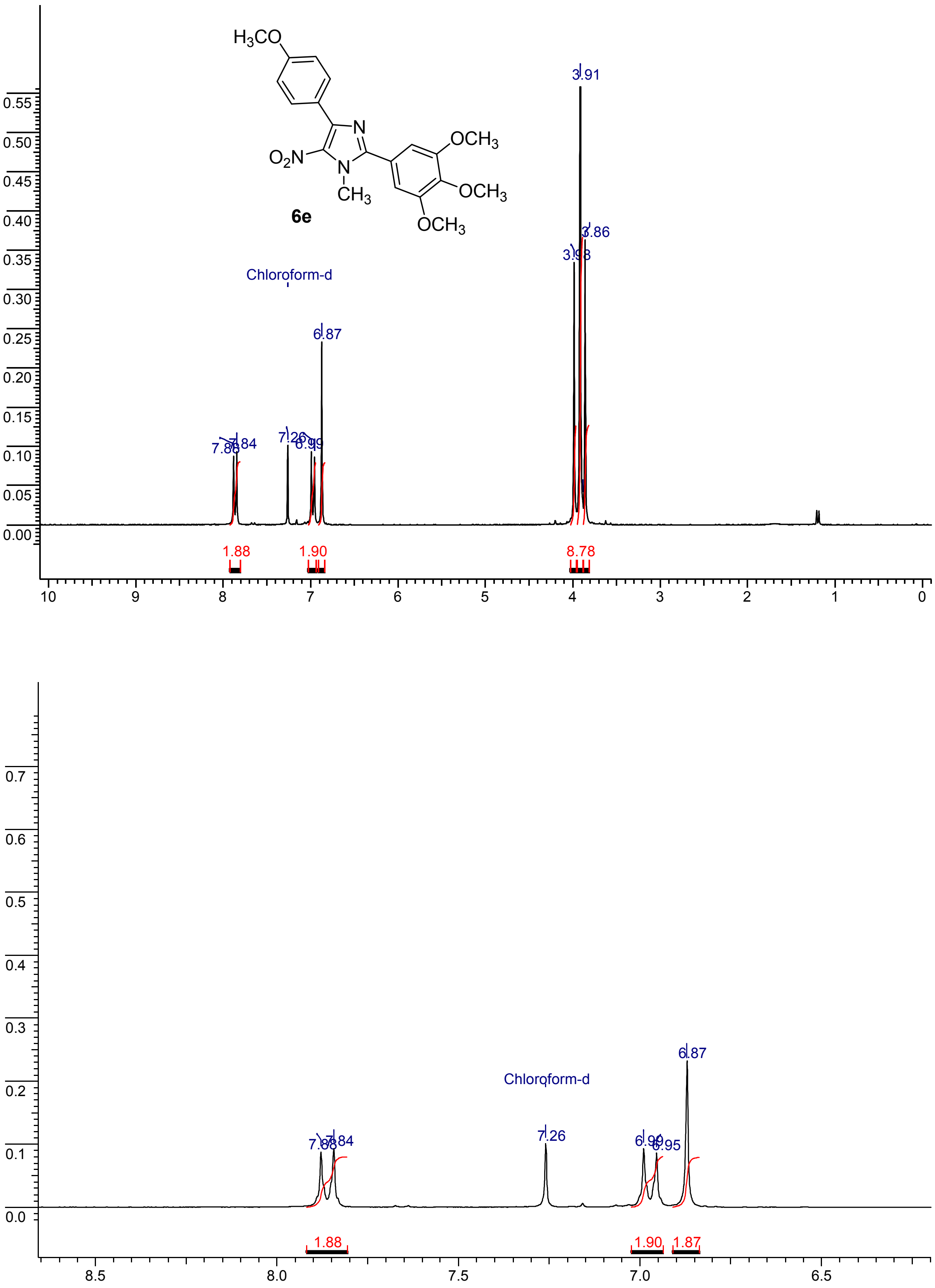

Figure S29. ^{1}H-NMR spectra of **6e**

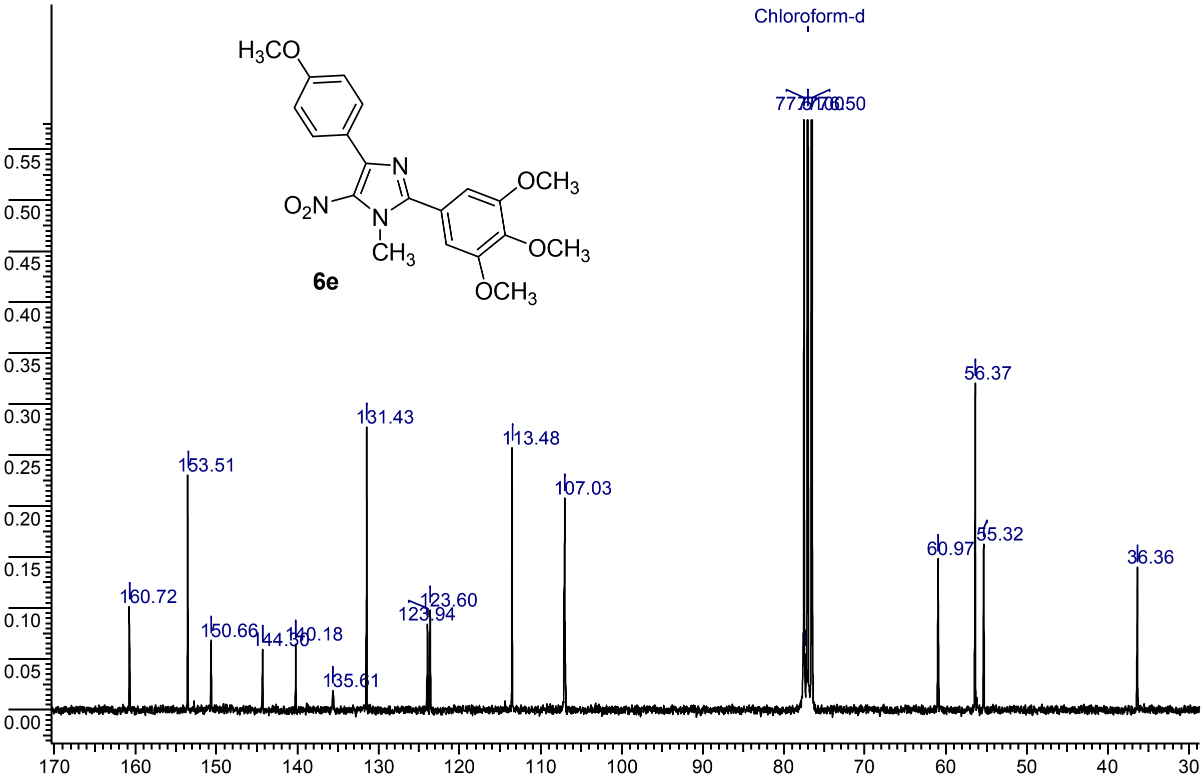

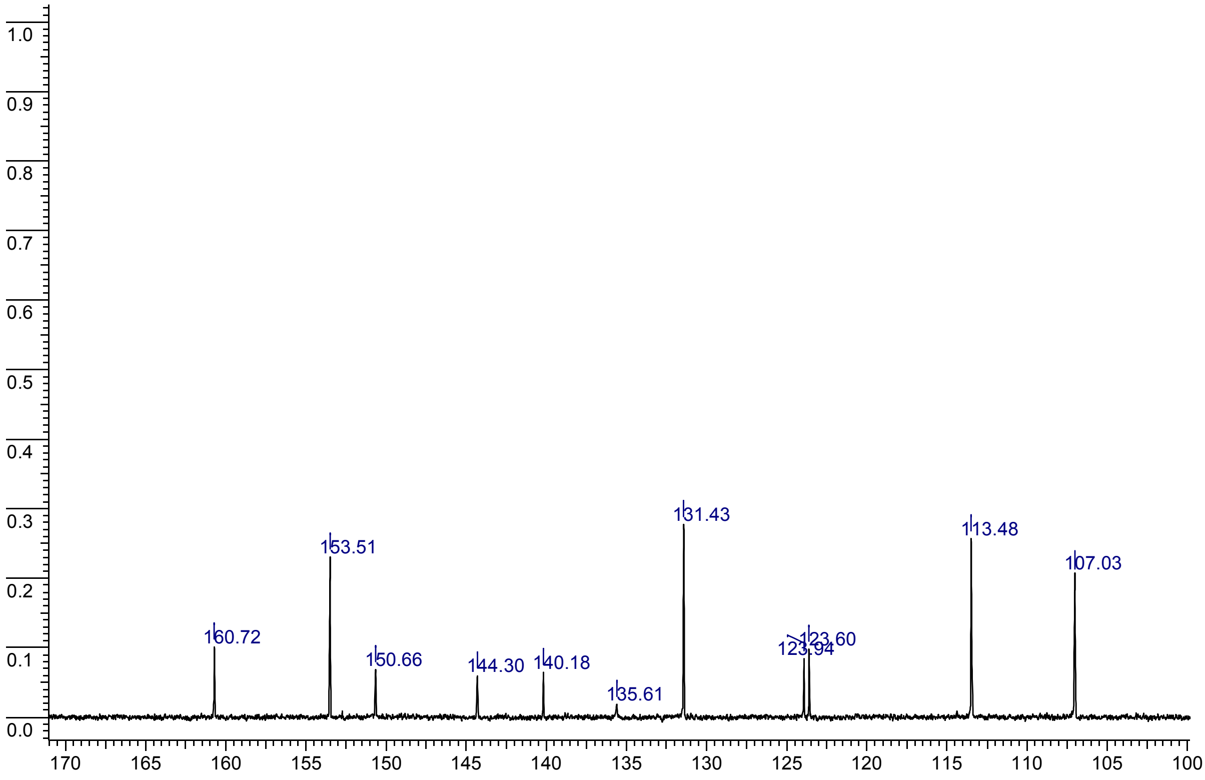

Figure S30. ^{13}C-NMR spectra of **6e**

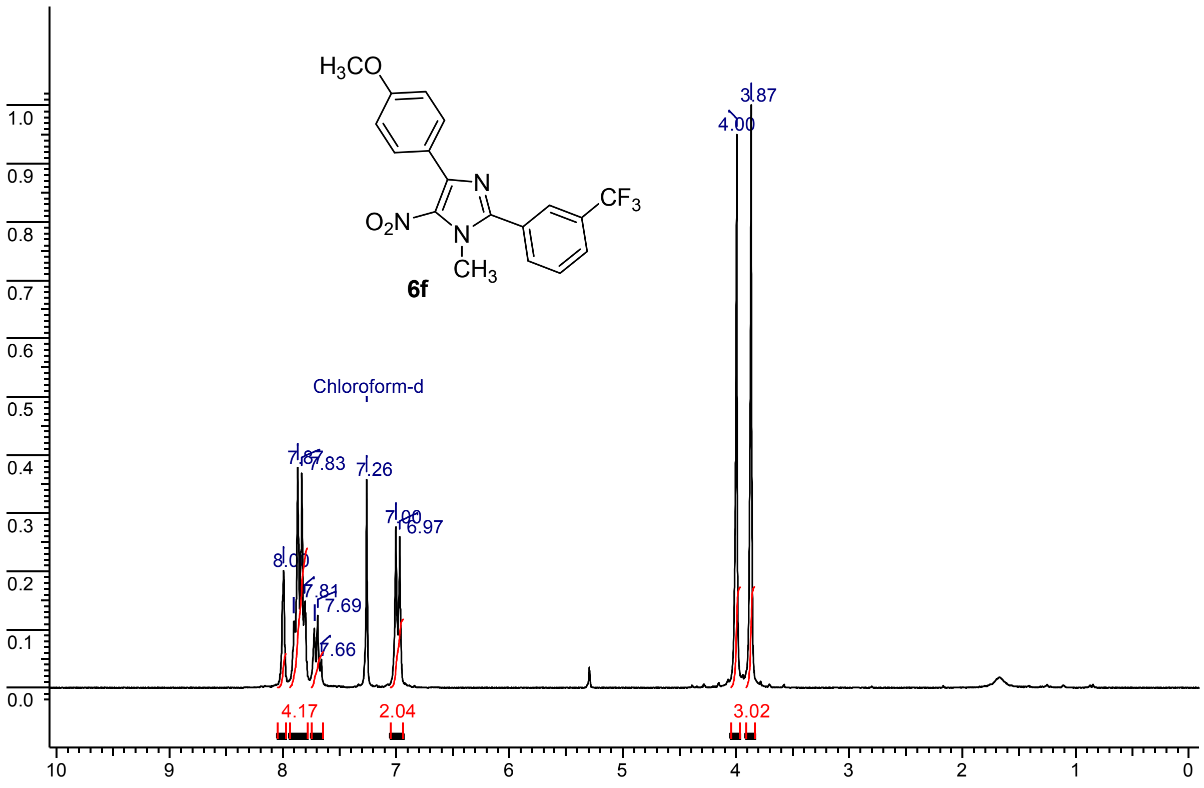

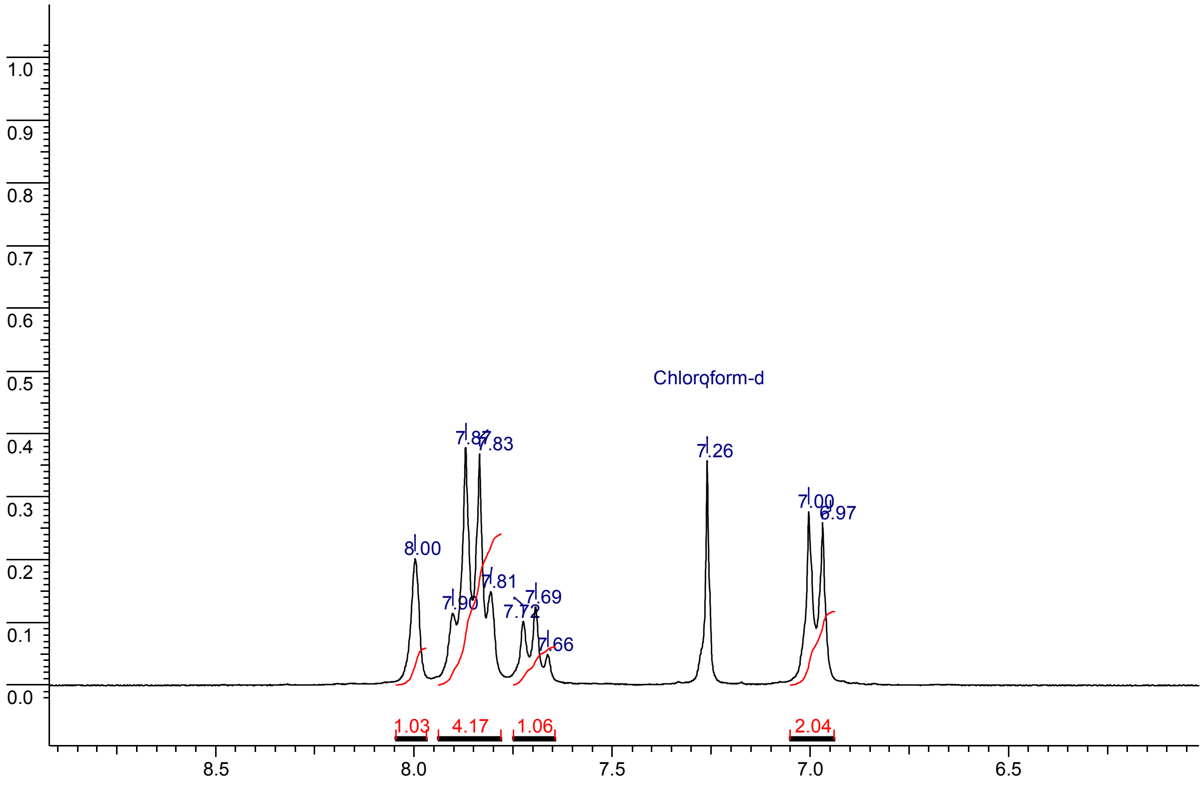

Figure S31. ^{1}H-NMR spectra of **6f**

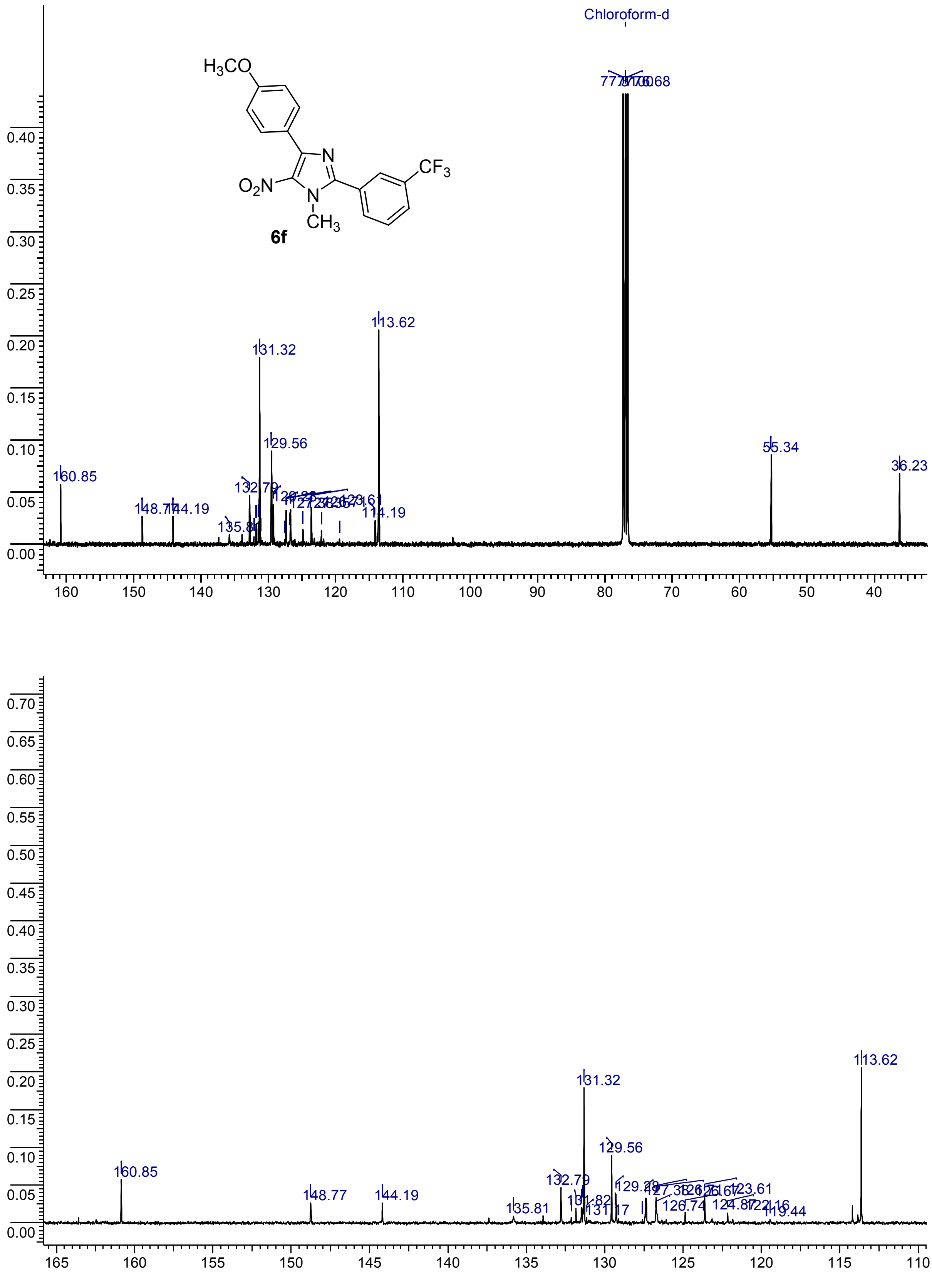

Figure S32. ^{13}C-NMR spectra of **6f**

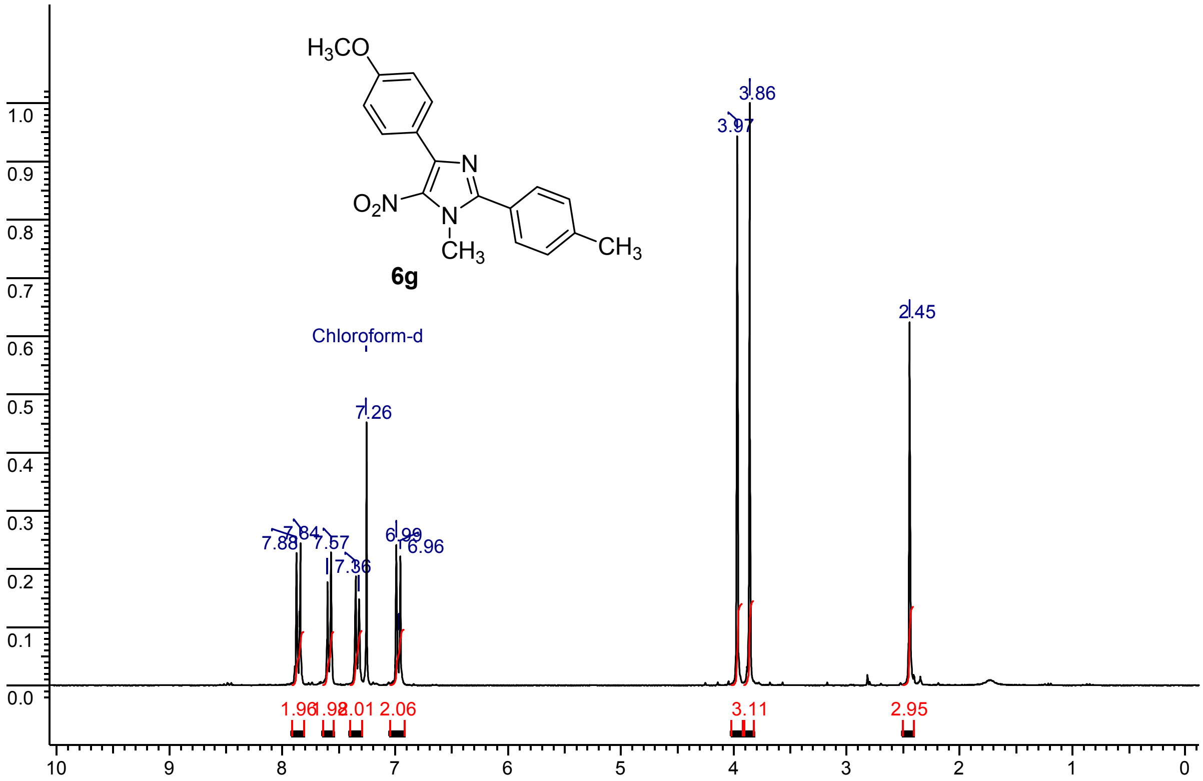

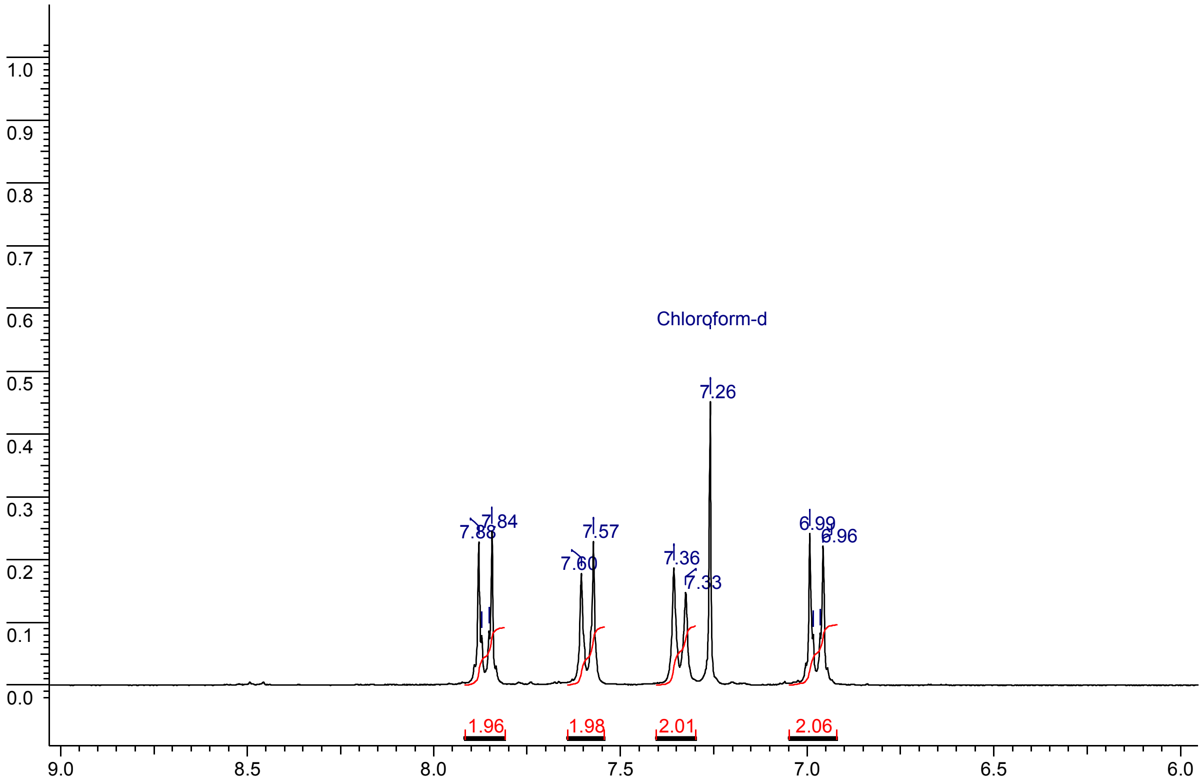

Figure S33. ^{1}H-NMR spectra of **6g**

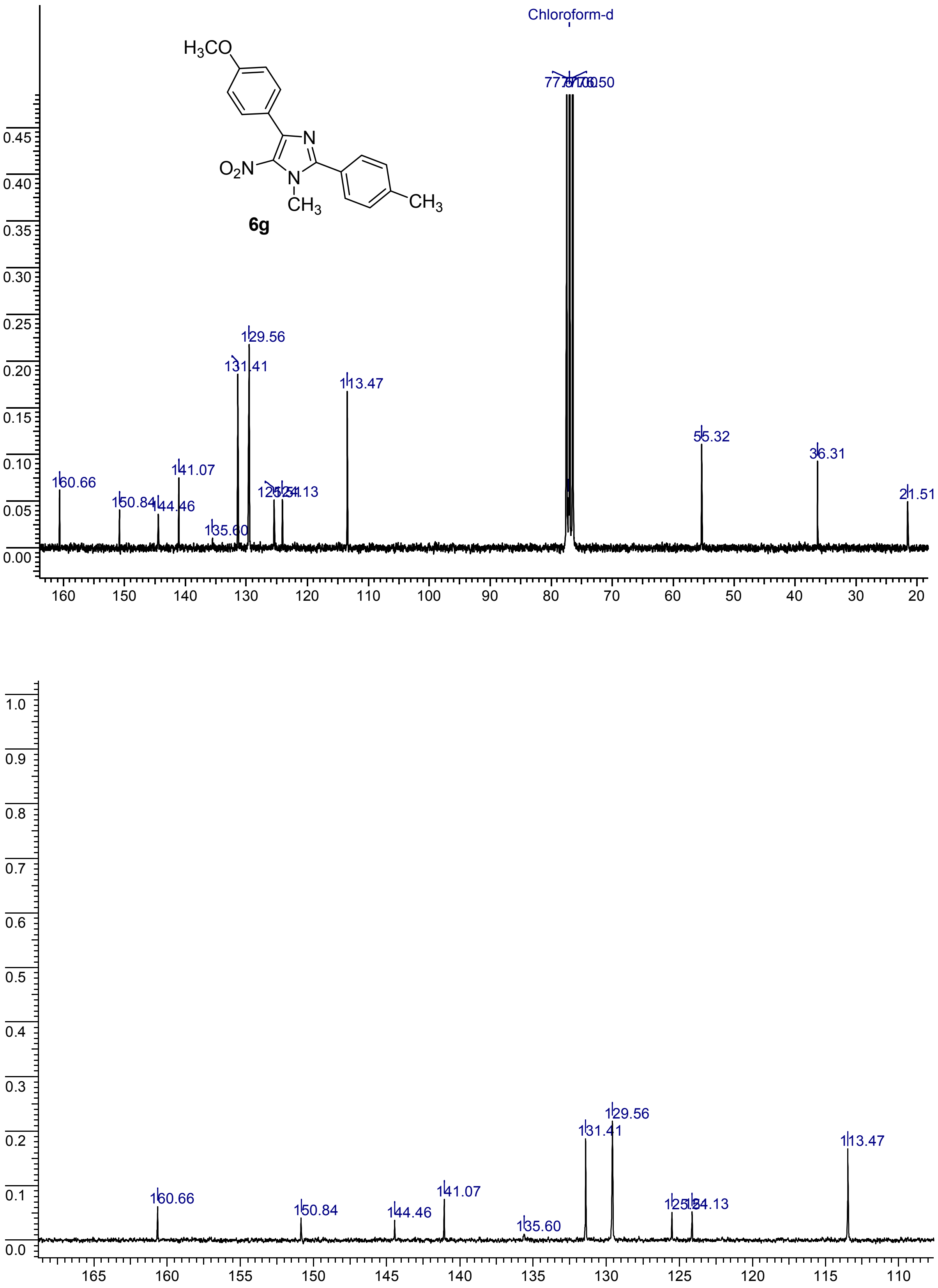

Figure S34. ^{13}C-NMR spectra of **6g**

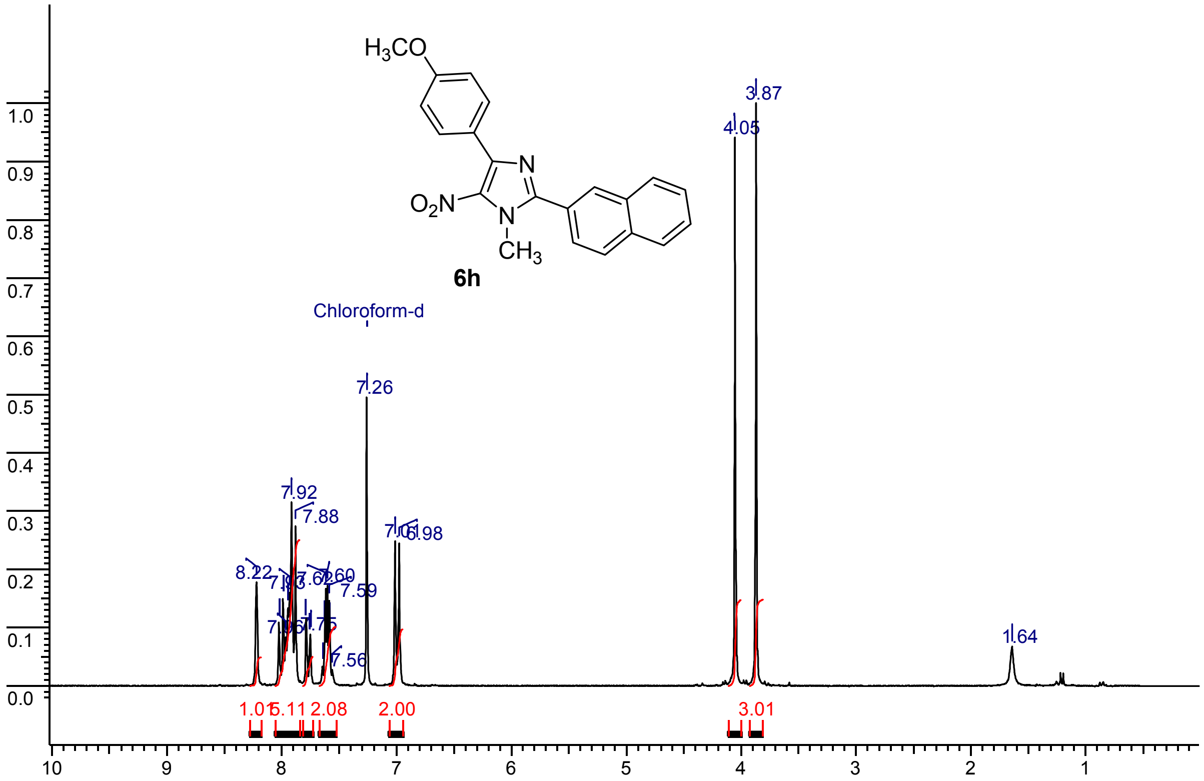

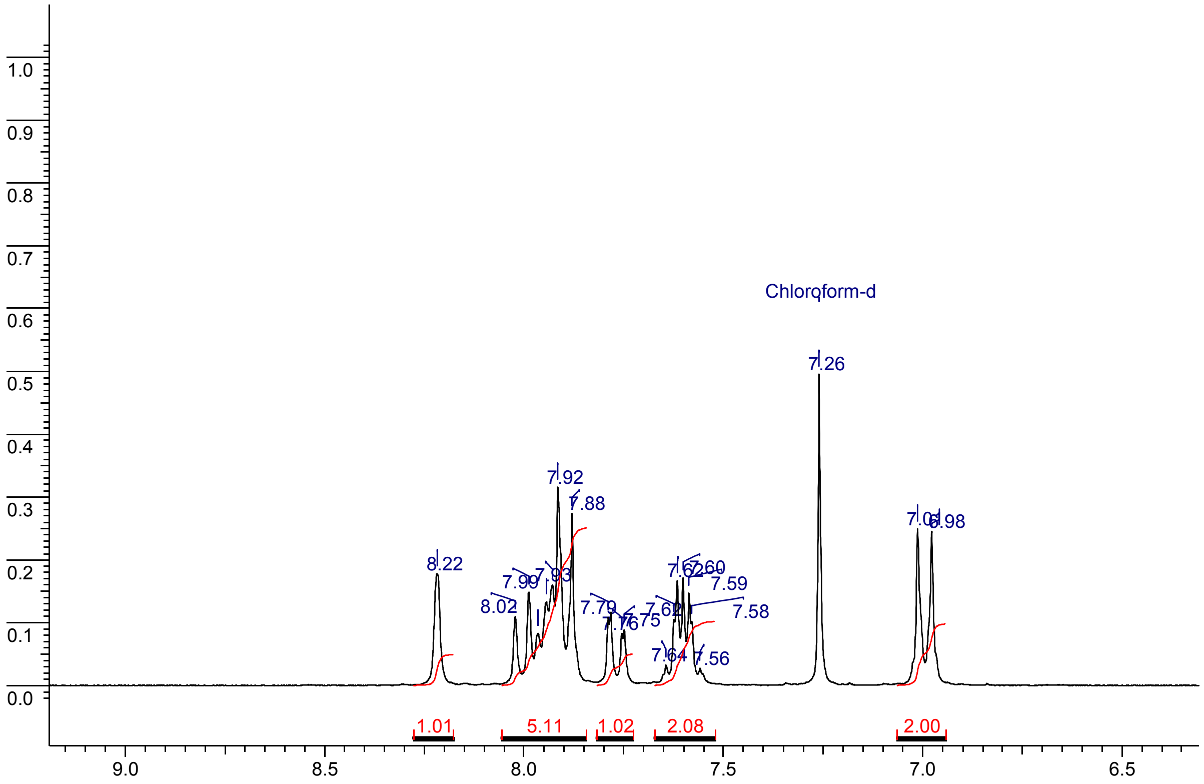

Figure S35. ^{1}H-NMR spectra of **6h**

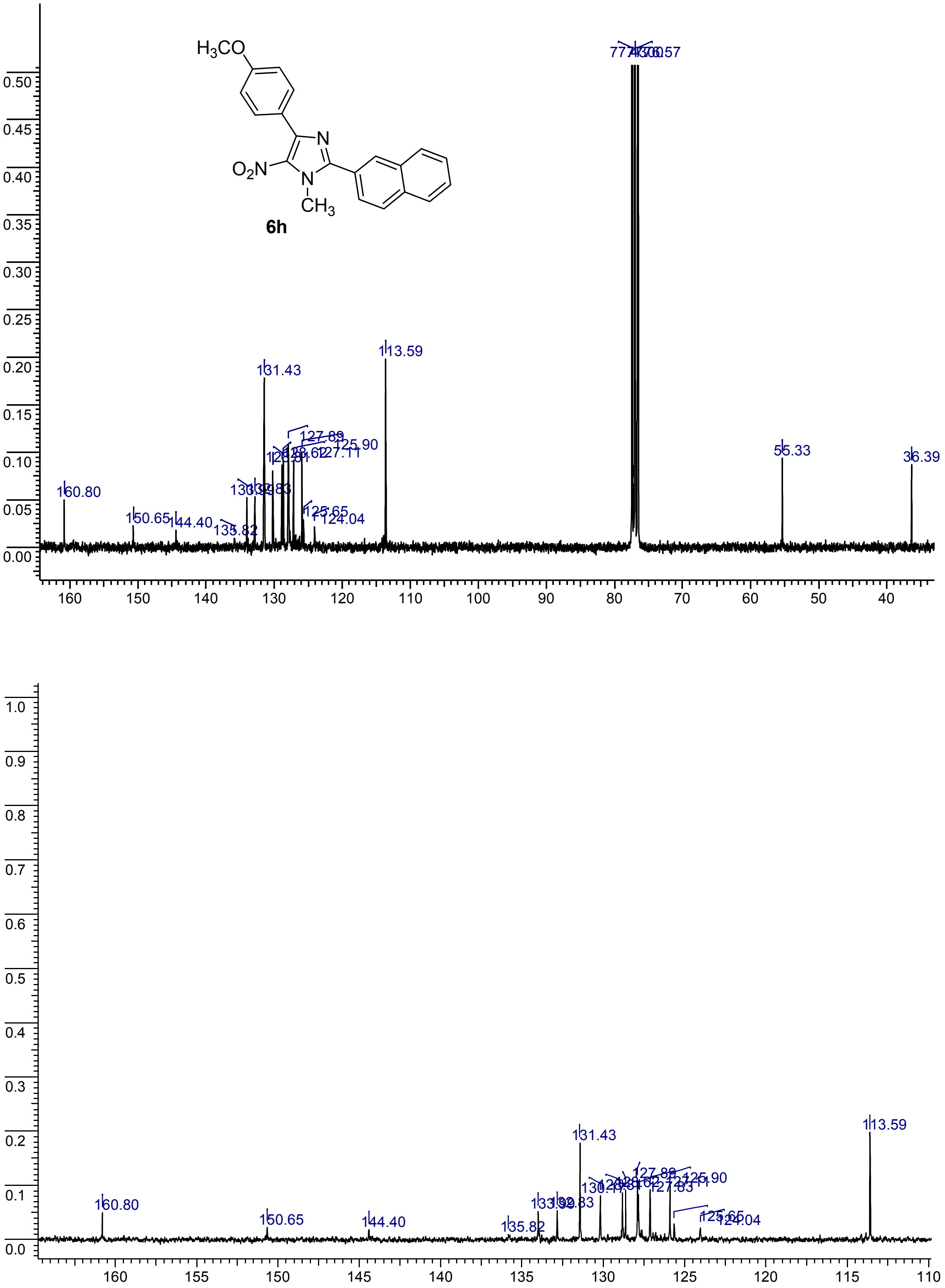

Figure S36. ^{13}C-NMR spectra of **6h**

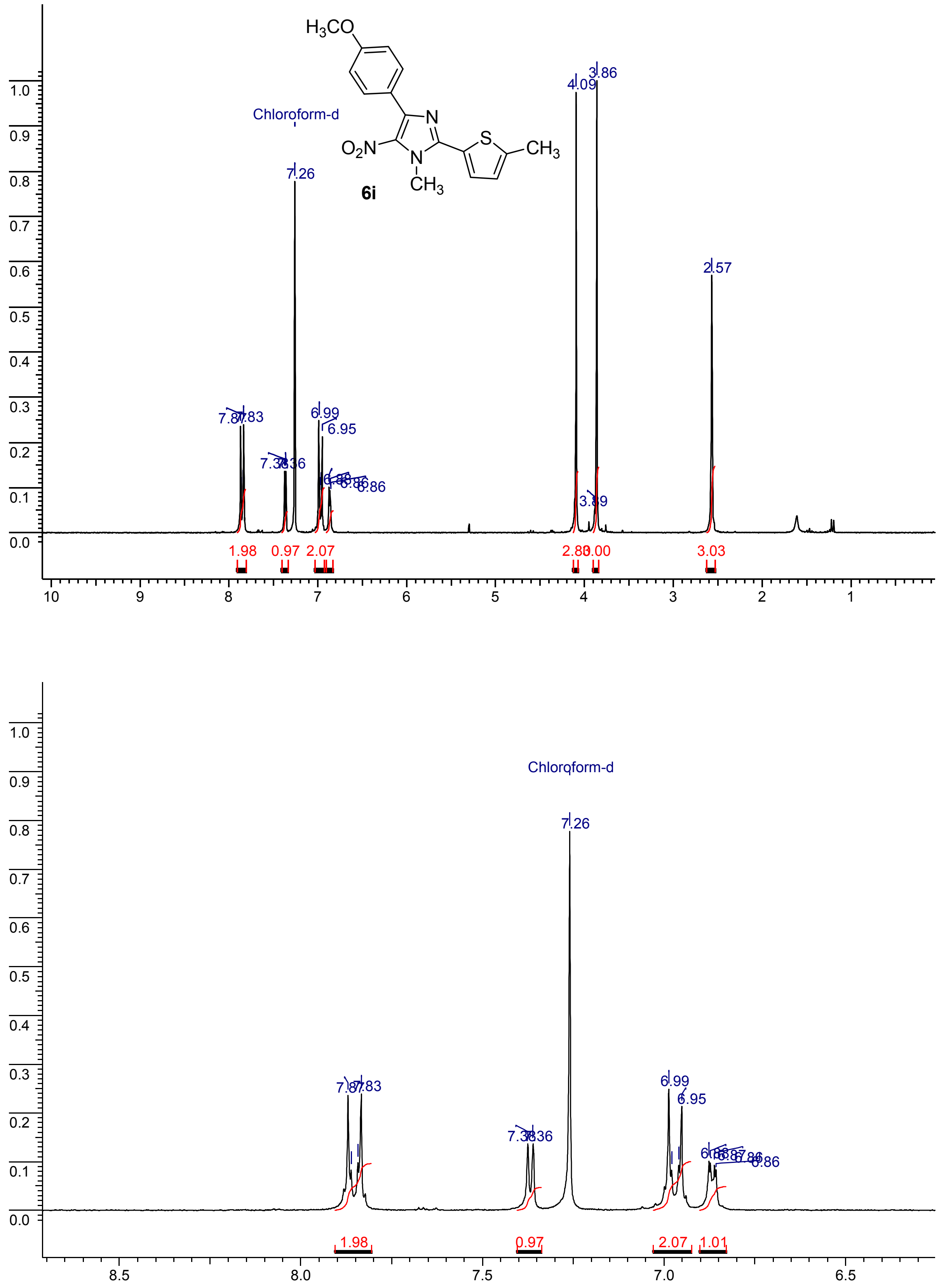

Figure S37. ^{1}H-NMR spectra of **6i**

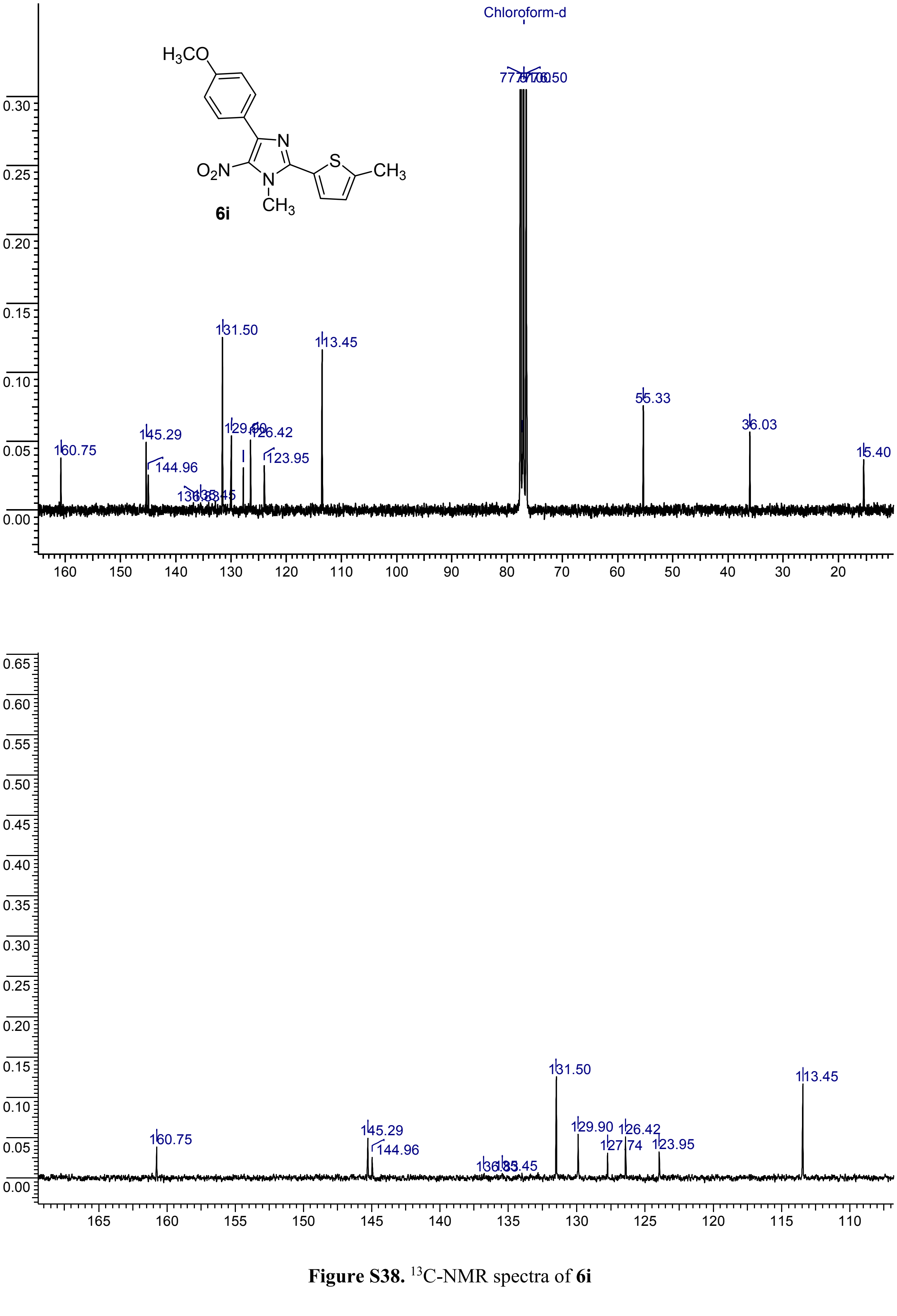

Figure S38. ^{13}C-NMR spectra of **6i**

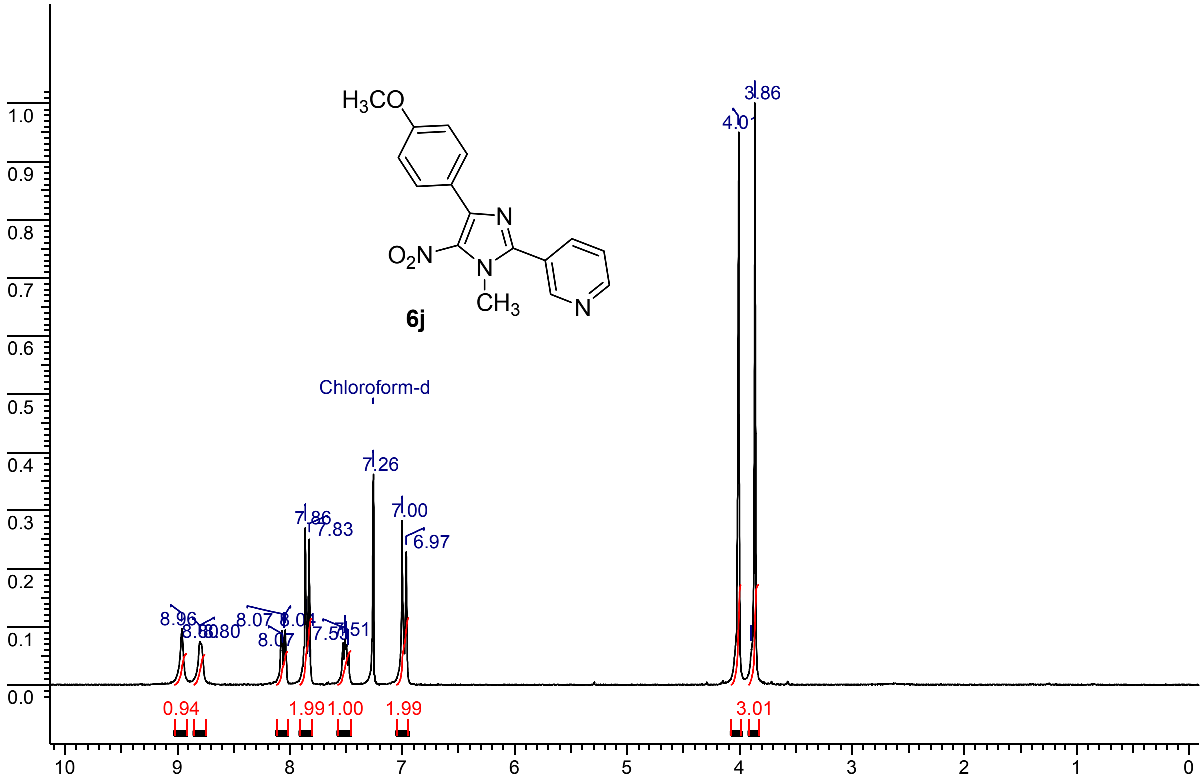

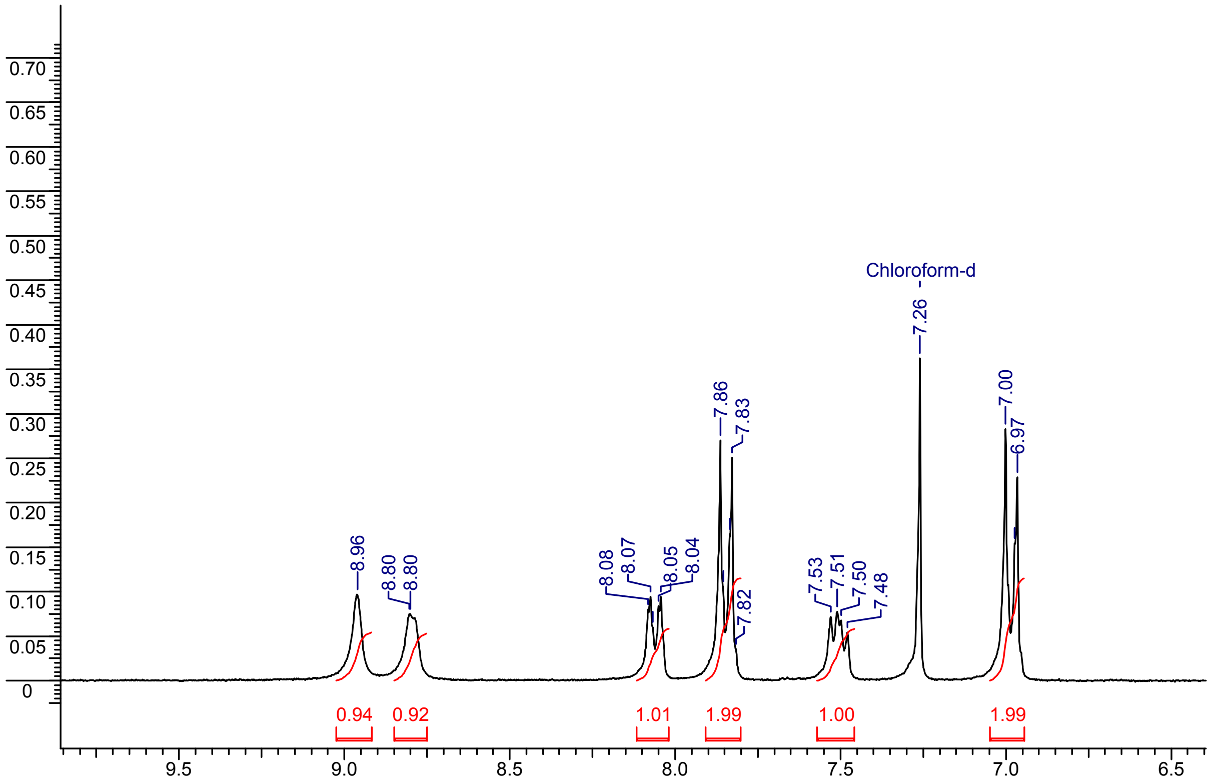

Figure S39. ^{1}H-NMR spectra of **6j**

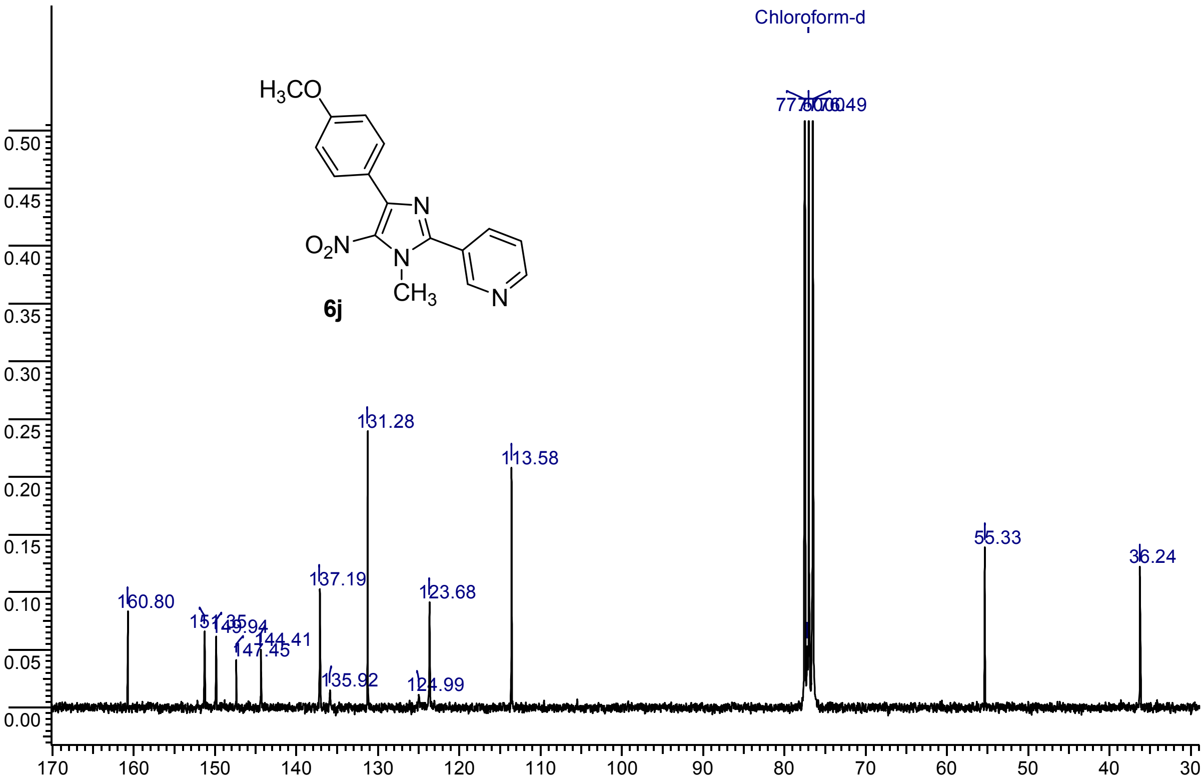

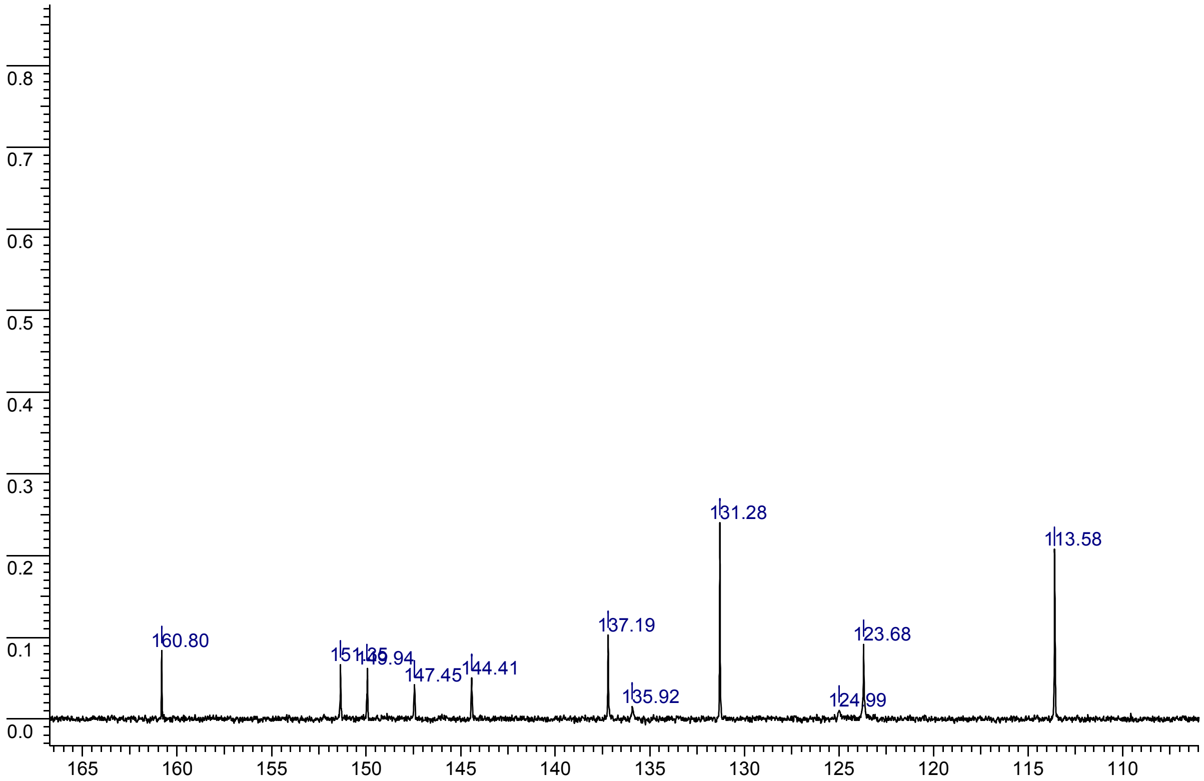

Figure S40. ^{13}C-NMR spectra of **6j**

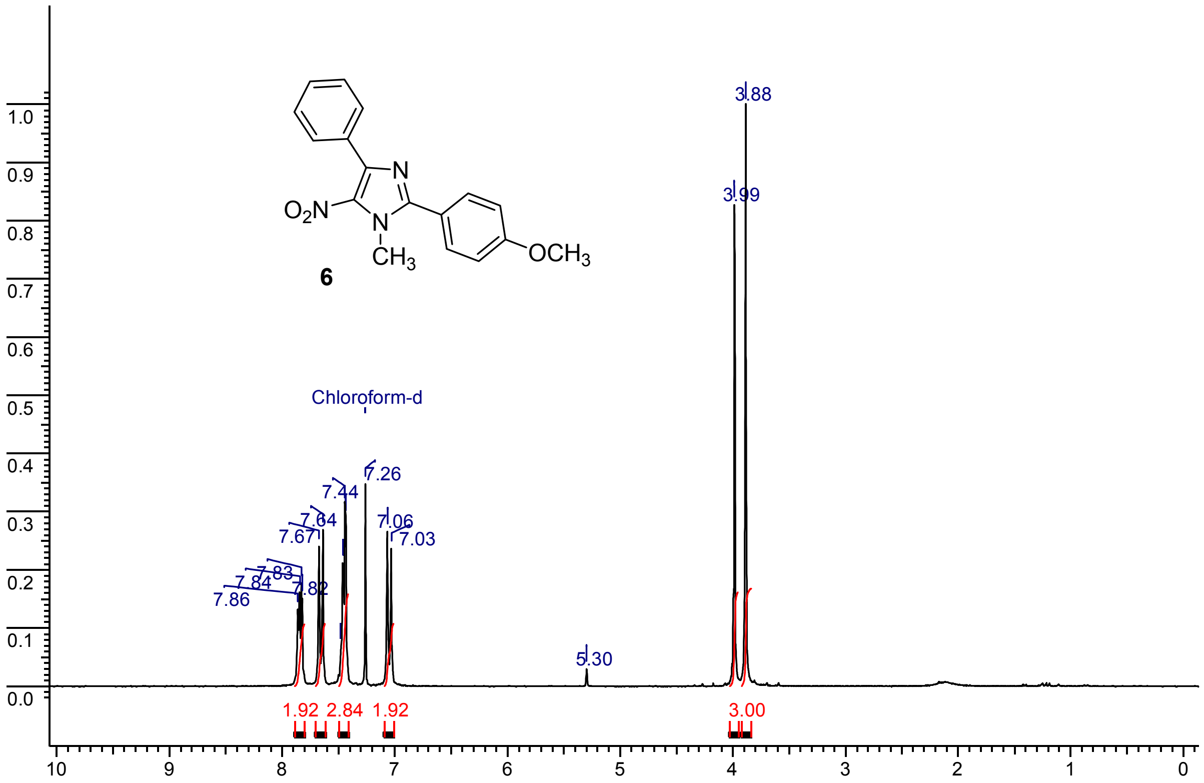

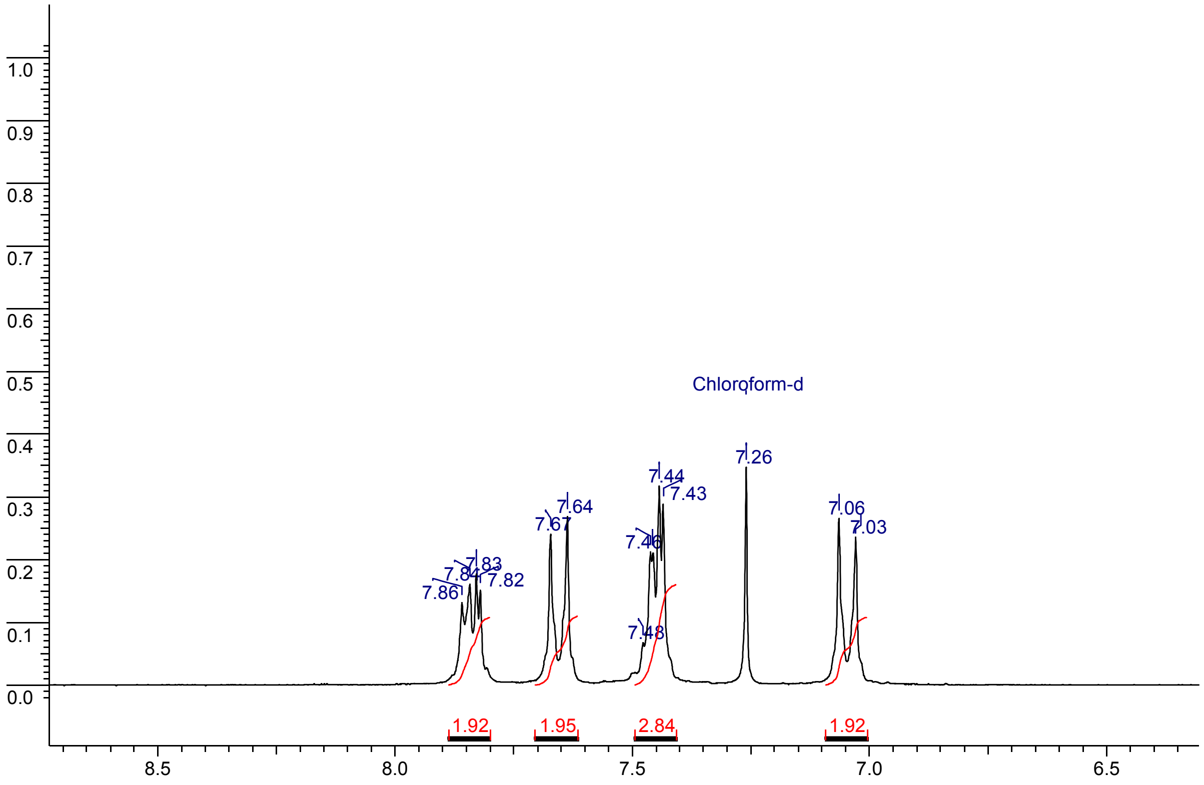

Figure S41. ^{1}H-NMR spectra of **6**

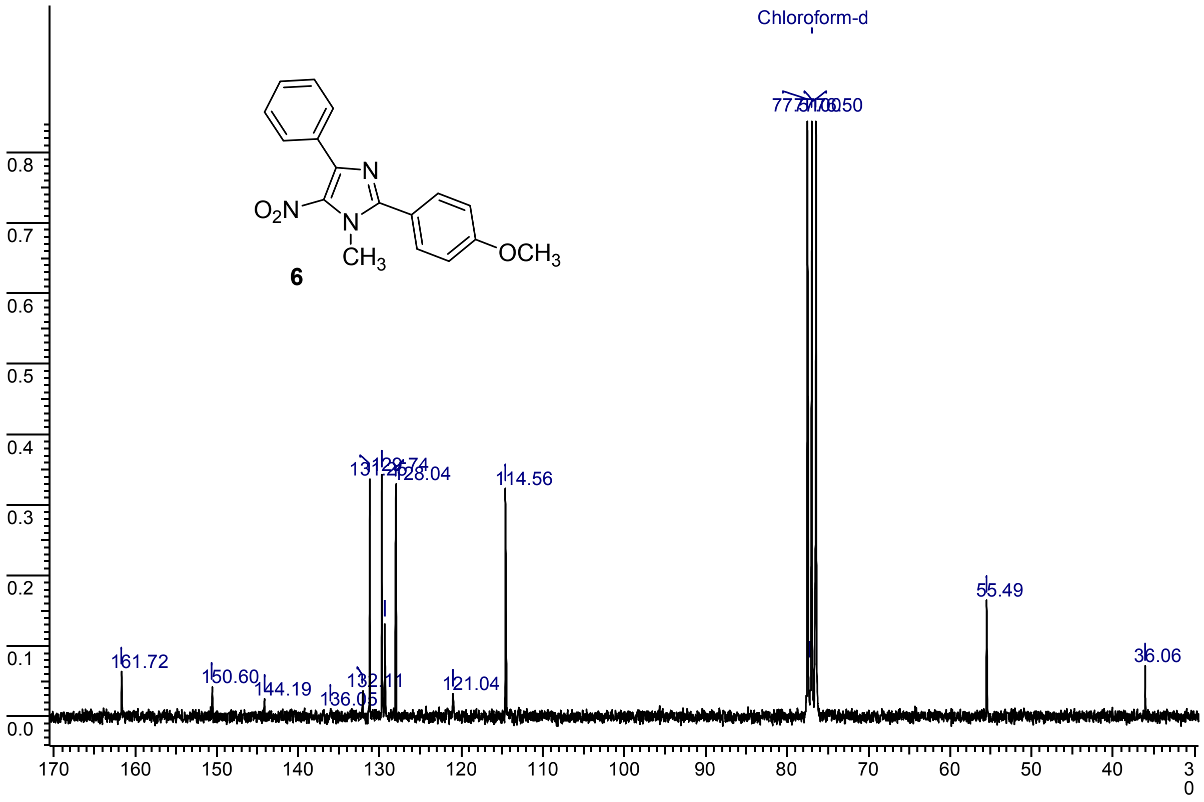

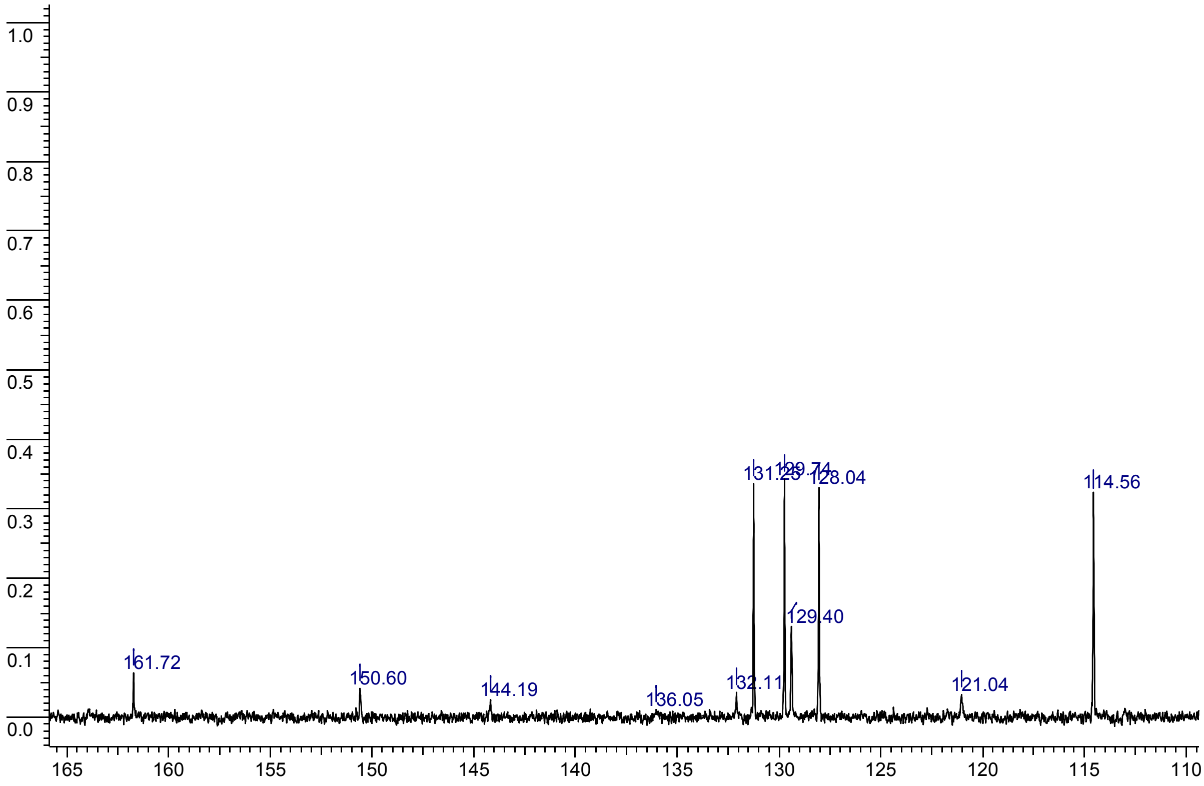

Figure S42. ^{13}C-NMR spectra of **6**

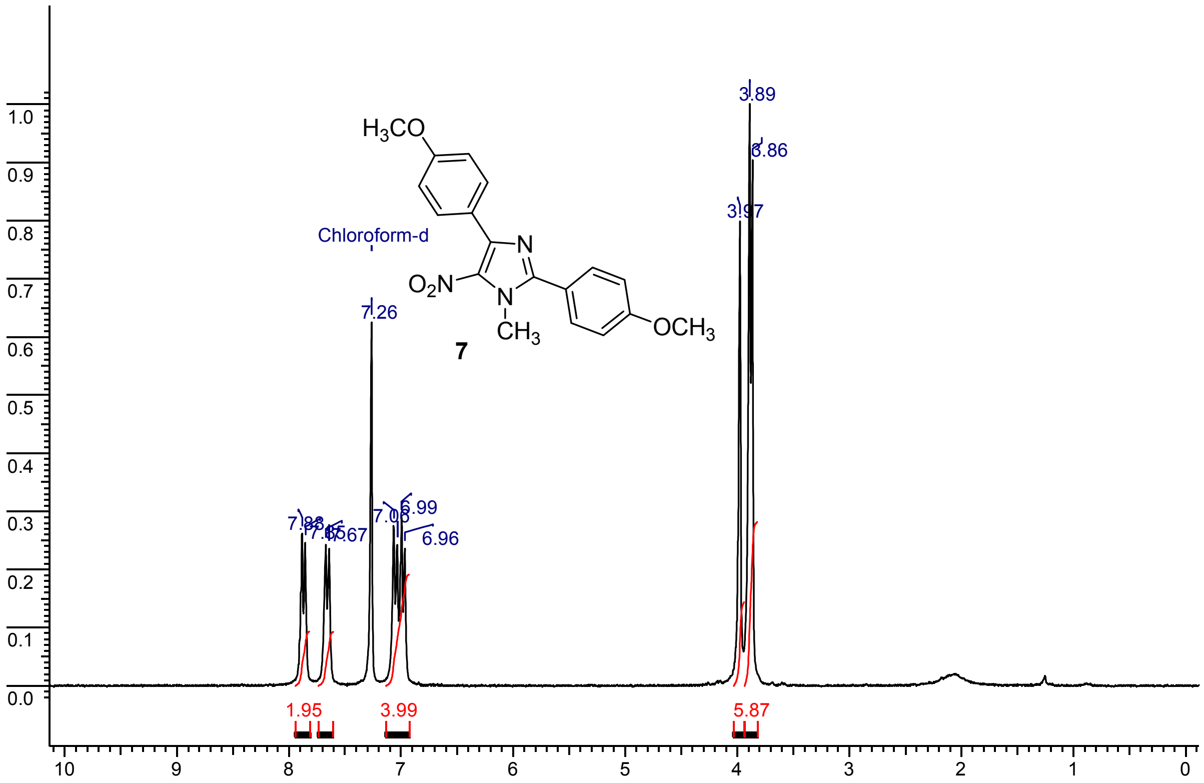

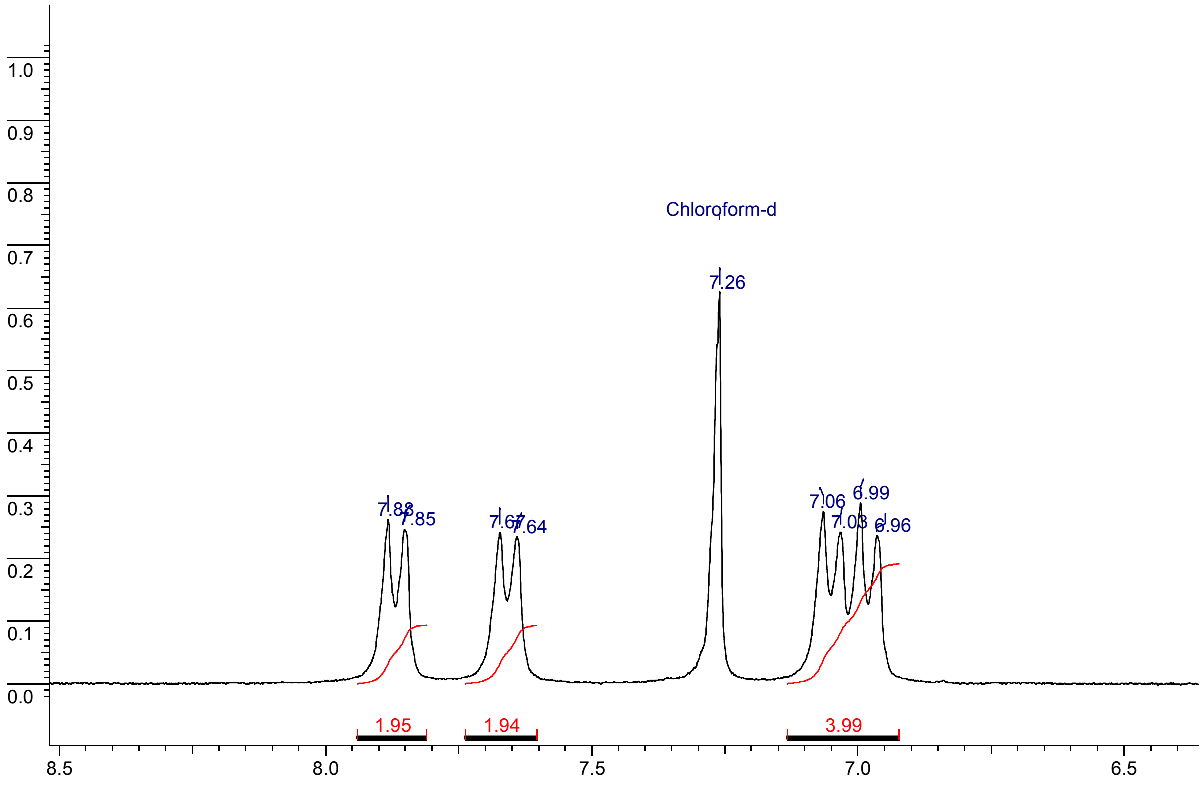

Figure S43. ^{1}H-NMR spectra of 7

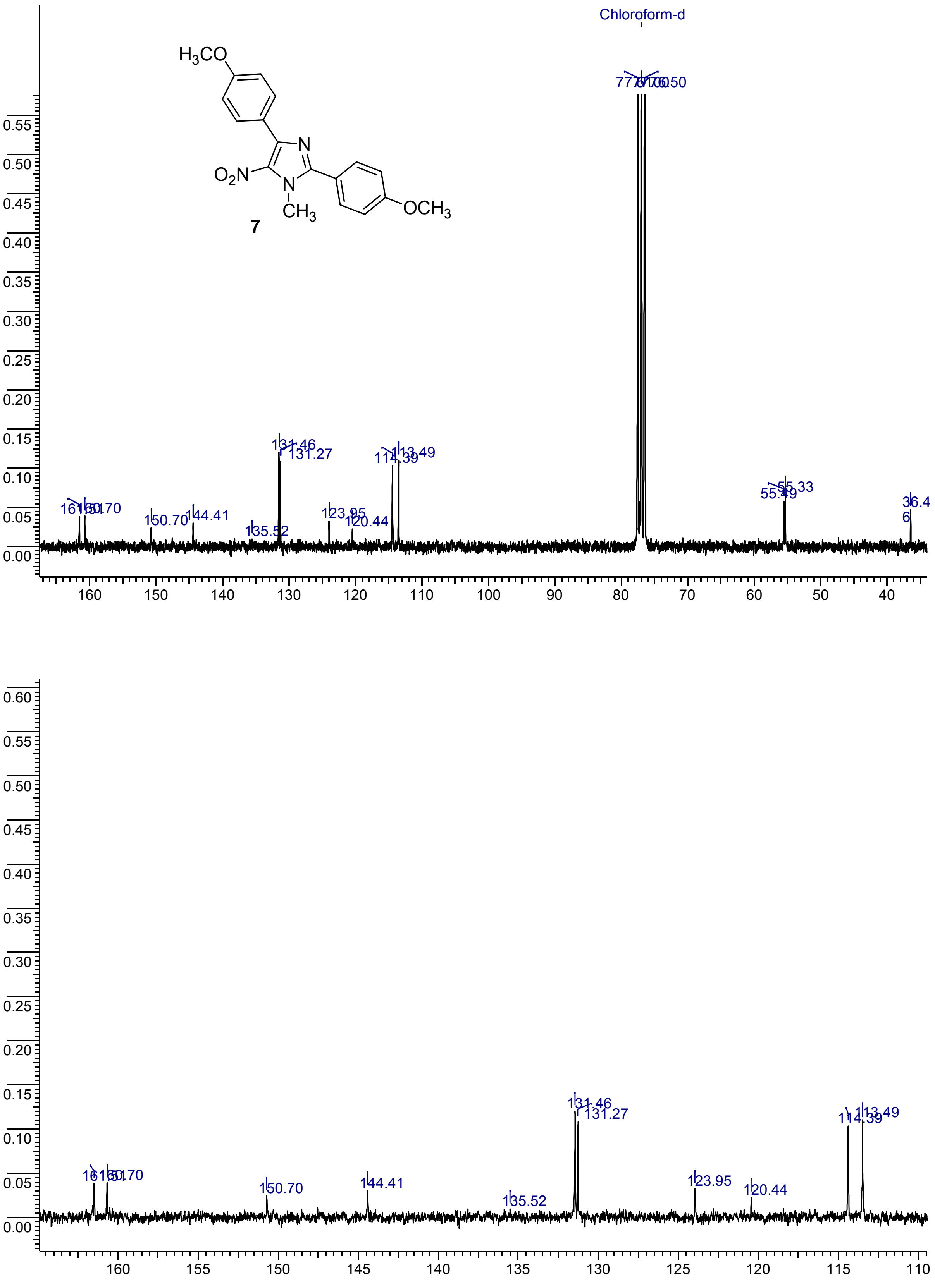

Figure S44. ^{13}C-NMR spectra of **7**

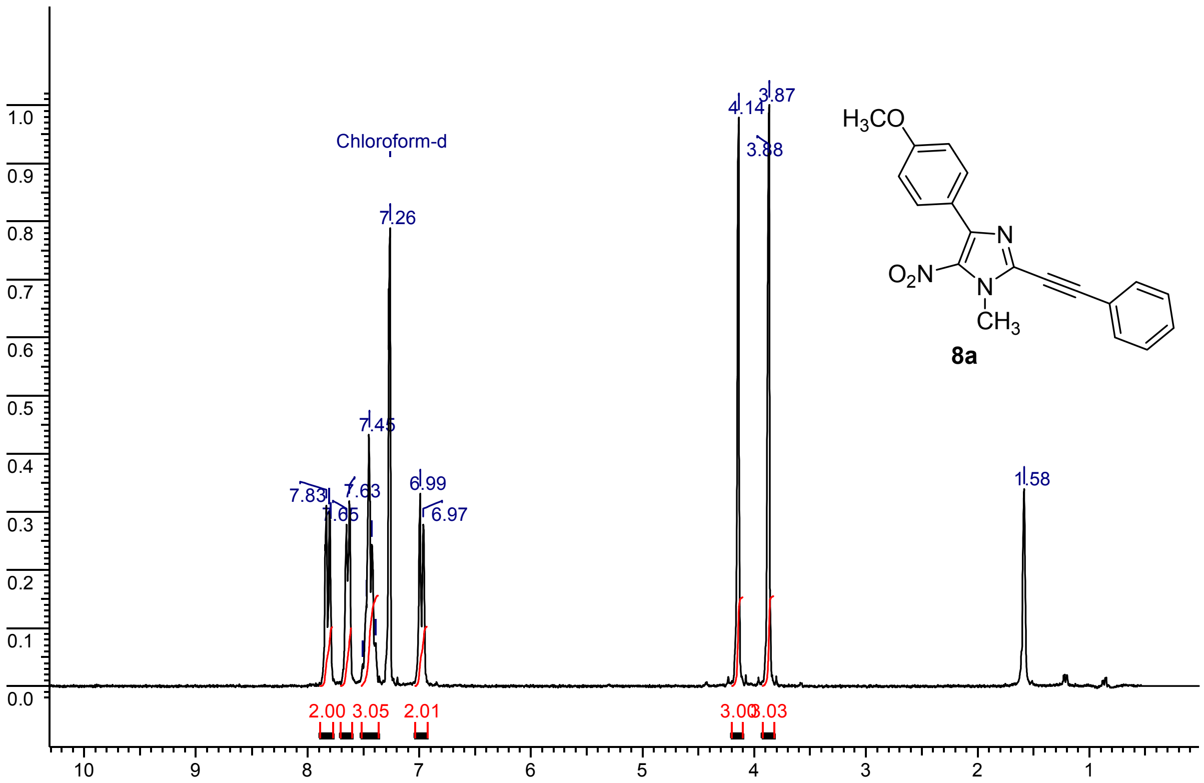

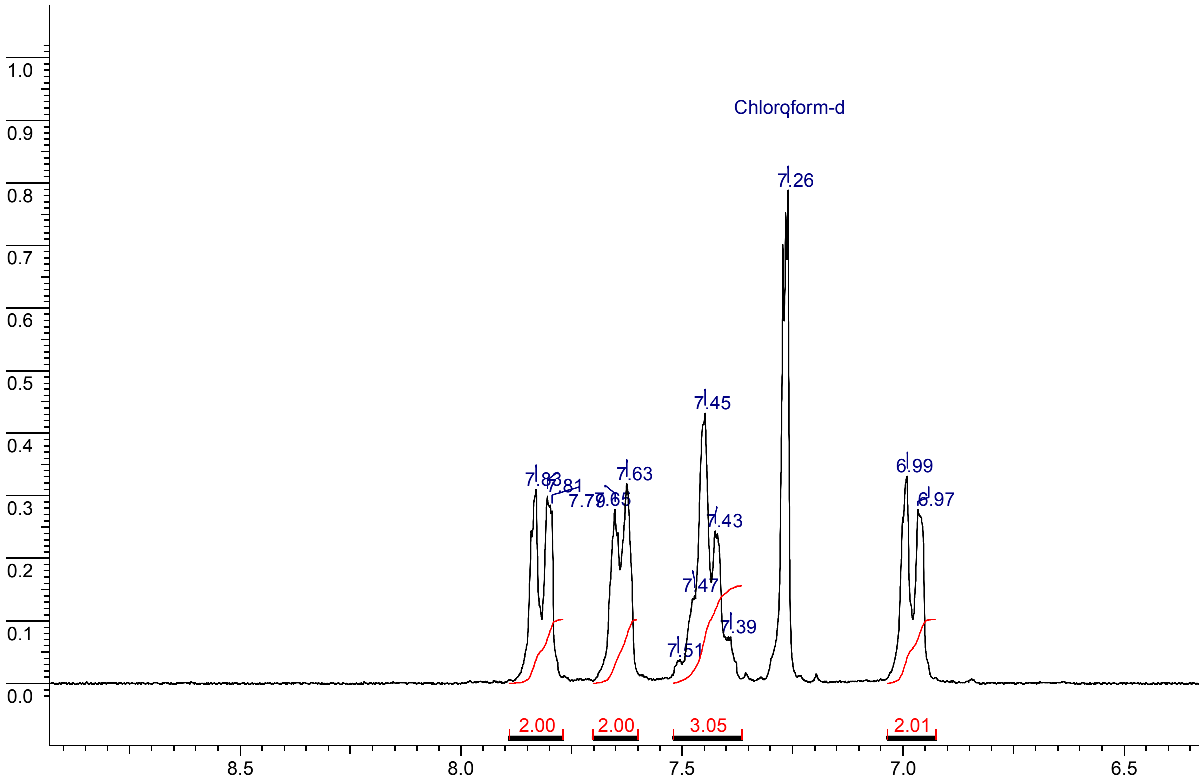

Figure S45. ^{1}H-NMR spectra of **8a**

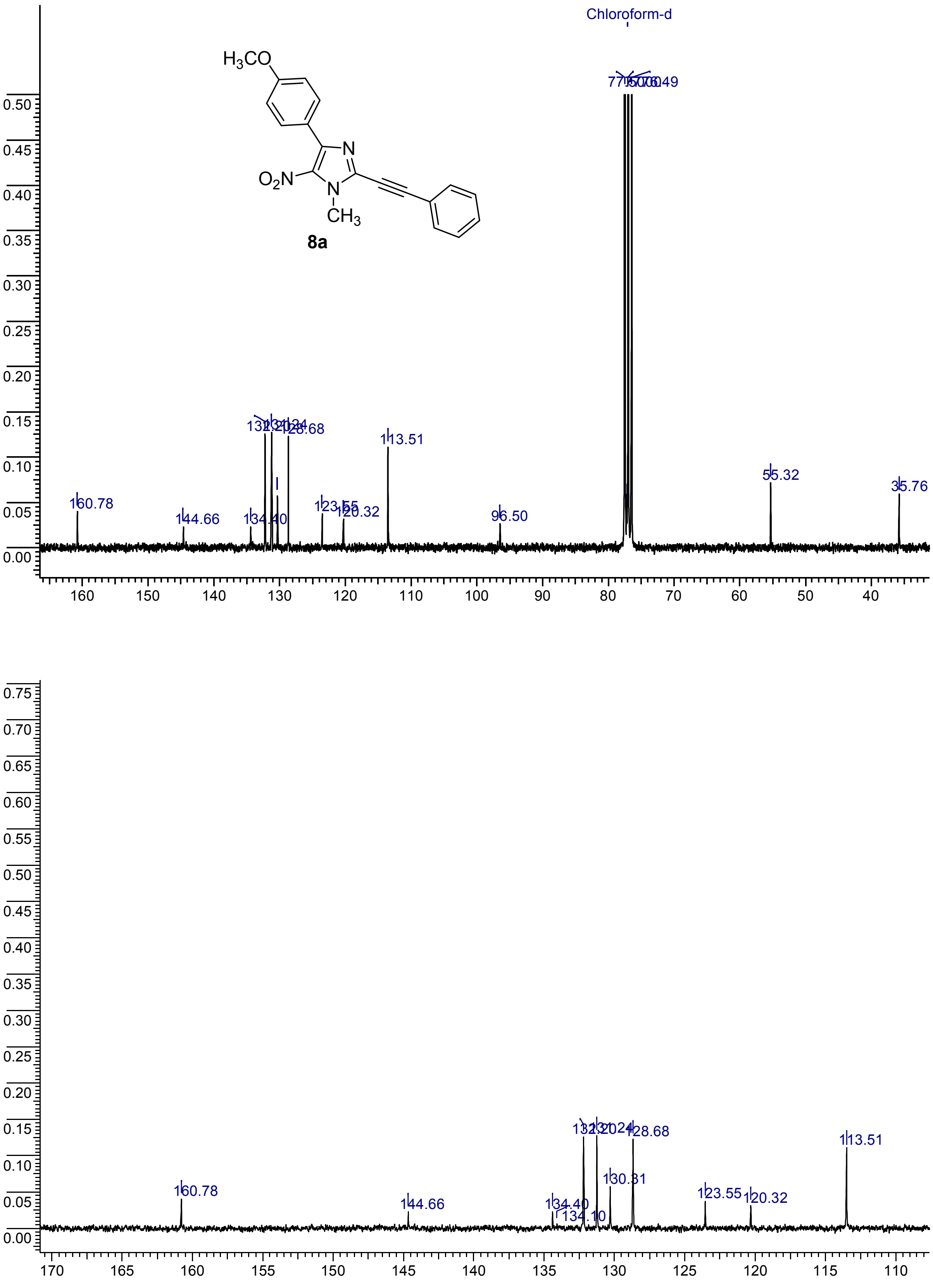

Figure S46. ^{13}C-NMR spectra of **8a**

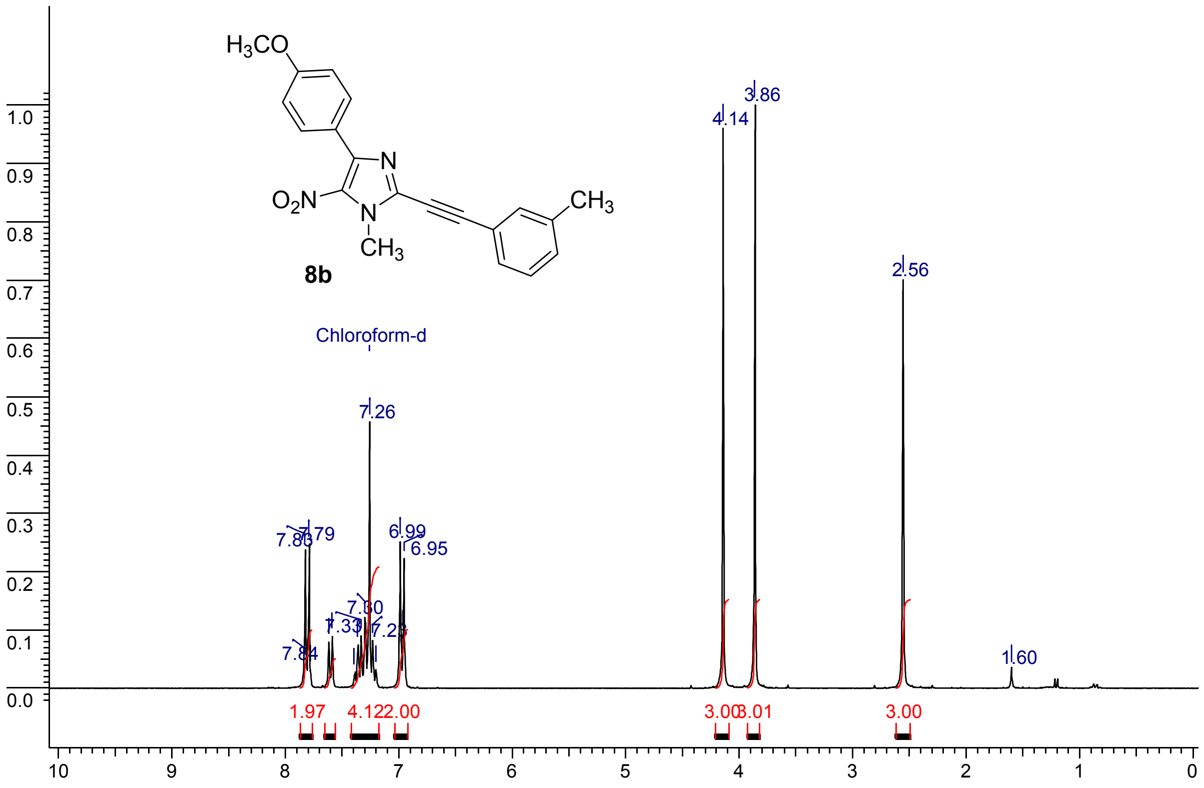

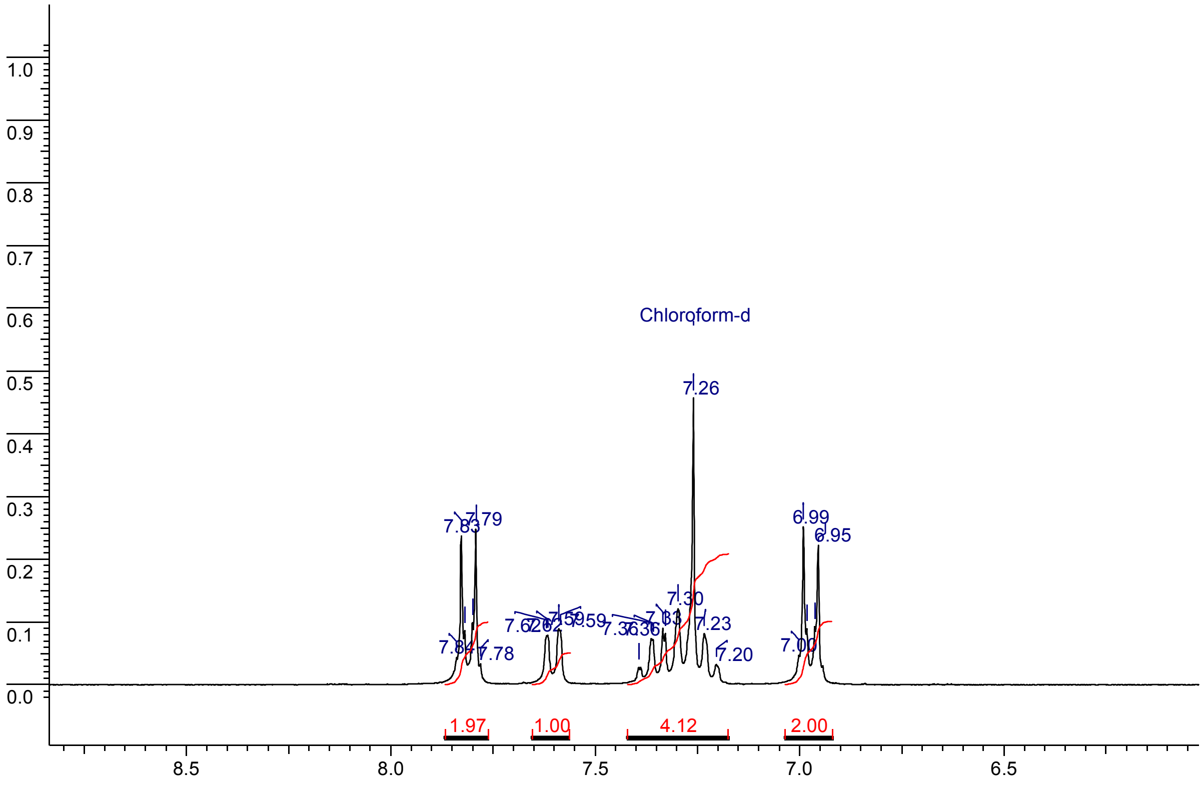

Figure S47. ^{1}H-NMR spectra of **8b**

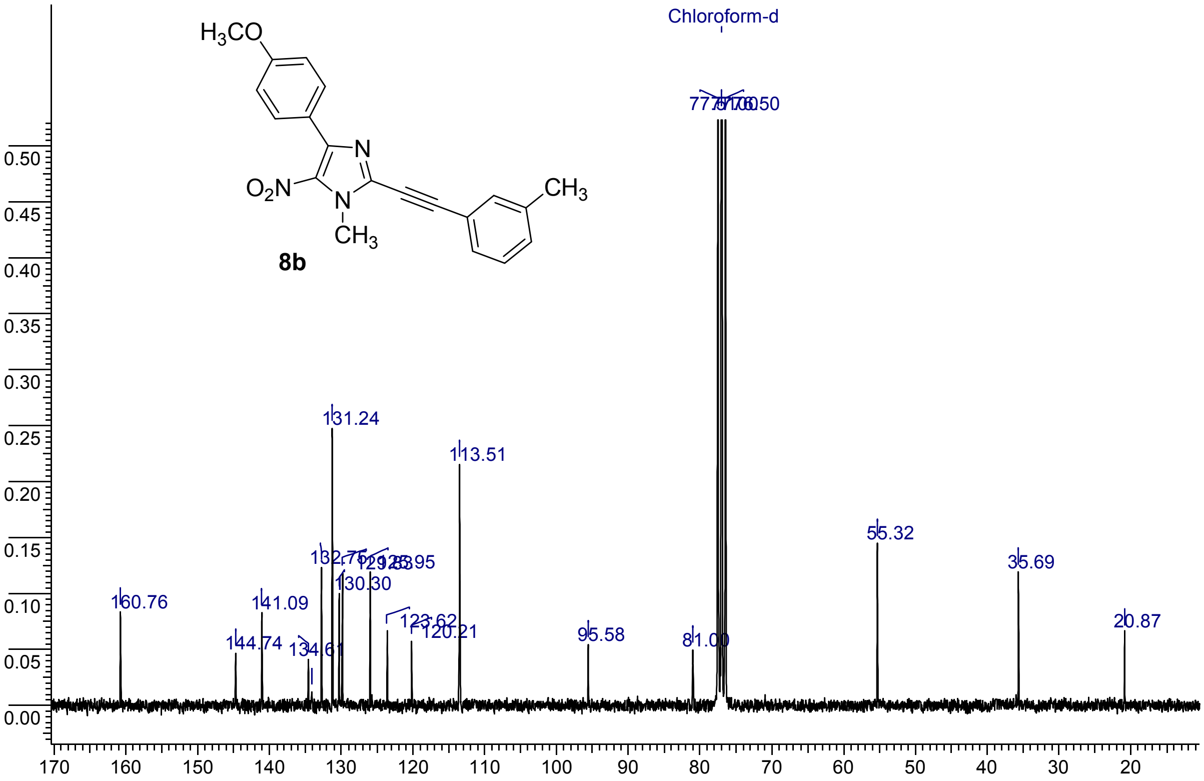

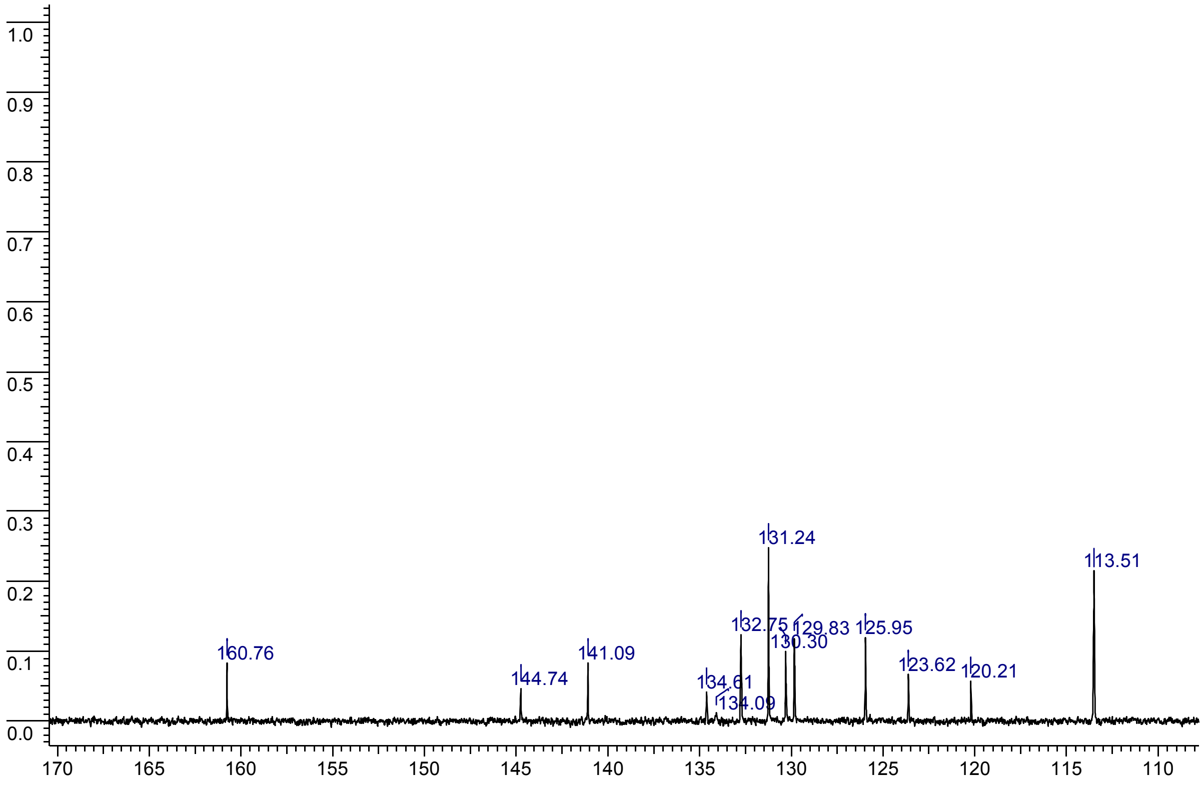

Figure S48. ^{13}C-NMR spectra of **8b**

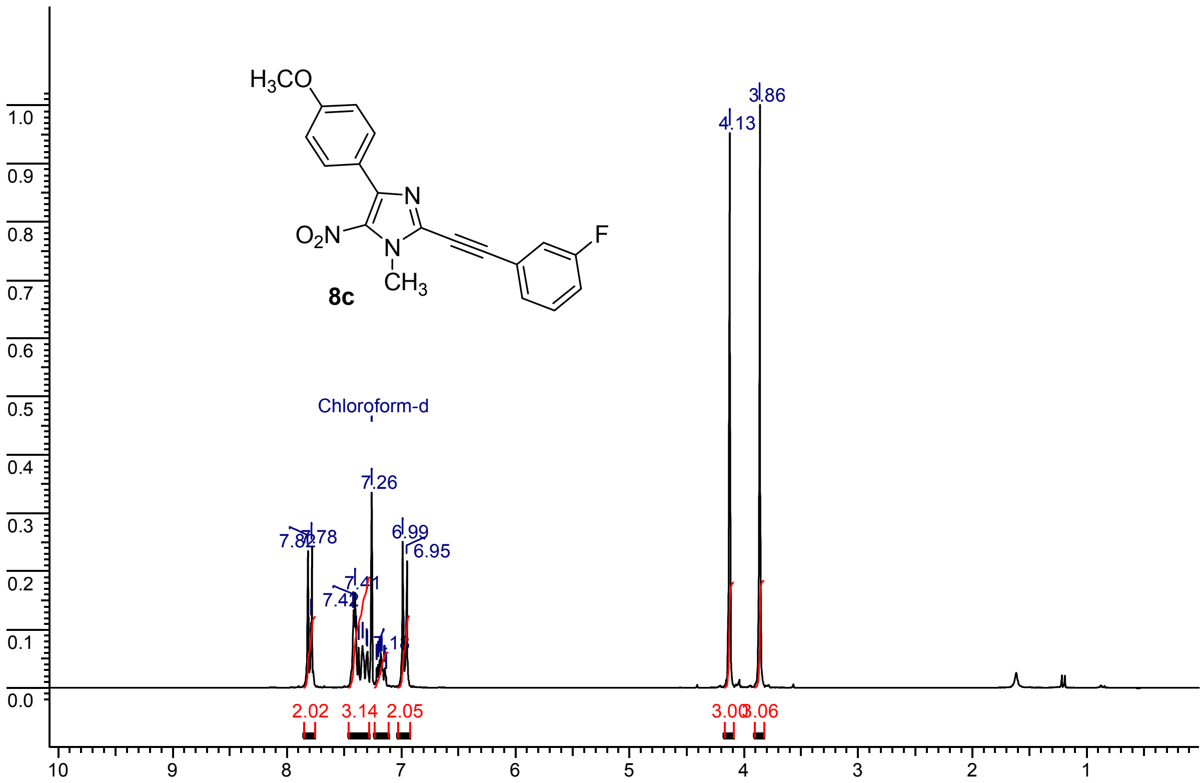

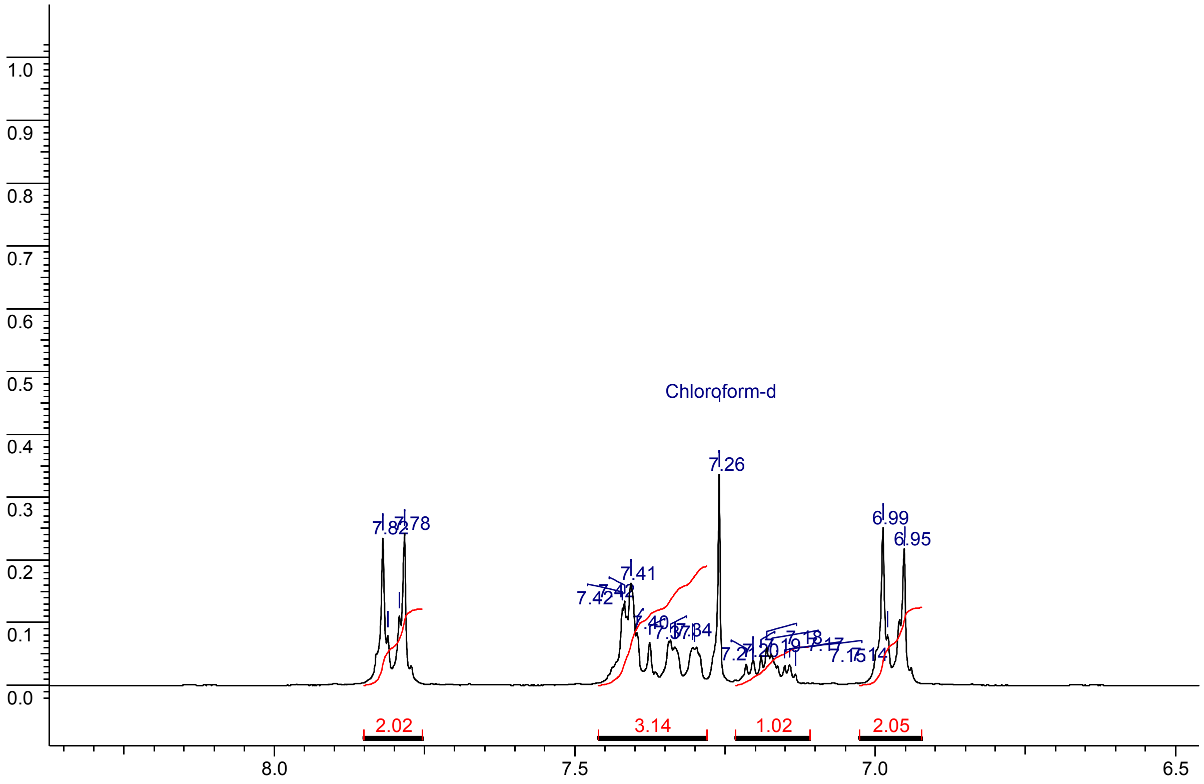

Figure S49. ^{1}H-NMR spectra of **8c**

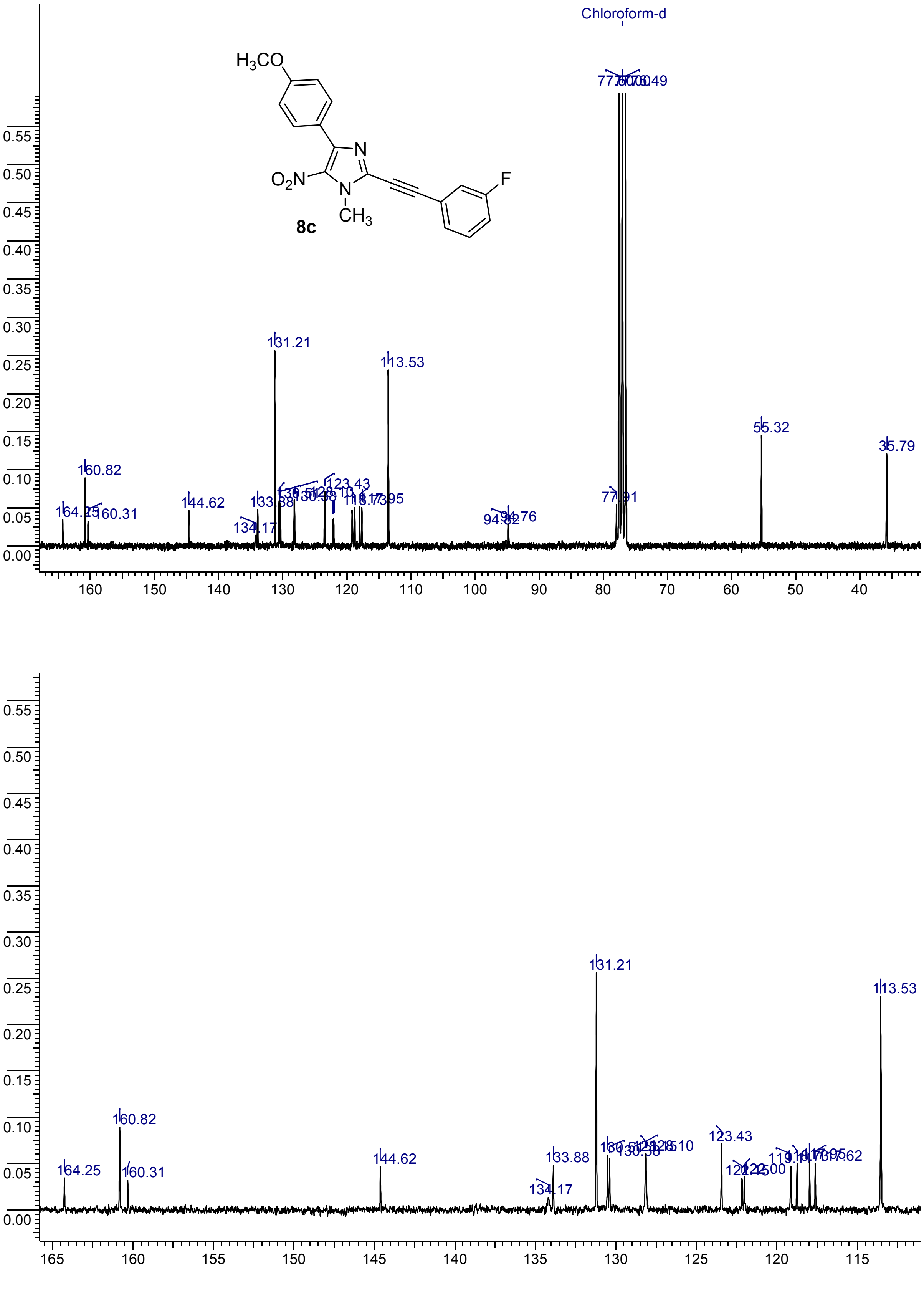

Figure S50. ^{13}C-NMR spectra of **8c**

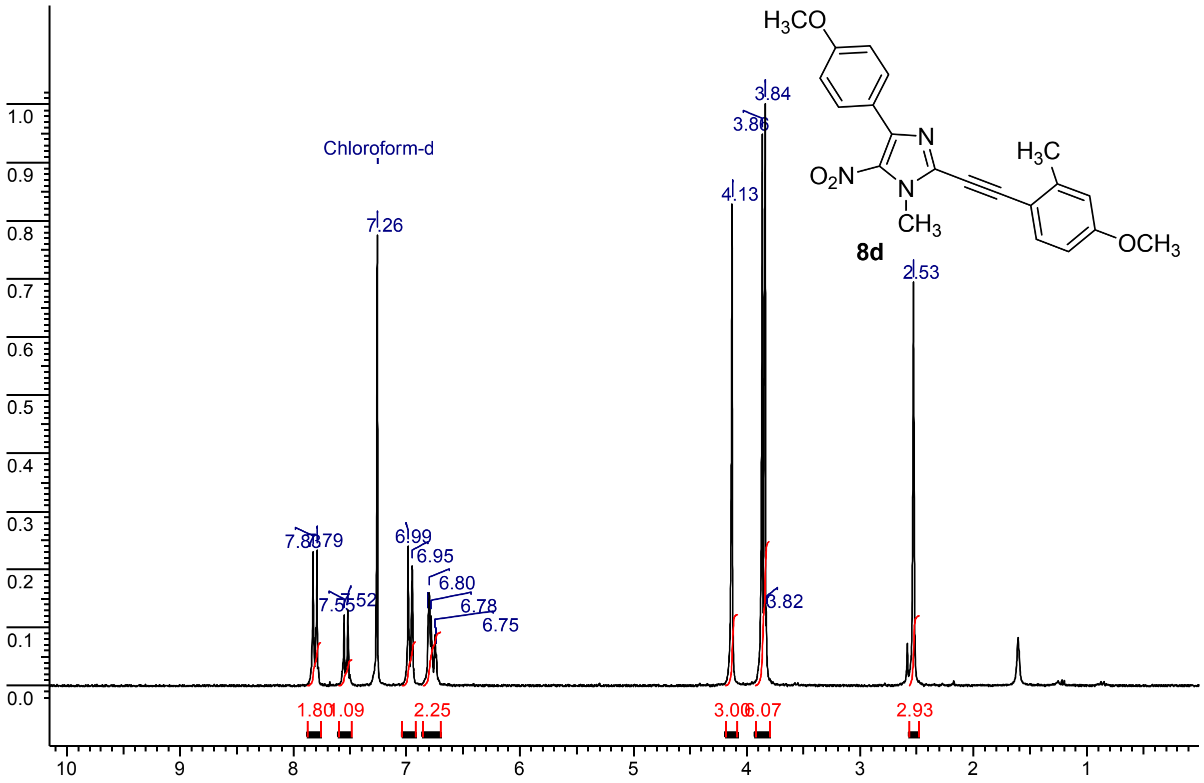

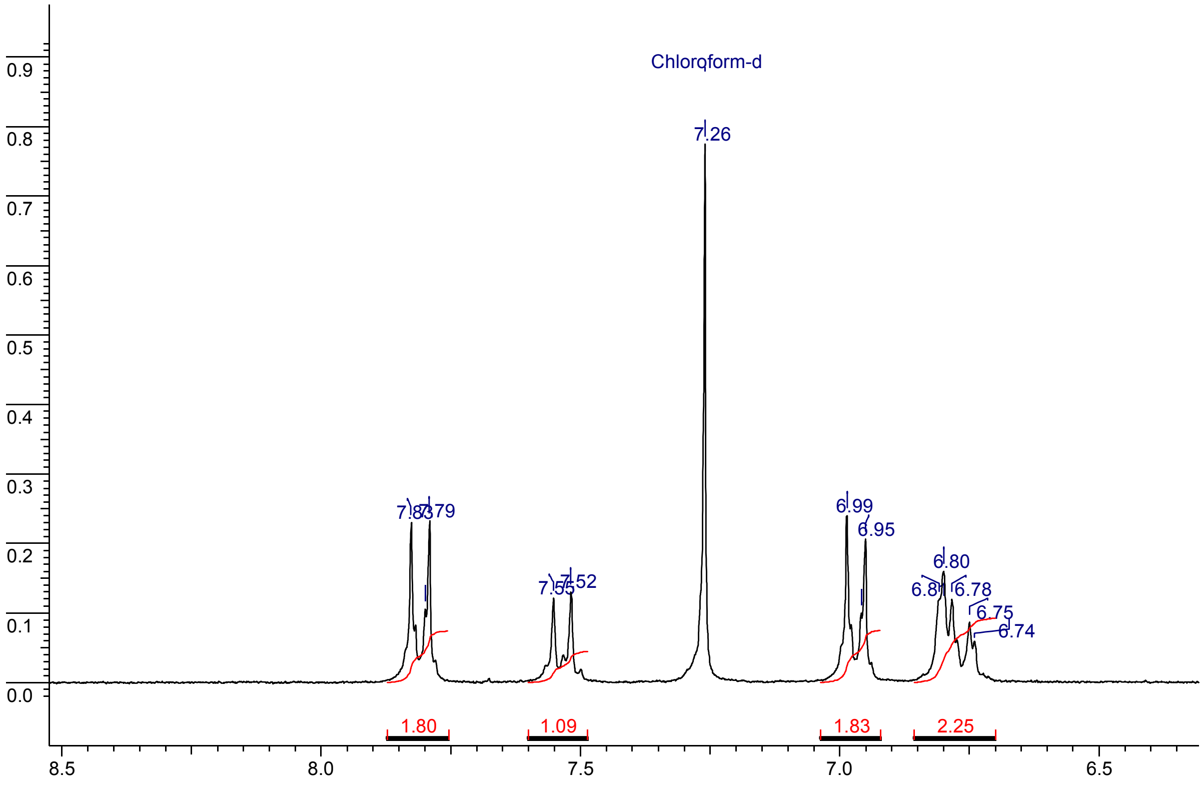

Figure S51. ^{1}H-NMR spectra of **8d**

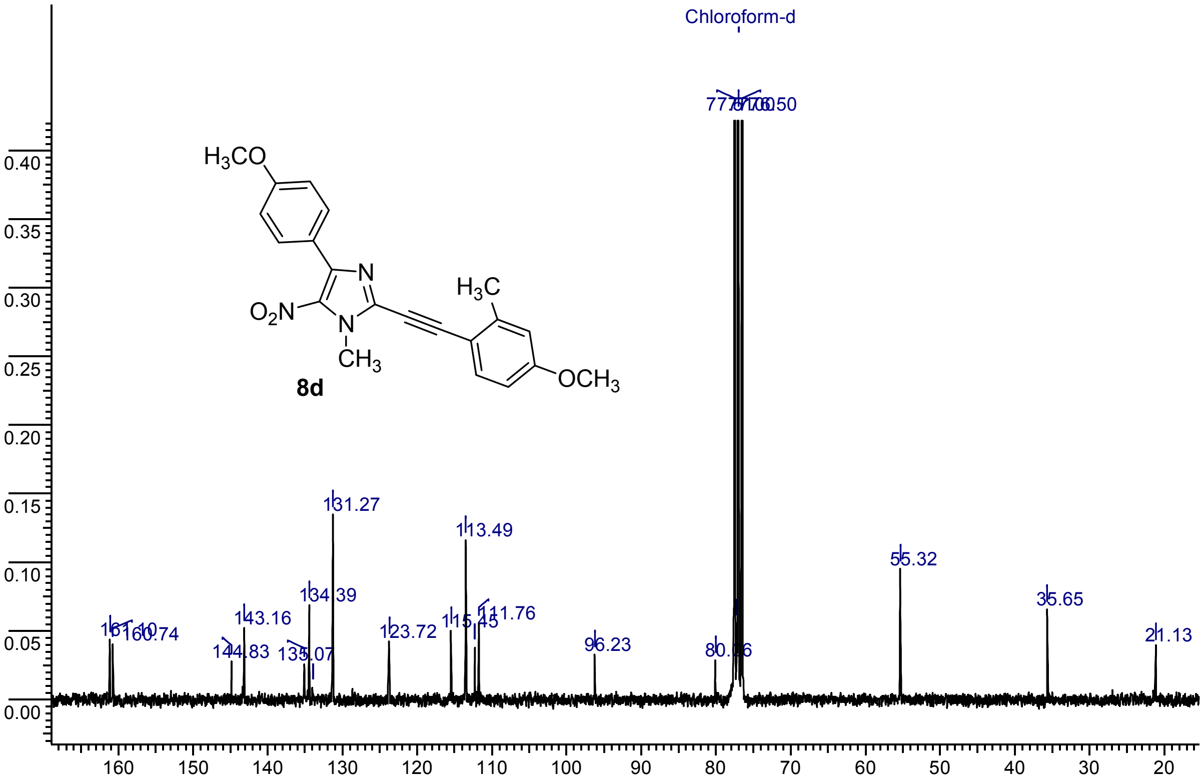

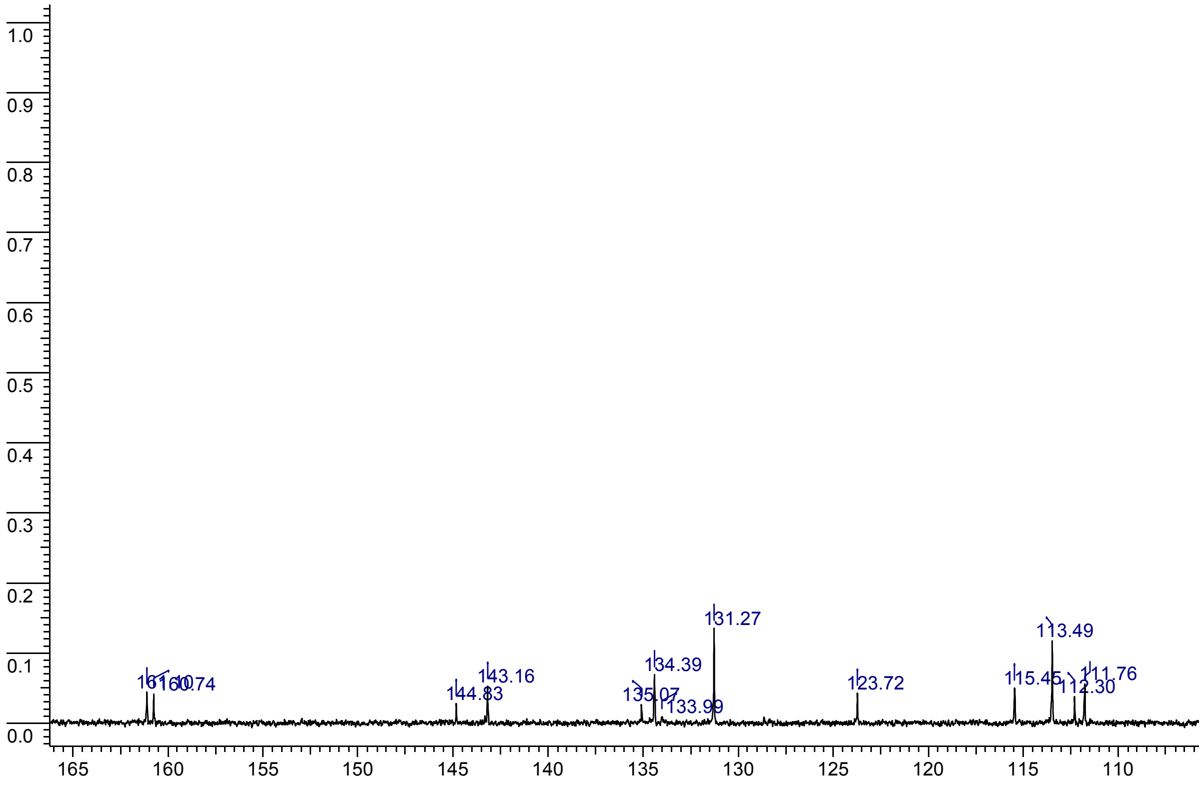

Figure S52. ^{13}C-NMR spectra of **8d**

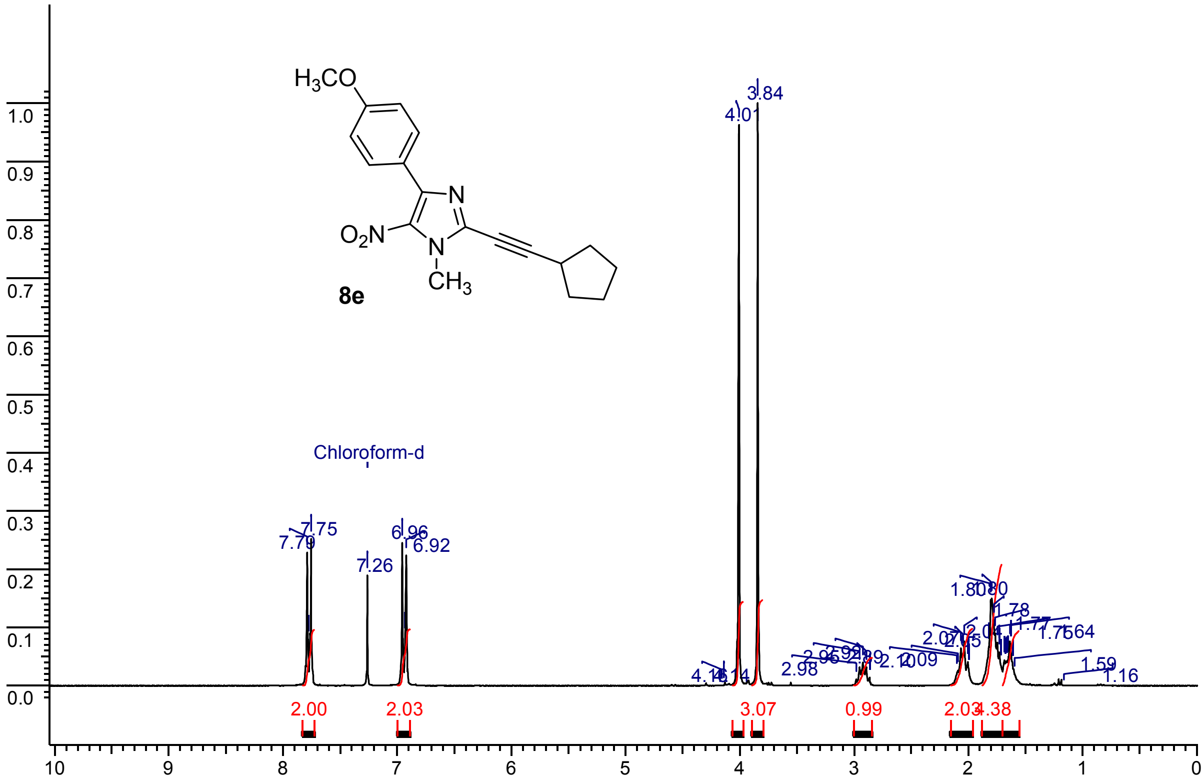

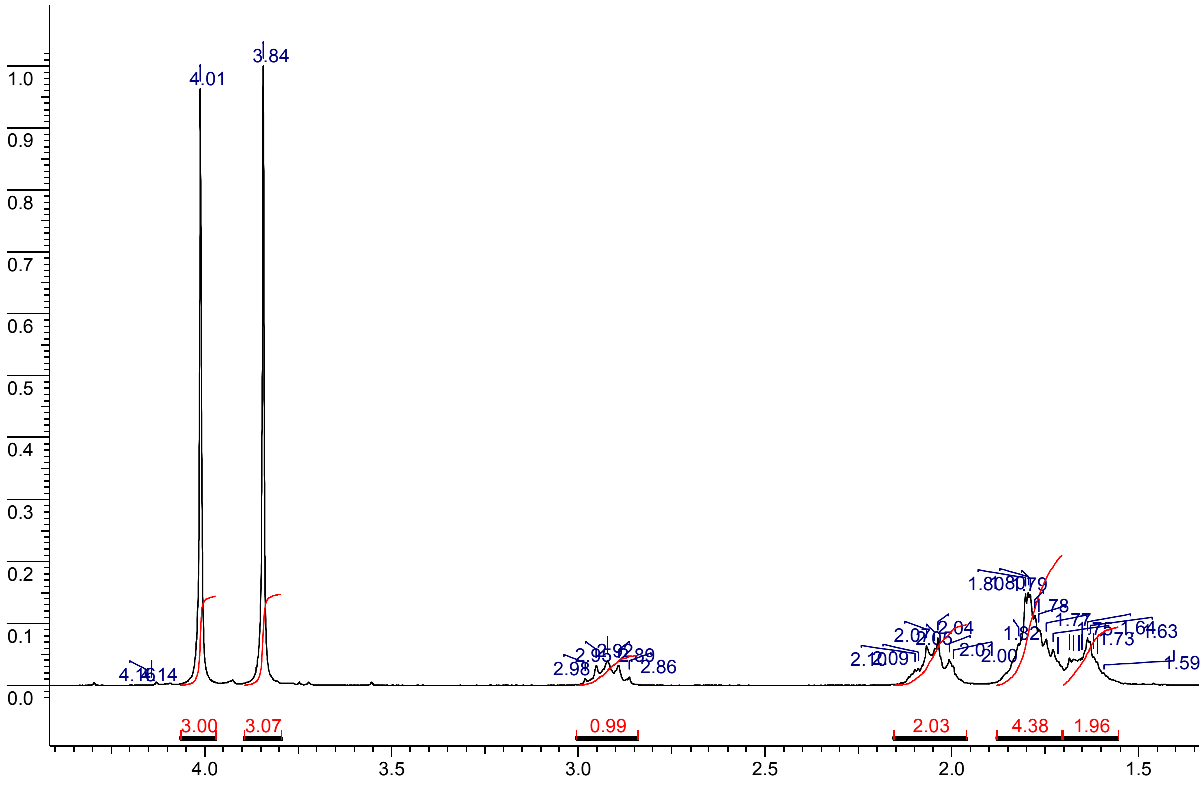

Figure S53. ^{1}H-NMR spectra of **8e**

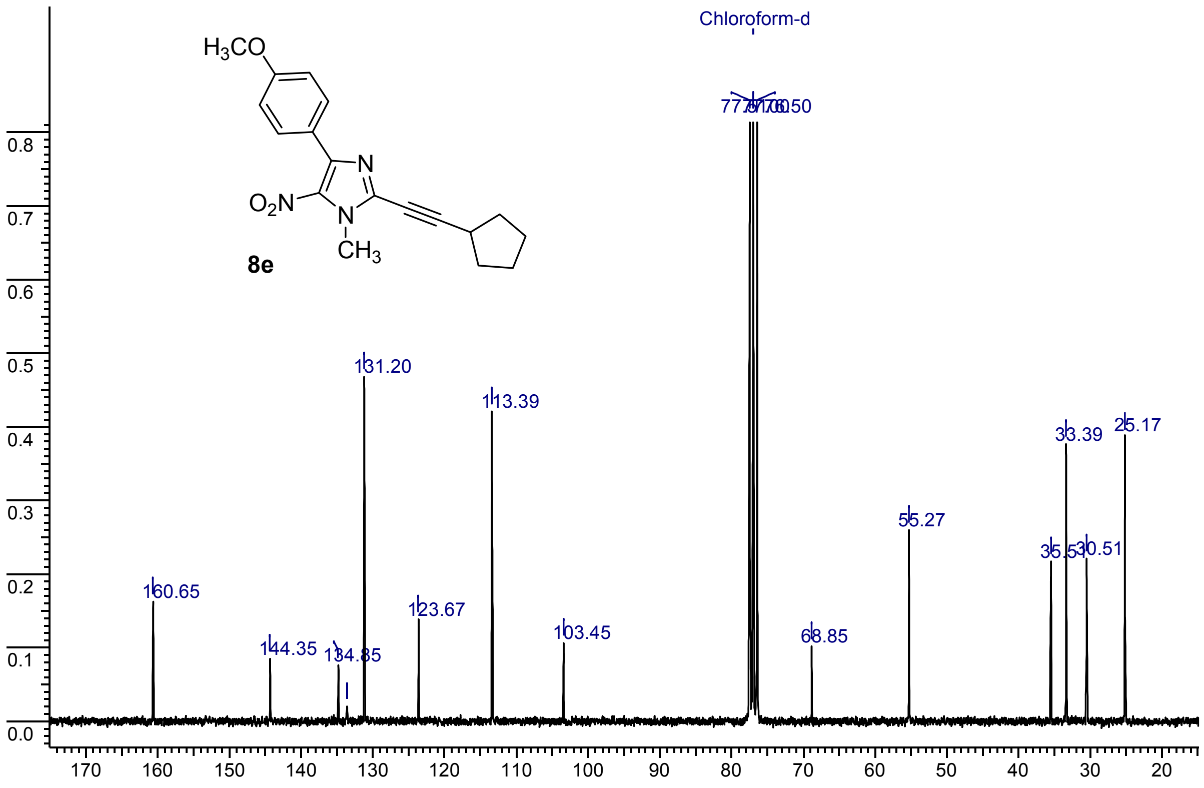

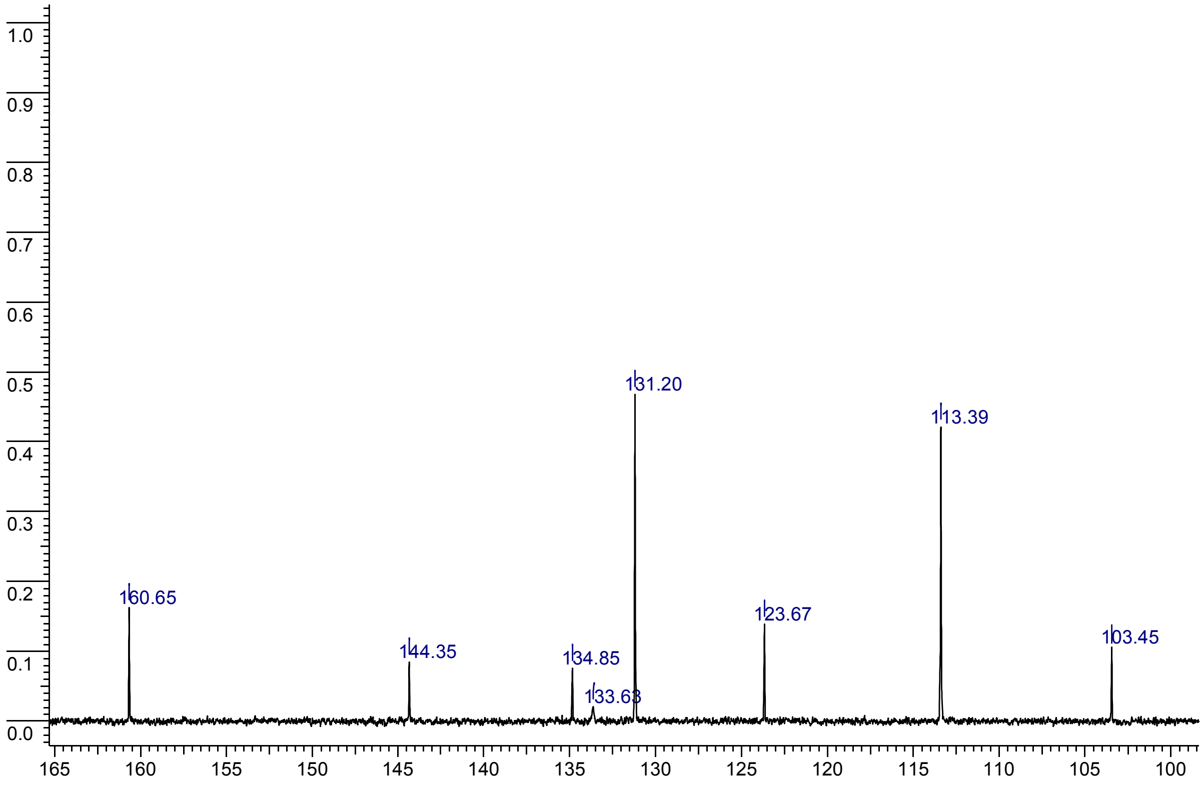

Figure S54. ^{13}C-NMR spectra of **8e**

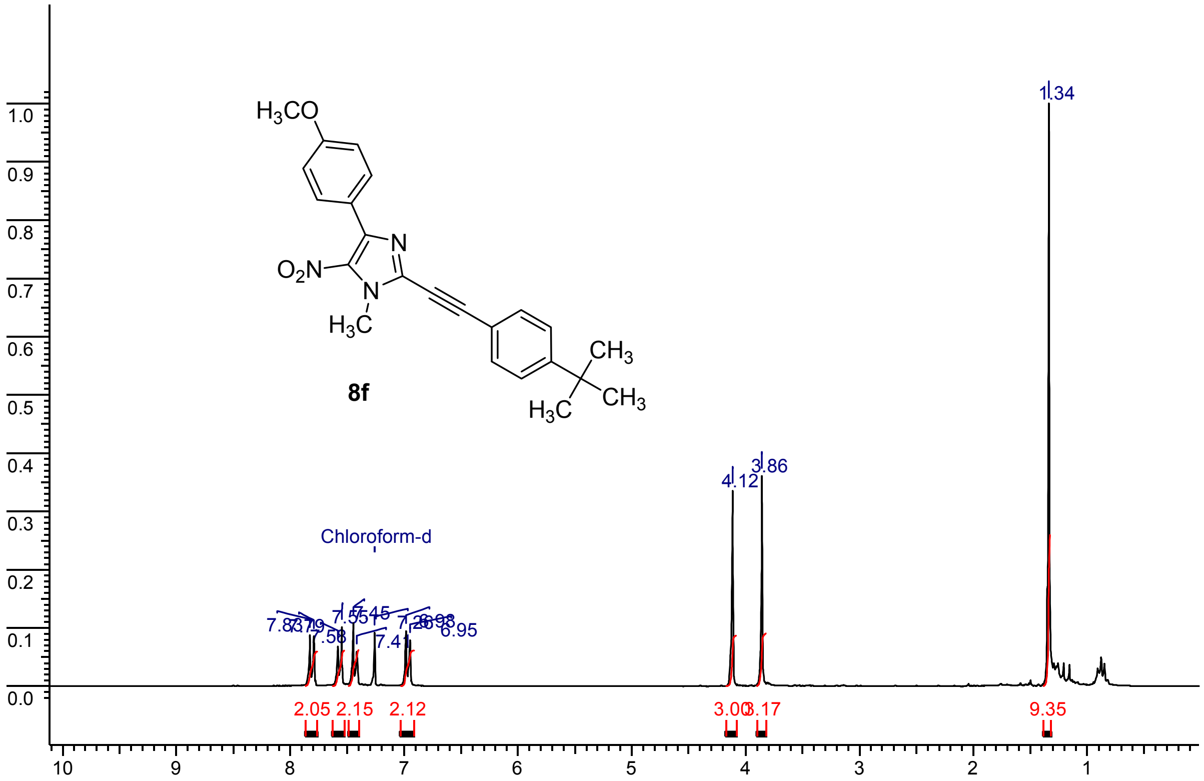

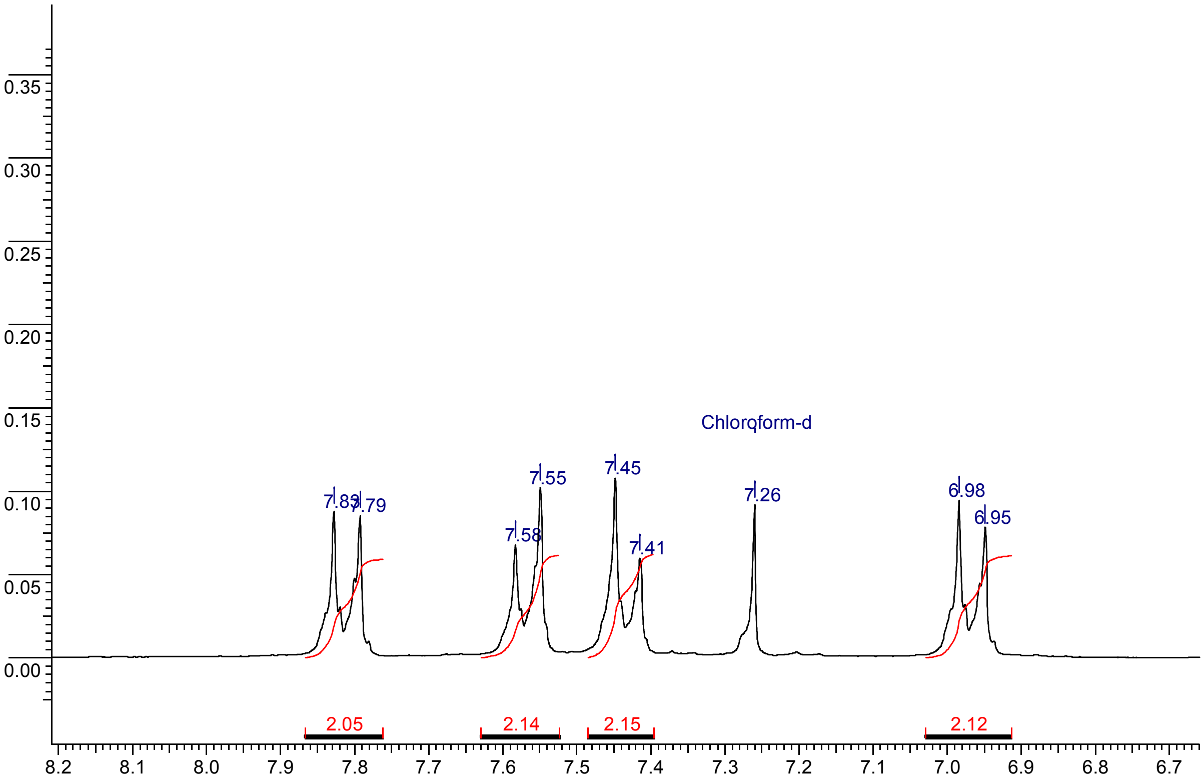

Figure S55. ^{1}H-NMR spectra of **8f**

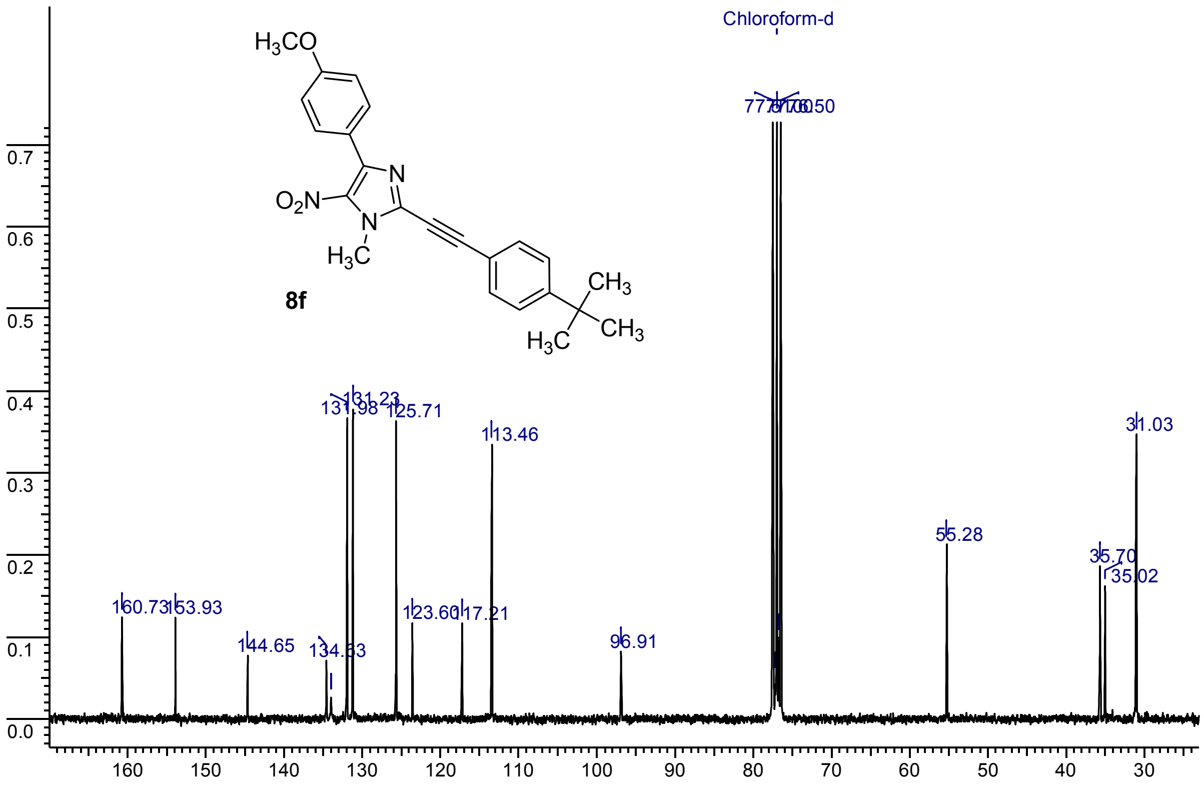

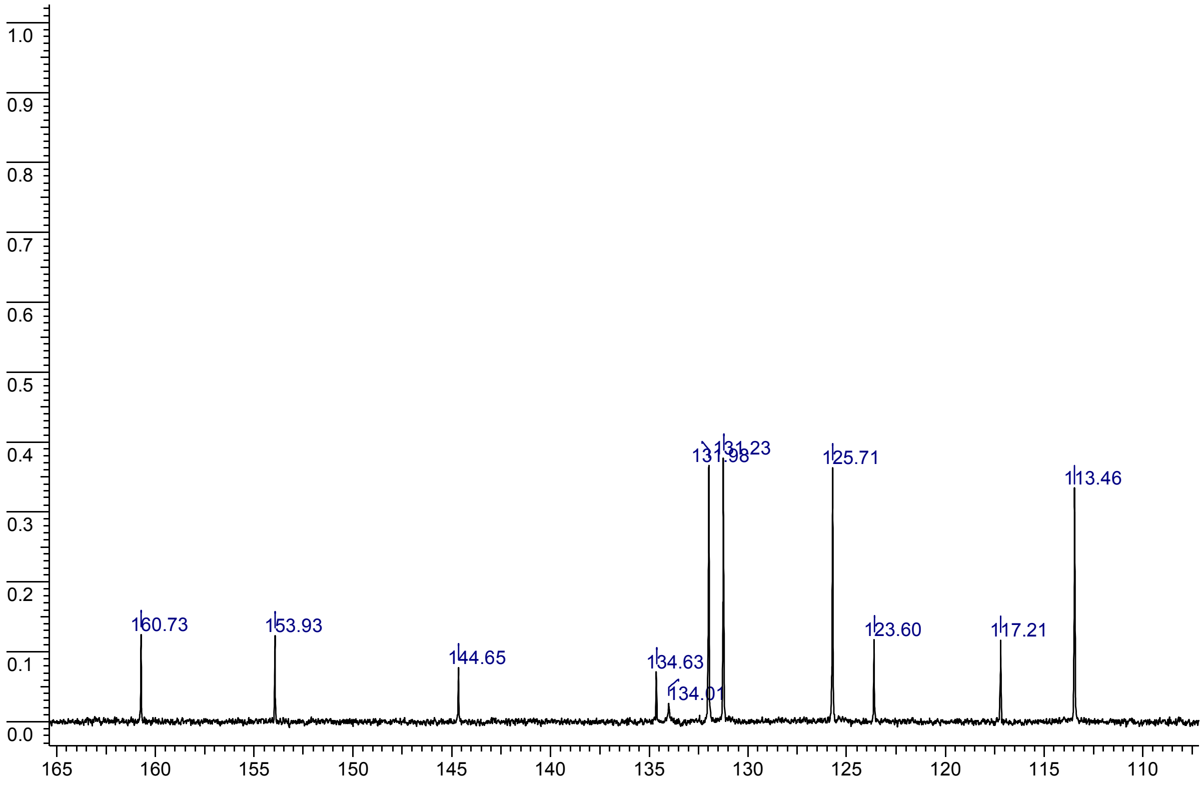

Figure S56. ^{13}C-NMR spectra of **8f**

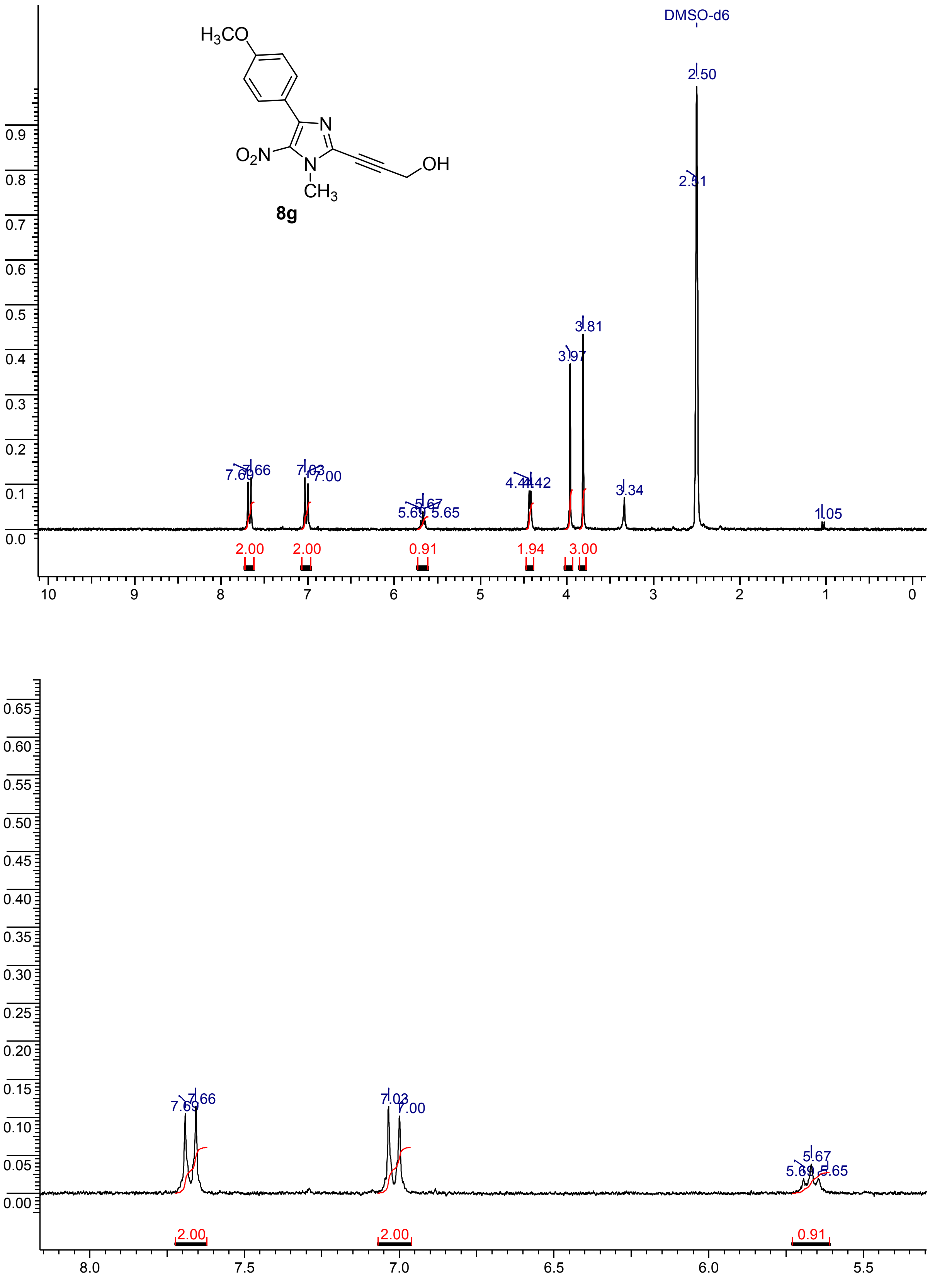

Figure S57. ^{1}H-NMR spectra of **8g**

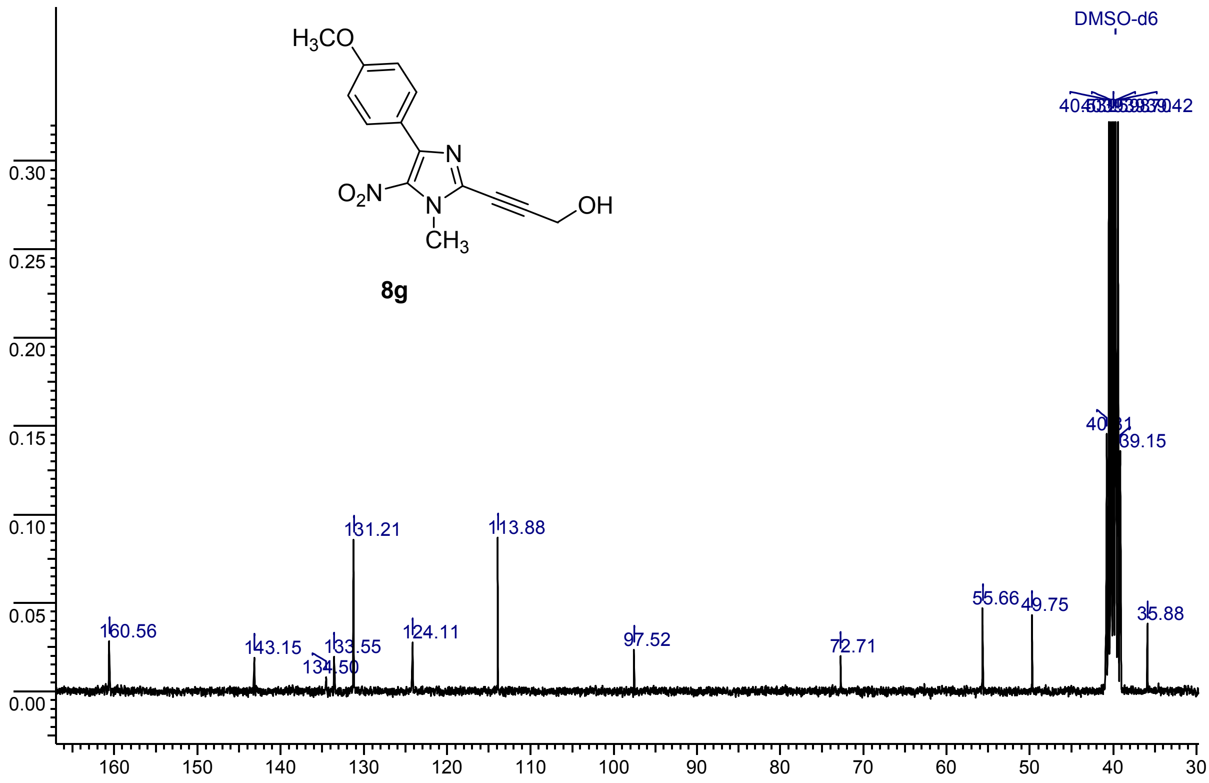

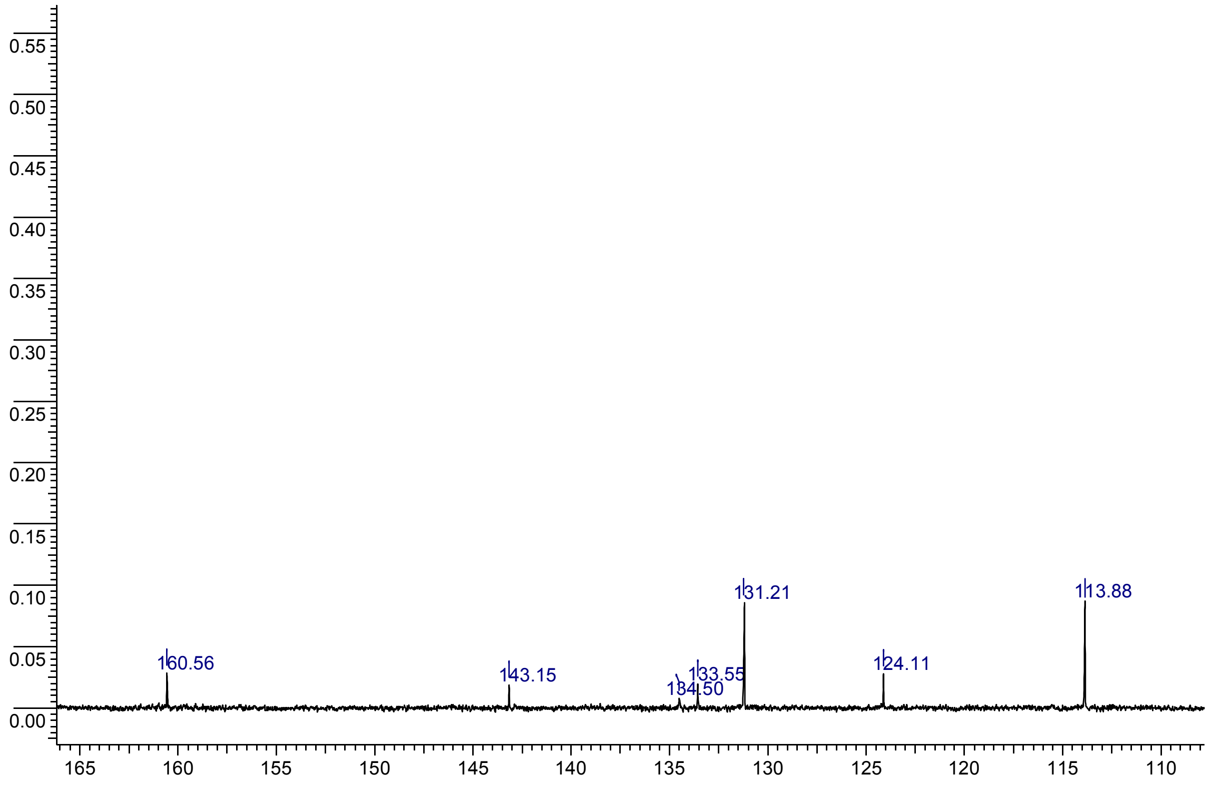

Figure S58. ^{13}C-NMR spectra of **8g**

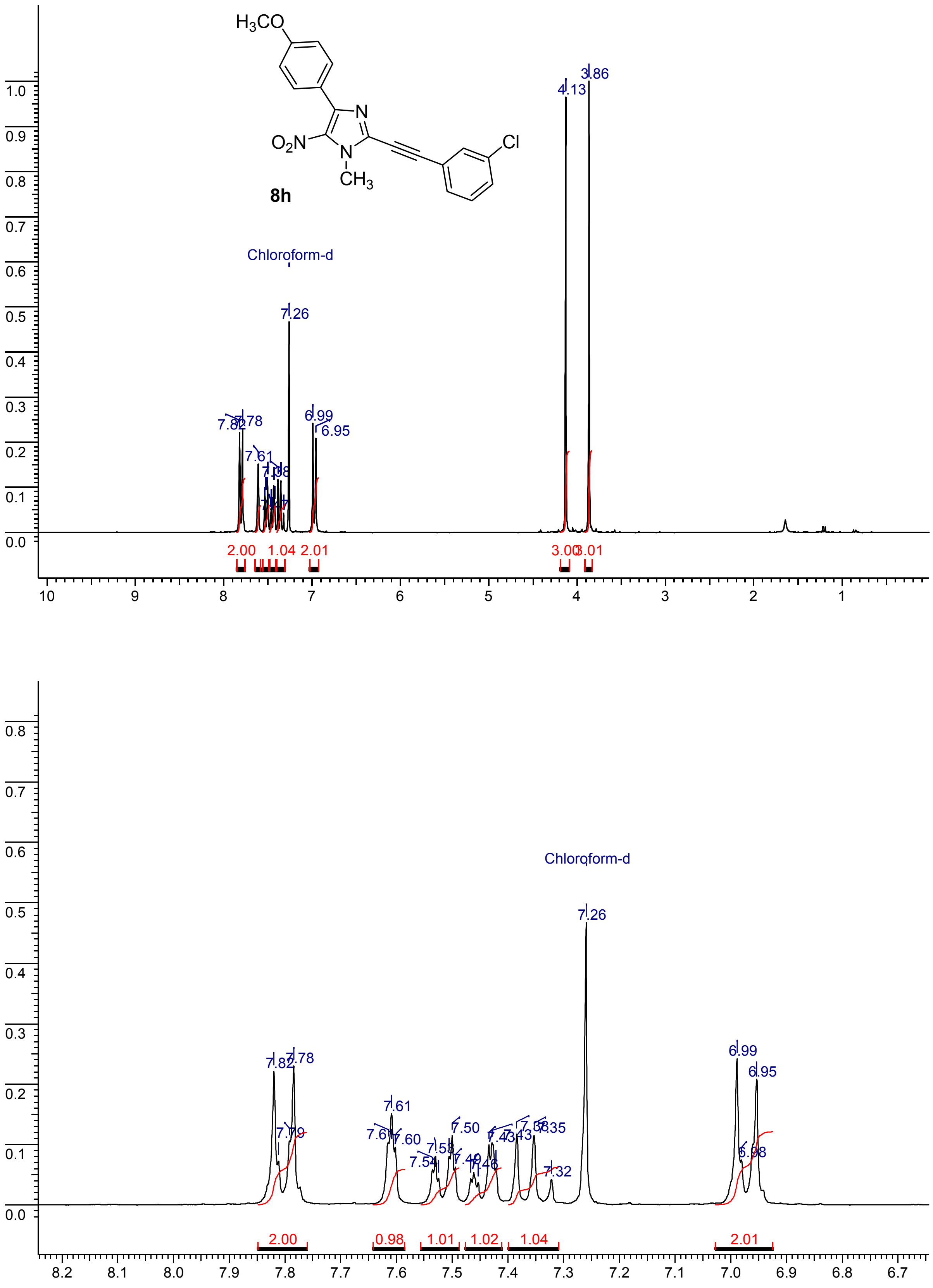

Figure S59. ^{1}H-NMR spectra of **8h**

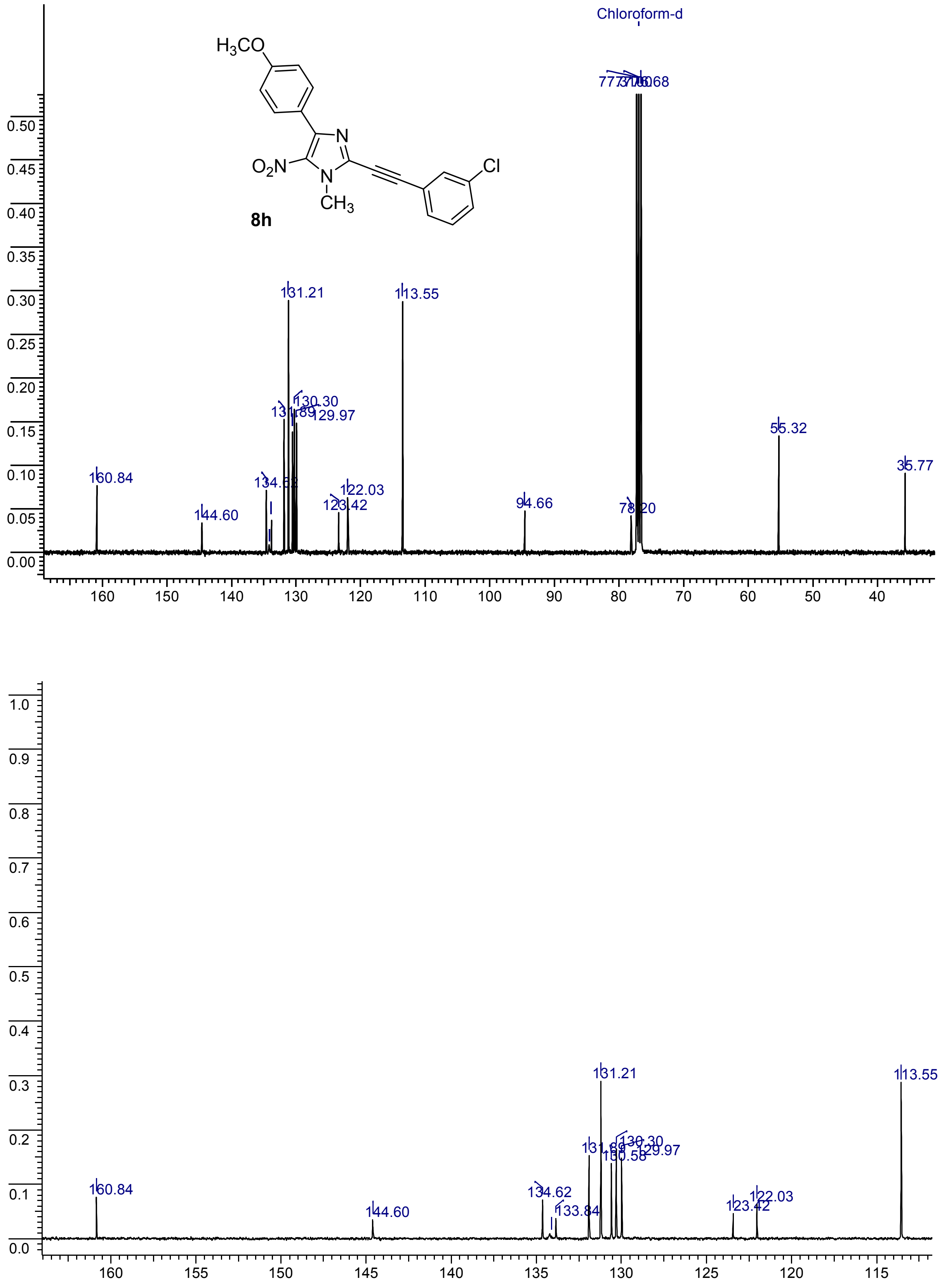

Figure S60. ^{13}C-NMR spectra of **8h**

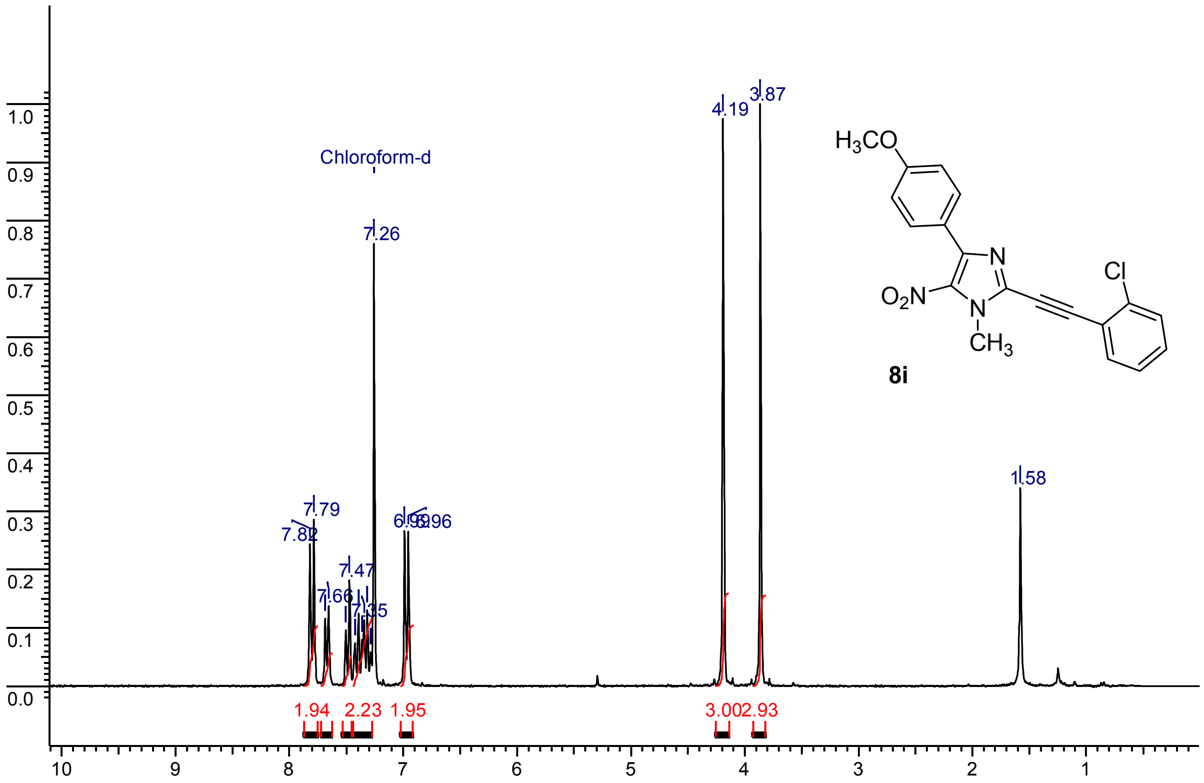

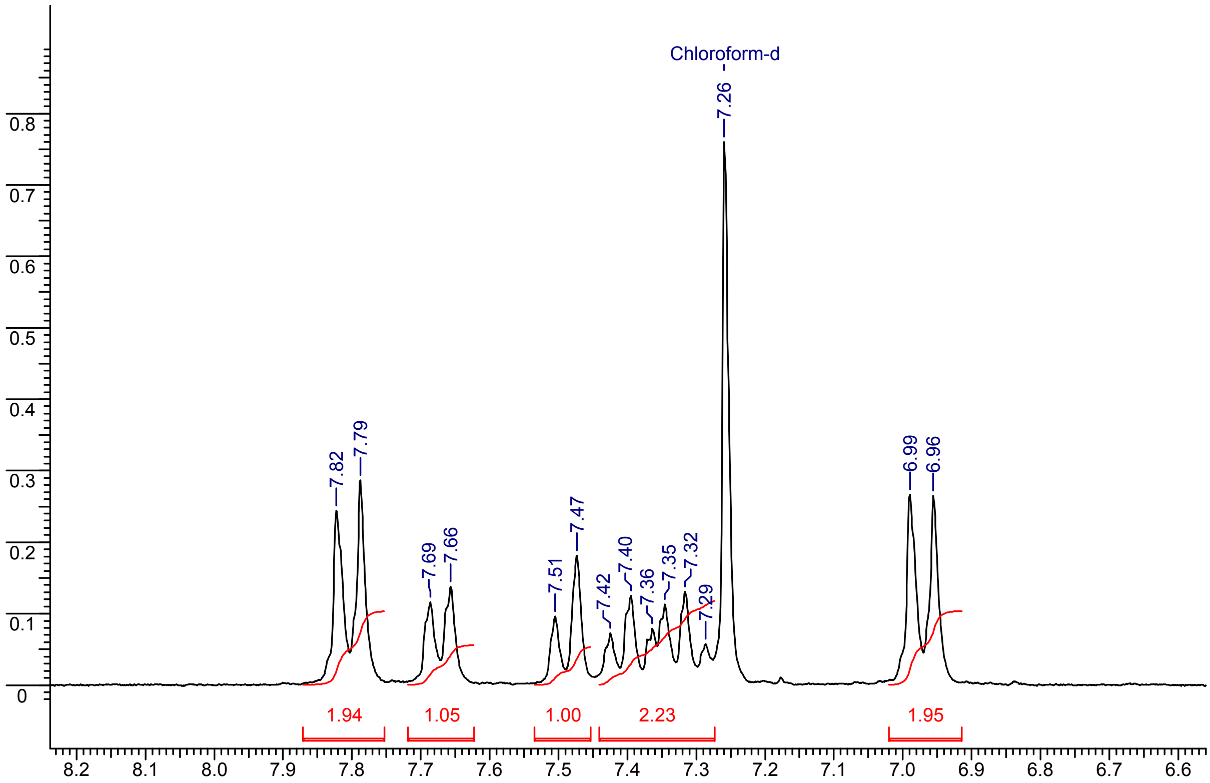

Figure S61. ^{1}H-NMR spectra of **8i**

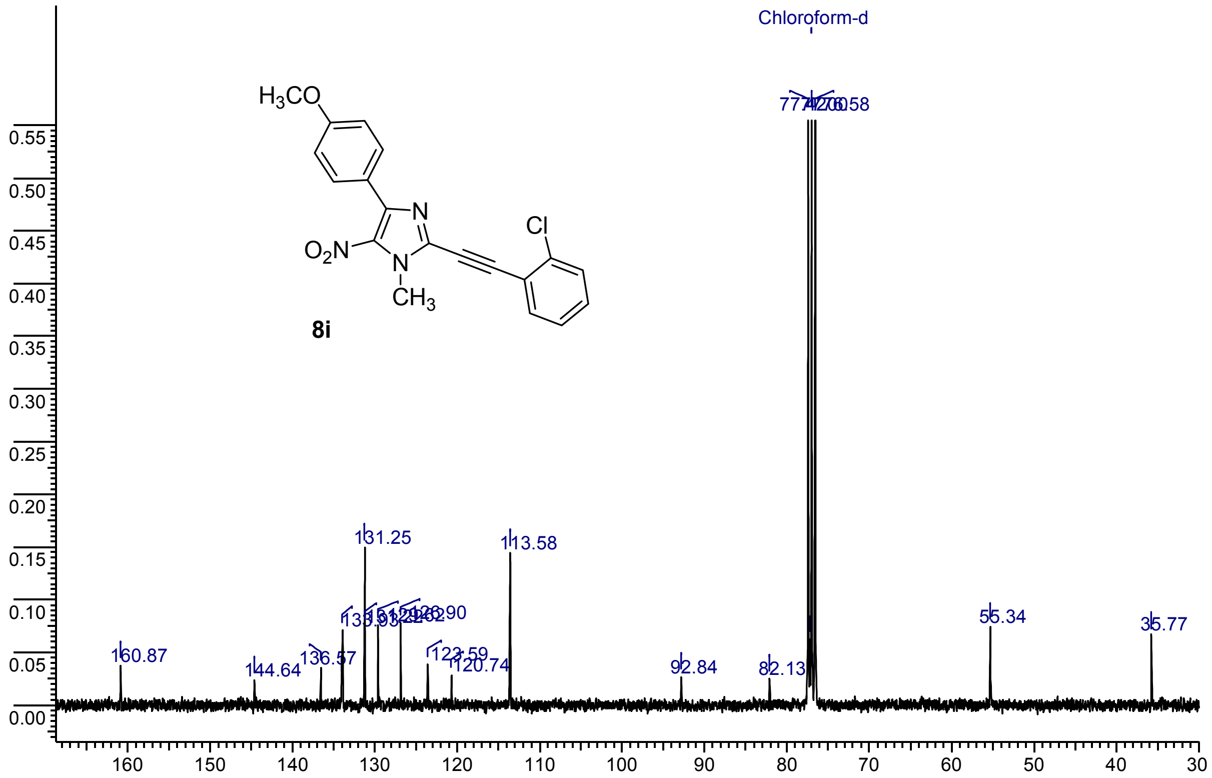

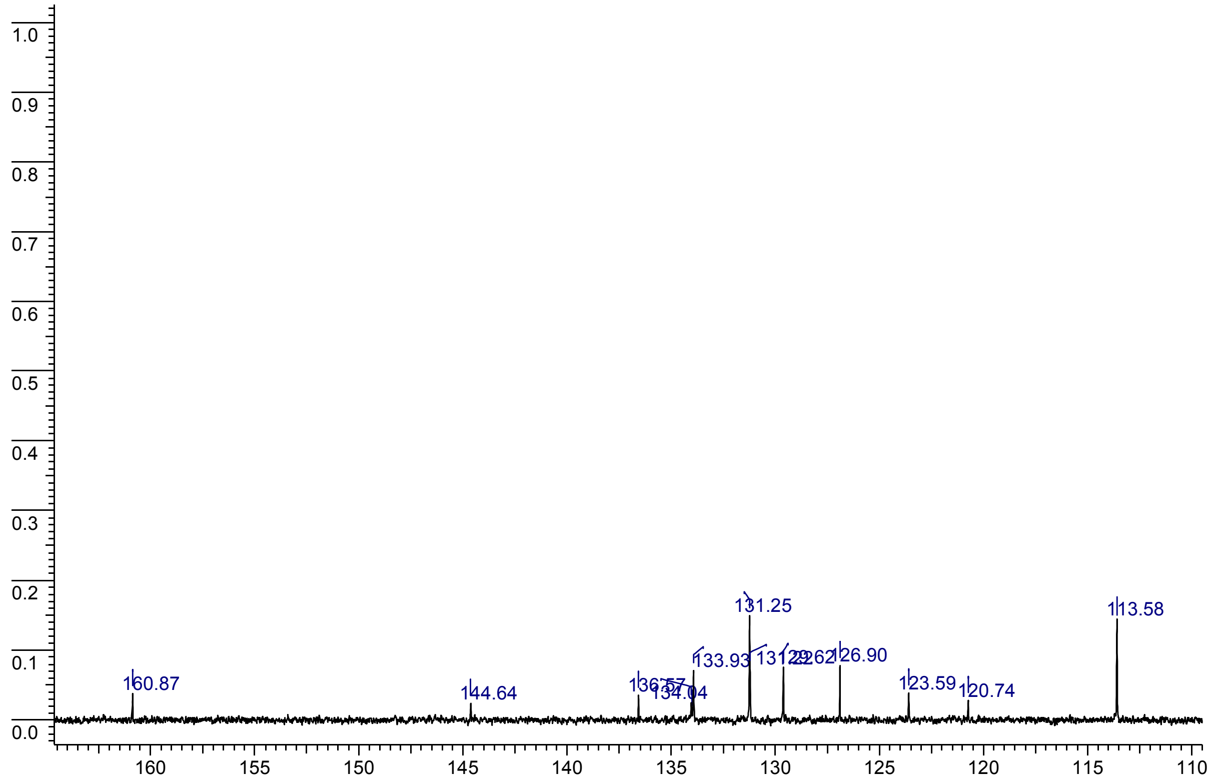

Figure S62. ^{13}C-NMR spectra of **8i**

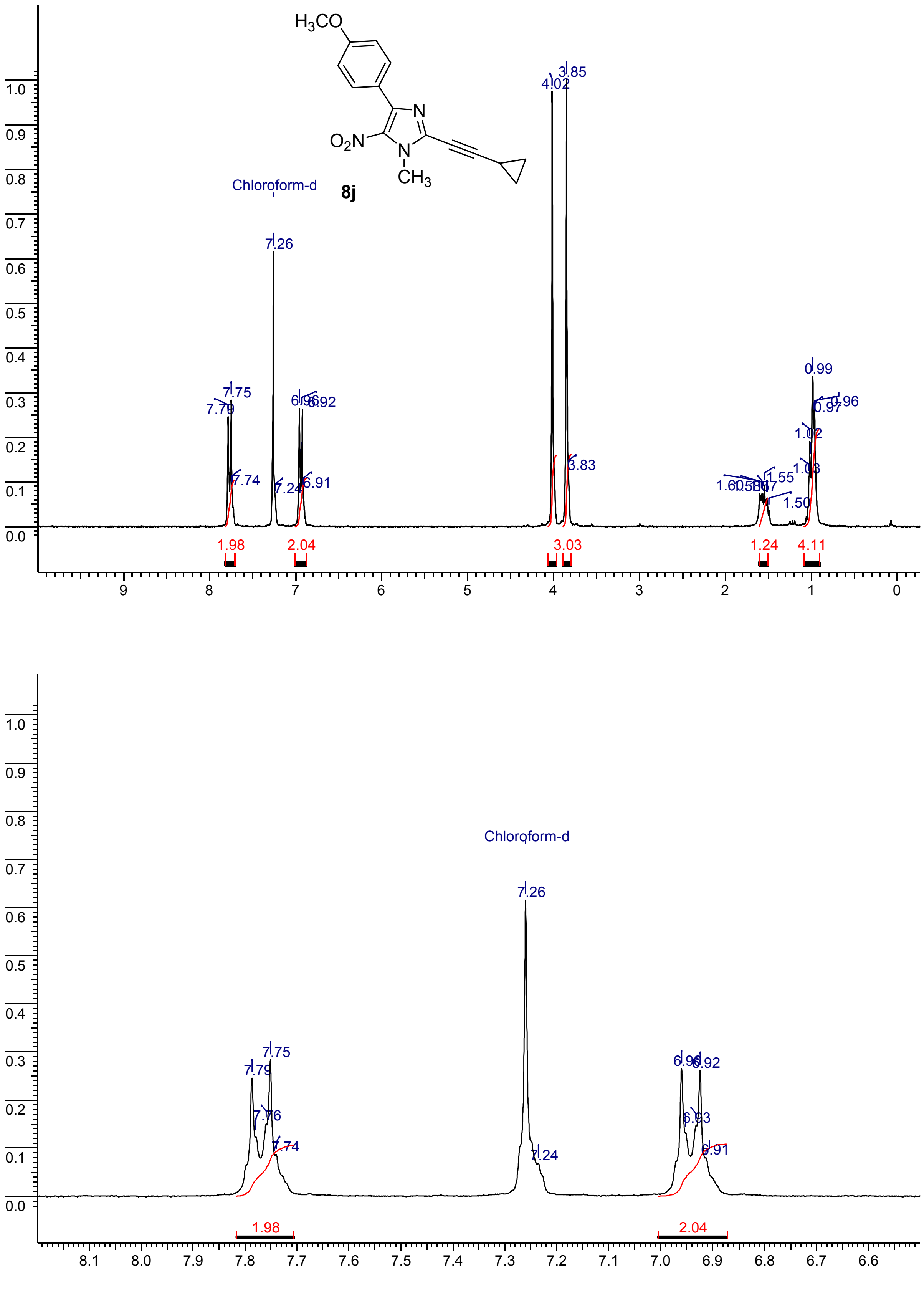

Figure S63. ^{1}H-NMR spectra of **8j**

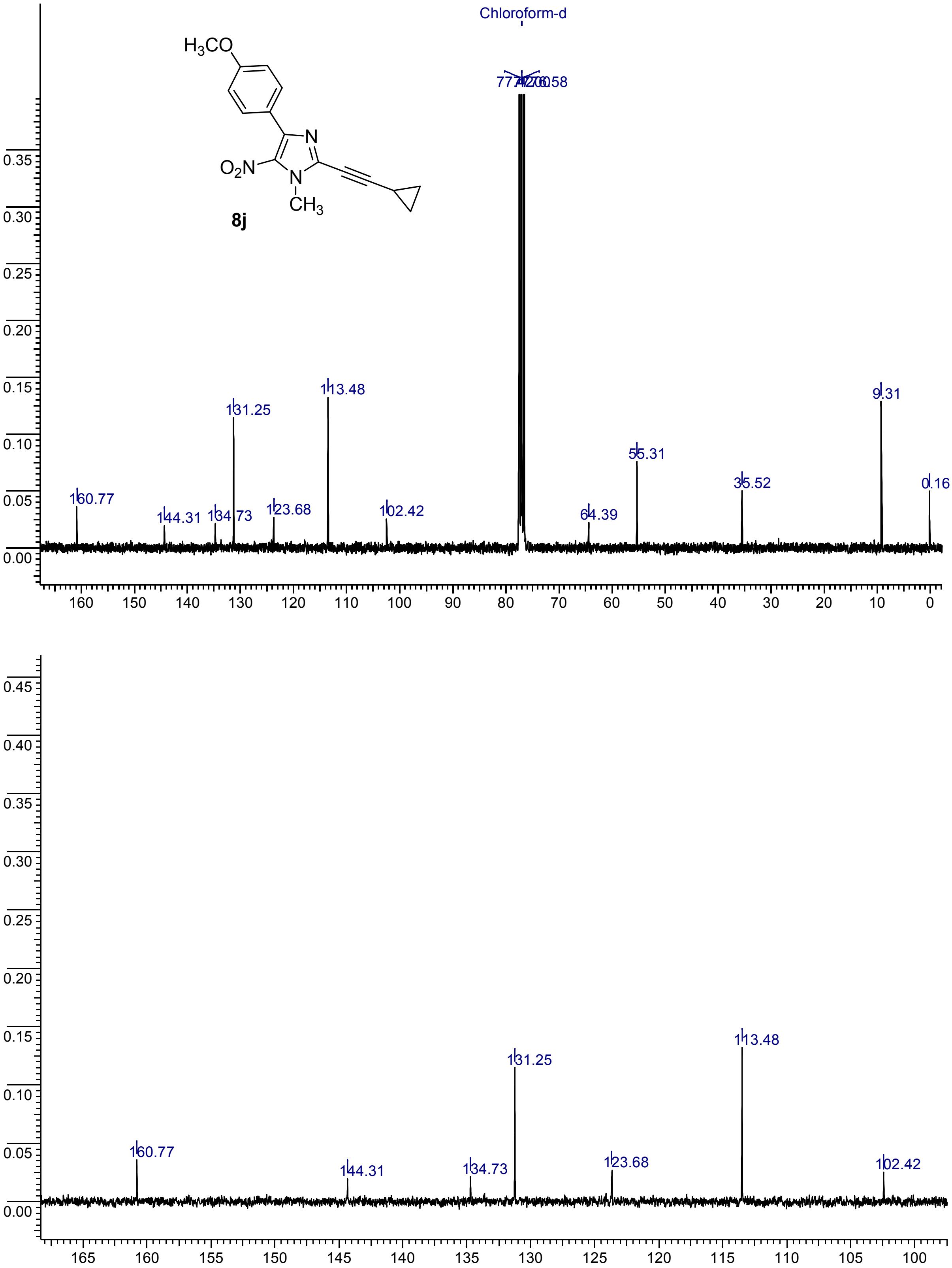

Figure S64. ^{13}C-NMR spectra of **8j**

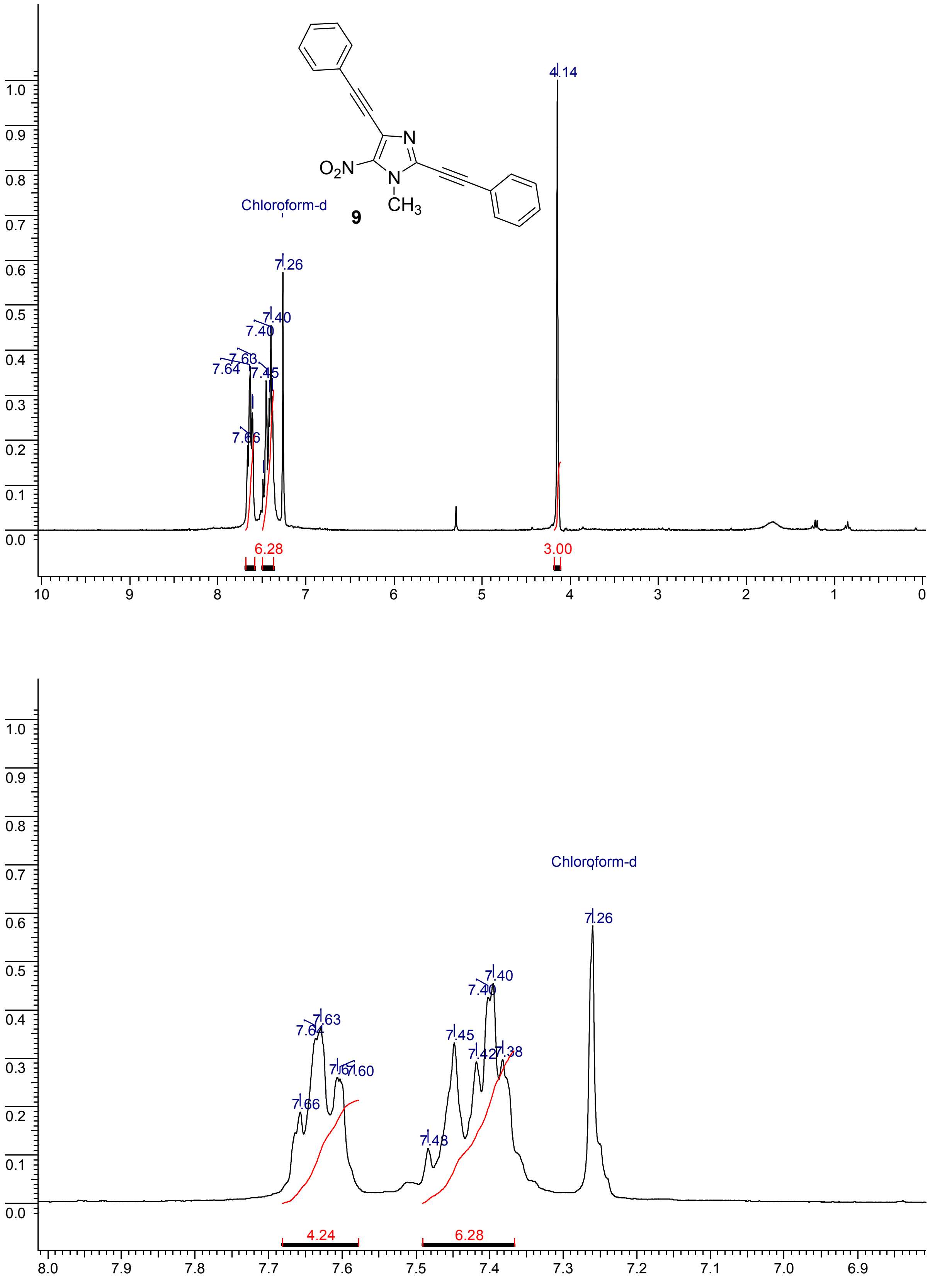

Figure S65. ^{1}H-NMR spectra **of 9**

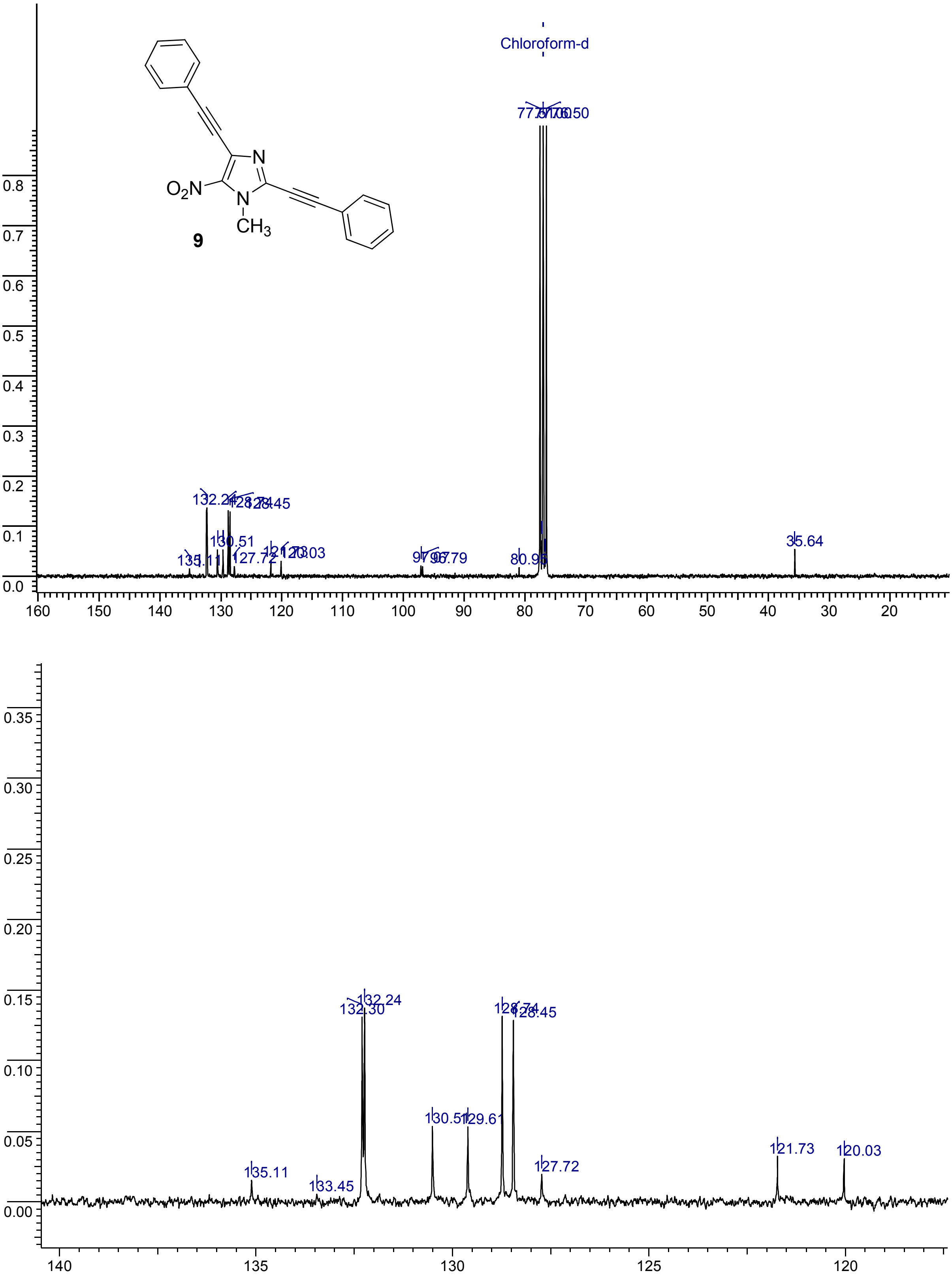

Figure S66. ^{13}C-NMR spectra of **9**
